# The complete mitochondrial genome of *Taxus cuspidata* (Taxaceae): eight protein-coding genes have transferred to the nuclear genome

**DOI:** 10.1186/s12862-020-1582-1

**Published:** 2020-01-20

**Authors:** Sheng-Long Kan, Ting-Ting Shen, Ping Gong, Jin-Hua Ran, Xiao-Quan Wang

**Affiliations:** 1 State Key Laboratory of Systematic and Evolutionary Botany, Institute of Botany, Chinese Academy of Sciences, Beijing, 100093 China; 2 University of Chinese Academy of Sciences, Beijing, 100049 China

**Keywords:** *Taxus cuspidata*, Mitogenome, Endosymbiotic gene transfer, RNA editing, Gymnosperms

## Abstract

**Background:**

Gymnosperms represent five of the six lineages of seed plants. However, most sequenced plant mitochondrial genomes (mitogenomes) have been generated for angiosperms, whereas mitogenomic sequences have been generated for only six gymnosperms. In particular, complete mitogenomes are available for all major seed plant lineages except Conifer II (non-Pinaceae conifers or Cupressophyta), an important lineage including six families, which impedes a comprehensive understanding of the mitogenomic diversity and evolution in gymnosperms.

**Results:**

Here, we report the complete mitogenome of *Taxus cuspidata* in Conifer II. In comparison with previously released gymnosperm mitogenomes, we found that the mitogenomes of *Taxus* and *Welwitschia* have lost many genes individually, whereas all genes were identified in the mitogenomes of *Cycas*, *Ginkgo* and Pinaceae. Multiple tRNA genes and introns also have been lost in some lineages of gymnosperms, similar to the pattern observed in angiosperms. In general, gene clusters could be less conserved in gymnosperms than in angiosperms. Moreover, fewer RNA editing sites were identified in the *Taxus* and *Welwitschia* mitogenomes than in other mitogenomes, which could be correlated with fewer introns and frequent gene losses in these two species.

**Conclusions:**

We have sequenced the *Taxus cuspidata* mitogenome, and compared it with mitogenomes from the other four gymnosperm lineages. The results revealed the diversity in size, structure, gene and intron contents, foreign sequences, and mutation rates of gymnosperm mitogenomes, which are different from angiosperm mitogenomes.

## Background

More than three thousand seed plant chloroplast genomes have been sequenced [1], but only 209 mitochondrial genomes (mitogenomes) are available for approximately 190 species of land plants (https://www.ncbi.nlm.nih.gov/genome/browse#!/organelles/, 09/12/2019) because plant mitogenomes are remarkably variable in both structure and sequence content [2]. Most (121) sequenced plant mitogenomes are from angiosperms. In contrast to the numerous sequenced angiosperm mitogenomes, however, mitogenomic sequences have been generated for only 6 gymnosperm species, i.e., *Cycas taitungensis* [3], *Ginkgo biloba*, *Welwitschia mirabilis* [4], *Pinus taeda* (direct submission, MF991879.1, http://www.ncbi.nlm.nih.gov/), *Picea abies* [5], and *Picea sitchensis* [6].

A comparison among the mitogenomes of *Cycas*, *Ginkgo* and *Welwitschia* showed that the *Cycas* and *Ginkgo* mitogenomes represent the ancestral mitogenome type in seed plants, which is small, has a low substitution rate, and possesses numerous genes, introns and RNA editing sites, whereas the *Welwitschia* mitogenome is relatively large, has a high substitution rate, and has lost many genes and introns [3, 4]. The mitogenome of three species of Pinaceae (*Picea abies*, ca. 4.90 MB; *Picea sitchensis*, 5.5 Mb; *Pinus taeda*, 1.19 Mb) is extremely expanded [5, 6] and larger than that of *Cycas*, *Ginkgo* and *Welwitschia* [3, 4]. Although all of these Pinaceae mitogenomes have gene and intron contents similar to those in the *Cycas* and *Ginkgo* mitogenomes [5–7], these mitogenomes from different gymnosperm lineages are different in many other aspects, such as genome structure, repeats, turnover rates, and foreign sequence ratios [4].

Based on the recent phylogenomic study of Ran et al. [8], gymnosperms represent five of the six main lineages of seed plants, namely, cycads, ginkgo, gnetophytes, Pinaceae and Conifer II (non-Pinaceae conifers or Cupressophyta) [9, 10]. These lineages diverged before the Jurassic and have diversified dramatically in morphological characters and molecular evolutionary rates [8, 11]. The six sequenced mitogenomes come from four lineages (cycads, ginkgo, gnetophytes and Pinaceae). Therefore, complete mitogenomes are available for all major seed plant lineages except Conifer II, an important lineage including six families (Araucariaceae, Cephalotaxaceae, Cupressaceae, Podocarpaceae, Sciadopityaceae, and Taxaceae). Conifer II includes approximately 380 species, which are widely distributed on all continents except Antarctica [12]. Previous morphological studies supported the sister relationship between Conifer II and Pinaceae. However, recent phylogenomic studies yielded a topology with Conifer II sister to Pinaceae + Gnetales [8]. Therefore, knowledge of mitogenomic features in Conifer II is essential for a comprehensive understanding of the evolution and diversification of gymnosperm mitogenomes.

In this study, we sequenced and analyzed the complete mitogenome of *Taxus cuspidata*, a species belonging to Taxaceae of Conifer II, and then compared it with published gymnosperm mitogenomes. By comparing mitogenomes from the five main lineages of gymnosperms, we aimed to reveal the diversity in size, structure, gene and intron contents, foreign sequences, and mutation rates in gymnosperm mitogenomes. This study will shed light on the evolution of plant mitogenomes.

## Methods

### Mitochondrial DNA isolation, total RNA extraction, sequencing and mitogenome assembly of *Taxus cuspidata*

Young leaves and seeds of *Taxus cuspidata* were collected from a female tree growing at the Institute of Botany, Chinese Academy of Sciences. Mitochondria were isolated from leaves by using density gradient centrifugation [13] and digested with DNase I (Promega, Madison, USA) to eliminate genomic DNA contamination. Total RNA was extracted from seeds after one day of germination using RNAplant Plus Reagent (Tiangen, Beijing, China) because almost all mitochondrial genes are highly expressed in germinating seeds [14] and then digested by DNase I.

To obtain a full-length mitogenome sequence and identify the comprehensive RNA editing sites, we used both short-read (Illumina) and long-read sequencing (Oxford Nanopore) technologies in this study. First, approximately two micrograms of mitochondrial DNA was sheared by using Megaruptor. A > 20-kb library was constructed by using the ONT Ligation Sequencing Kit 1D (SQK-LSK108) and sequenced using an Oxford Nanopore GridION X5 Sequencer following the manufacturer’s protocol. Second, approximately one microgram of mitochondrial DNA was sonicated to ~ 500 bp using the Covaris M220 system. The sonicated DNA was purified using a TIANgel Midi Purification Kit, and a sequencing library was constructed using the NEBNext® Ultra™ DNA Library Prep Kit for Illumina® (New England Biolabs, Ipswich, MA, England) according to the manufacturer’s instructions. In addition, approximately 5 μg of total RNA was used to construct the cDNA library (NEBNext Ultra Directional RNA Library Prep Kit for Illumina, Illumina, San Diego, CA). Libraries were sequenced using an Illumina HiSeq 2500 (Illumina) with paired-end reads of 150 bp for cDNA and 250 bp for DNA.

The short raw reads were checked with FastQC (http://www.bioinformatics.babraham.ac.uk/projects/fastqc/) and trimmed by Trimmomatic (ILLUMINACLIP:TruSeq-PE.fa:2:30:10 LEADING:3 TRAILING:3 MINLEN:20) [15]. The long raw reads were base-called by using Albacore v2.1.7 (mean_qscore > 7) with barcode demultiplexing.

The mitogenomes of higher plants are markedly variable in both structure and sequence content [2, 16–21]. To improve assembly reliability, we used two strategies to assemble the *Taxus* mitogenome. In the first strategy, we followed the assembly process of Gui et al. [22] and Ye et al. [23]. First, the short clean reads were de novo assembled with SPAdes v 3.13.0 [24] using multiple *k-mer* values [21, 33, 55, 77]. Second, potential mitochondrial contigs were extracted by aligning against the mitochondrial protein-coding genes of *Cycas taitungensis* [3] with BLAST v 2.3.0 [28]. Then, the putative long mitochondrial reads were baited by mapping the short reads to the plant mitogenome database (ftp://ftp.ncbi.nlm.nih.gov/refseq/release/mitochondrion/) and the potential mitochondrial contigs using BLASR v5.1 [29]. Finally, the putative long mitochondrial reads were assembled by Canu v1.7.1 [30]. In the second strategy, all short clean reads were assembled de novo by using Canu directly [30].

Subsequently, we used bowtie2 to map the short clean reads to the draft contigs and improved the draft contigs with Pilon v1.22 [31, 32]. Then, MUMmer was used to check whether these contigs were circular [33]. Finally, the corrected contigs obtained from the above two assembly strategies were aligned with each other using MUMmer 3.0 and MAFFT v. 7 [33, 34], and the result showed that these two contigs were identical. Based on the above assembly steps, we obtained a master circle of the *Taxus* mitogenome.

### Mitogenome annotation

Protein and rRNA genes of the *Taxus* mitogenome were annotated by tblastn using a local database of extracted gene sequences from *Cycas taitungensis*, *Ginkgo biloba* and *Welwitschia mirabilis* because *Cycas* and *Ginkgo* have all 41 protein-coding genes and three rRNA genes that are similar to those of the basal angiosperms [3, 4]. Then, we downloaded the mitogenome data of *Cycas taitungensis*, *Ginkgo biloba*, *Pinus taeda* and *Welwitschia mirabilis* from the National Center for Biotechnology Information (NCBI). We identified tRNA genes, introns, and open reading frames (ORFs) in all five mitogenomes (*Cycas taitungensis*, *Ginkgo biloba*, *Pinus taeda*, *Taxus cuspidata*, and *Welwitschia mirabilis*). tRNA genes were identified by tRNAscan-SE 2.0 [35], and group I and II introns were detected by the RNAweasel tool [25]. ORFs were predicted by ORF Finder (https://www.ncbi.nlm.nih.gov/orffinder/) with the standard genetic code and a minimal length of 102 nt, and ORFs longer than 300 bp were annotated by Blast2GO with default parameters [36]. The circular mitogenome map was drawn with OGDRAW [37].

### RNA editing site identification

RNA editing sites of the *Taxus* mitogenome were identified by RES-Scanner, a powerful software that provides comprehensive identification and annotation of RNA editing sites with the short clean reads of the genome and transcriptome as input files [38]. The editing efficiency of each site was estimated by calculating the proportion of cDNA reads that contained the edited nucleotide, and the minimum number of DNA and RNA reads required for determining RNA editing sites was set to ten and three (the default parameters in RES-Scanner), respectively. In addition, similar to Guo et al. [4], we predicted RNA editing sites in all five mitogenomes using PREP-Mt [39], with a cutoff value of 0.2, so that we could compare the evolutionary patterns of the mitochondrial RNA editing sites in gymnosperms.

### Identification of genes that have been transferred to the nuclear genome in *Taxus* and *Welwitschia*

We used the depth of sequencing coverage and real-time PCR to detect whether some protein-coding genes have transferred to the nuclear genome in *Taxus*. The depth of sequencing coverage of each gene was calculated with Bowtie2 v 2.2.9 [32] and SAMtools v 1.6 [40], using the short clean reads of the genome as the input file. Absolute quantitative real-time PCR was used to quantify the copy number of all 41 protein-coding genes (excluding *rpl*10, for which no homologous sequence was found by blast or PCR) in *Taxus*, with the single-copy nuclear gene *LEAFY* as an experimental control. The digested genomic DNA was used as the input DNA. All primers are listed in Additional file 1: Table S1. PCRs were conducted using a SYBR® Premix Ex Taq™ Kit (TaKaRa), and melting analysis was routinely performed to check the identity of PCR products. The detailed experimental and analytical protocols were similar to those of Ran et al. [41]. For *Welwitshcia*, we searched mitochondrial homologs in the transcriptome [8] using all mitochondrial genes of *Cycas* and *Pinus* as queries.

### Identification of repeats, tandem repeats, *Bpu* elements and foreign sequences

Repeats were detected by ROUSFinder.py, and tandem repeats were identified using Tandem Repeats Finder with default parameters [42]. Plastid-derived mtDNA (MTPTs) and *Bpu*-like elements were identified following the procedure of Guo et al. [4]. Briefly, MIPTs were identified by using blastn, and the sequences matching genes that occurred in the mitochondrial and plastid genomes simultaneously (i.e., *atp1*/*atpA*, *rrn26*/*rrn23*, and *rrn18*/*rrn16*) were excluded. *Bpu*-like elements were identified by blastn using the *Cycas Bpu* consensus sequence as a query. In addition, the nuclear-derived repetitive sequences were identified by using the RepeatMasker web server (http://www.repeatmasker.org/cgi-bin/WEBRepeatMasker).

### Shared DNAs and gene cluster analyses

To determine mtDNA shared between species, each pair of mitogenomes was searched using blastn with a word size of 7 and an e-value cutoff of 1 × 10^− 6^. Syntenic relationships were generated using Circos v. 0.69 [43]. To evaluate the conservation of gene order, we searched for gene clusters shared by gymnosperms by simple visual inspection.

### Evolutionary rate heterogeneity test

All mitochondrial protein-coding genes were obtained from the five gymnosperms, three basal angiosperms [*Amborella trichopoda* (KF754799, KF754800, KF754801, KF754802, and KF754803), *Liriodendron tulipifera* (NC_021152), and *Nymphaea colorata* (NC_037468)], and two fern species [*Ophioglossum californicum* (NC_030900) and *Psilotum nudum* (KX171638 and KX171639)], and putative transferred genes were retrieved from the transcriptome data for *Taxus cuspidata* and *Welwitschia mirabilis* [8]. Sequence alignment, unreliable sequence alignment filtering, synonymous (*d*_*S*_) and nonsynonymous (*d*_*N*_) length calculations, and absolute nonsynonymous (*R*_*N*_) and synonymous rate (*R*_*S*_) calculations were similar to those in Ran et al. [8]. To mitigate the confounding effects of C-to-U RNA editing on substitution-rate calculations and phylogenetic reconstruction, the predicted editing sites were excluded in the sequence alignments. The phylogenetic topology and divergence times among gymnosperms were obtained from Ran et al. [8].

## Results

### Mitogenome size and gene and intron contents of *Taxus cuspidata*

**Fig. 1:**
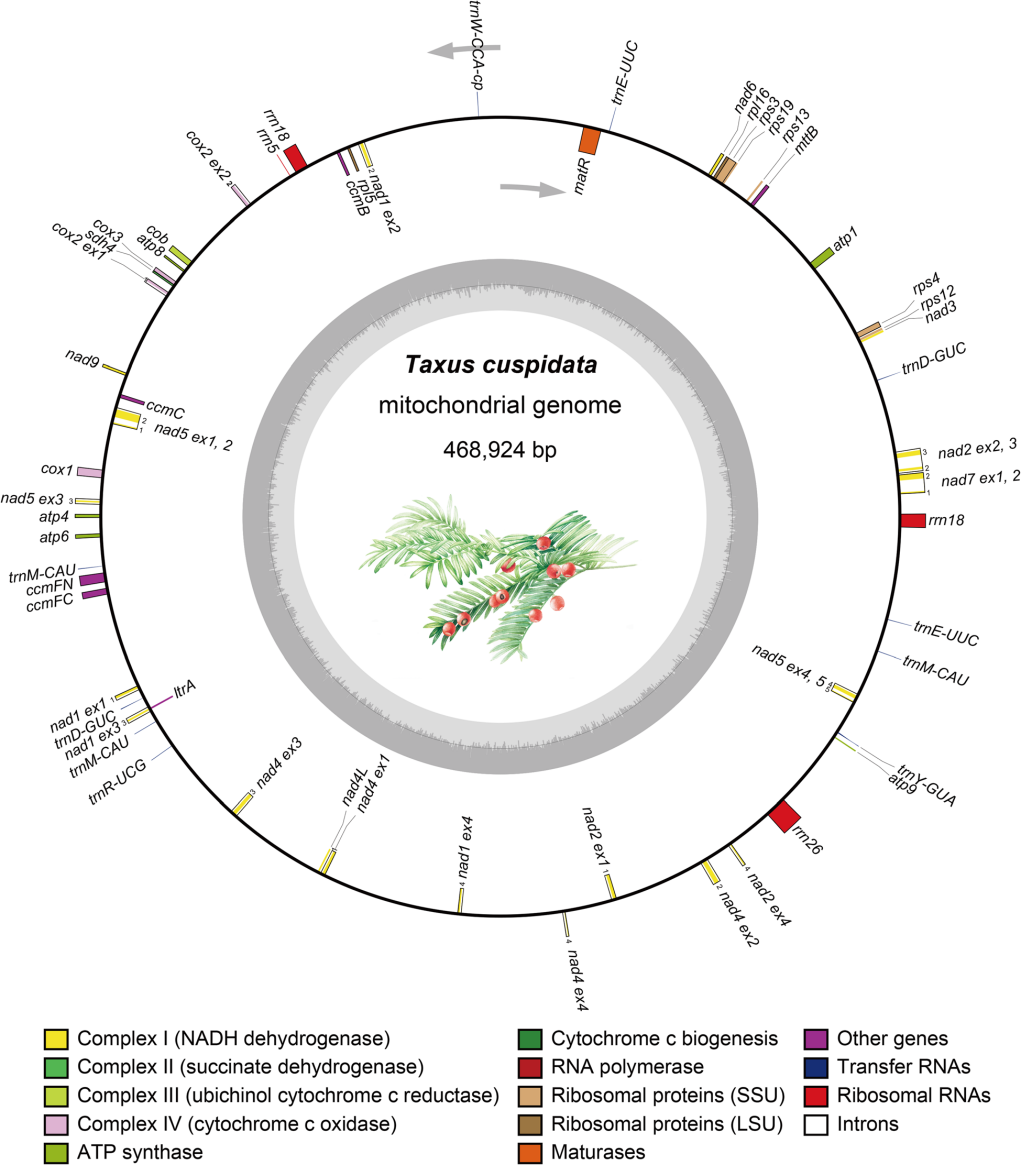
Gene map of the complete mitogenome of *Taxus cuspidata*. Genes inside and outside the outer circle are transcribed in clockwise and counterclockwise directions, respectively. The inner circle represents GC content (%)

The mitogenome of *Taxus cuspidata* was assembled as a single circular molecule (Fig. 1) with a size of 468,924 bp. The mitogenome contains 46 genes, including 32 for proteins, ten for tRNAs (three of them have two copies, respectively), and three for rRNAs. Among the 32 protein-coding genes, there are five and two encoding small and large subunit ribosomal proteins, respectively; nine, one, one, three, and five encoding mitochondrial respiratory chain complexes I, II, III, IV, and V, respectively; four involved in cytochrome C biogenesis; one for transport membrane protein, and one for maturase-related protein (Additional file 2: Table S2). No group I introns were detected in the *Taxus* mitogenome. In contrast, 15 group II introns were found in the *cox2*, *nad1*, *nad2*, *nad4*, *nad5* and *nad7* genes, of which four and eleven were *cis*- and *trans*-spliced, respectively (Fig. 1). Among the ten tRNA genes, nine were mitochondrially native, and one was derived from plastids. Among the three rRNA genes, *rrn5* and *rrn26* had one copy, and *rrn18* had two copies. The lengths of the *Taxus* mitochondrial genes, exons, and introns ranged from 225 to 4104 bp, 22 to 1224 bp, and 804 to 2461 bp, respectively. Detailed information on the *Taxus cuspidata* mitochondrial genes, exons, and introns is provided in supplementary Additional file 2: Table S2.

### Variation in gene and intron contents in the gymnosperm mitogenomes

**Fig. 2:**
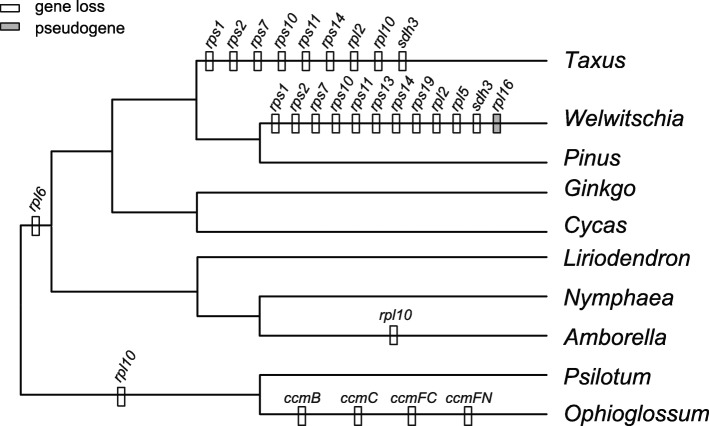
Loss and pseudogenes of mitochondrial protein-coding genes in selected gymnosperms, angiosperms and fern species. Gray and white bars represent events of pseudogenization and gene loss, respectively. The topology is based on Ran et al. [8] and the Angiosperm Phylogeny Website (http://www.mobot.org/mobot/research/apweb/)

We compared the mitogenomes of *Taxus*, *Pinus*, *Welwitschia*, *Cycas* and *Ginkgo*, representing all five lineages of gymnosperms. The genome sizes of *Pinus* and *Welwitschia* are 1.19 Mb and 979 kb, respectively, which are larger than those of *Taxus* (429 kb), *Cycas* (414 kb) and *Ginkgo* (346 kb). *Cycas*, *Ginkgo* and *Pinus* have 41 mitochondrial protein-coding genes, whereas only 32 and 29 such genes were found in the *Taxus* and *Welwitschia* mitogenomes (Table 1). Eight genes were lost in both *Taxus* and *Welwitschia*, one was lost only in *Taxus*, and four were lost only in *Welwitschia* (Fig. 2). Similar to angiosperm mitogenomes, the gymnosperm mitogenomes contain three kinds of rRNA genes (*rrn5*, *rrn16*, and *rrn26*) (Table 2). In addition, the number of tRNA genes varies greatly among these gymnosperm mitogenomes. *Cycas* and *Ginkgo* contain 27 and 23 tRNA genes for 17 and 16 amino acids, respectively. However, in the mitogenomes of *Pinus*, *Welwitschia* and *Taxus*, only twelve, eight and ten tRNA genes transporting ten, eight and seven amino acids, respectively, were found (Table 2).

**Table 1:** General features of five gymnosperm mitogenomes

	*Cycas*	*Ginkgo*	*Pinus*	*Welwitschia*	*Taxus*
Accession	AP009381	KM672373	MF991879.1	KT313400	MN593023
Size (bp)	414,903	346,544	1,191,054	978,846	468,924
GC%	46.9	50.4	47	53	50.39
Genes	71	67	59	40	46
tRNAs	27	23	12	8	10
rRNAs	3	3	6	3	4
Protein coding	41	41	41	29	32
ORF	3945 (414,858 bp)	3944 (323,967 bp)	10,587 (1,191,015 bp)	11,171 (978,799 bp)	3923 (468,857 bp)
Introns	26	25	26	10	15
Predicted edit sites	1206	1306	1179	225	1102
Repeats (kb)	80 (19.2%)	32 (9.3%)	170 (14.2%)	50 (5.0%)	62 (13.2%)
Tandem repeats (kb)	22 (5.3%)	3.6 (1.1%)	71 (6.0%)	24 (2.5%)	48 (10.2%)
Plastid-derived (kb)	19 (4.6%)	0.3 (0.1%)	5.6 (0.5%)	7.9 (0.8%)	0 (0%)
Nuclear-derived repetitive (kb)	3.4(0.8%)	1.9(0.6%)	5.3(0.5%)	2.5(0.3%)	3.5(0.8%)

**Table 2:** Mitochondrial RNA genes in Gymnosperms

Gene	*Cycas*	*Ginkgo*	*Pinus*	*Welwitschia*	*Taxus*
*rrn5*	1	1	3	1	1
*rrn18*	1	1	1	1	2
*rrn26*	1	1	2	1	1
*trnC-GCA*	1	1	0	0	0
*trnD-GUC*	1	1	1	1	2
*trnE-UUC*	1	1	1^c^	1	2
*trnF-GAA*	1	2	0	0	0
*trnG-UCC*	0	1^c^	0	0	0
*trnG-GCC*	1	0	0	0	0
*trnH-GUG*	0	0	1	0	0
*trnH-GUG-cp*	1	1	0	0	0
*trnK-UUU*	1	1^c^	1	0	0
*trnL-CAA*	1^a^	0	0	0	0
*trnL-UAA*	1^b^	2	0	0	0
*trnL-UAG*	1	1	0	0	0
*trnfM-CAU*	4	1	2	0	2
*trnfM-CAU-cp*	0	0	0	1	0
*trnM-CAU-cp*	2	1	0	0	0
*trnN-GUU*	1^d^	0	0	0	0
*trnP-AGG*	1	1	0	0	0
*trnP-UGG*	1^c^	1	2	0	0
*trnQ-UUG*	1^c^	1	1	1	0
*trnR-ACG-cp*	0	0	0	1	0
*trnR-UCU*	1^c^	1	0	0	0
*trnR-UCG*	0	0	0	0	1
*trnS-GCU*	1	1	0	0	0
*trnS-GGA-cp*	1^b^	0	0	0	0
*trnS-UGA*	1	1	0	0	0
*trnW-CCA*	1	2	1	0	0
*trnW-CCA-cp*	0	0	0	1	1
*trnY-GUA*	1	1	1	1	1
Total rRNA	3	3	6	3	4
Total tRNA	27	23	12	8	10

^a^previously un-annotated tRNA genes; ^b^ tRNA gene(s) containing intron(s); ^c^ Anticodon is inferred to be edited; ^d^ tRNA genes characterized as a “chlamydial” copy

Homologous transcripts of eight of the nine lost mitochondrial genes (excluding *rpl*10) were found in the transcriptome of *Taxus cuspidata* (GenBank accession numbers: MN886610-MN886617). Regardless of whether the universal primers [44] or specific primers we designed based on other sequences from gymnosperms were used, we failed to amplify the *rpl*10 gene in *Taxus*. The average sequencing coverage depth of the eight genes is much lower than that of other mitochondrial genes, and their copy numbers are similar to those for the single-copy nuclear gene *LEAFY* and much lower than those of other mitochondrial genes (Additional file 3: Figure S1). In addition, at least four genes (*rps*2, *rps*7, *rps*10 and *rps*11) have acquired one to two introns, showing different gene structures from their mitochondrial counterparts in *Cycas*, *Ginkgo* and *Pinus* (Additional file 4: Figure S2). Moreover, using all mitochondrial genes of *Cycas* and *Pinus* as queries, we found that eleven possible nuclear homologs (*rps1*, *rps2*, *rps7*, *rps10*, *rps11*, *rps13*, *rps14*, *rps19*, *rpl2*, *rpl5*, and *sdh3*) had been reported lost in the mitogenome of *Welwitschia* [4].

The mean GC content of the mitochondrial protein-coding genes of *Taxus* is much higher than that of the other four gymnosperms. In addition, the mean values of the GC, GC1, and GC2 contents of the mitochondrial protein-coding genes of *Welwitschia* are lower than those of the other four gymnosperms, but the GC3 content is similar to that of *Cycas*, *Ginkgo*, and *Pinus* and lower than that of *Taxus* (Additional file 5: Figure S3).

**Fig. 3:**
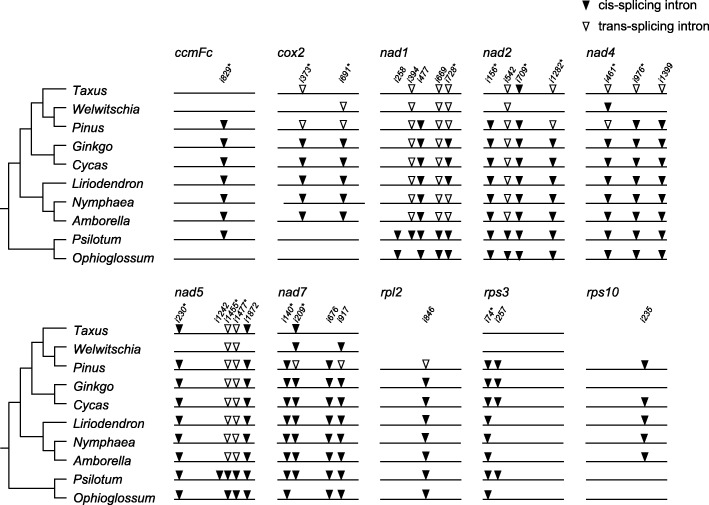
Comparison of mitochondrial introns among the studied plants. The arrowhead indicates the position of an intron insertion. Solid and hollow triangles represent *cis*- and *trans*-spliced introns, respectively. The asterisk indicates that the intron was acquired before the divergence of nonvascular and vascular plants

The detailed intron information for the five gymnosperm mitogenomes is shown in Fig. 3. *Cycas* has 26 introns, of which 21 are *cis*-spliced and five are *trans*-spliced. In comparison with *Cycas*, *Ginkgo* lost one intron (*rps10i235*). Simialr to *Cycas*, *Pinus* also have 26 introns, of which eight introns were converted from *cis*- to *trans*-spliced. In addition, the *Taxus* and *Welwitschia* mitogenomes contained only 15 (four *cis*- and eleven *trans*-spliced) and ten (three *cis*- and seven *trans*-spliced) introns, respectively, which are fewer than that of the *Pinus* mitogenome. Furthermore, the mean intron sizes are similar among these five mitogenomes (Additional file 6: Figure S4).

### RNA editing site abundance and efficiency in the gymnosperm mitogenomes

**Fig. 4:**
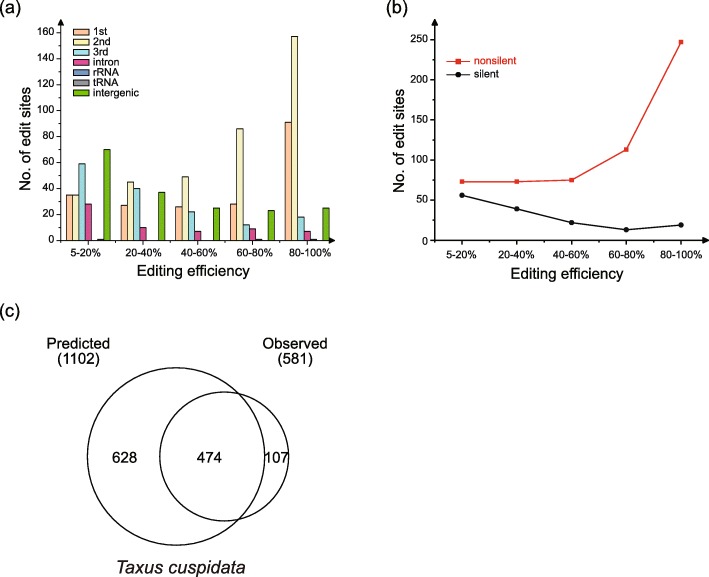
Frequency of RNA editing sites in the *Taxus* mitogenome. **a** Number of RNA editing sites with different editing efficiencies at the first, second and third codon positions; introns, rRNA and intergenic regions. **b** Number of RNA editing sites with different editing efficiencies at nonsilent and silent sites. **c** Comparison of the numbers of predicted and observed nonsilent RNA editing sites

By using RES-Scanner, we identified the exact number of RNA editing sites in the *Taxus* mitogenome. When the editing efficiency was set to 0.05, 974 C-to-U editing sites were detected. Most (791) of these editing sites were detected in protein-coding genes, of which 730 were in coding regions and 61 were in introns. In addition, two, one, and 180 editing sites were identified in rRNA, tRNA and intergenic regions, respectively (Additional file 7: Table S3 and Additional file 8: Table S4). Editing sites in the first and second codon positions have higher editing efficiencies than those in the third position, and nonsilent editing sites have higher editing efficiencies than silent sites (Fig. 4a and b).

**Fig. 5:**
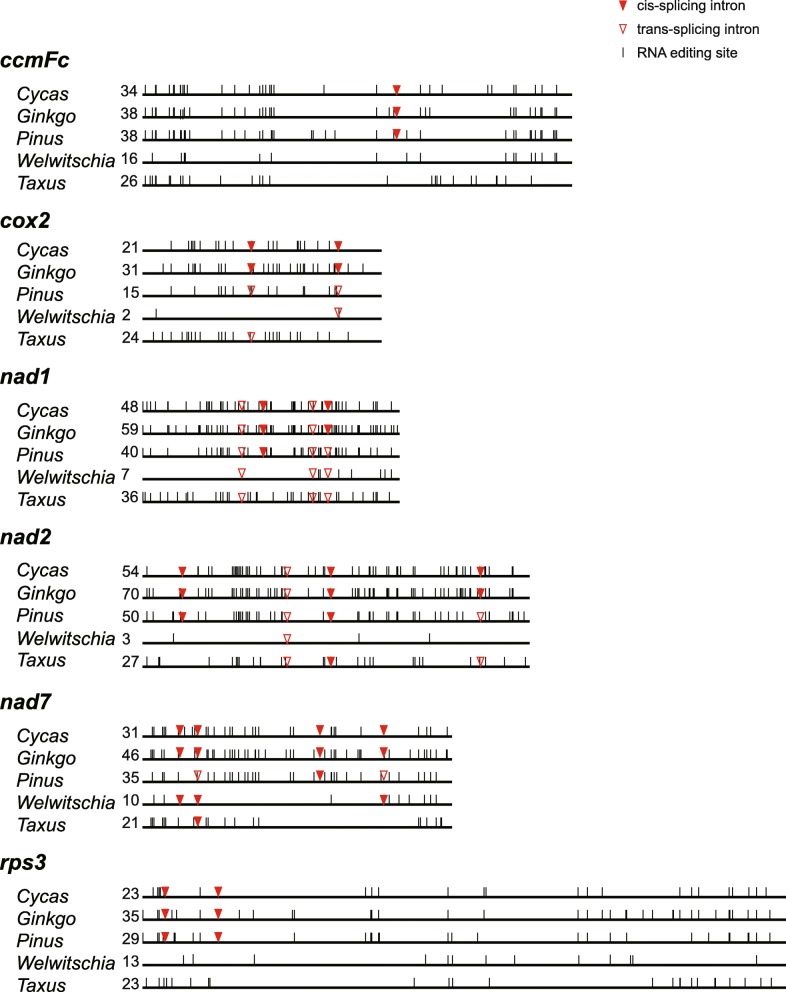
Localization of editing sites in exons of genes with intron losses in *Taxus* and *Welwitschia*. The red arrow represents the intron position

Because only nonsilent RNA editing sites in protein-coding genes could be predicted by using PREP-Mt, we compared the predicted editing sites with the empirically derived nonsilent editing sites. By using PREP-Mt with the cutoff score set to 0.2, 1102 C-to-U editing sites within the protein-coding genes of the *Taxus* mitogenome were predicted (Fig. 4c). However, only 474 were identical between the predicted and observed editing sites. Using the same cutoff score, we predicted the RNA editing sites in *Cycas*, *Ginkgo*, *Pinus* and *Welwitschia*. More than 1000 editing sites were found in *Cycas*, *Ginkgo* and *Pinus*, whereas only 225 editing sites were predicted in *Welwitschia* (Table 1). In *Welwitschia*, almost all genes have fewer editing sites than those in the other four mitogenomes. In *Taxus*, it is clear that genes with intron losses have fewer observed editing sites than their counterparts in *Cycas*, *Ginkgo* and *Pinus* (Fig. 5). In addition, we also used PREPACT 3.0 (Filter hits = 0.2) to predict the RNA editing sites [45], and the result showed that the numbers and positions of RNA editing sites predicted by PREP and PREPACT are similar (Additional file 9: Figure S5 and Additional file 10: Table S5).

### Structural and gene cluster dynamics in the gymnosperm mitogenomes

**Fig. 6:**
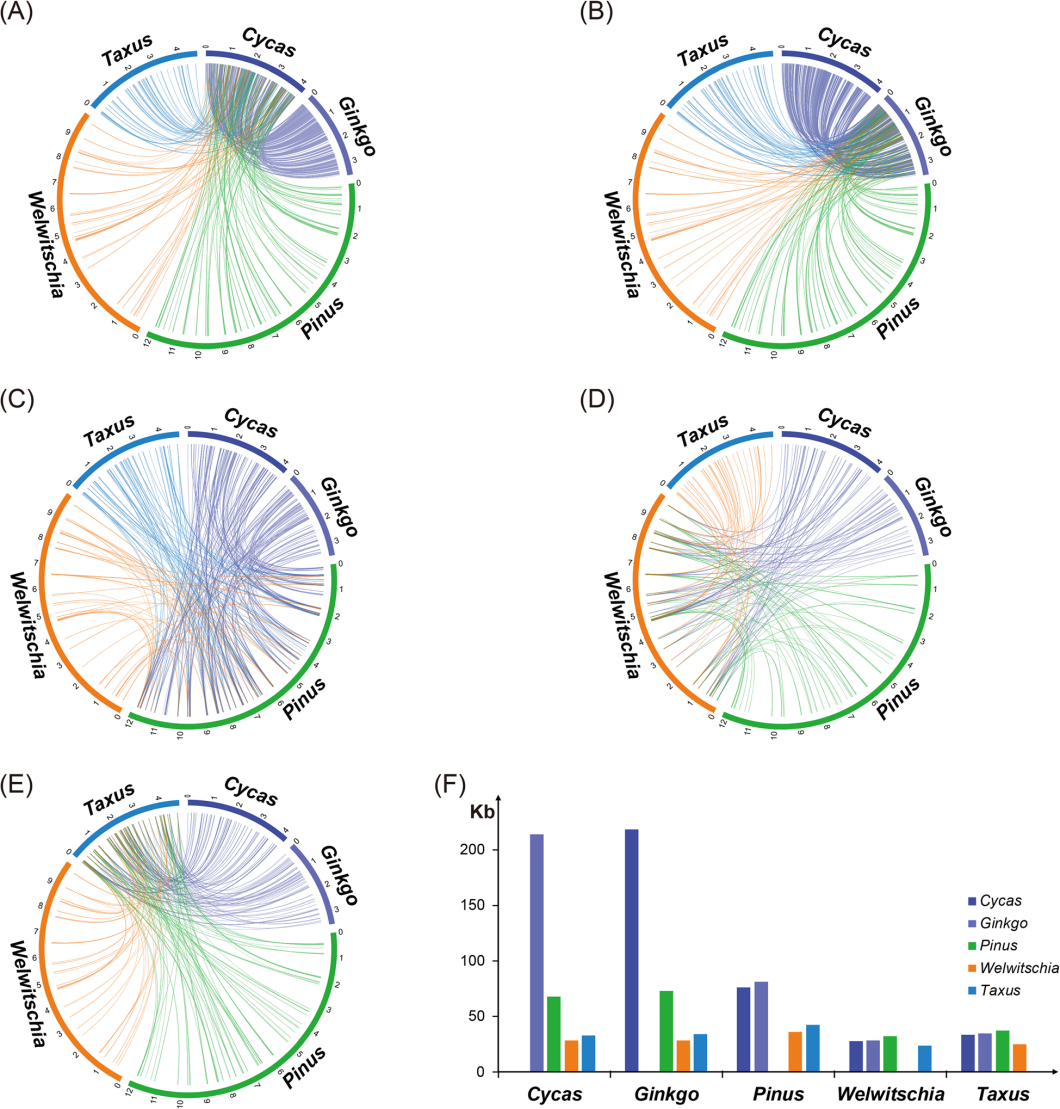
Syntenic block comparative analysis in gymnosperms generated using Circos. **a** Syntenic block of *Cycas* with the four other gymnosperms. **b** Syntenic block of *Ginkgo* with the four other gymnosperms. **c** Syntenic block of *Pinus* with the four other gymnosperms. **d** Syntenic block of *Welwitschia* with the four other gymnosperms. **e** Syntenic block of *Taxus* with the four other gymnosperms. **f** Shared sequence length of each species with the four other gymnosperms

A comparison of the syntenic blocks showed that the length of the DNA shared between *Cycas* and *Ginkgo* was up to 200 kb, approximately half the length of the mitogenomes of these two species. However, the lengths of the DNA shared among the other three species and between each of these three species and *Cycas* or *Ginkgo* were very short. For example, only approximately 50 kb were shared between *Pinus* and *Cycas* or between *Pinus* and *Ginkgo*, and only approximately 30 kb were shared between *Welwitschia* and the other four species and between *Taxus* and the other four species (Fig. 6).

Among the 29 conserved gene clusters identified in angiosperms [46], only one gene cluster (*nad3*-*rps12*) was shared by the five gymnosperm mitogenomes. In addition, only three were shared by *Cycas* and *Ginkgo*, and two were shared by *Cycas*, *Ginkgo* and *Pinus*. *Taxus*-*Ginkgo*, *Taxus*-*Welwitschia*, *Cycas*-*Ginkgo*-*Taxus*, and *Cycas*-*Ginkgo*-*Pinus*-*Taxus* each shared one cluster (Additional file 11: Figure S6).

### Repeats, tandem repeats, and foreign DNA sequences in the gymnosperm mitogenomes

**Fig. 7:**
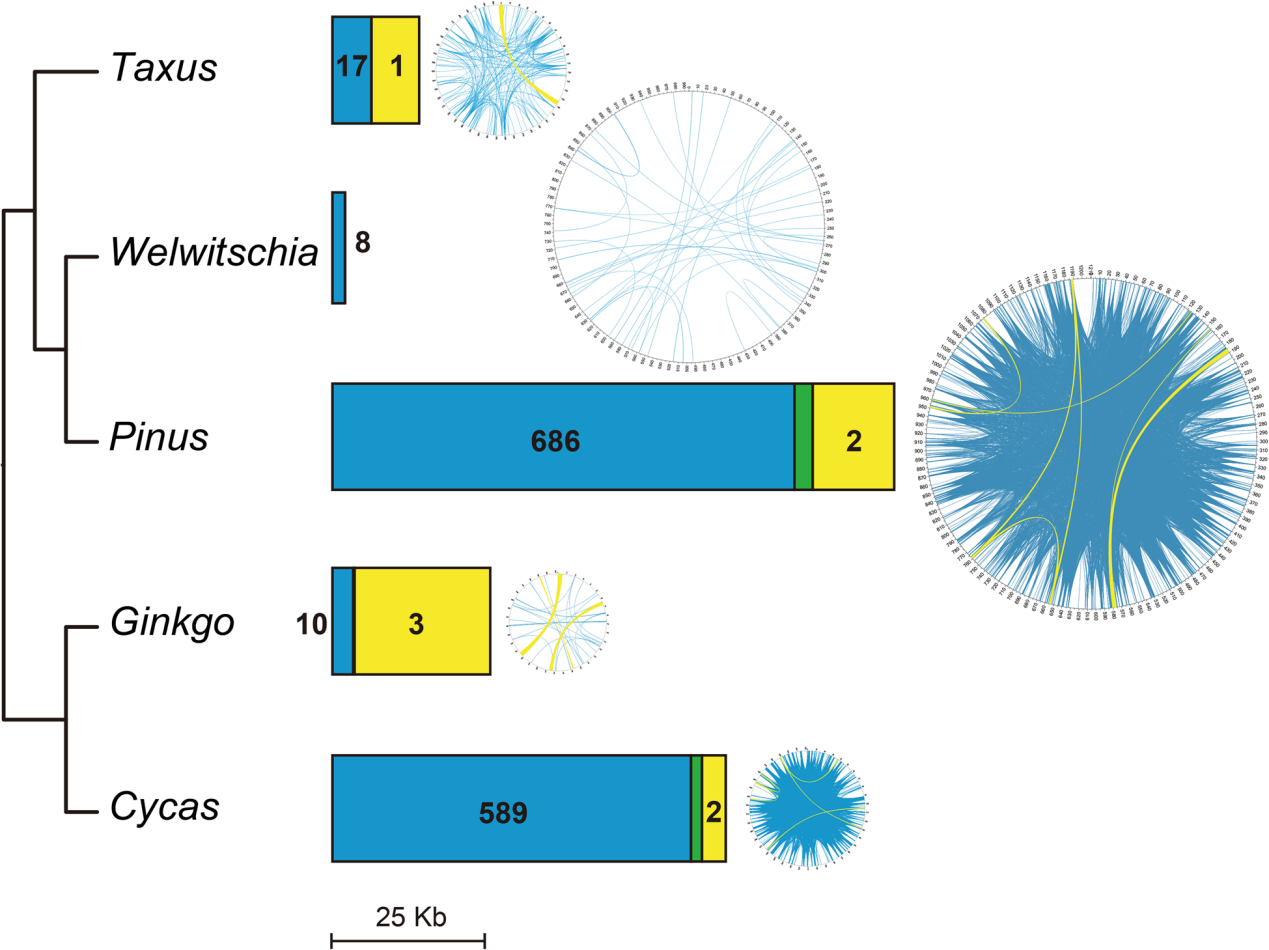
Length and distribution of repeats in gymnosperm mitogenomes. The bar shows the length of repeats, and the map shows the distributions of repeats. Yellow and blue represent large (> 1000 bp) and medium-sized (100–1000 bp) repeats, respectively, and green indicates the length of overlapping regions between large and medium-sized repeats

The *Cycas* and *Pinus* mitogenomes contain more dispersed repeats than those of the other three species (Table 1). A wealth of intermediate repeat pairs and a large number of small repeat pairs were identified in these two mitogenomes, and large repeats were found in all species except *Welwitschia* (Fig. 7). In addition, most repeats had more than two copies in the *Cycas* and *Pinus* mitogenomes (Additional file 12: Figure S7). Furthermore, the *Pinus* and *Taxus* mitogenomes contained more tandem repeat sequences (71 kb and 48 kb) than those of *Welwitschia*, *Cycas* and *Ginkgo* (24 kb, 22 kb, and 3.6 kb) (Table 1).

Plastid-derived sequences (length > 100 bp) were detected in *Cycas*, *Ginkgo*, *Pinus* and *Welwitschia* but were not found in *Taxus* after excluding sequences matching genes that occurred in the mitochondrial and plastid genomes simultaneously (i.e., *atp1*/*atpA*, *rrn26*/*rrn23*, and *rrn18*/*rrn16*) (Table 1). In addition, we also identified nuclear-derived repetitive sequences in the five mitogenomes, and two kinds of repeats (*copia* and *small RNA*) with a total length of 3.4 kb were identified in the *Cycas* mitogenome. Four kinds of repeats were found in the mitogenomes of *Ginkgo* (*CIN4*, *copia*, *gypsy*, and *small RNA*), *Welwitschia* (*copia*, *gypsy*, *DNA transposons* and *small RNA*) and *Taxus* (*copia*, *gypsy*, *DNA transposons* and *small RNA*), with lengths of 1.9 kb, 2.5 kb, and 3.5 kb, respectively. In addition, the *Pinus* mitogenome contains five kinds of repeats (*CIN4*, *copia*, *gypsy*, *DNA transposons* and *small RNA*) (Table 1 and Additional file 13: Table S6).

### Variation in nucleotide substitution rates among gymnosperm mitogenomes

The comparison of evolutionary rates of all mitochondrial protein-coding genes and eight putative transferred genes revealed that the synonymous substitution rate of the mitochondrial genes in *Welwitschia* and *Taxus* was higher. When genes were transferred to the nuclear genome, their synonymous and nonsynonymous substitution rates greatly increased. In addition, although eight putatively transferred genes in *Taxus* and *Welwitschia* were still found in the mitogenomes of *Cycas*, *Ginkgo* and *Pinus*, their synonymous and nonsynonymous substitution rates were higher than those of other mitochondrial genes (Additional file 14: Figure S8).

## Discussion

### Separate losses of multiple mitochondrial protein-coding genes in *Taxus* and *Welwitschia*

The transfer of functional mitochondrial genes to the nucleus is a frequent, ongoing process during plant evolution that has played a major role in cytonuclear interactions and mitogenome evolution [47–51]. The tempo of mitochondrial gene loss in plants is punctuated [47, 48, 52]. Only two genes were lost in the first approximately 300 myr of land plant evolution if maturase is not considered, and parallel gene losses documented in hornworts, lycophytes, and ferns also happened in more recent times [47, 52, 53]. In angiosperms, a large number of protein-coding genes have been lost in some lineages, although most of the oldest groups still exhibit near stasis in mitochondrial gene content [46, 47, 54, 55].

Previous studies showed that the *Cycas*, *Ginkgo*, *Picea* and *Pinus* mitogenomes contain 41 protein-coding genes [3–6, 56]. However, *Welwitschia mirabilis* lost eleven protein-coding genes [4], including ten ribosomal protein genes (*rpl*2, *rpl*5, *rps*1, *rps*2, *rps*7, *rps*10, *rps*11, *rps*13, *rps*14, and *rps*19), and the *sdh*3 gene. It seems that gene loss is an uncommon phenomenon in gymnosperm mitogenomes because gene loss has been detected in only one of the four lineages [5]. However, it is intriguing that nine protein-coding genes (*rpl*2, *rpl*10, *rps*1, *rps*2, *rps*7, *rps*10, *rps*11, *rps*14, and *sdh*3) have been lost from the newly sequenced *Taxus cuspidata* mitogenome. Except *rpl*10, these genes lost from the *Taxus* mitogenome were also absent from the *Welwitschia* mitogenome (Fig. 2). One may hypothesize that these genes were lost in the ancestor of *Taxus* and *Welwitschia*. However, mapping the lost genes onto the phylogeny of gymnosperms reveals that these genes could have been lost separately in the two species because Pinaceae contains all 41 protein-coding genes and Taxaceae is sister to *Welwitschia* + Pinaceae (Fig. 2). In addition, the genes lost from these two species are the most frequently lost genes in angiosperms [17, 47], implying that these genes could also be prone to loss in gymnosperms.

Whether genes missing from the mitogenome are completely lost or transferred to the nuclear genome is sometimes unknown. In theory, most or all lost mitochondrial genes are functionally transferred to the nucleus, such as *rpl5* being transferred to the nucleus in Poaceae [26]. However, frequent gene losses have also been reported for some species. For example, *rps*7 is one of the most frequently lost ribosomal protein genes, and it rarely appears to be functionally transferred to the nucleus [18, 57]. In combination with the genomic and transcriptomic sequences, we found that all mitochondrial genes missing from *Taxus* and *Welwitschia* (except *rpl10*) have been functionally transferred to the nucleus. Additionally, we found that compared to the rates in their mitochondrial counterparts, both the synonymous and nonsynonymous substitution rates of the transferred genes increased considerably (Additional file 14: Figure S8). Furthermore, in *Taxus*, four of the transferred genes have acquired one or two introns (Additional file 4: Figure S2).

### Separate losses of multiple mitochondrial tRNA genes in *Pinaceae*, *Taxus* and *Welwitschia*

Similar to angiosperms, all gymnosperms have three rRNA genes in their mitogenomes (Table 1). In contrast, the number of tRNAs differs greatly, with 27, 23, 12, 8 and 10 tRNAs in the *Cycas*, *Ginkgo*, *Pinus*, *Welwitschia* and *Taxus* mitogenomes, respectively (Table 2). After excluding plastid-derived tRNA genes due to their potential to be nonfunctional, only 23, 21, 12, 5, and 9 tRNA genes with 16, 15, 10, 5, and 6 amino acids remained, respectively, which seems to imply that tRNA genes have been lost in some lineages of gymnosperms. Considering that only four tRNA genes (*trnD-GUC*, *trnM-CAU*, *trnI-CAU* and *trnY-GUA*) are shared among all five gymnosperm mitogenomes and that *Cycas*, *Ginkgo*, and *Pinus* have some putative tRNA genes that other species do not have, we deduce that the mitogenome of the common ancestor of gymnosperms harbored many more tRNA genes than those of extant gymnosperms. Of course, we cannot rule out that some species have integrated some new tRNA genes by EGT (Table 2) [16].

### Frequent losses and *cis*- to *trans*-splicing of introns in the mitogenomes of gymnosperms

Both the ancestral angiosperm and gymnosperm mitogenomes contain 26 group II introns [3, 46]. In gymnosperms, *Cycas* and *Ginkgo*, have 26 and 25 introns, respectively, whereas only ten introns are found in *Welwitschia* [3, 4]. In addition, *Pinus taeda* contains 26 introns, and only 15 introns have been identified in the *Taxus* mitogenome (Fig. 3). Therefore, similar to in angiosperm mitogenomes [58–60], intron losses are more frequent than intron gains in gymnosperm mitogenomes.

Both *Taxus* and *Welwitschia* lost *ccmFc*i829, *nad*1i477, *nad*2i156, *nad*7i140/676, and *rps*3i74/257. We deduced that they lost these introns separately for the following reasons. First, the *nad*1i477 intron was found in *Gnetum* and *Ephedra*, the other two genera of gnetophytes, and *rps*3i74/257 were retained in *Gnetum* [41, 61]. Second, *Pinus taeda*, the sister group of *Welwitschia*, contains all of these introns (Fig. 3). When comparing the five gymnosperm mitogenomes, we found that an extremely large number of introns had converted from *cis*- to *trans*-spliced in *Pinus*, *Taxus* and *Welwitschia* (Fig. 3). This finding is consistent with evidence from other plant mitochondria, suggesting that the evolution of intron splicing patterns proceeds from *cis*- to *trans*-splicing [17, 62].

Previous studies suggested some possible mechanisms for intron loss, including genomic deletion, exonization, gene conversion, EGT, and retroprocessing [63]. Deletion can be ruled out because all introns in the *Taxus* and *Welwitschia* mitogenomes are precisely removed. Exonization is also impossible because the exon structures in all genes are intact and because no exonization has been detected in the plant mitogenome [64]. Gene conversion also seems impossible since no chimeric structures have been noted in any gene regions. EGT could be the reason for the losses of *rpl*2i846 and *rps*10i235 from the *Taxus* mitogenome because *rpl*2 and *rps*10 have been transferred to the nuclear genome. Considering the precision of the intron cut, the most likely mechanism of other intron losses from the *Taxus* mitogenome is retroprocessing [64, 65]. Retroprocessing, also known as a reverse transcriptase-mediated model, is the most frequently reported mechanism for the removal of introns [65–68]. Under this model, introns located at the 3′ ends of genes are more likely to be lost than those at the 5′ ends [63, 65, 69]. However, introns have been lost from the start or center of the genes *nad*1, *nad*2 and *nad*7 in *Taxus*, which is similar to the finding for *cox*1 in *Calypogeia* [63]. A mutational mechanism (e.g., internally primed reverse transcription) or selective pressure to maintain introns near the 5′ and 3′ ends of genes could explain this pattern of intron loss [70].

Usually, losses of introns are accompanied by the absence of editing sites in a gene [65]. However, based on transcriptome and genome high-throughput sequencing, the genes with instances of intron loss still have some RNA editing sites. Nevertheless, most genes with intron loss in *Taxus* and *Welwitschia* have fewer RNA editing sites than their counterparts in *Pinus*, *Cycas* and *Ginkgo* (Fig. 5). This discrepancy could be caused by a partially processed cDNA undergoing conversion with the native intron-bearing gene. As a result, although introns are removed, some RNA editing sites still remain [71]. Another possibility is that the full-length cDNA molecules have partially recombined with the native gene. The third possibility is that microconversion is responsible for the partial loss of edited sites [72]. The last possibility is that RNA editing resumed again after retroprocessing [73].

In our previous study, we reported the rapid evolution of the retroprocessed mitochondrial *rps*3 gene in Conifer II [41]. We did not find an RNA editing site in the *rps*3 gene of Conifer II. However, in this study, we found 23 RNA editing sites with an editing efficiency greater than 5% in the *Taxus rps*3 gene. This may have occurred because only a partial gene without an RNA editing site was chosen in the previous RT-PCR experiment or because of a difference in the number of edited sites between organs [74, 75], as needles were used in the previous study but seeds were used in this study.

### Gene clusters could be less conserved in gymnosperms, and transposable elements and specific repeats are rare in the *Taxus* and *Welwitschia* mitogenomes

Richardson et al. [46] described the distribution of 29 colinear gene clusters among angiosperm mitogenomes, 14 of which were assumed to be ancestral in angiosperms. However, in gymnosperms, eight gene clusters were found, and only one (*nad3*-*rps12*) was conserved in all five gymnosperm mitogenomes (Additional file 11: Figure S6). In addition, considering that at least seven gene clusters occur in the sampled angiosperms [46], but only two occur in *Welwitschia* and four in *Pinus* (Additional file 11: Figure S6), mitochondrial gene clusters in gymnosperms could be less conserved than in angiosperms. Seven gene clusters are conserved in the *Cycas* and *Ginkgo* mitogenomes, supporting their close relationship. The loss of the *cox3*-*sdh4* and *rrn18*-*rrn5* clusters seems to support the sister relationship between *Pinus* and *Welwitschia*. In contrast, the existence of the *atp8*-*cox3* cluster and the loss of *rpl2-rps19* and *trnP(TGG)-sdh3* seem to support the close relationship between *Taxus* and *Welwitschia*. However, *Taxus* and *Welwitschia* could have lost *rpl2-rps19* separately because *Taxus* lost only *rpl2* but *Welwitschia* lost a long cluster including *rpl2*, *rps19*, *rps3* and *rpl16* (Additional file 11: Figure S6) [41]. In addition, *Pinus* lost a long cluster including *cox3*, *sdh4* and *atp8*, whereas *Welwitschia* lost only the cluster *cox3-sdh4*. Therefore, it is difficult to find evidence to resolve the phylogenetic relationships of Pinaceae, Gnetales and Conifer II based on mitochondrial gene clusters.

Approximately 500 and 100 variants of a 36-bp *Bpu* element were identified in *Cycas* and *Ginkgo*, respectively. This element is putatively mobile because it contains a 4-bp direct terminal repeat [3, 4]. However, only one and two reduced similar sequences were found in *Welwitschia* and *Pinus,* respectively, and no similar sequences were found in *Taxus*, supporting expansion of the *Bpu* element in only *Cycas* and *Ginkgo* [3, 4]. In addition, we did not find other transposable elements in the *Pinus* and *Taxus* mitogenomes. In fact, no expansion of other repeat families has been reported in plants, implying that the expansion of repeat families is rare in land plant mitogenomes.

### The number of RNA editing sites is not correlated with the GC content of mitochondrial genes

We obtained a mitochondrial RNA editing site map of *Taxus cuspidata* by comparing the transcriptomic and genomic high-throughput sequencing data. The results showed that all editing sites are C-to-U conversions, which is similar to the findings for other seed plants [76]. RNA editing occurred not only in protein-coding genes (exons and introns) but also in rRNA, tRNA and intergenic regions. A total of 974 editing sites were identified when the editing efficiency was set to greater than 0.05. A total of 730 editing sites were found in exons, of which 582 were nonsilent, affecting 1.63% of coding sequences (Additional file 7: Table S3 and Additional file 8: Table S4). This supports the essential role of nonsilent editing sites in the proper functioning of mitochondrially encoded proteins [27, 77, 78].

A total of 1206 and 1306 editing sites were predicted in the 41 protein-coding genes shared by *Cycas* and *Ginkgo*, respectively, but only 225 predicted editing sites were found in *Welwitschia* (Additional file 15: Table S7). We predicted RNA editing sites by using the same online tool (PREP-Mt) with the same cutoff of 0.2 and found 1179 and 1102 editing sites in *Pinus taeda* and *Taxus cuspidata*, respectively (Table 1). The number of predicted editing sites was greater than the number from empirical data in *Taxus*, but the prediction and empirical data in *Cycas* and *Ginkgo* were similar in terms of the number of editing sites [4, 79, 80] (Additional file 16: Table S8). Therefore, multiple mitochondrial RNA editing sites have been lost in some lineages of gymnosperms, similar to the pattern observed in angiosperms [46].

Due to the very large difference between the numbers of predicted and empirically measured RNA editing sites in *Taxus* (this study) and *Welwitschia* [81], we did not compare the variation in RNA editing sites among the five focal species in detail. Generally, the number of mitochondrial RNA editing sites is not correlated with mitogenome size or GC content but is significantly correlated with the GC content of genes [53, 82]. However, this correlation is not supported in this study. The *Taxus* and *Welwitschia* mitogenomes have fewer editing sites than those of *Cycas*, *Ginkgo*, and *Pinus*, but the GC contents of the *Taxus* and *Welwitschia* mitochondrial genes are the highest and lowest among these five species, respectively.

### Size variation in gymnosperm mitogenomes is still a mystery

In land plants, mitogenome size varies greatly, from 66 kb in *Viscum scurruloideum* [83] to as large as 11 Mb in *Silene conica* [84]. Because there is no significant difference in the number of mitochondrial genes, the variation of noncoding DNA content are statistically associated with variation in mitogenome size. Variation in noncoding DNA content could be affected by different factors, such as the proliferation of retrotransposons, the generation of repetitive DNA by homologous recombination, and the incorporation of foreign sequences via intracellular transfer from the plastid or nuclear genome or horizontal transfer of mitochondrial DNA (e.g., [3, 4, 55, 85–87]). However, in different species, the increase in mitogenome size could be caused by different factors. For example, although foreign sequences were suggested to contribute to mitogenome size variation, the origins of foreign sequences differ among species. The mitogenome of *Amborella trichopoda* contains six genome equivalents of foreign mitochondrial DNA from algae, mosses, and other angiosperms, whereas DNA sequence transfer from the nucleus is a core mechanism for mtDNA size expansion in apple, maize and grape [86]. In some cases, mitogenome size variation is affected by multiple factors. For example, the mitogenome expansion of *Cucurbita pepo* was largely the result of the accumulation of unprecedented amounts of both chloroplast sequences (~ 113 kb) and short repeat sequences (~ 370 kb) [88]. In addition, changes in recombination, including gene conversion, may contribute to the variation in mitogenome size [84]. Furthermore, many mitogenomes contain multiple repeats, but there is no strict relationship between repeat content and genome size in angiosperm mtDNA [83, 87], although repeat was considered to be a main factor for some mitogenome expansion. For example, an accumulation of repeats in intergenic regions contributed to 371 kb or 38% of the *Cucurbita* mitogenome [88].

Both the *Welwitschia mirabilis* and *Pinus taeda* mitogenomes are larger than those of *Cycas* and *Ginkgo* (Table 1). The mitogenome size of *Welwitschia mirabilis* and *Pinus taeda* is 978,846 bp and 1,191,054 bp, respectively. That is, the size difference between their genomes is only approximately 200 kb. However, there is some disparity between their noncoding regions. In *Pinus*, 170 kb of repeats and 5.6 kb of chloroplast-derived sequences were identified, whereas 50 kb of repeats and 7.9 kb of chloroplast-derived sequences were found in *Welwitschia*. In addition, numerous tandem repeats (71 kb) were identified in *Pinus,* but only a few (24 kb) were found in *Welwitschia*. Considering that the difference in the mitogenome size of gymnosperms is larger than 500 bp, the number of repeats and the increase in the abundance of plastid-derived sequences were not the main reasons for the mitogenome expansion of *Pinus* and *Welwitschia*. Guo et al. [4] suggested that the substantial amount of unidentified DNA could contribute to the expansion of the *Welwitschia* mitogenome, and they deduced that these unidentified DNAs could be derived from the nuclear genome by intracellular transfer. As nuclear-derived repetitive sequences originated unambiguously and generally did not proliferate after transfer [89], we identified them in the mtDNA of *Pinus* and *Welwitschia*, and the results showed that only approximately 5.3 kb and 2.5 kb were found in these species (Table 1), which did not show significant differences from the other gymnosperms. Therefore, the origin of most unidentified noncoding regions in these two species is still unknown. Small repeats contributed to the recombination in mitogenomes [90]. However, although a large number of small repeats (150 kb) were found in the *Pinus* mitogenome, only 48 kb of small repeats were identified in the *Welwitschia* mitogenome. The newly sequenced mitogenome of *Taxus cuspidata* is slightly larger than that of *Cycas* and *Ginkgo* (Table 1). However, the mitogenome of *Taxus* has fewer protein-coding genes, tRNAs, introns and RNA editing sites, and higher mutation rates than that of *Cycas* and *Ginkgo* (Additional file 14: Figure S8 and Table 1). We speculate that the mechanisms of mitogenome expansion could differ in gymnosperms.

Some nonadaptive mechanisms have been developed to explain the origins of variation in mitogenome size and complexity, such as the mutational hazard hypothesis (MHH) [91], different DNA repair mechanisms in transcribed and nontranscribed regions [92], and break-induced replication [93]. However, the MHH was rejected because some *Silene* species have extremely large mitogenomes but also have high rates of mutation [55]. In this study, *Welwitschia* also has a large mitogenome but high rates of mutation (Additional file 14: Figure S8). In addition, the possibility of different mechanisms of repair for coding and noncoding DNA was also not supported because transcription-coupled repair (TCR) is not found in plants [92, 93]. The number of repeats is significantly different between *Pinus* and *Welwitschia*, both of which have a large mitogenome, implying that the frequency of recombination caused by repeats would also be different between their mitogenomes. Furthermore, the model of Christensen [92, 93] cannot explain the occurrence of RNA editing, HGT and intron accumulation in land plant mitogenomes [94]. Smith (2016) suggested that there could be a threshold mutation rate in mitogenomes, but it is difficult to determine this value. More research will help uncover the underlying mechanism of the size variation in plant mitogenomes.

## Conclusions

In this project, we sequenced the complete mitogenome of *Taxus cuspidata* in Conifer II. By comparing the mitogenomes from the five gymnosperm lineages, we show that some protein-coding genes have been transferred to the nuclear genomes in *Taxus* and *Welwitschia*, individually. We also show that similar to the pattern observed in angiosperms, multiple tRNA genes and introns have been lost in some lineages of gymnosperms, but gene clusters in gymnosperms could be less conserved than those of angiosperms. In addition, we show that number of introns and genes could be positively correlated with number of RNA editing sites.

## Supplementary information

**Additional file 1: Table S1.** Primers used in this study.

**Additional file 2: Table S2.** The detailed information of the *Taxus cuspidata* mitochondrial genes, exons, and introns.

**Additional file 3: Figure S1.** Average sequencing coverage (A) and qPCR cycle number (B) for mitochondrial and putative transferred genes in *Taxus*.

**Additional file 4: Figure S2.** Structure of transferred genes in *Taxus* and their counterparts in *Cycas*, *Ginkgo* and *Pinus*. Lines and boxes represent introns and exons, respectively. Open boxes indicate partial exons, and dotted lines indicate that some sequences of introns are absent. Grey shadows represent well aligned exons.

**Additional file 5: Figure S3.** GC content variation in the protein-coding genes of the sampled species. (A) All codon positions; (B) the first codon position; (C) the second codon position; (D) the third codon position.

**Additional file 6: Figure S4.** Length variation in *cis*-spliced introns of the selected plant mitogenomes.

**Additional file 7: Table S3.** Summary of mitochondrial RNA editing events in *Taxus*.

**Additional file 8: Table S4.** Detailed information of RNA editing sites in the *Taxus cuspidata* mitogenome.

**Additional file 9: Figure S5.** Examples of genes with striking divergence between observed and predicted (PREP with cutoff value = 0.2, PREPACT with filter threshold = 20%) RNA editing sites. The horizontal line represents gene length, and the vertical line indicates the position of RNA editing site.

**Additional file 10: Table S5.** Comparison of RNA editing sites among observed, predicted by PREP and PREPACT, respectively, in the *Taxus* mitogenome.

**Additional file 11: Figure S6.** Mitochondrial gene clusters across gymnosperms.

**Additional file 12: Figure S7.** (A) Total length of repeats in the mitogenomes of gymnosperms. The values in the bar indicate the number of repeat pairs. (B) Copy number of repeat pairs in each species.

**Additional file 13 Table S6**. Nuclear-derived repetitive sequences in the sampled gymnosperm mitogenomes.

**Additional file 14: Figure S8.** Synonymous (A) and nonsynonymous (B) sequence divergence in the conserved region of mitochondrial protein-coding genes in gymnosperms. Dots indicate mitochondrial protein-coding genes that have not transferred to the nucleus in all five gymnosperm species. Circles indicate the transferred genes in *Taxus* and *Welwitschia* and their homologous genes in *Cycas*, *Ginkgo* and Pinaceae.

**Additional file 15: Table S7.** Number of RNA editing sites predicted from PREP-Mt.

**Additional file 16: Table S8.** Comparison of the predicted (by PREP) and observed RNA editing sites in the sampled gymnosperm mitogenomes.

## Data Availability

The raw data were deposited in the Short Read Archive database under accession numbers SRR10305024-SRR10305026 (BioProject: PRJNA578185) and the assembled and annotated *Taxus* mitogenome has been deposited in GenBank under accession number MN593023. Homologous transcripts of eight lost mitochondrial genes of *Taxus cuspidata* have been submitted to GenBank, with accession numbers MN886610-MN886617.

## Abbreviations

EGT: Endosymbiotic gene transfer
GC1/2/3: GC content of the first, second and third codon positions, respectively
HGT: Horizontal gene transfer
MHH: Mutational hazard hypothesis
mitogenome: Mitochondrial genome
MTPTs: Plastid-derived mtDNA
NCBI: National Center for Biotechnology Information
NGS: Next-generation sequencing
ORF: Open reading frame
TCR: Transcription-coupled repair
