# Supplementary material for: The complete mitochondrial genome of Taxus cuspidata (Taxaceae): eight protein-coding genes have transferred to the nuclear genome
Source: BMC Evol Biol. 2020 Jan 20;20:10. doi: 10.1186/s12862-020-1582-1 (PMC6971862; doi:10.1186/s12862-020-1582-1)
Supplement: Supplementary file 1 — Additional file 1: Table S1. Primers used in this study. [file 12862_2020_1582_MOESM1_ESM.docx]

**Additional file 1: Table S1.** Primers used in this study.

| **Gene** | **Primer** | **Sequence(5'to3')** |
| --- | --- | --- |
| *LFY* | LFYE1F5_tax | TATGACAGAACAAGAGTTGGATGAT |
|  | LFYE1R1 | GCATTCTCATCAATCTTGCGT |
| *nad1* | nad1_Tax_qE3F1 | ATGGTTTGGTATTCCCTTGTTC |
|  | nad1_Tax_qE3R1 | TGCATTATAGCCTGCGACTGA |
| *nad2* | nad2_Tax_qE5F1 | CTTAGCGAAAATAATGTATCCCG |
|  | nad2_Tax_qE5R1 | TGATGAGTAACTAAGAACGAGGGAG |
| *nad3* | nad3_Tax_qF1 | TCCCTTCCTCTTCACTTCCAAT |
|  | nad3_Tax_qR1 | GAAACGACTTCTGGCATCACC |
| *nad4* | nad4_Tax_qE2F1 | TATATACTCCCCCGACCACTCTG |
|  | nad4_Tax_qE2R1 | GTCACAGGATTCGTATGGGCTA |
| *nad4L* | nad4L_Tax_qF1 | CCCAAATAGGCGGAATATTACTATT |
|  | nad4L_Tax_qR1 | CGATAAAGCAAATGATTGACCC |
| *nad5* | Tax_nad5_E2F1p | ATTACAGAACGATCCGAAGAGG |
|  | Tax_nad5_E2R1p | GGCATAGGTAAAGGGTAACGAG |
| *nad6* | nad6_Tax_qF1 | ATTCCGTTCGGTTCTTGGTT |
|  | nad6_Tax_qR1 | GTCCTCCTCATCATAGTCCTCTTG |
| *nad7* | nad7_Tax_qE4F1 | GGGAGTATGCTGGGATTTGA |
|  | nad7_Tax_qE4R1 | CTTACGATCATCGGCTTTCA |
| *nad9* | nad9_Tax_qF1 | TACCGATACAGACTACCCATTTCA |
|  | nad9_Tax_qR1 | ATCCCATACTTCTCGCTCCC |
| *sdh3* | sdh3p_Tax_F2 | CATAGTAAACCCAAATAGGAAC |
|  | sdh3p_Tax_R2 | AGATTGGTAGAGACTGGTATAAG |
| *sdh4* | sdh4_Tax_qF1 | GGTCCATGAAGGAAAGAACAA |
|  | sdh4_Tax_qR1 | GAGGAGTAGGGAAGCAGCAA |
| *cob* | cob_Tax_qF1 | GGATTTCTTGTGCTCCTAATGTCT |
|  | cob_Tax_qR1 | AAACGAGTGCTATTGCGGCTAC |
| *cox1* | cox1_Tax_qF1 | CTCACGCTTCTCCCACGACCTT |
|  | cox1_Tax_qR1 | CCCAGTGCCGCTACCTACTTCTAC |
| *cox2* | cox2_Tax_qF1 | TCATAGTCGGGCTGTCCCTTCC |
|  | cox2_Tax_qR1 | GCACTGACCGTAATAAACTCCTTCT |
| *cox3* | cox3_Tax_qF1 | ATGTTCCTCTTCGCTTTCTTCC |
|  | cox3_Tax_qR1 | TAAACTGCTTGTCGCTCCTTCC |
| *atp1* | atp1_Tax_qF1 | AGGATTACCGACTACAGCACCG |
|  | atp1_Tax_qR1 | GATTCAACGCTATTCCTTTCACA |
| *atp4* | atp4_Tax_qF1 | CGCCTAAGTGCGAAAGGACAGT |
|  | atp4_Tax_qR1 | AAGACGGATGCGACGGGAATAA |
| *atp6* | atp6_Tax_qF1 | AGTCCTGCTCTTGGTTCATTCC |
|  | atp6_Tax_qR1 | ATTTCCAGAACGACCGCCTATT |
| *atp8* | atp8_Tax_qF1 | AATCAGTGGCTCACGGAGAATG |
|  | atp8_Tax_qR1 | CATTATGTTCTCCCTACAAGGTTTC |
| *atp9* | apt9_Tax_qF1 | TGGAGGGTGCGAAATCAATAGG |
|  | apt9_Tax_qR1 | TGGCGTGACCGAATGATTGTTT |
| *ccmB* | ccmB_Tax_qF1 | CGAGCCTTTCTTTCGGAGTGAT |
|  | ccmB_Tax_qR1 | CCCTAGTGGAATGTTGAACCAATTC |
| *ccmC* | ccmC_Tax_qF1 | GCAGTGGCTATCAGCAGTTCTC |
|  | ccmC_Tax_qR1 | GGGTAAATGAAGGACGAGATGAA |
| *ccmFC* | ccmFC_Tax_qF1 | TTTACTATTCTCCCGCACCCTT |
|  | ccmFC_Tax_qR1 | GAGTAAACTGTGTCTTTCTCGTGCT |
| *ccmFN* | ccmFN_Tax_qF1 | GCTTCCATTCACATTCCTGCTA |
|  | ccmFN_Tax_qR1 | TGCTCTTTCTATGGACCGATGT |
| *rpl2* | rpl2_Tax_qF2 | TATTGCTTTGGTGCGATGGA |
|  | rpl2_Tax_qR2 | TTCTGTAGTTCGTGGAGCGTAT |
| *rpl5* | rpl5_Tax_qF1 | CACCGAATCACGCCAACATC |
|  | rpl5_Tax_qR1 | TCGTGCGACTCCCTCTTTCC |
| *rpl16* | rpl16_Tax_qF1 | GGGTTGCGAAACAGGCAGTA |
|  | rpl16_Tax_qR1 | GCTTCAATGGCTCGATATGAGA |
| *rps1* | rps1p_Tax_F1 | GGTAGGGTTTCTCGACGTGGCA |
|  | rps1p_Tax_R1 | ATCCAAGCCCTCTGCTGCCTAA |
| *rps2* | rps2p_Tax_F2 | CACTTTCTCGGTTCCCTCTTTC |
|  | rps2p_Tax_R2 | TCACCACCACACAGTCGGGC |
| *rps3* | rps3_Tax_qF1 | CCGATCACCATCGAGCAGTT |
|  | rps3_Tax_qR1 | GGGAGGAGGAAGTTGAAGCA |
| *rps4* | rps4_Tax_qF1 | AGACTGACGAGGATACAACGC |
|  | rps4_Tax_qR1 | ATGGAGACGAACCAGGATGA |
| *rps7* | rps7_Tax_qF1 | TTCAAGCTGTAGGGAATATCAAAC |
|  | rps7_Tax_qR1 | CTGCTCCAAGTATCCAACGA |
| *rps10* | rps10p_Tax_F2 | ATCGGAGACGCCAAGATTCGC |
|  | rps10p_Tax_R2 | GGCCAATTCATTCACATCTGTCTC |
| *rps11* | rps11p_Tax_F2 | TCTCGCCGTAGGACCAAGTATG |
|  | rps11p_Tax_R2 | ATCCATTATGTGGAAGTTGGGTT |
| *rps12* | rps12_F2 | ATAGCCAAAGTACGGTTGAGC |
|  | rps12_R2 | TCGCACCATATTTCGATCTGC |
| *rps13* | rps13_Tax_qF1 | CGACCGACTTGAACGAATAA |
|  | rps13_Tax_qR1 | CGTAAGGGCAATCCATCTTG |
| *rps14* | rps14p_Tax_F1 | TATGAATTGAAACGGGTGCTT |
|  | rps14p_Tax_R1 | GATGCTAATTCACGGAAACAAA |
| *rps19* | rps19_Tax_qF1 | GAATTGGTTGATCGCTCCGTAC |
|  | rps19_Tax_qR1 | TTTCCGTGCAAAGGCAAACT |
| *matR* | matR_Tax_qF1 | GATCGAGGTATCATTAGCCGGAGAA |
|  | matR_Tax_qR1 | GCCGAGGATTTGTGCTTGTGG |
| *mttB* | mttB_Tax_qF1 | CCACCAATACGCTCCCAAGTCC |
|  | mttB_Tax_qR1 | TCGGATAAATAGGCGAACAAGC |
| *rpl10* | rpl10_F1 | ATTCCAGTGGCTTGACCAGT |
|  | rpl10_R1 | AAGTGGGACCTGCACTATGA |
|  | rpl10_F2 | AGTGGCTCGACCAGTAACCAAT |
|  | rpl10_R2 | GAACTGGGTAAATGGAAATGG |
