# Supplementary material for: The complete mitochondrial genome of Taxus cuspidata (Taxaceae): eight protein-coding genes have transferred to the nuclear genome
Source: BMC Evol Biol. 2020 Jan 20;20:10. doi: 10.1186/s12862-020-1582-1 (PMC6971862; doi:10.1186/s12862-020-1582-1)
Supplement: Supplementary file 2 — Additional file 2: Table S2. The detailed information of the Taxus cuspidata mitochondrial genes, exons, and introns. [file 12862_2020_1582_MOESM2_ESM.docx]

**Additional file 2: Table S2.** The detailed information of the *Taxus cuspidata* mitochondrial genes, exons, and introns.

| **Gene** | | **Size (bp)** | **Coordinates** | | **Strand** | **No. of Amino acids** | **Cis-/trans- spliced** | **Note** |
| --- | --- | --- | --- | --- | --- | --- | --- | --- |
|  |  |  | **from** | **to** |  |  |  |  |
| I. Complex I genes | | | | | | | | |
|  | *nad*1 | 984 |  |  |  | 327 |  | NADH dehydrogenase subunit 1 |
|  | exon 1 | 385 | 266951 | 267335 | + |  |  |  |
|  | intron1 |  | 267336 | 267835 | + |  | ө | 5' trans-spliced intron |
|  | intron1 |  | 143959 | 144458 | - |  |  | 3' trans-spliced intron |
|  | exon 2 | 275 | 143684 | 143958 | - |  |  |  |
|  | intron2 |  | 143184 | 143683 | - |  | ө | 5' trans-spliced intron |
|  | intron2 |  | 271353 | 271852 | + |  |  | 3' trans-spliced intron |
|  | exon 3 | 59 | 271853 | 271911 | + |  |  |  |
|  | intron3 |  | 271912 | 272411 | + |  | ө | 5' trans-spliced intron |
|  | intron3 |  | 343906 | 344405 | - |  |  | 3' trans-spliced intron |
|  | exon 4 | 265 | 343641 | 343905 | - |  |  |  |
|  | *nad*2 | 1473 |  |  |  | 490 |  | NADH dehydrogenase subunit 2 |
|  | exon 1 | 545 | 373233 | 373777 | - |  |  |  |
|  | intron1 |  | 372733 | 373232 | - |  | ө | 5' trans-spliced intron |
|  | intron1 |  | 8027 | 8526 | + |  |  | 3' trans-spliced intron |
|  | exon 2 | 167 | 8527 | 8693 | + |  |  |  |
|  | intron2 |  | 8694 | 11057 | + |  | ● |  |
|  | exon 3 | 573 | 11058 | 11630 | + |  |  |  |
|  | intron3 |  | 11631 | 12130 | + |  | ө | 5' trans-spliced intron |
|  | intron3 |  | 396897 | 397396 | + |  |  | 3' trans-spliced intron |
|  | exon 4 | 188 | 397397 | 397584 | + |  |  |  |
|  | *nad*3 | 357 | 33528 | 33884 | + | 118 |  | NADH dehydrogenase subunit 3 |
|  | *nad*4 | 1488 |  |  |  | 495 |  | NADH dehydrogenase subunit 4 |
|  | exon 1 | 461 | 317302 | 317762 | - |  |  |  |
|  | intron1 |  | 316802 | 317301 | - |  | ө | 5' trans-spliced intron |
|  | intron1 |  | 390796 | 391295 | + |  |  | 3' trans-spliced intron |
|  | exon 2 | 515 | 391296 | 391810 | + |  |  |  |
|  | intron2 |  | 391811 | 392310 | + |  | ө | 5' trans-spliced intron |
|  | intron2 |  | 297315 | 297814 | - |  |  | 3' trans-spliced intron |
|  | exon 3 | 423 | 296892 | 297314 | - |  |  |  |
|  | intron3 |  | 296392 | 296891 | - |  | ө | 5' trans-spliced intron |
|  | intron3 |  | 363273 | 363772 | + |  |  | 3' trans-spliced intron |
|  | exon 4 | 89 | 363773 | 363861 | + |  |  |  |
|  | *nad*4L | 303 | 316203 | 316505 | - | 100 |  | NADH dehydrogenase subunit 4L |
|  | *nad*5 | 2007 |  |  |  | 668 |  | NADH dehydrogenase subunit 5 |
|  | exon 1 | 231 | 216729 | 216959 | - |  |  |  |
|  | intron1 |  | 215436 | 216728 | - |  | ● |  |
|  | exon 2 | 1224 | 214212 | 215435 | - |  |  |  |
|  | intron2 |  | 213712 | 214211 | - |  | ө | 5' trans-spliced intron |
|  | intron2 |  | 231222 | 231721 | + |  |  | 3' trans-spliced intron |
|  | exon 3 | 22 | 231724 | 231745 | + |  |  |  |
|  | intron3 |  | 231746 | 232245 | + |  | ө | 5' trans-spliced intron |
|  | intron3 |  | 434067 | 434566 | - |  |  | 3' trans-spliced intron |
|  | exon 4 | 395 | 433672 | 434066 | - |  |  |  |
|  | intron4 |  | 432868 | 433671 | - |  | ● |  |
|  | exon 5 | 135 | 432733 | 432867 | - |  |  |  |
|  | *nad*6 | 669 | 75856 | 76524 | + | 222 |  | NADH dehydrogenase subunit 6 |
|  | *nad*7 | 1185 |  |  |  | 394 |  | NADH dehydrogenase subunit 7 |
|  | exon 1 | 212 | 4172 | 4383 | + |  |  |  |
|  | intron1 |  | 4384 | 6844 | + |  | ● |  |
|  | exon 2 | 973 | 6845 | 7817 | + |  |  |  |
|  | *nad*9 | 585 | 207027 | 207611 | + | 194 |  | NADH dehydrogenase subunit 9 |
| II. Complex II genes | | | | | | | | |
|  | *sdh*4 | 420 | 188336 | 188755 | + | 139 |  | succinate dehydrogenase cytochrome subunit 4 |
| III. Complex III & IV genes | | | | | | | | |
|  | *cob* | 1185 | 182798 | 183982 | + | 394 |  | apocytochrome b |
|  | *cox*1 | 1572 | 225703 | 227274 | - | 523 |  | cytochrome c oxidase subunit 1 |
|  | *cox*2 | 882 |  |  |  | 293 |  | cytochrome c oxidase subunit 2 |
|  | exon 1 | 394 | 190068 | 190461 | + |  |  |  |
|  | intron1 |  | 190462 | 190961 | + |  | ө | 5' trans-spliced intron |
|  | intron1 |  | 167463 | 167962 | + |  |  | 3' trans-spliced intron |
|  | exon 2 | 488 | 167963 | 168450 | + |  |  |  |
|  | *cox*3 | 792 | 187617 | 188408 | + | 263 |  | cytochrome c oxidase subunit 3 |
| IV. Complex V genes | | | | | | | | |
|  | *atp*1 | 1536 | 49745 | 51280 | + | 511 |  | ATPase subunit 1 |
|  | *atp*4 | 705 | 233957 | 234661 | + | 234 |  | ATPase subunit 4 |
|  | *atp*6 | 765 | 237555 | 238319 | + | 254 |  | ATPase subunit 6 |
|  | *atp*8 | 468 | 184943 | 185410 | + | 155 |  | ATPase subunit 8 |
|  | *atp*9 | 225 | 425277 | 425501 | + | 74 |  | ATPase subunit 9 |
| V. Cytochrome c biogenesis genes | | | | | | | | |
|  | *ccm*B | 642 | 148160 | 148801 | - | 213 |  | cytochrome c biogenesis B |
|  | *ccm*C | 750 | 211128 | 211877 | - | 249 |  | cytochrome c biogenesis C |
|  | *ccm*FC | 1224 | 247830 | 249053 | + | 407 |  | cytochrome c biogenesis FC |
|  | *ccm*FN | 1824 | 244901 | 246724 | + | 607 |  | cytochrome c biogenesis FN |
| VI. Ribosomal protein genes | | | | | | | | |
|  | *rpl*5 | 561 | 146100 | 146660 | - | 186 |  | ribosomal protein L5 |
|  | *rpl*16 | 618 | 74830 | 75447 | + | 205 |  | ribosomal protein L16 |
|  | *rps*3 | 1677 | 73348 | 75024 | + | 558 |  | ribosomal protein S3 |
|  | *rps*4 | 1056 | 34516 | 35571 | + | 351 |  | ribosomal protein S4 |
|  | *rps*12 | 390 | 33943 | 34332 | + | 129 |  | ribosomal protein S12 |
|  | *rps*13 | 357 | 67574 | 67930 | + | 118 |  | ribosomal protein S13 |
|  | *rps*19 | 300 | 73045 | 73344 | + | 99 |  | ribosomal protein S19 |
| VII. Other protein-coding genes | | | | | | | | |
|  | *mat*R | 3417 | 98146 | 101562 | - | 1138 |  | maturase-related protein |
|  | *mtt*B | 750 | 66127 | 66876 | + | 249 |  | transport membrane protein |
| VIII. rRNA genes | | | | | | | | |
|  | *rrn*5 | 111 | 158429 | 158539 | + |  |  | 5S ribosomal RNA |
|  | *rrn*18_1 | 2538 | 154703 | 157240 | + |  |  | 18S ribosomal RNA |
|  | *rrn*18_2 | 2538 | 466951 | 562 | + |  |  |  |
|  | *rrn*26 | 4113 | 406246 | 410358 | + |  |  | 26S ribosomal RNA |
| IX. tRNA genes | |  |  |  |  |  |  |  |
|  | *trn*D-GUC | D | 25888 | 25961 | + |  |  | GAC |
|  | *trn*E-UUC | E | 96646 | 96717 | + |  |  | GAA |
|  | *trn*M-CAU | M | 243635 | 243708 | + |  |  | ATG |
|  | *trn*D-GUC | D | 269464 | 269537 | + |  |  | GAC |
|  | *trn*I-CAU | I | 274460 | 274533 | + |  |  | ATG |
|  | *trn*Y-GUA | Y | 426362 | 426444 | + |  |  | TAC |
|  | *trn*E-UUC | E | 449504 | 449575 | - |  |  | GAA |
|  | *trn*M-CAU | M | 443321 | 443394 | - |  |  | ATG |
|  | *trn*R-UCG | R | 279911 | 279982 | - |  |  | CGA |
|  | *trn*W-CCA-cp | W | 121247 | 121320 | - |  |  | TGG |

(“●” and “ө” indicate cis- and trans-spliced introns, respectively.)
