# Supplementary material for: The complete mitochondrial genome of Taxus cuspidata (Taxaceae): eight protein-coding genes have transferred to the nuclear genome
Source: BMC Evol Biol. 2020 Jan 20;20:10. doi: 10.1186/s12862-020-1582-1 (PMC6971862; doi:10.1186/s12862-020-1582-1)
Supplement: Supplementary file 3 — Additional file 3: Figure S1. Average sequencing coverage (A) and qPCR cycle number (B) for mitochondrial and putative transferred genes in Taxus. [file 12862_2020_1582_MOESM3_ESM.pdf]

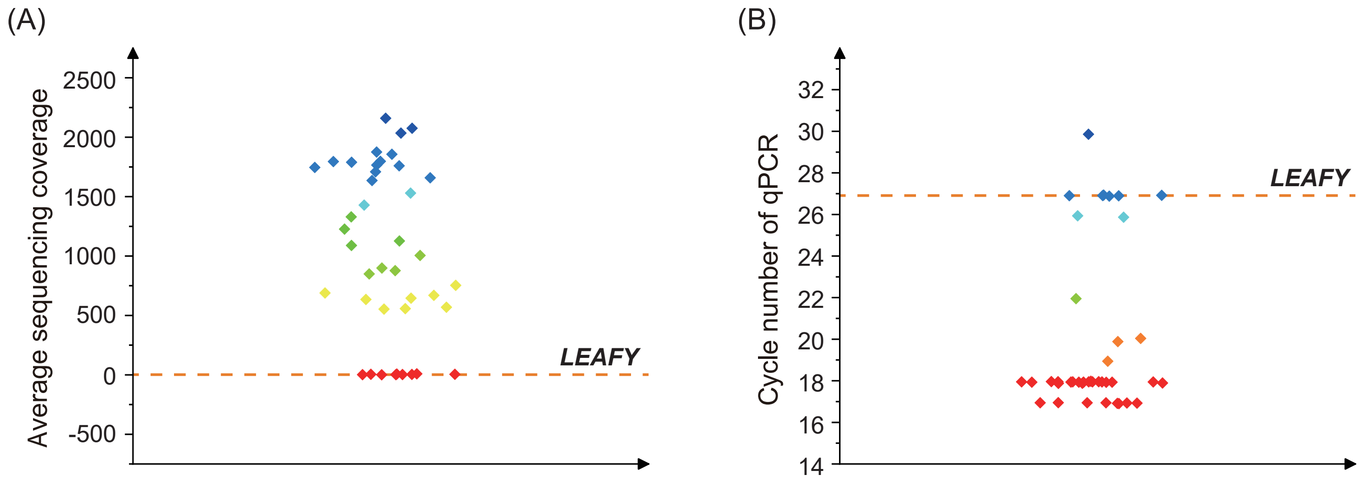


**Additional file 3: Figure S1.** Average sequencing coverage (A) and qPCR cycle number (B) for mitochondrial and putative transferred genes in *Taxus*.
