# Supplementary material for: The complete mitochondrial genome of Taxus cuspidata (Taxaceae): eight protein-coding genes have transferred to the nuclear genome
Source: BMC Evol Biol. 2020 Jan 20;20:10. doi: 10.1186/s12862-020-1582-1 (PMC6971862; doi:10.1186/s12862-020-1582-1)
Supplement: Supplementary file 4 — Additional file 4: Figure S2. Structure of transferred genes in Taxus and their counterparts in Cycas, Ginkgo and Pinus. Lines and boxes represent introns and exons, respectively. Open boxes indicate partial exons, and dotted lines indicate that some sequences of introns are absent. Grey shadows represent well aligned exons. [file 12862_2020_1582_MOESM4_ESM.pdf]

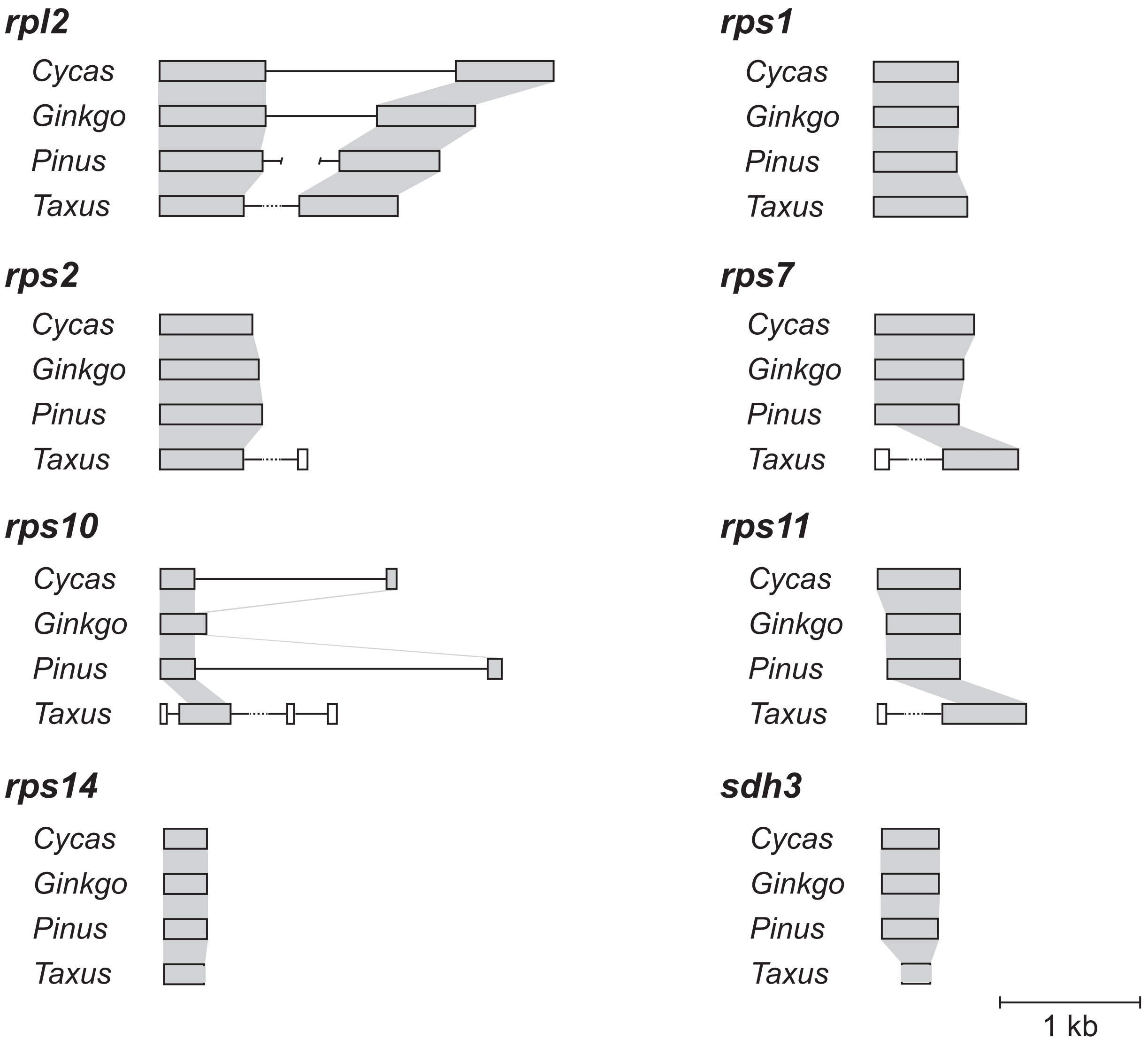

Additional file 4: Figure S2. Structure of transferred genes in *Taxus* and their counterparts in *Cycas*, *Ginkgo* and *Pinus*. Lines and boxes represent introns and exons, respectively. Open boxes indicate partial exons, and dotted lines indicate that some sequences of introns are
