# Supplementary material for: The complete mitochondrial genome of Taxus cuspidata (Taxaceae): eight protein-coding genes have transferred to the nuclear genome
Source: BMC Evol Biol. 2020 Jan 20;20:10. doi: 10.1186/s12862-020-1582-1 (PMC6971862; doi:10.1186/s12862-020-1582-1)
Supplement: Supplementary file 5 — Additional file 5: Figure S3. GC content variation in the protein-coding genes of the sampled species. (A) All codon positions; (B) the first codon position; (C) the second codon position; (D) the third codon position. [file 12862_2020_1582_MOESM5_ESM.pdf]

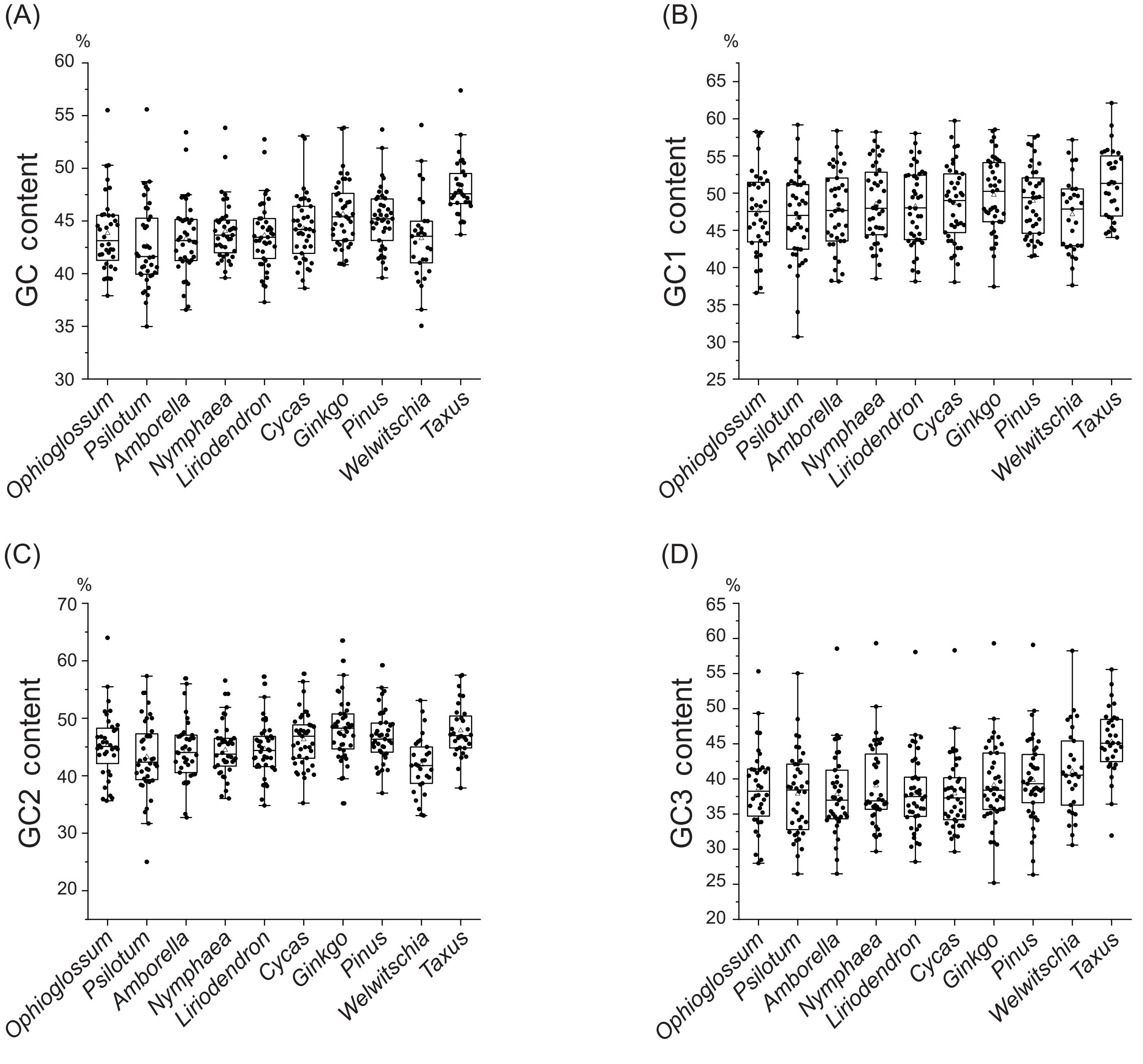

Additional file 5: Figure S3. GC content variation in the protein-coding genes of the sampled species. (A) All codon positions; (B) the first codon position; (C) the second codon position; (D) the third codon position.
