# Supplementary material for: The complete mitochondrial genome of Taxus cuspidata (Taxaceae): eight protein-coding genes have transferred to the nuclear genome
Source: BMC Evol Biol. 2020 Jan 20;20:10. doi: 10.1186/s12862-020-1582-1 (PMC6971862; doi:10.1186/s12862-020-1582-1)
Supplement: Supplementary file 6 — Additional file 6: Figure S4. Length variation in cis-spliced introns of the selected plant mitogenomes. [file 12862_2020_1582_MOESM6_ESM.pdf]

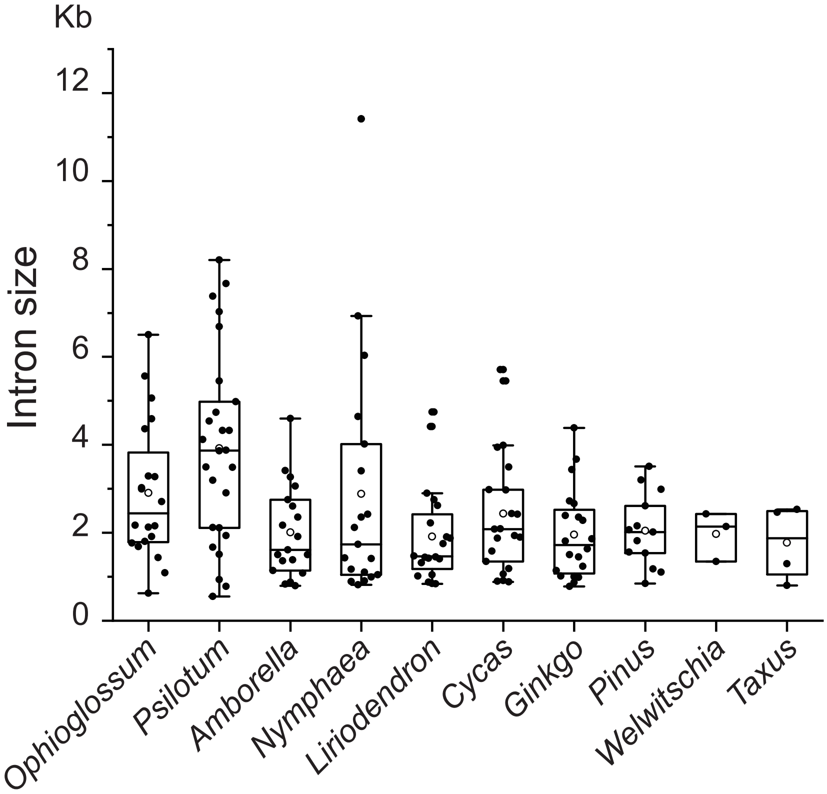

Additional file 6: Figure S4. Length variation in *cis*-spliced introns of the selected plant mitogenomes.
