# Supplementary material for: The complete mitochondrial genome of Taxus cuspidata (Taxaceae): eight protein-coding genes have transferred to the nuclear genome
Source: BMC Evol Biol. 2020 Jan 20;20:10. doi: 10.1186/s12862-020-1582-1 (PMC6971862; doi:10.1186/s12862-020-1582-1)
Supplement: Supplementary file 7 — Additional file 7: Table S3. Summary of mitochondrial RNA editing events in Taxus. [file 12862_2020_1582_MOESM7_ESM.docx]

**Additional file 7: Table S3.** Summary of mitochondrial RNA editing events in *Taxus*.

|  | **Total** | **5-20%** | **20-40%** | **40-60%** | **60-80%** | **80-100%** |
| --- | --- | --- | --- | --- | --- | --- |
| **C-to-U** | 974 | 228 | 159 | 129 | 159 | 299 |
| **U-to-C** | 0 |  | 0 | 0 | 0 | 0 |
| **Coding** |  |  |  |  |  |  |
| **1st** | **207** | **35** | **27** | **26** | **28** | **91** |
| nonsilent | 201 | 35 | 26 | 25 | 26 | 89 |
| silent | 6 | 0 | 1 | 1 | 2 | 2 |
| **2nd** | **372** | **35** | **45** | **49** | **86** | **157** |
| nonsilent | 372 | 35 | 45 | 49 | 86 | 157 |
| silent | 0 | 0 | 0 | 0 | 0 | 0 |
| **3rd** | **151** | **59** | **40** | **22** | **12** | **18** |
| nonsilent | 9 | 3 | 3 | 1 | 1 | 1 |
| silent | 142 | 56 | 37 | 21 | 11 | 17 |
| **Nocoding** |  |  |  |  |  |  |
| **intron** | 61 |  | 10 | 7 | 9 | 7 |
| **rRNA** | 2 |  | 0 | 0 | 1 | 1 |
| **tRNA** | 0 |  | 0 | 0 | 0 | 0 |
| **intergenic** | 180 |  | 37 | 25 | 23 | 25 |
