# Supplementary material for: The complete mitochondrial genome of Taxus cuspidata (Taxaceae): eight protein-coding genes have transferred to the nuclear genome
Source: BMC Evol Biol. 2020 Jan 20;20:10. doi: 10.1186/s12862-020-1582-1 (PMC6971862; doi:10.1186/s12862-020-1582-1)
Supplement: Supplementary file 8 — Additional file 8: Table S4. Detailed information of RNA editing sites in the Taxus cuspidata mitogenome. [file 12862_2020_1582_MOESM8_ESM.docx]

**Additional file 8: Table S4.** Detailed information of RNA editing sites in the *Taxus cuspidata* mitogenome.

| **Genome**  **Position** | **Strand** | **Ref**  **Base** | **Edit**  **Type** | **DNA Base Count** | | | | **RNA Base Count** | | | | **Edit**  **Efficiency** | **Genomic**  **Feature** | **Location** | **Codon**  **Phase** | **Codon**  **Change** | **Amino Acid Change** |
| --- | --- | --- | --- | --- | --- | --- | --- | --- | --- | --- | --- | --- | --- | --- | --- | --- | --- |
|  |  |  |  | **A** | **C** | **G** | **T** | **A** | **C** | **G** | **T** |  |  |  |  |  |  |
| 49746 | + | C | C->T | 0 | 1139 | 0 | 0 | 0 | 84 | 0 | 231 | 73.33% | CDS | *atp1* | **2** | ACG->ATG | T->M |
| 49753 | + | C | C->T | 0 | 1134 | 0 | 0 | 0 | 62 | 0 | 244 | 79.74% | CDS | *atp1* | **3** | CTC->CTT | L->L |
| 49761 | + | C | C->T | 0 | 1154 | 0 | 0 | 0 | 24 | 0 | 298 | 92.55% | CDS | *atp1* | **2** | CCA->CTA | P->L |
| 49764 | + | C | C->T | 0 | 1131 | 0 | 0 | 0 | 38 | 0 | 369 | 90.66% | CDS | *atp1* | **2** | TCG->TTG | S->L |
| 49826 | + | C | C->T | 0 | 1255 | 0 | 0 | 0 | 33 | 0 | 819 | 96.13% | CDS | *atp1* | **1** | CCA->TCA | P->S |
| 49852 | + | C | C->T | 0 | 1438 | 0 | 0 | 0 | 1135 | 0 | 103 | 8.32% | CDS | *atp1* | **3** | GTC->GTT | V->V |
| 49888 | + | C | C->T | 0 | 1596 | 0 | 0 | 0 | 1323 | 0 | 115 | 8.00% | CDS | *atp1* | **3** | GTC->GTT | V->V |
| 49926 | + | C | C->T | 0 | 1693 | 0 | 0 | 0 | 48 | 0 | 1658 | 97.19% | CDS | *atp1* | **2** | CCT->CTT | P->L |
| 49957 | + | C | C->T | 0 | 1811 | 0 | 0 | 0 | 1361 | 0 | 736 | 35.10% | CDS | *atp1* | **3** | TTC->TTT | F->F |
| 50149 | + | C | C->T | 2 | 2127 | 0 | 0 | 0 | 2141 | 0 | 146 | 6.38% | CDS | *atp1* | **3** | GCC->GCT | A->A |
| 50301 | + | C | C->T | 0 | 2144 | 0 | 0 | 0 | 12 | 0 | 1972 | 99.40% | CDS | *atp1* | **2** | TCA->TTA | S->L |
| 50347 | + | C | C->T | 0 | 2113 | 0 | 0 | 0 | 1675 | 1 | 144 | 7.92% | CDS | *atp1* | **3** | ACC->ACT | T->T |
| 50363 | + | C | C->T | 0 | 2101 | 0 | 0 | 0 | 70 | 0 | 1621 | 95.86% | CDS | *atp1* | **1** | CGC->TGC | R->C |
| 50369 | + | C | C->T | 0 | 2105 | 0 | 0 | 0 | 35 | 0 | 1632 | 97.90% | CDS | *atp1* | **1** | CAT->TAT | H->Y |
| 50471 | + | C | C->T | 0 | 1990 | 0 | 0 | 0 | 68 | 0 | 1047 | 93.90% | CDS | *atp1* | **1** | CCG->TCG | P->S |
| 50496 | + | C | C->T | 0 | 2076 | 0 | 1 | 0 | 18 | 0 | 917 | 98.07% | CDS | *atp1* | **2** | CCG->CTG | P->L |
| 50507 | + | C | C->T | 0 | 1977 | 0 | 0 | 0 | 39 | 0 | 788 | 95.28% | CDS | *atp1* | **1** | CCC->TCC | P->S |
| 50532 | + | C | C->T | 0 | 2004 | 0 | 0 | 0 | 20 | 0 | 908 | 97.84% | CDS | *atp1* | **2** | TCT->TTT | S->F |
| 50574 | + | C | C->T | 0 | 2099 | 0 | 1 | 0 | 15 | 0 | 749 | 98.04% | CDS | *atp1* | **2** | CCG->CTG | P->L |
| 50613 | + | C | C->T | 0 | 2095 | 0 | 0 | 0 | 13 | 0 | 504 | 97.49% | CDS | *atp1* | **2** | TCG->TTG | S->L |
| 50616 | + | C | C->T | 1 | 2044 | 0 | 0 | 0 | 14 | 0 | 489 | 97.22% | CDS | *atp1* | **2** | CCT->CTT | P->L |
| 50660 | + | C | C->T | 0 | 2187 | 0 | 0 | 0 | 19 | 0 | 421 | 95.68% | CDS | *atp1* | **1** | CAT->TAT | H->Y |
| 50664 | + | C | C->T | 0 | 2156 | 0 | 0 | 0 | 18 | 0 | 409 | 95.78% | CDS | *atp1* | **2** | TCG->TTG | S->L |
| 50671 | + | C | C->T | 0 | 2128 | 0 | 0 | 0 | 16 | 0 | 425 | 96.37% | CDS | *atp1* | **3** | TCC->TCT | S->S |
| 50679 | + | C | C->T | 0 | 2181 | 0 | 0 | 0 | 18 | 0 | 608 | 97.12% | CDS | *atp1* | **2** | TCG->TTG | S->L |
| 50792 | + | C | C->T | 0 | 2055 | 0 | 1 | 0 | 33 | 0 | 816 | 96.11% | CDS | *atp1* | **1** | CCC->TCC | P->S |
| 50813 | + | C | C->T | 1 | 2088 | 0 | 0 | 0 | 14 | 0 | 729 | 98.12% | CDS | *atp1* | **1** | CGT->TGT | R->C |
| 50817 | + | C | C->T | 1 | 2082 | 0 | 3 | 0 | 23 | 0 | 704 | 96.84% | CDS | *atp1* | **2** | CCG->CTG | P->L |
| 50829 | + | C | C->T | 0 | 2101 | 0 | 0 | 0 | 14 | 0 | 594 | 97.70% | CDS | *atp1* | **2** | CCC->CTC | P->L |
| 50870 | + | C | C->T | 0 | 2101 | 0 | 0 | 0 | 29 | 0 | 326 | 91.83% | CDS | *atp1* | **1** | CCT->TCT | P->S |
| 50921 | + | C | C->T | 0 | 2148 | 0 | 1 | 0 | 33 | 0 | 74 | 69.16% | CDS | *atp1* | **1** | CGC->TGC | R->C |
| 50931 | + | C | C->T | 0 | 2127 | 0 | 0 | 0 | 32 | 0 | 56 | 63.64% | CDS | *atp1* | **2** | TCA->TTA | S->L |
| 50937 | + | C | C->T | 1 | 2209 | 0 | 0 | 0 | 26 | 0 | 66 | 71.74% | CDS | *atp1* | **2** | CCC->CTC | P->L |
| 50943 | + | C | C->T | 0 | 2182 | 0 | 0 | 0 | 40 | 0 | 54 | 57.45% | CDS | *atp1* | **2** | TCG->TTG | S->L |
| 50970 | + | C | C->T | 1 | 2202 | 0 | 0 | 0 | 39 | 0 | 247 | 86.36% | CDS | *atp1* | **2** | TCT->TTT | S->F |
| 50979 | + | C | C->T | 0 | 2222 | 0 | 5 | 0 | 56 | 0 | 334 | 85.64% | CDS | *atp1* | **2** | TCT->TTT | S->F |
| 50991 | + | C | C->T | 0 | 2180 | 0 | 2 | 0 | 44 | 0 | 530 | 92.33% | CDS | *atp1* | **2** | CCT->CTT | P->L |
| 51045 | + | C | C->T | 0 | 2135 | 0 | 1 | 0 | 16 | 0 | 1231 | 98.72% | CDS | *atp1* | **2** | CCG->CTG | P->L |
| 51062 | + | C | C->T | 0 | 2031 | 0 | 0 | 0 | 27 | 0 | 1498 | 98.23% | CDS | *atp1* | **1** | CCA->TCA | P->S |
| 51098 | + | C | C->T | 0 | 2059 | 0 | 0 | 0 | 10 | 0 | 1773 | 99.44% | CDS | *atp1* | **1** | CAT->TAT | H->Y |
| 51163 | + | C | C->T | 0 | 2080 | 0 | 1 | 0 | 1361 | 0 | 89 | 6.14% | CDS | *atp1* | **3** | GCC->GCT | A->A |
| 51177 | + | C | C->T | 1 | 2128 | 0 | 3 | 0 | 36 | 0 | 1218 | 97.13% | CDS | *atp1* | **2** | CCA->CTA | P->L |
| 51198 | + | C | C->T | 2 | 2114 | 0 | 0 | 0 | 12 | 0 | 820 | 98.56% | CDS | *atp1* | **2** | CCA->CTA | P->L |
| 51208 | + | C | C->T | 0 | 2207 | 0 | 0 | 0 | 665 | 0 | 51 | 7.12% | CDS | *atp1* | **3** | ATC->ATT | I->I |
| 51249 | + | C | C->T | 0 | 2096 | 0 | 0 | 0 | 71 | 0 | 205 | 74.28% | CDS | *atp1* | **2** | CCA->CTA | P->L |
| 51258 | + | C | C->T | 0 | 2120 | 0 | 0 | 0 | 18 | 0 | 165 | 90.16% | CDS | *atp1* | **2** | TCT->TTT | S->F |
| 51261 | + | C | C->T | 0 | 2106 | 0 | 0 | 0 | 32 | 0 | 128 | 80.00% | CDS | *atp1* | **2** | TCA->TTA | S->L |
| 51276 | + | C | C->T | 1 | 2066 | 0 | 0 | 0 | 43 | 0 | 210 | 83.00% | CDS | *atp1* | **2** | TCA->TTA | S->L |
| 233985 | + | C | C->T | 0 | 1831 | 0 | 0 | 0 | 5 | 0 | 75 | 93.75% | CDS | *atp4* | **2** | CCA->CTA | P->L |
| 234000 | + | C | C->T | 0 | 1883 | 0 | 0 | 0 | 6 | 0 | 71 | 92.21% | CDS | *atp4* | **2** | CCA->CTA | P->L |
| 234033 | + | C | C->T | 0 | 1896 | 0 | 0 | 0 | 7 | 0 | 60 | 89.55% | CDS | *atp4* | **2** | TCA->TTA | S->L |
| 234065 | + | C | C->T | 0 | 1931 | 0 | 0 | 0 | 15 | 0 | 59 | 79.73% | CDS | *atp4* | **1** | CGT->TGT | R->C |
| 234069 | + | C | C->T | 1 | 1938 | 0 | 0 | 0 | 21 | 0 | 58 | 73.42% | CDS | *atp4* | **2** | TCC->TTC | S->F |
| 234070 | + | C | C->T | 0 | 1926 | 0 | 0 | 0 | 23 | 0 | 57 | 71.25% | CDS | *atp4* | **3** | TCC->TCT | S->S |
| 234088 | + | C | C->T | 0 | 1975 | 0 | 0 | 0 | 94 | 0 | 13 | 12.15% | CDS | *atp4* | **3** | TTC->TTT | F->F |
| 234114 | + | C | C->T | 0 | 2037 | 0 | 0 | 0 | 14 | 0 | 119 | 89.47% | CDS | *atp4* | **2** | TCC->TTT | S->F |
| 234115 | + | C | C->T | 0 | 2038 | 0 | 1 | 0 | 116 | 0 | 21 | 15.33% | CDS | *atp4* | **3** | TCC->TTT | S->F |
| 234192 | + | C | C->T | 0 | 2162 | 0 | 0 | 0 | 16 | 0 | 227 | 93.42% | CDS | *atp4* | **2** | TCT->TTT | S->F |
| 234195 | + | C | C->T | 0 | 2240 | 0 | 1 | 0 | 14 | 0 | 242 | 94.53% | CDS | *atp4* | **2** | CCG->CTG | P->L |
| 234270 | + | C | C->T | 0 | 2307 | 0 | 0 | 0 | 6 | 0 | 177 | 96.72% | CDS | *atp4* | **2** | CCG->CTG | P->L |
| 234279 | + | C | C->T | 0 | 2257 | 0 | 1 | 0 | 8 | 0 | 157 | 95.15% | CDS | *atp4* | **2** | ACG->ATG | T->M |
| 234324 | + | C | C->T | 1 | 2107 | 0 | 0 | 0 | 10 | 0 | 91 | 90.10% | CDS | *atp4* | **2** | TCA->TTA | S->L |
| 234336 | + | C | C->T | 2 | 2036 | 1 | 0 | 0 | 9 | 0 | 60 | 86.96% | CDS | *atp4* | **2** | CCA->CTA | P->L |
| 234348 | + | C | C->T | 1 | 1944 | 0 | 0 | 0 | 6 | 0 | 60 | 90.91% | CDS | *atp4* | **2** | TCA->TTA | S->L |
| 234357 | + | C | C->T | 0 | 1921 | 0 | 0 | 0 | 7 | 0 | 58 | 89.23% | CDS | *atp4* | **2** | CCT->CTT | P->L |
| 234360 | + | C | C->T | 0 | 1940 | 0 | 3 | 0 | 6 | 0 | 61 | 91.04% | CDS | *atp4* | **2** | CCA->CTA | P->L |
| 234406 | + | C | C->T | 0 | 1728 | 0 | 0 | 0 | 155 | 0 | 41 | 20.92% | CDS | *atp4* | **3** | GTC->GTT | V->V |
| 234421 | + | C | C->T | 0 | 1690 | 0 | 0 | 0 | 138 | 0 | 79 | 36.41% | CDS | *atp4* | **3** | TCC->TCT | S->S |
| 234442 | + | C | C->T | 2 | 1507 | 0 | 0 | 0 | 224 | 0 | 12 | 5.08% | CDS | *atp4* | **3** | GTC->GTT | V->V |
| 234465 | + | C | C->T | 0 | 1483 | 0 | 1 | 0 | 14 | 0 | 226 | 94.17% | CDS | *atp4* | **2** | CCC->CTC | P->L |
| 234466 | + | C | C->T | 0 | 1544 | 0 | 0 | 0 | 214 | 0 | 30 | 12.30% | CDS | *atp4* | **3** | CCC->CCT | P->L |
| 234474 | + | C | C->T | 0 | 1467 | 0 | 1 | 0 | 12 | 0 | 249 | 95.40% | CDS | *atp4* | **2** | CCG->TTG | P->L |
| 234536 | + | C | C->T | 1 | 1339 | 0 | 0 | 0 | 203 | 0 | 27 | 11.74% | CDS | *atp4* | **1** | CCG->TTG | P->L |
| 234537 | + | C | C->T | 0 | 1351 | 0 | 0 | 0 | 15 | 0 | 214 | 93.45% | CDS | *atp4* | **2** | CCG->TTG | P->L |
| 234573 | + | C | C->T | 0 | 1245 | 0 | 0 | 0 | 224 | 0 | 20 | 8.20% | CDS | *atp4* | **2** | CCG->CTG | P->L |
| 234606 | + | C | C->T | 1 | 1051 | 0 | 0 | 0 | 214 | 0 | 30 | 12.30% | CDS | *atp4* | **2** | CCC->CTC | P->L |
| 234624 | + | C | C->T | 0 | 976 | 0 | 0 | 0 | 238 | 0 | 16 | 6.30% | CDS | *atp4* | **2** | CCT->CTT | P->L |
| 237556 | + | C | C->T | 0 | 1158 | 0 | 0 | 0 | 10 | 0 | 107 | 91.45% | CDS | *atp6* | **2** | ACG->ATG | T->M |
| 237563 | + | C | C->T | 0 | 1216 | 0 | 0 | 0 | 112 | 0 | 17 | 13.18% | CDS | *atp6* | **3** | AAC->AAT | N->N |
| 237581 | + | C | C->T | 0 | 1312 | 0 | 1 | 0 | 148 | 0 | 14 | 8.64% | CDS | *atp6* | **3** | TTC->TTT | F->F |
| 237619 | + | C | C->T | 0 | 1567 | 0 | 0 | 0 | 2 | 0 | 189 | 98.95% | CDS | *atp6* | **2** | TCG->TTG | S->L |
| 237642 | + | C | C->T | 0 | 1705 | 0 | 0 | 0 | 2 | 0 | 224 | 99.12% | CDS | *atp6* | **1** | CCT->TCT | P->S |
| 237658 | + | C | C->T | 0 | 1766 | 0 | 0 | 0 | 1 | 0 | 215 | 99.54% | CDS | *atp6* | **2** | CCA->CTA | P->L |
| 237665 | + | C | C->T | 1 | 1769 | 0 | 0 | 0 | 199 | 0 | 21 | 9.55% | CDS | *atp6* | **3** | ACC->ACT | T->T |
| 237675 | + | C | C->T | 0 | 1857 | 0 | 0 | 0 | 189 | 0 | 52 | 21.58% | CDS | *atp6* | **1** | CTG->TTG | L->L |
| 237753 | + | C | C->T | 0 | 1999 | 0 | 0 | 0 | 2 | 0 | 137 | 98.56% | CDS | *atp6* | **1** | CAT->TAT | H->Y |
| 237772 | + | C | C->T | 0 | 1972 | 0 | 1 | 0 | 3 | 0 | 102 | 97.14% | CDS | *atp6* | **2** | CCG->CTG | P->L |
| 237779 | + | C | C->T | 0 | 1950 | 0 | 0 | 0 | 71 | 0 | 21 | 22.83% | CDS | *atp6* | **3** | AAC->AAT | N->N |
| 237861 | + | C | C->T | 0 | 2132 | 0 | 2 | 0 | 2 | 0 | 10 | 83.33% | CDS | *atp6* | **1** | CGT->TGT | R->C |
| 237868 | + | C | C->T | 1 | 2062 | 0 | 0 | 0 | 3 | 0 | 9 | 75.00% | CDS | *atp6* | **2** | CCC->CTC | P->L |
| 237892 | + | C | C->T | 0 | 2044 | 0 | 0 | 0 | 4 | 0 | 10 | 71.43% | CDS | *atp6* | **2** | TCC->TTC | S->F |
| 237922 | + | C | C->T | 0 | 2161 | 1 | 2 | 0 | 3 | 0 | 12 | 80.00% | CDS | *atp6* | **2** | TCG->TTG | S->L |
| 237967 | + | C | C->T | 0 | 2224 | 0 | 0 | 0 | 3 | 0 | 6 | 66.67% | CDS | *atp6* | **2** | TCT->TTT | S->F |
| 238084 | + | C | C->T | 0 | 2168 | 2 | 2 | 0 | 3 | 0 | 6 | 66.67% | CDS | *atp6* | **2** | TCA->TTA | S->L |
| 238096 | + | C | C->T | 1 | 2053 | 0 | 0 | 0 | 2 | 0 | 7 | 77.78% | CDS | *atp6* | **2** | TCA->TTA | S->L |
| 238126 | + | C | C->T | 0 | 1948 | 0 | 1 | 0 | 2 | 0 | 9 | 81.82% | CDS | *atp6* | **2** | TCA->TTA | S->L |
| 238152 | + | C | C->T | 0 | 1892 | 0 | 0 | 0 | 0 | 0 | 9 | 100.00% | CDS | *atp6* | **1** | CGG->TGG | R->W |
| 238198 | + | C | C->T | 0 | 1608 | 0 | 1 | 0 | 0 | 0 | 8 | 100.00% | CDS | *atp6* | **2** | CCC->CTC | P->L |
| 238210 | + | C | C->T | 1 | 1764 | 0 | 0 | 0 | 3 | 0 | 5 | 62.50% | CDS | *atp6* | **2** | TCA->TTA | S->L |
| 238225 | + | C | C->T | 1 | 1622 | 0 | 0 | 0 | 5 | 0 | 4 | 44.44% | CDS | *atp6* | **2** | CCG->CTG | P->L |
| 238255 | + | C | C->T | 0 | 1647 | 0 | 0 | 0 | 3 | 0 | 3 | 50.00% | CDS | *atp6* | **2** | TCA->TTA | S->L |
| 238290 | + | C | C->T | 0 | 1610 | 0 | 0 | 0 | 0 | 0 | 11 | 100.00% | CDS | *atp6* | **1** | CAC->TAC | H->Y |
| 238294 | + | C | C->T | 2 | 1659 | 0 | 1 | 0 | 2 | 0 | 9 | 81.82% | CDS | *atp6* | **2** | CCG->CTG | P->L |
| 238317 | + | C | C->T | 0 | 1564 | 0 | 0 | 0 | 0 | 0 | 7 | 100.00% | CDS | *atp6* | **1** | CAA->TAA | Q->U |
| 184988 | + | C | C->T | 0 | 1857 | 0 | 2 | 0 | 2 | 0 | 21 | 91.30% | CDS | *atp8* | **1** | CGG->TGG | R->W |
| 184994 | + | C | C->T | 0 | 1851 | 0 | 0 | 0 | 4 | 0 | 11 | 73.33% | CDS | *atp8* | **1** | CGC->TGC | R->C |
| 185049 | + | C | C->T | 0 | 1951 | 0 | 0 | 0 | 1 | 0 | 14 | 93.33% | CDS | *atp8* | **2** | CCC->CTC | P->L |
| 185061 | + | C | C->T | 0 | 2014 | 0 | 0 | 0 | 4 | 0 | 20 | 83.33% | CDS | *atp8* | **2** | CCC->CTC | P->L |
| 185067 | + | C | C->T | 0 | 2006 | 0 | 0 | 0 | 4 | 0 | 28 | 87.50% | CDS | *atp8* | **2** | CCA->CTA | P->L |
| 185101 | + | C | C->T | 0 | 2025 | 0 | 0 | 0 | 79 | 0 | 6 | 7.06% | CDS | *atp8* | **3** | ATC->ATT | I->I |
| 185102 | + | C | C->T | 2 | 2090 | 0 | 0 | 0 | 88 | 0 | 5 | 5.38% | CDS | *atp8* | **1** | CGG->TGG | R->W |
| 185136 | + | C | C->T | 0 | 2058 | 0 | 0 | 0 | 11 | 0 | 128 | 92.09% | CDS | *atp8* | **2** | TCG->TTG | S->L |
| 185148 | + | C | C->T | 0 | 2033 | 1 | 0 | 0 | 5 | 0 | 159 | 96.95% | CDS | *atp8* | **2** | TCT->TTT | S->F |
| 185152 | + | C | C->T | 0 | 2023 | 0 | 0 | 0 | 142 | 0 | 27 | 15.98% | CDS | *atp8* | **3** | CTC->CTT | L->L |
| 185194 | + | C | C->T | 0 | 1970 | 0 | 2 | 0 | 212 | 0 | 27 | 11.30% | CDS | *atp8* | **3** | TCC->TCT | S->S |
| 185243 | + | C | C->T | 0 | 1978 | 0 | 0 | 0 | 170 | 0 | 75 | 30.61% | CDS | *atp8* | **1** | CCA->TCA | P->S |
| 185244 | + | C | C->T | 0 | 1929 | 0 | 0 | 0 | 7 | 0 | 243 | 97.20% | CDS | *atp8* | **2** | CCA->CTA | P->L |
| 185293 | + | C | C->T | 0 | 1945 | 0 | 0 | 0 | 232 | 0 | 53 | 18.60% | CDS | *atp8* | 3 | ATC->ATT | I->I |
| 185343 | + | C | C->T | 2 | 1806 | 0 | 0 | 0 | 153 | 0 | 67 | 30.45% | CDS | *atp8* | **2** | TCT->TTT | S->F |
| 185384 | + | C | C->T | 0 | 1669 | 0 | 2 | 0 | 131 | 0 | 11 | 7.75% | CDS | *atp8* | 1 | CAT->TAT | H->Y |
| 185395 | + | C | C->T | 1 | 1677 | 0 | 2 | 0 | 64 | 0 | 88 | 57.89% | CDS | *atp8* | **3** | CAC->CAT | H->H |
| 148774 | - | G | C->T | 1 | 0 | 1763 | 1 | 16 | 0 | 55 | 0 | 22.54% | CDS | *ccmB* | **1** | CAT->TAT | H->Y |
| 148668 | - | G | C->T | 0 | 0 | 1862 | 1 | 14 | 0 | 35 | 0 | 28.57% | CDS | *ccmB* | **2** | TCA->TTA | S->L |
| 148533 | - | G | C->T | 0 | 0 | 1769 | 0 | 12 | 0 | 32 | 0 | 27.27% | CDS | *ccmB* | **2** | CCA->CTA | P->L |
| 148424 | - | G | C->T | 1 | 0 | 1571 | 0 | 14 | 0 | 37 | 0 | 27.45% | CDS | *ccmB* | **3** | TTC->TTT | F->F |
| 211876 | - | G | C->T | 0 | 0 | 1475 | 0 | 24 | 0 | 1 | 0 | 96.00% | CDS | *ccmC* | **2** | ACG->ATG | T->M |
| 211872 | - | G | C->T | 3 | 0 | 1578 | 0 | 16 | 0 | 7 | 0 | 69.57% | CDS | *ccmC* | **3** | TTC->TTT | F->F |
| 211838 | - | G | C->T | 0 | 0 | 1844 | 1 | 11 | 0 | 14 | 0 | 44.00% | CDS | *ccmC* | **1** | CTG->TTG | L->L |
| 211812 | - | G | C->T | 0 | 0 | 1870 | 0 | 22 | 0 | 0 | 0 | 100.00% | CDS | *ccmC* | **3** | ATC->ATT | I->I |
| 211802 | - | G | C->T | 2 | 0 | 1897 | 0 | 22 | 0 | 0 | 0 | 100.00% | CDS | *ccmC* | **1** | CGG->TGG | R->W |
| 211765 | - | G | C->T | 0 | 0 | 1955 | 0 | 9 | 0 | 3 | 0 | 75.00% | CDS | *ccmC* | **2** | CCT->CTT | P->L |
| 211744 | - | G | C->T | 0 | 0 | 2117 | 0 | 8 | 0 | 4 | 0 | 66.67% | CDS | *ccmC* | **2** | TCT->TTT | S->F |
| 211694 | - | G | C->T | 1 | 0 | 2173 | 0 | 3 | 0 | 2 | 0 | 60.00% | CDS | *ccmC* | **1** | CGG->TGG | R->W |
| 211684 | - | G | C->T | 3 | 0 | 2144 | 0 | 4 | 0 | 0 | 0 | 100.00% | CDS | *ccmC* | **2** | CCG->CTG | P->L |
| 211252 | - | G | C->T | 0 | 1 | 1850 | 0 | 5 | 0 | 3 | 0 | 62.50% | CDS | *ccmC* | **2** | TCG->TTG | S->L |
| 211222 | - | G | C->T | 0 | 0 | 1695 | 0 | 11 | 0 | 4 | 0 | 73.33% | CDS | *ccmC* | **2** | CCA->CTA | P->L |
| 211183 | - | G | C->T | 0 | 0 | 1600 | 0 | 5 | 0 | 20 | 0 | 20.00% | CDS | *ccmC* | **2** | TCT->TTT | S->F |
| 247840 | + | C | C->T | 0 | 1357 | 0 | 1 | 0 | 18 | 0 | 21 | 53.85% | CDS | *ccmFC* | **2** | CCG->CTG | P->L |
| 247858 | + | C | C->T | 0 | 1383 | 0 | 0 | 0 | 23 | 0 | 10 | 30.30% | CDS | *ccmFC* | **2** | TCC->TTC | S->F |
| 247867 | + | C | C->T | 0 | 1395 | 0 | 0 | 0 | 14 | 0 | 16 | 53.33% | CDS | *ccmFC* | **2** | TCC->TTC | S->F |
| 247879 | + | C | C->T | 0 | 1368 | 0 | 0 | 0 | 14 | 0 | 10 | 41.67% | CDS | *ccmFC* | **2** | CCT->CTT | P->L |
| 247881 | + | C | C->T | 0 | 1362 | 0 | 0 | 0 | 16 | 0 | 9 | 36.00% | CDS | *ccmFC* | **1** | CGT->TGT | R->C |
| 247916 | + | C | C->T | 0 | 1439 | 0 | 1 | 0 | 11 | 0 | 4 | 26.67% | CDS | *ccmFC* | **3** | TTC->TTT | F->F |
| 247932 | + | C | C->T | 0 | 1424 | 0 | 1 | 0 | 12 | 0 | 3 | 20.00% | CDS | *ccmFC* | **1** | CCC->TCC | P->S |
| 247936 | + | C | C->T | 0 | 1465 | 0 | 0 | 0 | 15 | 0 | 3 | 16.67% | CDS | *ccmFC* | 2 | ACA->ATA | T->I |
| 247974 | + | C | C->T | 0 | 1555 | 0 | 0 | 0 | 12 | 0 | 4 | 25.00% | CDS | *ccmFC* | **1** | CCC->TCC | P->S |
| 247975 | + | C | C->T | 1 | 1575 | 0 | 0 | 0 | 10 | 0 | 4 | 28.57% | CDS | *ccmFC* | **2** | CCC->CTC | P->L |
| 247984 | + | C | C->T | 0 | 1581 | 0 | 1 | 0 | 10 | 0 | 4 | 28.57% | CDS | *ccmFC* | **2** | CCC->CTC | P->L |
| 248010 | + | C | C->T | 0 | 1624 | 0 | 0 | 0 | 10 | 0 | 14 | 58.33% | CDS | *ccmFC* | **1** | CCC->TCC | P->S |
| 248116 | + | C | C->T | 1 | 1913 | 0 | 0 | 0 | 34 | 0 | 3 | 8.11% | CDS | *ccmFC* | **2** | CCA->CTA | P->L |
| 248207 | + | C | C->T | 1 | 2002 | 0 | 0 | 0 | 19 | 0 | 9 | 32.14% | CDS | *ccmFC* | **3** | CTC->CTT | L->L |
| 248232 | + | C | C->T | 0 | 2012 | 0 | 0 | 0 | 2 | 0 | 24 | 92.31% | CDS | *ccmFC* | **1** | CCA->TCA | P->S |
| 248244 | + | C | C->T | 0 | 2019 | 0 | 0 | 0 | 3 | 0 | 21 | 87.50% | CDS | *ccmFC* | **1** | CGC->TGC | R->C |
| 248255 | + | C | C->T | 0 | 1982 | 0 | 0 | 0 | 1 | 0 | 18 | 94.74% | CDS | *ccmFC* | **3** | CTC->CTT | L->L |
| 248267 | + | C | C->T | 1 | 1947 | 0 | 0 | 0 | 14 | 0 | 4 | 22.22% | CDS | *ccmFC* | **3** | TTC->TTT | F->F |
| 248271 | + | C | C->T | 0 | 2029 | 0 | 0 | 0 | 3 | 0 | 18 | 85.71% | CDS | *ccmFC* | **1** | CGC->TGC | R->C |
| 248400 | + | C | C->T | 1 | 1908 | 1 | 0 | 0 | 0 | 0 | 23 | 100.00% | CDS | *ccmFC* | **1** | CGC->TGC | R->C |
| 248408 | + | C | C->T | 0 | 1970 | 0 | 0 | 0 | 0 | 0 | 22 | 100.00% | CDS | *ccmFC* | **3** | TTC->TTT | F->F |
| 248567 | + | C | C->T | 0 | 2047 | 0 | 0 | 0 | 40 | 0 | 4 | 9.09% | CDS | *ccmFC* | 3 | ATC->ATT | I->I |
| 248581 | + | C | C->T | 0 | 1981 | 0 | 0 | 0 | 3 | 0 | 31 | 91.18% | CDS | *ccmFC* | **2** | CCG->CTG | P->L |
| 248587 | + | C | C->T | 0 | 1998 | 0 | 0 | 0 | 14 | 0 | 27 | 65.85% | CDS | *ccmFC* | **2** | TCG->TTG | S->L |
| 248601 | + | C | C->T | 0 | 2035 | 0 | 0 | 0 | 39 | 0 | 3 | 7.14% | CDS | *ccmFC* | 1 | CAT->TAT | H->Y |
| 248649 | + | C | C->T | 0 | 1916 | 1 | 0 | 0 | 32 | 0 | 4 | 11.11% | CDS | *ccmFC* | 1 | CCA->TCA | P->S |
| 248661 | + | C | C->T | 0 | 1902 | 0 | 0 | 0 | 26 | 0 | 6 | 18.75% | CDS | *ccmFC* | 1 | CCC->TCC | P->S |
| 248663 | + | C | C->T | 0 | 1995 | 0 | 1 | 0 | 16 | 0 | 18 | 52.94% | CDS | *ccmFC* | **3** | CCC->CCT | P->P |
| 248686 | + | C | C->T | 0 | 1942 | 0 | 1 | 0 | 24 | 0 | 9 | 27.27% | CDS | *ccmFC* | **2** | TCG->TTG | S->L |
| 248764 | + | C | C->T | 0 | 2013 | 0 | 0 | 0 | 4 | 0 | 14 | 77.78% | CDS | *ccmFC* | **2** | TCT->TTT | S->F |
| 248766 | + | C | C->T | 0 | 2005 | 0 | 0 | 0 | 6 | 0 | 11 | 64.71% | CDS | *ccmFC* | **1** | CTC->TTC | L->F |
| 248800 | + | C | C->T | 0 | 1957 | 0 | 0 | 0 | 3 | 0 | 6 | 66.67% | CDS | *ccmFC* | **2** | GCG->GTG | A->V |
| 244902 | + | C | C->T | 0 | 832 | 0 | 0 | 0 | 56 | 0 | 6 | 9.68% | CDS | *ccmFN* | 2 | ACG->ATG | T->M |
| 244938 | + | C | C->T | 0 | 1053 | 0 | 0 | 0 | 66 | 0 | 14 | 17.50% | CDS | *ccmFN* | 2 | CCG->CTG | P->L |
| 244929 | + | C | C->T | 1 | 983 | 0 | 0 | 0 | 59 | 0 | 19 | 24.36% | CDS | *ccmFN* | **2** | CCG->CTG | P->L |
| 244947 | + | C | C->T | 0 | 1063 | 0 | 0 | 0 | 61 | 0 | 18 | 22.78% | CDS | *ccmFN* | **2** | TCC->TTC | S->F |
| 244998 | + | C | C->T | 0 | 1153 | 0 | 0 | 0 | 49 | 0 | 13 | 20.97% | CDS | *ccmFN* | **2** | CCT->CTT | P->L |
| 245051 | + | C | C->T | 0 | 1113 | 0 | 0 | 0 | 32 | 0 | 5 | 13.51% | CDS | *ccmFN* | 1 | CCT->TCT | P->S |
| 245110 | + | C | C->T | 0 | 1192 | 0 | 0 | 0 | 26 | 0 | 5 | 16.13% | CDS | *ccmFN* | 3 | CCC->CCT | P->P |
| 245157 | + | C | C->T | 2 | 1286 | 0 | 0 | 0 | 23 | 0 | 7 | 23.33% | CDS | *ccmFN* | **2** | CCA->CTA | P->L |
| 245190 | + | C | C->T | 0 | 1478 | 0 | 0 | 0 | 50 | 0 | 19 | 27.54% | CDS | *ccmFN* | **2** | CCC->CTC | P->L |
| 245262 | + | C | C->T | 0 | 1503 | 0 | 0 | 0 | 56 | 0 | 52 | 48.15% | CDS | *ccmFN* | **2** | TCG->TTG | S->L |
| 245311 | + | C | C->T | 0 | 1431 | 0 | 0 | 0 | 96 | 0 | 16 | 14.29% | CDS | *ccmFN* | 3 | TTC->TTT | F->F |
| 245498 | + | C | C->T | 0 | 1375 | 0 | 0 | 0 | 22 | 0 | 30 | 57.69% | CDS | *ccmFN* | **1** | CTT->TTT | L->F |
| 245589 | + | C | C->T | 0 | 1345 | 0 | 0 | 0 | 22 | 0 | 6 | 21.43% | CDS | *ccmFN* | **2** | TCC->TTC | S->F |
| 245604 | + | C | C->T | 0 | 1383 | 0 | 0 | 0 | 28 | 0 | 4 | 12.50% | CDS | *ccmFN* | 2 | TCG->TTG | S->L |
| 245642 | + | C | C->T | 0 | 1368 | 0 | 0 | 0 | 21 | 0 | 9 | 30.00% | CDS | *ccmFN* | **1** | CGT->TGT | R->C |
| 245655 | + | C | C->T | 0 | 1326 | 0 | 0 | 0 | 14 | 0 | 19 | 57.58% | CDS | *ccmFN* | **2** | CCT->CTT | P->L |
| 245664 | + | C | C->T | 0 | 1374 | 0 | 0 | 0 | 21 | 0 | 15 | 41.67% | CDS | *ccmFN* | **2** | TCA->TTA | S->L |
| 245671 | + | C | C->T | 1 | 1336 | 0 | 0 | 0 | 26 | 0 | 9 | 25.71% | CDS | *ccmFN* | **3** | CCC->CCT | P->P |
| 245676 | + | C | C->T | 1 | 1336 | 0 | 0 | 0 | 17 | 0 | 13 | 43.33% | CDS | *ccmFN* | **2** | CCG->CTG | P->L |
| 245751 | + | C | C->T | 0 | 1172 | 0 | 0 | 0 | 18 | 0 | 39 | 68.42% | CDS | *ccmFN* | **2** | CCA->CTA | P->L |
| 245761 | + | C | C->T | 0 | 1163 | 0 | 0 | 0 | 59 | 0 | 12 | 16.90% | CDS | *ccmFN* | 3 | TAC->TAT | Y->Y |
| 246293 | + | C | C->T | 0 | 2013 | 0 | 0 | 0 | 22 | 0 | 39 | 63.93% | CDS | *ccmFN* | **1** | CCG->TCG | P->S |
| 246294 | + | C | C->T | 0 | 2110 | 0 | 0 | 0 | 33 | 0 | 29 | 46.77% | CDS | *ccmFN* | **2** | CCG->CTG | P->L |
| 246302 | + | C | C->T | 1 | 2034 | 0 | 0 | 0 | 24 | 0 | 31 | 56.36% | CDS | *ccmFN* | **1** | CGG->TGG | R->W |
| 246330 | + | C | C->T | 0 | 2050 | 0 | 5 | 0 | 26 | 0 | 7 | 21.21% | CDS | *ccmFN* | **2** | CCA->CTA | P->L |
| 246362 | + | C | C->T | 1 | 1985 | 0 | 0 | 0 | 17 | 0 | 4 | 19.05% | CDS | *ccmFN* | 1 | CGG->TGG | R->W |
| 246431 | + | C | C->T | 0 | 2053 | 0 | 0 | 0 | 39 | 0 | 5 | 11.36% | CDS | *ccmFN* | 1 | CGT->TGT | R->C |
| 246440 | + | C | C->T | 0 | 1975 | 0 | 0 | 0 | 38 | 0 | 4 | 9.52% | CDS | *ccmFN* | 1 | CCA->TCA | P->S |
| 246537 | + | C | C->T | 0 | 1681 | 0 | 0 | 0 | 29 | 0 | 4 | 12.12% | CDS | *ccmFN* | 2 | TCG->TTG | S->L |
| 246588 | + | C | C->T | 0 | 1514 | 0 | 0 | 0 | 18 | 0 | 12 | 40.00% | CDS | *ccmFN* | **2** | CCA->CTA | P->L |
| 246599 | + | C | C->T | 0 | 1403 | 0 | 0 | 0 | 26 | 0 | 12 | 31.58% | CDS | *ccmFN* | **1** | CTC->TTC | L->F |
| 246603 | + | C | C->T | 1 | 1371 | 0 | 0 | 0 | 25 | 0 | 19 | 43.18% | CDS | *ccmFN* | **2** | CCT->CTT | P->L |
| 246624 | + | C | C->T | 1 | 1244 | 0 | 0 | 0 | 18 | 0 | 37 | 67.27% | CDS | *ccmFN* | **2** | CCG->CTG | P->L |
| 246631 | + | C | C->T | 0 | 1269 | 0 | 0 | 0 | 22 | 0 | 35 | 61.40% | CDS | *ccmFN* | **3** | CTC->CTT | L->L |
| 246643 | + | C | C->T | 0 | 1215 | 0 | 0 | 0 | 53 | 0 | 7 | 11.67% | CDS | *ccmFN* | 3 | ATC->ATT | I->I |
| 246685 | + | C | C->T | 0 | 1124 | 0 | 0 | 0 | 71 | 0 | 5 | 6.58% | CDS | *ccmFN* | 3 | CCC->CCT | P->P |
| 246690 | + | C | C->T | 0 | 1095 | 0 | 0 | 0 | 65 | 0 | 13 | 16.67% | CDS | *ccmFN* | 2 | ACG->ATG | T->M |
| 246712 | + | C | C->T | 0 | 1073 | 0 | 0 | 0 | 85 | 0 | 12 | 12.37% | CDS | *ccmFN* | 3 | CGC->CGT | R->R |
| 183212 | + | C | C->T | 0 | 2397 | 0 | 0 | 0 | 12 | 0 | 6 | 33.33% | CDS | *cob* | **1** | CGG->TGG | R->W |
| 183230 | + | C | C->T | 0 | 2396 | 0 | 1 | 0 | 13 | 0 | 4 | 23.53% | CDS | *cob* | **1** | CGG->TGG | R->W |
| 183255 | + | C | C->T | 1 | 2399 | 2 | 0 | 0 | 12 | 0 | 8 | 40.00% | CDS | *cob* | **2** | TCA->TTA | S->L |
| 183288 | + | C | C->T | 1 | 2362 | 1 | 0 | 0 | 18 | 0 | 3 | 14.29% | CDS | *cob* | **2** | ACA->ATA | T->I |
| 183351 | + | C | C->T | 1 | 2398 | 0 | 1 | 0 | 18 | 0 | 3 | 14.29% | CDS | *cob* | **2** | CCT->CTT | P->L |
| 183375 | + | C | C->T | 0 | 2360 | 0 | 0 | 0 | 13 | 0 | 9 | 40.91% | CDS | *cob* | **2** | CCA->CTA | P->L |
| 183503 | + | C | C->T | 0 | 2276 | 0 | 1 | 0 | 6 | 0 | 174 | 96.67% | CDS | *cob* | **1** | CGG->TGG | R->W |
| 183513 | + | C | C->T | 1 | 2310 | 1 | 0 | 0 | 16 | 0 | 168 | 91.30% | CDS | *cob* | **2** | TCC->TTC | S->F |
| 183521 | + | C | C->T | 1 | 2299 | 0 | 0 | 0 | 10 | 0 | 192 | 95.05% | CDS | *cob* | **1** | CTC->TTC | L->F |
| 183525 | + | C | C->T | 0 | 2347 | 0 | 0 | 0 | 188 | 0 | 13 | 6.47% | CDS | *cob* | 2 | TCT->TTT | S->F |
| 183540 | + | C | C->T | 0 | 2297 | 0 | 0 | 0 | 20 | 0 | 165 | 89.19% | CDS | *cob* | **2** | TCT->TTT | S->F |
| 183630 | + | C | C->T | 0 | 2000 | 0 | 0 | 0 | 9 | 0 | 61 | 87.14% | CDS | *cob* | **2** | TCC->TTC | S->F |
| 183633 | + | C | C->T | 0 | 1963 | 0 | 0 | 0 | 9 | 0 | 59 | 86.76% | CDS | *cob* | **2** | CCA->CTA | P->L |
| 183641 | + | C | C->T | 0 | 2061 | 0 | 0 | 0 | 6 | 0 | 39 | 86.67% | CDS | *cob* | **1** | CAT->TAT | H->Y |
| 183651 | + | C | C->T | 0 | 1967 | 1 | 0 | 0 | 4 | 0 | 39 | 90.70% | CDS | *cob* | **2** | CCT->CTT | P->L |
| 183672 | + | C | C->T | 0 | 2001 | 0 | 1 | 0 | 4 | 0 | 71 | 94.67% | CDS | *cob* | **2** | TCA->TTA | S->L |
| 183704 | + | C | C->T | 1 | 2022 | 0 | 0 | 0 | 5 | 0 | 95 | 95.00% | CDS | *cob* | **1** | CCA->TCA | P->S |
| 183705 | + | C | C->T | 0 | 1988 | 0 | 1 | 0 | 4 | 0 | 99 | 96.12% | CDS | *cob* | **2** | CCA->CTA | P->L |
| 183785 | + | C | C->T | 0 | 1966 | 0 | 0 | 0 | 13 | 0 | 133 | 91.10% | CDS | *cob* | **1** | CGG->TGG | R->W |
| 183810 | + | C | C->T | 0 | 1787 | 0 | 0 | 0 | 24 | 0 | 85 | 77.98% | CDS | *cob* | **2** | CCA->CTA | P->L |
| 183813 | + | C | C->T | 1 | 1753 | 1 | 2 | 0 | 29 | 0 | 80 | 73.39% | CDS | *cob* | **2** | TCA->TTA | S->L |
| 183818 | + | C | C->T | 0 | 1745 | 0 | 0 | 0 | 26 | 0 | 79 | 75.24% | CDS | *cob* | **1** | CGG->TGG | R->W |
| 183869 | + | C | C->T | 0 | 1296 | 0 | 0 | 0 | 55 | 0 | 169 | 75.45% | CDS | *cob* | **1** | CCT->TCT | P->S |
| 183872 | + | C | C->T | 1 | 1310 | 0 | 0 | 0 | 20 | 0 | 211 | 91.34% | CDS | *cob* | **1** | CCA->TCA | P->S |
| 183881 | + | C | C->T | 0 | 1173 | 0 | 0 | 0 | 14 | 0 | 243 | 94.55% | CDS | *cob* | **1** | CTC->TTC | L->F |
| 183886 | + | C | C->T | 0 | 1167 | 0 | 0 | 0 | 208 | 0 | 80 | 27.78% | CDS | *cob* | **3** | TTC->TTT | F->F |
| 183955 | + | C | C->T | 0 | 821 | 0 | 0 | 0 | 358 | 0 | 53 | 12.90% | CDS | *cob* | **3** | CAC->CAT | H->H |
| 227273 | - | G | C->T | 2 | 0 | 610 | 1 | 166 | 0 | 125 | 0 | 57.04% | CDS | *cox1* | **2** | ACG->ATG | T->M |
| 227244 | - | G | C->T | 0 | 0 | 805 | 0 | 415 | 0 | 15 | 0 | 96.51% | CDS | *cox1* | **1** | CCC->TCC | P->S |
| 227214 | - | G | C->T | 0 | 0 | 1014 | 0 | 510 | 0 | 20 | 0 | 96.23% | CDS | *cox1* | **1** | CAT->TAT | H->Y |
| 227210 | - | G | C->T | 0 | 0 | 1030 | 0 | 501 | 0 | 14 | 0 | 97.28% | CDS | *cox1* | **2** | TCA->TTA | S->L |
| 227162 | - | G | C->T | 0 | 0 | 1445 | 0 | 389 | 0 | 32 | 0 | 92.40% | CDS | *cox1* | **2** | CCA->CTA | P->L |
| 227075 | - | G | C->T | 0 | 0 | 1494 | 0 | 245 | 0 | 27 | 0 | 90.07% | CDS | *cox1* | **2** | TCT->TTT | S->F |
| 227072 | - | G | C->T | 0 | 2 | 1519 | 0 | 214 | 0 | 47 | 0 | 81.99% | CDS | *cox1* | **2** | CCC->CTC | P->L |
| 227069 | - | G | C->T | 2 | 0 | 1545 | 0 | 235 | 0 | 23 | 0 | 91.09% | CDS | *cox1* | **2** | ACG->ATG | T->M |
| 227066 | - | G | C->T | 0 | 0 | 1541 | 0 | 232 | 0 | 37 | 0 | 86.25% | CDS | *cox1* | **2** | ACC->ATC | T->I |
| 227006 | - | G | C->T | 0 | 0 | 1466 | 0 | 272 | 0 | 46 | 0 | 85.53% | CDS | *cox1* | **2** | CCT->CTT | P->L |
| 226958 | - | G | C->T | 0 | 0 | 1642 | 0 | 406 | 0 | 41 | 0 | 90.83% | CDS | *cox1* | **2** | TCC->TTC | S->F |
| 226956 | - | G | C->T | 0 | 0 | 1699 | 0 | 414 | 0 | 39 | 0 | 91.39% | CDS | *cox1* | **1** | CGG->TGG | R->W |
| 226920 | - | G | C->T | 0 | 0 | 1723 | 0 | 450 | 0 | 24 | 0 | 94.94% | CDS | *cox1* | **1** | CCA->TCA | P->S |
| 226913 | - | G | C->T | 1 | 0 | 1773 | 1 | 427 | 0 | 30 | 0 | 93.44% | CDS | *cox1* | **2** | TCG->TTG | S->L |
| 226829 | - | G | C->T | 0 | 0 | 2291 | 1 | 29 | 0 | 16 | 0 | 64.44% | CDS | *cox1* | **2** | CCC->CTC | P->L |
| 226828 | - | G | C->T | 0 | 0 | 2332 | 1 | 28 | 0 | 16 | 0 | 63.64% | CDS | *cox1* | **3** | CCC->CCT | P->P |
| 226814 | - | G | C->T | 0 | 0 | 2371 | 0 | 4 | 0 | 16 | 0 | 20.00% | CDS | *cox1* | **2** | CCT->CTT | P->L |
| 226797 | - | G | C->T | 0 | 0 | 2339 | 1 | 9 | 0 | 23 | 0 | 28.13% | CDS | *cox1* | **1** | CCA->TCA | P->S |
| 226608 | - | G | C->T | 1 | 1 | 2272 | 0 | 167 | 0 | 5 | 0 | 97.09% | CDS | *cox1* | **1** | CCT->TCT | P->S |
| 226607 | - | G | C->T | 0 | 0 | 2260 | 0 | 166 | 0 | 11 | 0 | 93.79% | CDS | *cox1* | **2** | CCT->CTT | P->L |
| 226551 | - | G | C->T | 0 | 0 | 2325 | 0 | 14 | 0 | 8 | 0 | 63.64% | CDS | *cox1* | **1** | CTC->TTC | L->F |
| 226520 | - | G | C->T | 1 | 0 | 2321 | 1 | 61 | 0 | 6 | 0 | 91.04% | CDS | *cox1* | **2** | CCG->CTG | P->L |
| 226504 | - | G | C->T | 0 | 0 | 2290 | 0 | 38 | 0 | 71 | 0 | 34.86% | CDS | *cox1* | **3** | ATC->ATT | I->I |
| 226479 | - | G | C->T | 1 | 0 | 2295 | 0 | 233 | 0 | 8 | 0 | 96.68% | CDS | *cox1* | **1** | CCG->TCG | P->S |
| 226433 | - | G | C->T | 1 | 1 | 2282 | 0 | 218 | 0 | 16 | 0 | 93.16% | CDS | *cox1* | **2** | ACC->ATC | T->I |
| 226427 | - | G | C->T | 1 | 0 | 2286 | 0 | 224 | 0 | 10 | 0 | 95.73% | CDS | *cox1* | **2** | ACT->ATT | T->I |
| 226404 | - | G | C->T | 0 | 0 | 2318 | 2 | 310 | 0 | 7 | 0 | 97.79% | CDS | *cox1* | **1** | CGG->TGG | R->W |
| 226356 | - | G | C->T | 1 | 0 | 2231 | 0 | 242 | 0 | 11 | 0 | 95.65% | CDS | *cox1* | **1** | CAC->TAC | H->Y |
| 226354 | - | G | C->T | 0 | 0 | 2292 | 0 | 242 | 0 | 13 | 0 | 94.90% | CDS | *cox1* | **3** | CAC->CAT | H->H |
| 226352 | - | G | C->T | 0 | 0 | 2246 | 1 | 243 | 0 | 15 | 0 | 94.19% | CDS | *cox1* | **2** | TCT->TTT | S->F |
| 226250 | - | G | C->T | 0 | 0 | 2390 | 0 | 72 | 0 | 8 | 0 | 90.00% | CDS | *cox1* | **2** | CCA->CTA | P->L |
| 226247 | - | G | C->T | 1 | 1 | 2428 | 0 | 68 | 0 | 12 | 0 | 85.00% | CDS | *cox1* | **2** | TCT->TTT | S->F |
| 226235 | - | G | C->T | 0 | 0 | 2402 | 0 | 45 | 0 | 7 | 0 | 86.54% | CDS | *cox1* | **2** | TCC->TTC | S->F |
| 226155 | - | G | C->T | 1 | 0 | 2407 | 0 | 6 | 0 | 15 | 0 | 28.57% | CDS | *cox1* | **1** | CAT->TAT | H->Y |
| 226040 | - | G | C->T | 1 | 0 | 2367 | 0 | 282 | 0 | 10 | 0 | 96.58% | CDS | *cox1* | **2** | TCA->TTA | S->L |
| 226023 | - | G | C->T | 0 | 0 | 2277 | 0 | 371 | 0 | 15 | 0 | 96.11% | CDS | *cox1* | **1** | CGG->TGG | R->W |
| 225993 | - | G | C->T | 0 | 0 | 2115 | 0 | 479 | 0 | 17 | 0 | 96.57% | CDS | *cox1* | **1** | CTC->TTC | L->F |
| 225974 | - | G | C->T | 3 | 0 | 2057 | 0 | 576 | 0 | 21 | 0 | 96.48% | CDS | *cox1* | **2** | TCG->TTG | S->L |
| 225891 | - | G | C->T | 0 | 0 | 1702 | 0 | 254 | 0 | 284 | 0 | 47.21% | CDS | *cox1* | **1** | CAT->TAT | H->Y |
| 225870 | - | G | C->T | 0 | 0 | 1688 | 0 | 451 | 0 | 12 | 0 | 97.41% | CDS | *cox1* | **1** | CGT->TGT | R->C |
| 225867 | - | G | C->T | 1 | 0 | 1735 | 0 | 418 | 0 | 32 | 0 | 92.89% | CDS | *cox1* | **1** | CGT->TGT | R->C |
| 225860 | - | G | C->T | 1 | 0 | 1634 | 0 | 425 | 0 | 34 | 0 | 92.59% | CDS | *cox1* | **2** | TCC->TTC | S->F |
| 225787 | - | G | C->T | 0 | 0 | 1520 | 0 | 348 | 0 | 197 | 0 | 63.85% | CDS | *cox1* | **3** | ACC->ACT | T->T |
| 190082 | + | C | C->T | 0 | 1434 | 0 | 0 | 0 | 410 | 0 | 60 | 12.77% | CDS | *cox2* | 3 | TTC->TTT | F->F |
| 190083 | + | C | C->T | 1 | 1452 | 0 | 0 | 0 | 172 | 0 | 301 | 63.64% | CDS | *cox2* | **1** | CTC->TTC | L->F |
| 190088 | + | C | C->T | 0 | 1510 | 0 | 0 | 0 | 417 | 0 | 53 | 11.28% | CDS | *cox2* | 3 | TCC->TCT | S->S |
| 190116 | + | C | C->T | 0 | 1498 | 0 | 0 | 0 | 265 | 0 | 190 | 41.76% | CDS | *cox2* | **1** | CCT->TCT | P->S |
| 190150 | + | C | C->T | 0 | 1481 | 0 | 0 | 0 | 42 | 0 | 194 | 82.20% | CDS | *cox2* | **2** | TCT->TTT | S->F |
| 190192 | + | C | C->T | 0 | 1558 | 0 | 0 | 0 | 38 | 0 | 97 | 71.85% | CDS | *cox2* | **2** | TCA->TTA | S->L |
| 190216 | + | C | C->T | 0 | 1609 | 0 | 1 | 0 | 36 | 0 | 47 | 56.63% | CDS | *cox2* | **2** | CCA->CTA | P->L |
| 190228 | + | C | C->T | 0 | 1622 | 0 | 0 | 0 | 33 | 0 | 26 | 44.07% | CDS | *cox2* | **2** | TCG->TTG | S->L |
| 190240 | + | C | C->T | 1 | 1583 | 0 | 0 | 0 | 57 | 0 | 6 | 9.52% | CDS | *cox2* | 2 | TCA->TTA | S->L |
| 190242 | + | C | C->T | 0 | 1585 | 0 | 0 | 0 | 41 | 0 | 20 | 32.79% | CDS | *cox2* | **1** | CGG->TGG | R->W |
| 190261 | + | C | C->T | 0 | 1642 | 0 | 0 | 0 | 31 | 0 | 57 | 64.77% | CDS | *cox2* | **2** | TCA->TTA | S->L |
| 190332 | + | C | C->T | 0 | 1823 | 0 | 3 | 0 | 33 | 0 | 150 | 81.97% | CDS | *cox2* | **1** | CGG->TGG | R->W |
| 190343 | + | C | C->T | 0 | 1855 | 0 | 0 | 0 | 98 | 0 | 86 | 46.74% | CDS | *cox2* | **3** | TTC->TTT | F->F |
| 190357 | + | C | C->T | 0 | 1905 | 0 | 0 | 0 | 37 | 0 | 146 | 79.78% | CDS | *cox2* | **2** | CCG->CTG | P->L |
| 190364 | + | C | C->T | 0 | 1893 | 0 | 0 | 0 | 174 | 0 | 14 | 7.45% | CDS | *cox2* | 3 | TTC->TTT | F->F |
| 190367 | + | C | C->T | 0 | 1916 | 0 | 3 | 0 | 178 | 0 | 11 | 5.82% | CDS | *cox2* | 3 | ATC->ATT | I->I |
| 190387 | + | C | C->T | 0 | 1869 | 0 | 1 | 0 | 26 | 0 | 142 | 84.52% | CDS | *cox2* | **2** | CCG->CTG | P->L |
| 190403 | + | C | C->T | 0 | 1935 | 0 | 0 | 0 | 117 | 0 | 20 | 14.60% | CDS | *cox2* | 3 | GAC->GAT | D->D |
| 190452 | + | C | C->T | 0 | 2023 | 0 | 0 | 0 | 52 | 0 | 45 | 46.39% | CDS | *cox2* | **1** | CGG->TGG | R->W |
| 190458 | + | C | C->T | 0 | 2035 | 0 | 0 | 0 | 92 | 0 | 11 | 10.68% | CDS | *cox2* | 1 | CGG->TGG | R->W |
| 168011 | + | C | C->T | 0 | 1201 | 0 | 2 | 0 | 9 | 0 | 31 | 77.50% | CDS | *cox2* | **2** | TCT->TTT | S->F |
| 168027 | + | C | C->T | 0 | 1142 | 0 | 0 | 0 | 26 | 0 | 18 | 40.91% | CDS | *cox2* | **3** | ATC->ATT | I->I |
| 168041 | + | C | C->T | 1 | 1131 | 0 | 1 | 0 | 10 | 0 | 32 | 76.19% | CDS | *cox2* | **2** | CCA->CTA | P->L |
| 168056 | + | C | C->T | 0 | 1150 | 0 | 0 | 0 | 12 | 0 | 45 | 78.95% | CDS | *cox2* | **2** | TCG->TTG | S->L |
| 168065 | + | C | C->T | 0 | 1141 | 1 | 0 | 0 | 23 | 0 | 52 | 69.33% | CDS | *cox2* | **2** | TCG->TTG | S->L |
| 168107 | + | C | C->T | 0 | 1093 | 0 | 2 | 0 | 30 | 0 | 156 | 83.87% | CDS | *cox2* | **2** | CCA->CTA | P->L |
| 168134 | + | C | C->T | 0 | 1048 | 0 | 0 | 0 | 17 | 0 | 202 | 92.24% | CDS | *cox2* | **2** | GCA->GTA | A->V |
| 168145 | + | C | C->T | 0 | 1042 | 0 | 0 | 0 | 9 | 0 | 225 | 96.15% | CDS | *cox2* | **1** | CGG->TGG | R->W |
| 168212 | + | C | C->T | 0 | 995 | 0 | 0 | 0 | 7 | 0 | 191 | 96.46% | CDS | *cox2* | **2** | CCG->CTG | P->L |
| 168256 | + | C | C->T | 0 | 820 | 0 | 0 | 0 | 12 | 0 | 172 | 93.48% | CDS | *cox2* | **1** | CGC->TGC | R->C |
| 168273 | + | C | C->T | 0 | 717 | 0 | 0 | 0 | 145 | 0 | 24 | 14.20% | CDS | *cox2* | 3 | GCC->GCT | A->A |
| 168322 | + | C | C->T | 0 | 441 | 0 | 0 | 0 | 3 | 0 | 83 | 96.51% | CDS | *cox2* | **1** | CGG->TGG | R->W |
| 187618 | + | C | C->T | 0 | 952 | 0 | 1 | 0 | 7 | 0 | 105 | 93.75% | CDS | *cox3* | **2** | ACG->ATG | T->M |
| 187635 | + | C | C->T | 0 | 1041 | 0 | 0 | 0 | 6 | 0 | 145 | 96.03% | CDS | *cox3* | **1** | CAT->TAT | H->Y |
| 187679 | + | C | C->T | 0 | 1194 | 0 | 0 | 0 | 130 | 0 | 49 | 27.37% | CDS | *cox3* | **3** | CTC->CTT | L->L |
| 187727 | + | C | C->T | 0 | 1274 | 0 | 0 | 0 | 185 | 0 | 17 | 8.42% | CDS | *cox3* | 3 | TTC->TTT | F->F |
| 187766 | + | C | C->T | 0 | 1472 | 0 | 1 | 0 | 133 | 0 | 33 | 19.88% | CDS | *cox3* | 3 | TTC->TTT | F->F |
| 187771 | + | C | C->T | 0 | 1476 | 1 | 0 | 0 | 5 | 0 | 161 | 96.99% | CDS | *cox3* | **2** | CCA->CTA | P->L |
| 187788 | + | C | C->T | 0 | 1432 | 0 | 4 | 0 | 6 | 0 | 168 | 96.55% | CDS | *cox3* | **1** | CGG->TGG | R->W |
| 187841 | + | C | C->T | 0 | 1619 | 0 | 0 | 0 | 16 | 0 | 123 | 88.49% | CDS | *cox3* | **3** | GTC->GTT | V->V |
| 187872 | + | C | C->T | 0 | 1760 | 0 | 0 | 0 | 95 | 0 | 35 | 26.92% | CDS | *cox3* | **1** | CCG->TCG | P->S |
| 187873 | + | C | C->T | 0 | 1767 | 0 | 2 | 0 | 9 | 0 | 116 | 92.80% | CDS | *cox3* | **2** | CCG->CTG | P->L |
| 187880 | + | C | C->T | 1 | 1813 | 0 | 0 | 0 | 120 | 0 | 7 | 5.51% | CDS | *cox3* | 3 | ATC->ATT | I->I |
| 187899 | + | C | C->T | 0 | 1785 | 0 | 0 | 0 | 13 | 0 | 109 | 89.34% | CDS | *cox3* | **1** | CTC->TTC | L->F |
| 187914 | + | C | C->T | 0 | 1888 | 0 | 1 | 0 | 6 | 0 | 121 | 95.28% | CDS | *cox3* | **1** | CGG->TGG | R->W |
| 187965 | + | C | C->T | 1 | 1878 | 0 | 0 | 0 | 7 | 0 | 138 | 95.17% | CDS | *cox3* | **1** | CGG->TGG | R->W |
| 188030 | + | C | C->T | 0 | 1912 | 0 | 0 | 0 | 87 | 0 | 106 | 54.92% | CDS | *cox3* | **3** | CTC->CTT | L->L |
| 188039 | + | C | C->T | 0 | 1874 | 0 | 1 | 0 | 21 | 0 | 168 | 88.89% | CDS | *cox3* | **3** | TCC->TCT | S->S |
| 188164 | + | C | C->T | 0 | 2159 | 0 | 0 | 0 | 2 | 0 | 94 | 97.92% | CDS | *cox3* | **2** | TCT->TTT | S->F |
| 188196 | + | C | C->T | 0 | 2181 | 0 | 1 | 0 | 0 | 0 | 61 | 100.00% | CDS | *cox3* | **1** | CAC->TAC | H->Y |
| 188198 | + | C | C->T | 0 | 2216 | 0 | 0 | 0 | 15 | 0 | 43 | 74.14% | CDS | *cox3* | **3** | CAC->CAT | H->H |
| 188202 | + | C | C->T | 0 | 2098 | 0 | 0 | 0 | 1 | 0 | 55 | 98.21% | CDS | *cox3* | **1** | CCT->TCT | P->S |
| 188213 | + | C | C->T | 0 | 2259 | 0 | 0 | 0 | 65 | 0 | 9 | 12.16% | CDS | *cox3* | 3 | TTC->TTT | F->F |
| 188215 | + | C | C->T | 0 | 2218 | 0 | 0 | 0 | 0 | 0 | 82 | 100.00% | CDS | *cox3* | **2** | TCA->TTA | S->L |
| 188261 | + | C | C->T | 0 | 2032 | 0 | 1 | 0 | 126 | 0 | 37 | 22.70% | CDS | *cox3* | **3** | TTC->TTT | F->F |
| 188263 | + | C | C->T | 0 | 2056 | 0 | 0 | 0 | 4 | 0 | 166 | 97.65% | CDS | *cox3* | **2** | CCA->CTA | P->L |
| 188290 | + | C | C->T | 1 | 1954 | 1 | 0 | 0 | 68 | 0 | 112 | 62.22% | CDS | *cox3* | **2** | TCG->TTG | S->L |
| 188351 | + | C | C->T | 2 | 1702 | 0 | 0 | 0 | 75 | 0 | 21 | 21.88% | CDS | *cox3* | **3** | TCC->TCT | S->S |
| 188364 | + | C | C->T | 0 | 1566 | 0 | 1 | 0 | 1 | 0 | 74 | 98.67% | CDS | *cox3* | **1** | CGG->TGG | R->W |
| 188374 | + | C | C->T | 0 | 1493 | 0 | 0 | 0 | 1 | 0 | 71 | 98.61% | CDS | *cox3* | **2** | CCA->CTA | P->L |
| 188387 | + | C | C->T | 0 | 1467 | 0 | 1 | 0 | 60 | 0 | 29 | 32.58% | CDS | *cox3* | **3** | ATC->ATT | I->I |
| 188404 | + | C | C->T | 0 | 1452 | 0 | 0 | 0 | 13 | 0 | 101 | 88.60% | CDS | *cox3* | **2** | CCA->CTA | P->L |
| 101555 | - | G | C->T | 0 | 1 | 1675 | 0 | 40 | 0 | 19 | 0 | 67.80% | CDS | *matR* | **2** | TCC->TTC | S->F |
| 101390 | - | G | C->T | 1 | 0 | 1192 | 1 | 10 | 0 | 32 | 0 | 23.81% | CDS | *matR* | **2** | TCA->TTA | S->L |
| 101365 | - | G | C->T | 0 | 0 | 1146 | 0 | 13 | 0 | 37 | 0 | 26.00% | CDS | *matR* | **3** | ATC->ATT | I->I |
| 101325 | - | G | C->T | 0 | 0 | 1003 | 0 | 31 | 0 | 29 | 0 | 51.67% | CDS | *matR* | **1** | CCA->TCA | P->S |
| 101231 | - | G | C->T | 0 | 0 | 638 | 1 | 12 | 0 | 19 | 0 | 38.71% | CDS | *matR* | **2** | CCG->CTG | P->L |
| 101212 | - | G | C->T | 0 | 0 | 568 | 0 | 22 | 0 | 2 | 0 | 91.67% | CDS | *matR* | **3** | CTC->CTT | L->L |
| 100813 | - | G | C->T | 0 | 0 | 1224 | 0 | 16 | 0 | 25 | 0 | 39.02% | CDS | *matR* | **3** | TCC->TCT | S->S |
| 100657 | - | G | C->T | 0 | 0 | 1246 | 0 | 14 | 0 | 34 | 0 | 29.17% | CDS | *matR* | **3** | CTC->CTT | L->L |
| 100639 | - | G | C->T | 0 | 0 | 1222 | 0 | 26 | 0 | 23 | 0 | 53.06% | CDS | *matR* | **3** | CTC->CTT | L->L |
| 100444 | - | G | C->T | 0 | 0 | 1411 | 0 | 6 | 0 | 24 | 0 | 20.00% | CDS | *matR* | **3** | TAC->TAT | Y->Y |
| 100391 | - | G | C->T | 0 | 0 | 1168 | 0 | 33 | 0 | 9 | 0 | 78.57% | CDS | *matR* | **2** | CCG->CTG | P->L |
| 100292 | - | G | C->T | 0 | 0 | 608 | 2 | 15 | 0 | 2 | 0 | 88.24% | CDS | *matR* | **2** | CCC->CTC | P->L |
| 98594 | - | G | C->T | 0 | 0 | 873 | 0 | 66 | 0 | 19 | 0 | 77.65% | CDS | *matR* | **2** | ACC->ATC | T->I |
| 98466 | - | G | C->T | 0 | 0 | 648 | 0 | 68 | 0 | 29 | 0 | 70.10% | CDS | *matR* | **1** | CGG->TGG | R->W |
| 98439 | - | G | C->T | 1 | 0 | 537 | 0 | 50 | 0 | 42 | 0 | 54.35% | CDS | *matR* | **1** | CCG->TCG | P->S |
| 98438 | - | G | C->T | 0 | 0 | 539 | 0 | 60 | 0 | 30 | 0 | 66.67% | CDS | *matR* | **2** | CCG->CTG | P->L |
| 98421 | - | G | C->T | 0 | 0 | 530 | 0 | 67 | 0 | 19 | 0 | 77.91% | CDS | *matR* | **1** | CGC->TGC | R->C |
| 98360 | - | G | C->T | 0 | 0 | 630 | 0 | 32 | 0 | 5 | 0 | 86.49% | CDS | *matR* | **2** | TCC->TTC | S->F |
| 98354 | - | G | C->T | 0 | 0 | 662 | 0 | 31 | 0 | 5 | 0 | 86.11% | CDS | *matR* | **2** | CCT->CTT | P->L |
| 98320 | - | G | C->T | 0 | 0 | 769 | 0 | 13 | 0 | 25 | 0 | 34.21% | CDS | *matR* | **3** | ATC->ATT | I->I |
| 98315 | - | G | C->T | 1 | 0 | 788 | 1 | 28 | 0 | 15 | 0 | 65.12% | CDS | *matR* | **2** | CCG->CTG | P->L |
| 98182 | - | G | C->T | 0 | 1 | 372 | 0 | 4 | 0 | 15 | 0 | 21.05% | CDS | *matR* | **3** | TAC->TAT | Y->Y |
| 98161 | - | G | C->T | 0 | 1 | 231 | 0 | 8 | 0 | 7 | 0 | 53.33% | CDS | *matR* | **3** | TAC->TAT | Y->Y |
| 66152 | + | C | C->T | 0 | 1796 | 0 | 2 | 0 | 13 | 0 | 6 | 31.58% | CDS | *mttB* | **2** | CCG->CTG | P->L |
| 66185 | + | C | C->T | 0 | 1849 | 0 | 3 | 0 | 1 | 0 | 11 | 91.67% | CDS | *mttB* | **2** | TCG->TTG | S->L |
| 66193 | + | C | C->T | 0 | 1808 | 0 | 0 | 0 | 5 | 0 | 3 | 37.50% | CDS | *mttB* | **1** | CCC->TCC | P->S |
| 66470 | + | C | C->T | 0 | 2189 | 0 | 0 | 0 | 0 | 0 | 3 | 100.00% | CDS | *mttB* | **2** | CCG->CTG | P->L |
| 66785 | + | C | C->T | 0 | 1803 | 1 | 0 | 0 | 6 | 0 | 13 | 68.42% | CDS | *mttB* | **2** | TCG->TTG | S->L |
| 66797 | + | C | C->T | 0 | 1798 | 0 | 3 | 0 | 4 | 0 | 13 | 76.47% | CDS | *mttB* | **2** | CCG->CTG | P->L |
| 66818 | + | C | C->T | 0 | 1747 | 0 | 0 | 0 | 9 | 0 | 12 | 57.14% | CDS | *mttB* | **2** | TCT->TTT | S->F |
| 66827 | + | C | C->T | 0 | 1732 | 0 | 2 | 0 | 6 | 0 | 18 | 75.00% | CDS | *mttB* | **2** | TCG->TTG | S->L |
| 266952 | + | C | C->T | 0 | 1682 | 0 | 0 | 0 | 249 | 0 | 110 | 30.64% | CDS | *nad1* | 2 | ACG->ATG | T->M |
| 266961 | + | C | C->T | 0 | 1685 | 0 | 0 | 0 | 30 | 0 | 333 | 91.74% | CDS | *nad1* | **2** | GCT->GTT | A->V |
| 266966 | + | C | C->T | 0 | 1680 | 0 | 0 | 0 | 87 | 0 | 253 | 74.41% | CDS | *nad1* | **1** | CTA->TTA | L->L |
| 266979 | + | C | C->T | 0 | 1651 | 0 | 0 | 0 | 22 | 0 | 315 | 93.47% | CDS | *nad1* | 2 | CCC->CTC | P->L |
| 267018 | + | C | C->T | 0 | 1738 | 0 | 0 | 0 | 32 | 0 | 309 | 90.62% | CDS | *nad1* | 2 | TCA->TTA | S->L |
| 267047 | + | C | C->T | 0 | 1782 | 0 | 1 | 0 | 29 | 0 | 257 | 89.86% | CDS | *nad1* | 1 | CTC->TTC | L->F |
| 267084 | + | C | C->T | 1 | 1983 | 0 | 0 | 0 | 124 | 0 | 130 | 51.18% | CDS | *nad1* | **2** | TCG->TTG | S->L |
| 267117 | + | C | C->T | 1 | 2064 | 0 | 1 | 0 | 59 | 0 | 81 | 57.86% | CDS | *nad1* | 2 | TCG->TTG | S->L |
| 267123 | + | C | C->T | 0 | 2105 | 0 | 0 | 0 | 59 | 0 | 52 | 46.85% | CDS | *nad1* | 2 | TCA->TTA | S->L |
| 267143 | + | C | C->T | 0 | 2097 | 0 | 0 | 0 | 63 | 0 | 22 | 25.88% | CDS | *nad1* | 1 | CCA->TCA | P->S |
| 267165 | + | C | C->T | 0 | 2140 | 0 | 0 | 0 | 64 | 0 | 5 | 7.25% | CDS | *nad1* | 2 | TCT->TTT | S->F |
| 267171 | + | C | C->T | 0 | 2227 | 0 | 0 | 0 | 63 | 0 | 5 | 7.35% | CDS | *nad1* | 2 | TCT->TTT | S->F |
| 267195 | + | C | C->T | 0 | 2236 | 0 | 0 | 0 | 50 | 0 | 4 | 7.41% | CDS | *nad1* | 2 | TCT->TTT | S->F |
| 267215 | + | C | C->T | 0 | 2143 | 0 | 4 | 0 | 39 | 0 | 4 | 9.30% | CDS | *nad1* | 1 | CGG->TGG | R->W |
| 267231 | + | C | C->T | 0 | 2233 | 0 | 1 | 0 | 27 | 0 | 7 | 20.59% | CDS | *nad1* | 2 | TCT->TTT | S->F |
| 267249 | + | C | C->T | 2 | 2149 | 0 | 1 | 0 | 38 | 0 | 34 | 47.22% | CDS | *nad1* | 2 | TCG->TTG | S->L |
| 267258 | + | C | C->T | 0 | 2078 | 0 | 3 | 0 | 30 | 0 | 45 | 60.00% | CDS | *nad1* | 2 | TCG->TTG | S->L |
| 267275 | + | C | C->T | 0 | 1991 | 0 | 0 | 0 | 53 | 0 | 78 | 59.54% | CDS | *nad1* | 1 | CAT->TAT | H->Y |
| 267282 | + | C | C->T | 2 | 2011 | 0 | 1 | 0 | 36 | 0 | 107 | 74.83% | CDS | *nad1* | 2 | TCT->TTT | S->F |
| 143940 | - | G | C->T | 2 | 0 | 1782 | 0 | 31 | 0 | 16 | 0 | 65.96% | CDS | *nad1* | 2 | CCG->CTG | P->L |
| 143931 | - | G | C->T | 0 | 0 | 1803 | 1 | 21 | 0 | 18 | 0 | 53.85% | CDS | *nad1* | 2 | TCA->TTA | S->L |
| 143908 | - | G | C->T | 0 | 1 | 1825 | 0 | 11 | 0 | 16 | 0 | 40.74% | CDS | *nad1* | 1 | CCT->TCT | P->S |
| 143905 | - | G | C->T | 0 | 0 | 1857 | 0 | 10 | 0 | 18 | 0 | 35.71% | CDS | *nad1* | 1 | CAC->TAC | H->Y |
| 143844 | - | G | C->T | 0 | 0 | 2027 | 0 | 6 | 0 | 7 | 0 | 46.15% | CDS | *nad1* | 2 | TCG->TTG | S->L |
| 143789 | - | G | C->T | 1 | 0 | 1979 | 0 | 11 | 0 | 7 | 0 | 61.11% | CDS | *nad1* | **3** | CCC->CCT | P->P |
| 143775 | - | G | C->T | 0 | 0 | 1912 | 0 | 5 | 0 | 16 | 0 | 23.81% | CDS | *nad1* | 2 | TCC->TTC | S->F |
| 143767 | - | G | C->T | 1 | 0 | 1932 | 0 | 14 | 0 | 4 | 0 | 77.78% | CDS | *nad1* | 1 | CCT->TCT | P->S |
| 143737 | - | G | C->T | 0 | 0 | 2102 | 0 | 48 | 0 | 2 | 0 | 96.00% | CDS | *nad1* | 1 | CTT->TTT | L->F |
| 143709 | - | G | C->T | 0 | 0 | 2087 | 0 | 34 | 0 | 41 | 0 | 45.33% | CDS | *nad1* | 2 | TCA->TTA | S->L |
| 143691 | - | G | C->T | 0 | 0 | 2144 | 0 | 67 | 0 | 12 | 0 | 84.81% | CDS | *nad1* | 2 | GCA->GTA | A->V |
| 343882 | - | G | C->T | 1 | 0 | 1794 | 0 | 177 | 0 | 17 | 0 | 91.24% | CDS | *nad1* | 2 | CCA->CTA | P->L |
| 343874 | - | G | C->T | 0 | 0 | 1762 | 0 | 193 | 0 | 11 | 0 | 94.61% | CDS | *nad1* | 1 | CGG->TGG | R->W |
| 343819 | - | G | C->T | 0 | 0 | 1788 | 0 | 185 | 0 | 25 | 0 | 88.10% | CDS | *nad1* | 2 | TCT->TTT | S->F |
| 343781 | - | G | C->T | 1 | 0 | 1753 | 0 | 177 | 0 | 1 | 0 | 99.44% | CDS | *nad1* | 1 | CGG->TGG | R->W |
| 343741 | - | G | C->T | 0 | 0 | 1691 | 1 | 71 | 0 | 23 | 0 | 75.53% | CDS | *nad1* | 2 | TCA->TTA | S->L |
| 343731 | - | G | C->T | 0 | 0 | 1661 | 1 | 50 | 0 | 44 | 0 | 53.19% | CDS | *nad1* | **3** | CTC->CTT | L->L |
| 343727 | - | G | C->T | 0 | 0 | 1620 | 0 | 73 | 0 | 16 | 0 | 82.02% | CDS | *nad1* | 1 | CGG->TGG | R->W |
| 343714 | - | G | C->T | 0 | 0 | 1638 | 0 | 109 | 0 | 18 | 0 | 85.83% | CDS | *nad1* | 2 | TCG->TTG | S->L |
| 343697 | - | G | C->T | 1 | 0 | 1572 | 0 | 161 | 0 | 19 | 0 | 89.44% | CDS | *nad1* | 1 | CGG->TGG | R->W |
| 343680 | - | G | C->T | 0 | 0 | 1540 | 0 | 41 | 0 | 156 | 0 | 20.81% | CDS | *nad1* | **3** | TCC->TCT | S->S |
| 267474 | + | C | C->T | 0 | 1440 | 0 | 0 | 0 | 759 | 0 | 65 | 7.89% | intron | *nad1* |  | - |  |
| 267515 | + | C | C->T | 0 | 1300 | 0 | 0 | 0 | 742 | 0 | 41 | 5.24% | intron | *nad1* |  | - |  |
| 144006 | - | G | C->T | 0 | 0 | 1396 | 0 | 61 | 0 | 14 | 0 | 81.33% | intron | *nad1* |  | - | - |
| 143416 | - | G | C->T | 0 | 0 | 2216 | 1 | 43 | 0 | 87 | 0 | 33.08% | intron | *nad1* |  | - | - |
| 143459 | - | G | C->T | 0 | 0 | 2302 | 0 | 72 | 0 | 97 | 0 | 42.60% | intron | *nad1* |  | - | - |
| 143465 | - | G | C->T | 0 | 0 | 2342 | 0 | 79 | 0 | 83 | 0 | 48.77% | intron | *nad1* |  | - | - |
| 272380 | + | C | C->T | 0 | 808 | 0 | 0 | 0 | 84 | 0 | 18 | 17.65% | intron | *nad1* |  | - |  |
| 344053 | - | G | C->T | 0 | 0 | 1351 | 0 | 37 | 0 | 145 | 0 | 20.33% | intron | *nad1* |  | - | - |
| 344144 | - | G | C->T | 0 | 0 | 1069 | 0 | 32 | 0 | 97 | 0 | 24.81% | intron | *nad1* |  | - | - |
| 344174 | - | G | C->T | 0 | 0 | 844 | 0 | 30 | 0 | 79 | 0 | 27.52% | intron | *nad1* |  | - | - |
| 373761 | - | G | C->T | 0 | 0 | 2438 | 1 | 43 | 0 | 3 | 0 | 93.48% | CDS | *nad2* | **2** | CCA->CTA | P->L |
| 373716 | - | G | C->T | 0 | 0 | 2416 | 2 | 10 | 0 | 29 | 0 | 25.64% | CDS | *nad2* | **2** | TCG->TTG | S->L |
| 373713 | - | G | C->T | 0 | 0 | 2374 | 1 | 40 | 0 | 5 | 0 | 88.89% | CDS | *nad2* | **2** |  | p->l |
| 373712 | - | G | C->T | 0 | 0 | 2430 | 0 | 28 | 0 | 19 | 0 | 59.57% | CDS | *nad2* | **3** | CCC->CCT | P->P |
| 373523 | - | G | C->T | 0 | 0 | 2393 | 0 | 63 | 0 | 7 | 0 | 90.00% | CDS | *nad2* | **3** | CGC->CGT | R->R |
| 373443 | - | G | C->T | 1 | 0 | 1819 | 0 | 28 | 0 | 11 | 0 | 71.79% | CDS | *nad2* | **2** | TCT->TTT | S->L |
| 373428 | - | G | C->T | 0 | 0 | 1682 | 0 | 13 | 0 | 15 | 0 | 46.43% | CDS | *nad2* | **2** | TCA->TTA | S->L |
| 373422 | - | G | C->T | 0 | 0 | 1611 | 0 | 26 | 0 | 1 | 0 | 96.30% | CDS | *nad2* | **2** | CCA->CTA | P->L |
| 373417 | - | G | C->T | 0 | 0 | 1638 | 0 | 21 | 0 | 10 | 0 | 67.74% | CDS | *nad2* | **1** | CCT->TCT | P->S |
| 373362 | - | G | C->T | 0 | 0 | 1398 | 0 | 55 | 0 | 0 | 0 | 100.00% | CDS | *nad2* | **2** | TCA->TTA | S->L |
| 373336 | - | G | C->T | 0 | 0 | 1226 | 0 | 62 | 0 | 1 | 0 | 98.41% | CDS | *nad2* | **1** | CCT->TTT | P->F |
| 373335 | - | G | C->T | 0 | 0 | 1240 | 0 | 62 | 0 | 1 | 0 | 98.41% | CDS | *nad2* | **2** | CCT->TTT | ->F |
| 373255 | - | G | C->T | 0 | 0 | 1042 | 0 | 32 | 0 | 2 | 0 | 94.12% | CDS | *nad2* | **1** | CCC->TCC | P->S |
| 373239 | - | G | C->T | 0 | 0 | 977 | 0 | 21 | 0 | 8 | 0 | 72.41% | CDS | *nad2* | **2** | TCG->TTG | S->L |
| 8664 | + | C | C->T | 2 | 1167 | 0 | 0 | 0 | 38 | 0 | 41 | 51.90% | CDS | *nad2* | **2** | TCT->TTT | S->L |
| 8670 | + | C | C->T | 0 | 1195 | 0 | 0 | 0 | 54 | 0 | 29 | 34.94% | CDS | *nad2* | **2** | TCC->TTT | S->F |
| 8671 | + | C | C->T | 0 | 1194 | 0 | 0 | 0 | 70 | 0 | 14 | 16.67% | CDS | *nad2* | 3 | TCC->TTT | S->F |
| 10239 | + | C | C->T | 0 | 1560 | 0 | 0 | 0 | 57 | 0 | 3 | 5.00% | intron | *nad2* |  | - |  |
| 10294 | + | C | C->T | 0 | 1571 | 0 | 0 | 0 | 48 | 0 | 7 | 12.73% | intron | *nad2* |  | - |  |
| 11078 | + | C | C->T | 1 | 717 | 0 | 0 | 0 | 10 | 0 | 13 | 56.52% | CDS | *nad2* | **1** | CAT->TAT | H->Y |
| 11160 | + | C | C->T | 0 | 1359 | 0 | 0 | 0 | 6 | 0 | 5 | 45.45% | CDS | *nad2* | **2** | TCT->TTT | S->F |
| 11187 | + | C | C->T | 0 | 1593 | 0 | 0 | 0 | 1 | 0 | 5 | 83.33% | CDS | *nad2* | **2** | TCG->TTG | S->L |
| 11207 | + | C | C->T | 1 | 1739 | 0 | 0 | 0 | 2 | 0 | 3 | 60.00% | CDS | *nad2* | **1** | CGC->TGC | R->C |
| 11219 | + | C | C->T | 0 | 1780 | 0 | 0 | 0 | 1 | 0 | 4 | 80.00% | CDS | *nad2* | **1** | CCC->TCC | P->S |
| 11372 | + | C | C->T | 0 | 1993 | 0 | 0 | 0 | 5 | 0 | 23 | 82.14% | CDS | *nad2* | **1** | CAT->TAT | H->Y |
| 11379 | + | C | C->T | 0 | 1956 | 0 | 1 | 0 | 5 | 0 | 25 | 83.33% | CDS | *nad2* | **2** | TCA->TTA | S->L |
| 11397 | + | C | C->T | 0 | 2012 | 0 | 0 | 0 | 4 | 0 | 25 | 86.21% | CDS | *nad2* | **2** | TCC->TTC | S->F |
| 11409 | + | C | C->T | 0 | 2043 | 0 | 1 | 0 | 7 | 0 | 23 | 76.67% | CDS | *nad2* | **2** | CCG->CTG | P->L |
| 11592 | + | C | C->T | 0 | 2311 | 0 | 0 | 0 | 5 | 0 | 14 | 73.68% | CDS | *nad2* | **2** | CCA->CTA | P->L |
| 11627 | + | C | C->T | 1 | 2352 | 0 | 0 | 0 | 18 | 0 | 5 | 21.74% | CDS | *nad2* | **1** | CGT->TGT | R->C |
| 397415 | + | C | C->T | 0 | 1949 | 0 | 1 | 0 | 11 | 0 | 18 | 62.07% | CDS | *nad2* | **2** | GCG->GTG | A->V |
| 397487 | + | C | C->T | 0 | 1601 | 0 | 0 | 0 | 1 | 0 | 4 | 80.00% | CDS | *nad2* | **2** | CCA->CTA | P->L |
| 8150 | + | C | C->T | 1 | 963 | 0 | 0 | 0 | 25 | 0 | 18 | 41.86% | intron | *nad2* |  | - | - |
| 8160 | + | C | C->T | 0 | 990 | 0 | 1 | 0 | 23 | 0 | 14 | 37.84% | intron | *nad2* |  | - | - |
| 8067 | + | C | C->T | 0 | 716 | 0 | 0 | 0 | 23 | 0 | 3 | 11.54% | intron | *nad2* |  | - |  |
| 10232 | + | C | C->T | 0 | 1536 | 0 | 0 | 0 | 17 | 0 | 37 | 68.52% | intron | *nad2* |  | - | - |
| 10788 | + | C | C->T | 0 | 873 | 0 | 0 | 0 | 20 | 0 | 30 | 60.00% | intron | *nad2* |  | - | - |
| 10904 | + | C | C->T | 0 | 919 | 0 | 0 | 0 | 4 | 0 | 70 | 94.59% | intron | *nad2* |  | - | - |
| 10919 | + | C | C->T | 0 | 910 | 0 | 0 | 0 | 26 | 0 | 39 | 60.00% | intron | *nad2* |  | - | - |
| 11049 | + | C | C->T | 0 | 432 | 0 | 0 | 0 | 10 | 0 | 7 | 41.18% | intron | *nad2* |  | - | - |
| 397050 | + | C | C->T | 0 | 1349 | 0 | 0 | 0 | 105 | 0 | 68 | 39.31% | intron | *nad2* |  | - | - |
| 397074 | + | C | C->T | 0 | 1519 | 0 | 0 | 0 | 81 | 0 | 107 | 56.91% | intron | *nad2* |  | - | - |
| 397076 | + | C | C->T | 0 | 1542 | 0 | 0 | 0 | 13 | 0 | 176 | 93.12% | intron | *nad2* |  | - | - |
| 397121 | + | C | C->T | 0 | 1779 | 0 | 0 | 0 | 85 | 0 | 59 | 40.97% | intron | *nad2* |  | - | - |
| 397174 | + | C | C->T | 0 | 1902 | 0 | 1 | 0 | 10 | 0 | 119 | 92.25% | intron | *nad2* |  | - | - |
| 397209 | + | C | C->T | 0 | 1925 | 0 | 0 | 0 | 19 | 0 | 106 | 84.80% | intron | *nad2* |  | - | - |
| 397388 | + | C | C->T | 0 | 1942 | 0 | 0 | 0 | 10 | 0 | 50 | 83.33% | intron | *nad2* |  | - | - |
| 396984 | + | C | C->T | 0 | 1246 | 0 | 0 | 0 | 138 | 0 | 11 | 7.38% | intron | *nad2* |  | - |  |
| 397045 | + | C | C->T | 0 | 1369 | 0 | 1 | 0 | 160 | 0 | 12 | 6.98% | intron | *nad2* |  | - |  |
| 397089 | + | C | C->T | 1 | 1574 | 0 | 0 | 0 | 170 | 0 | 10 | 5.56% | intron | *nad2* |  | - |  |
| 397097 | + | C | C->T | 0 | 1601 | 0 | 0 | 0 | 146 | 0 | 14 | 8.75% | intron | *nad2* |  | - |  |
| 397181 | + | C | C->T | 0 | 1865 | 0 | 0 | 0 | 107 | 0 | 12 | 10.08% | intron | *nad2* |  | - |  |
| 397201 | + | C | C->T | 1 | 1902 | 0 | 0 | 0 | 101 | 0 | 22 | 17.89% | intron | *nad2* |  | - |  |
| 397202 | + | C | C->T | 0 | 1937 | 0 | 1 | 0 | 109 | 0 | 22 | 16.79% | intron | *nad2* |  | - |  |
| 397217 | + | C | C->T | 0 | 1900 | 0 | 0 | 0 | 122 | 0 | 11 | 8.27% | intron | *nad2* |  | - |  |
| 397222 | + | C | C->T | 0 | 1970 | 0 | 0 | 0 | 138 | 0 | 8 | 5.48% | intron | *nad2* |  | - |  |
| 397223 | + | C | C->T | 0 | 1964 | 0 | 0 | 0 | 128 | 0 | 9 | 6.57% | intron | *nad2* |  | - |  |
| 33549 | + | C | C->T | 0 | 1712 | 0 | 1 | 0 | 9 | 0 | 69 | 88.46% | CDS | *nad3* | **1** | CGT->TGT | R->C |
| 33558 | + | C | C->T | 0 | 1755 | 0 | 0 | 0 | 77 | 0 | 9 | 10.47% | CDS | *nad3* | 1 | CCA->TTA | P->L |
| 33559 | + | C | C->T | 0 | 1771 | 1 | 0 | 0 | 57 | 0 | 29 | 33.72% | CDS | *nad3* | **2** | CCA->TTA | P->L |
| 33578 | + | C | C->T | 0 | 1797 | 0 | 0 | 0 | 79 | 0 | 13 | 14.13% | CDS | *nad3* | 3 | GTC->GTT | V->V |
| 33611 | + | C | C->T | 0 | 1956 | 0 | 0 | 0 | 10 | 0 | 64 | 86.49% | CDS | *nad3* | **3** | TTC->TTT | F->F |
| 33651 | + | C | C->T | 0 | 1971 | 0 | 0 | 0 | 10 | 0 | 30 | 75.00% | CDS | *nad3* | **1** | CAC->TAC | H->Y |
| 33664 | + | C | C->T | 0 | 1941 | 0 | 0 | 0 | 16 | 0 | 18 | 52.94% | CDS | *nad3* | **2** | TCC->TTC | S->F |
| 33796 | + | C | C->T | 0 | 1858 | 0 | 0 | 0 | 9 | 0 | 3 | 25.00% | CDS | *nad3* | **2** | TCC->TTC | S->F |
| 33832 | + | C | C->T | 2 | 1883 | 0 | 0 | 0 | 4 | 0 | 13 | 76.47% | CDS | *nad3* | **2** | TCG->TTG | S->L |
| 33849 | + | C | C->T | 0 | 1948 | 0 | 1 | 0 | 5 | 0 | 18 | 78.26% | CDS | *nad3* | **1** | CAT->TAT | H->Y |
| 33876 | + | C | C->T | 0 | 1798 | 0 | 0 | 0 | 3 | 0 | 31 | 91.18% | CDS | *nad3* | **1** | CGG->TGG | R->W |
| 317761 | - | G | C->T | 0 | 0 | 1307 | 0 | 81 | 0 | 14 | 0 | 85.26% | CDS | *nad4* | 2 | ACG->ATG | T->M |
| 317747 | - | G | C->T | 0 | 0 | 1299 | 0 | 82 | 0 | 6 | 0 | 93.18% | CDS | *nad4* | 1 | CGT->TGT | R->C |
| 317734 | - | G | C->T | 2 | 0 | 1342 | 0 | 66 | 0 | 14 | 0 | 82.50% | CDS | *nad4* | 2 | TCC->TTC | S->F |
| 317714 | - | G | C->T | 0 | 0 | 1317 | 0 | 27 | 0 | 31 | 0 | 46.55% | CDS | *nad4* | 1 | CCG->TTG | P->L |
| 317713 | - | G | C->T | 2 | 0 | 1324 | 1 | 53 | 0 | 5 | 0 | 91.38% | CDS | *nad4* | 2 | CCG->TTG | P->L |
| 317626 | - | G | C->T | 0 | 0 | 1503 | 0 | 72 | 0 | 10 | 0 | 87.80% | CDS | *nad4* | 2 | CCC->CTT | P->L |
| 317625 | - | G | C->T | 1 | 0 | 1541 | 0 | 54 | 0 | 30 | 0 | 64.29% | CDS | *nad4* | 3 | CCC->CTT | P->L |
| 317607 | - | G | C->T | 0 | 0 | 1516 | 0 | 23 | 0 | 48 | 0 | 32.39% | CDS | *nad4* | 3 | TCC->TCT | S->S |
| 317597 | - | G | C->T | 1 | 0 | 1579 | 0 | 63 | 0 | 8 | 0 | 88.73% | CDS | *nad4* | 1 | CGG->TGG | R->W |
| 317577 | - | G | C->T | 0 | 0 | 1504 | 0 | 35 | 0 | 22 | 0 | 61.40% | CDS | *nad4* | 3 | TCC->TCT | S->S |
| 317534 | - | G | C->T | 0 | 1 | 1715 | 0 | 12 | 0 | 11 | 0 | 52.17% | CDS | *nad4* | 1 | CAT->TAT | H->Y |
| 317514 | - | G | C->T | 0 | 0 | 1804 | 0 | 14 | 0 | 3 | 0 | 82.35% | CDS | *nad4* | 3 | TAC->TAT | Y->Y |
| 317495 | - | G | C->T | 0 | 0 | 1789 | 0 | 12 | 0 | 2 | 0 | 85.71% | CDS | *nad4* | 1 | CCC->TCC | P->S |
| 317491 | - | G | C->T | 0 | 0 | 1851 | 0 | 11 | 0 | 3 | 0 | 78.57% | CDS | *nad4* | 2 | TCA->TTA | S->L |
| 317464 | - | G | C->T | 1 | 0 | 1968 | 0 | 14 | 0 | 4 | 0 | 77.78% | CDS | *nad4* | 2 | CCA->CTA | P->L |
| 317453 | - | G | C->T | 2 | 0 | 2097 | 0 | 13 | 0 | 4 | 0 | 76.47% | CDS | *nad4* | 1 | CGC->TGC | R->C |
| 317392 | - | G | C->T | 0 | 0 | 2216 | 0 | 8 | 0 | 7 | 0 | 53.33% | CDS | *nad4* | 2 | CCA->CTA | P->L |
| 317360 | - | G | C->T | 0 | 0 | 2201 | 0 | 6 | 0 | 5 | 0 | 54.55% | CDS | *nad4* | 1 | CGC->TGC | R->C |
| 317336 | - | G | C->T | 0 | 0 | 2224 | 0 | 10 | 0 | 11 | 0 | 47.62% | CDS | *nad4* | 1 | CAT->TAT | H->Y |
| 317327 | - | G | C->T | 1 | 0 | 2195 | 0 | 16 | 0 | 11 | 0 | 59.26% | CDS | *nad4* | 1 | CCC->TTC | P->F |
| 317326 | - | G | C->T | 2 | 0 | 2197 | 0 | 16 | 0 | 11 | 0 | 59.26% | CDS | *nad4* | 2 | CCC->TTC | P->F |
| 391371 | + | C | C->T | 0 | 2197 | 0 | 2 | 0 | 44 | 0 | 3 | 6.38% | CDS | *nad4* | 3 | CCC->CCT | P->P |
| 391408 | + | C | C->T | 0 | 2133 | 0 | 0 | 0 | 42 | 0 | 3 | 6.67% | CDS | *nad4* | 1 | CTC->TTC | L->F |
| 391433 | + | C | C->T | 1 | 2148 | 0 | 0 | 0 | 51 | 0 | 9 | 15.00% | CDS | *nad4* | 2 | TCG->TTG | S->L |
| 391457 | + | C | C->T | 0 | 2166 | 1 | 0 | 0 | 50 | 0 | 15 | 23.08% | CDS | *nad4* | 2 | TCC->TTC | S->F |
| 391540 | + | C | C->T | 1 | 1944 | 0 | 0 | 0 | 24 | 0 | 19 | 44.19% | CDS | *nad4* | 1 | CGG->TGG | R->W |
| 391589 | + | C | C->T | 0 | 1696 | 0 | 4 | 0 | 31 | 0 | 3 | 8.82% | CDS | *nad4* | 2 | TCG->TTG | S->L |
| 391604 | + | C | C->T | 0 | 1690 | 0 | 1 | 0 | 30 | 0 | 4 | 11.76% | CDS | *nad4* | 2 | TCA->TTA | S->L |
| 391666 | + | C | C->T | 0 | 1392 | 0 | 2 | 0 | 37 | 0 | 10 | 21.28% | CDS | *nad4* | 1 | CGC->TGC | R->C |
| 391670 | + | C | C->T | 1 | 1349 | 0 | 0 | 0 | 43 | 0 | 7 | 14.00% | CDS | *nad4* | 2 | CCC->CTC | P->L |
| 391697 | + | C | C->T | 0 | 1355 | 0 | 0 | 0 | 54 | 0 | 5 | 8.47% | CDS | *nad4* | 2 | GCG->GTG | A->V |
| 391745 | + | C | C->T | 0 | 1314 | 0 | 0 | 0 | 51 | 0 | 14 | 21.54% | CDS | *nad4* | 2 | CCT->CTT | P->L |
| 391762 | + | C | C->T | 0 | 1181 | 0 | 0 | 0 | 62 | 0 | 4 | 6.06% | CDS | *nad4* | 1 | CAC->TAC | H->Y |
| 391768 | + | C | C->T | 0 | 1277 | 0 | 0 | 0 | 63 | 0 | 4 | 5.97% | CDS | *nad4* | 1 | CCA->TCA | P->S |
| 391786 | + | C | C->T | 0 | 1250 | 0 | 0 | 0 | 47 | 0 | 15 | 24.19% | CDS | *nad4* | 1 | CCT->TCT | P->S |
| 297281 | - | G | C->T | 1 | 0 | 1007 | 0 | 61 | 0 | 68 | 0 | 47.29% | CDS | *nad4* | 2 | TCG->TTG | S->L |
| 297263 | - | G | C->T | 2 | 0 | 1103 | 0 | 43 | 0 | 72 | 0 | 37.39% | CDS | *nad4* | 2 | CCG->CTG | P->L |
| 297203 | - | G | C->T | 0 | 0 | 1347 | 0 | 17 | 0 | 60 | 0 | 22.08% | CDS | *nad4* | 2 | CCC->CTC | P->L |
| 297119 | - | G | C->T | 1 | 0 | 1379 | 0 | 32 | 0 | 53 | 0 | 37.65% | CDS | *nad4* | 2 | TCA->TTA | S->L |
| 297101 | - | G | C->T | 0 | 0 | 1480 | 0 | 23 | 0 | 76 | 0 | 23.23% | CDS | *nad4* | 2 | TCT->TTT | S->L |
| 297086 | - | G | C->T | 0 | 0 | 1508 | 0 | 29 | 0 | 84 | 0 | 25.66% | CDS | *nad4* | 2 | CCC->CTC | P->L |
| 297006 | - | G | C->T | 0 | 0 | 1566 | 0 | 29 | 0 | 81 | 0 | 26.36% | CDS | *nad4* | 1 | CCC->TCC | P->S |
| 296984 | - | G | C->T | 1 | 0 | 1599 | 1 | 60 | 0 | 63 | 0 | 48.78% | CDS | *nad4* | 2 | GCG->GTG | A->V |
| 296979 | - | G | C->T | 0 | 0 | 1615 | 0 | 27 | 0 | 85 | 0 | 24.11% | CDS | *nad4* | 1 | CTC->TTC | L->F |
| 296962 | - | G | C->T | 0 | 0 | 1630 | 0 | 58 | 0 | 40 | 0 | 59.18% | CDS | *nad4* | 3 | CCC->CCT | P->P |
| 296937 | - | G | C->T | 1 | 0 | 1655 | 0 | 63 | 0 | 47 | 0 | 57.27% | CDS | *nad4* | 1 | CCA->TTA | P->L |
| 296936 | - | G | C->T | 0 | 1 | 1674 | 0 | 66 | 0 | 45 | 0 | 59.46% | CDS | *nad4* | 2 | CCA->TTA | P->L |
| 296918 | - | G | C->T | 0 | 0 | 1740 | 1 | 23 | 0 | 90 | 0 | 20.35% | CDS | *nad4* | 2 | ACG->ATG | L->M |
| 296904 | - | G | C->T | 0 | 0 | 1637 | 0 | 41 | 0 | 74 | 0 | 35.65% | CDS | *nad4* | 1 | CTT->TTT | L->F |
| 363806 | + | C | C->T | 1 | 2098 | 0 | 3 | 0 | 9 | 0 | 6 | 40.00% | CDS | *nad4* | 2 | CCG->CTG | P->L |
| 363819 | + | C | C->T | 0 | 2004 | 0 | 0 | 0 | 10 | 0 | 4 | 28.57% | CDS | *nad4* | 3 | CAC->CAT | H->H |
| 316961 | - | G | C->T | 0 | 0 | 1160 | 0 | 45 | 0 | 30 | 0 | 60.00% | intron | *nad4* |  | - | - |
| 391011 | + | C | C->T | 0 | 2255 | 0 | 0 | 0 | 201 | 0 | 58 | 22.39% | intron | *nad4* |  | - | - |
| 363415 | + | C | C->T | 1 | 1093 | 0 | 0 | 0 | 41 | 0 | 6 | 12.77% | intron | *nad4* |  | - |  |
| 363417 | + | C | C->T | 0 | 1097 | 0 | 0 | 0 | 41 | 0 | 4 | 8.89% | intron | *nad4* |  | - |  |
| 363438 | + | C | C->T | 0 | 1081 | 0 | 0 | 0 | 38 | 0 | 3 | 7.32% | intron | *nad4* |  | - |  |
| 363622 | + | C | C->T | 0 | 1672 | 0 | 0 | 0 | 25 | 0 | 6 | 19.35% | intron | *nad4* |  | - |  |
| 363658 | + | C | C->T | 0 | 1798 | 0 | 1 | 0 | 0 | 0 | 31 | 100.00% | intron | *nad4* |  | - | - |
| 363764 | + | C | C->T | 1 | 2168 | 0 | 0 | 0 | 10 | 0 | 22 | 68.75% | intron | *nad4* |  | - | - |
| 316478 | - | G | C->T | 0 | 0 | 1082 | 0 | 7 | 0 | 16 | 0 | 30.43% | CDS | *nad4L* | **1** | CCT->TCT | P->S |
| 316465 | - | G | C->T | 0 | 0 | 1007 | 0 | 6 | 0 | 11 | 0 | 35.29% | CDS | *nad4L* | **2** | CCC->CTC | P->L |
| 316404 | - | G | C->T | 0 | 1 | 1036 | 0 | 5 | 0 | 8 | 0 | 38.46% | CDS | *nad4L* | **3** | TCC->TCT | S->S |
| 316390 | - | G | C->T | 0 | 0 | 1040 | 0 | 7 | 0 | 5 | 0 | 58.33% | CDS | *nad4L* | **2** | TCA->TTA | S->L |
| 316369 | - | G | C->T | 0 | 0 | 1096 | 0 | 3 | 0 | 10 | 0 | 23.08% | CDS | *nad4L* | **2** | CCT->CTT | P->L |
| 316327 | - | G | C->T | 0 | 0 | 1237 | 0 | 8 | 0 | 23 | 0 | 25.81% | CDS | *nad4L* | **2** | TCA->TTA | S->L |
| 316315 | - | G | C->T | 1 | 0 | 1285 | 0 | 14 | 0 | 20 | 0 | 41.18% | CDS | *nad4L* | **2** | TCG->TTG | S->L |
| 316287 | - | G | C->T | 3 | 0 | 1389 | 1 | 33 | 0 | 24 | 0 | 57.89% | CDS | *nad4L* | **3** | TCC->TCT | S->S |
| 316267 | - | G | C->T | 0 | 0 | 1497 | 0 | 50 | 0 | 31 | 0 | 61.73% | CDS | *nad4L* | **2** | CCG->CTG | P->L |
| 316225 | - | G | C->T | 0 | 0 | 1549 | 0 | 105 | 0 | 13 | 0 | 88.98% | CDS | *nad4L* | **2** | TCT->TTT | S->F |
| 316217 | - | G | C->T | 0 | 0 | 1564 | 0 | 112 | 0 | 10 | 0 | 91.80% | CDS | *nad4L* | **1** | CGC->TGC | R->C |
| 316205 | - | G | C->T | 0 | 0 | 1512 | 1 | 108 | 0 | 17 | 0 | 86.40% | CDS | *nad4L* | **1** | CGA->TGA | R->U |
| 216952 | - | G | C->T | 0 | 0 | 1064 | 0 | 5 | 0 | 9 | 0 | 35.71% | CDS | *nad5* | **2** | TCA->TTA | S->L |
| 216892 | - | G | C->T | 1 | 0 | 1295 | 0 | 4 | 0 | 9 | 0 | 30.77% | CDS | *nad5* | **2** | CCA->CTA | P->L |
| 216842 | - | G | C->T | 0 | 0 | 1304 | 0 | 4 | 0 | 10 | 0 | 28.57% | CDS | *nad5* | **1** | CCT->TCT | P->S |
| 216835 | - | G | C->T | 0 | 0 | 1220 | 0 | 5 | 0 | 9 | 0 | 35.71% | CDS | *nad5* | **2** | TCA->TTA | S->L |
| 216820 | - | G | C->T | 0 | 0 | 1328 | 0 | 7 | 0 | 10 | 0 | 41.18% | CDS | *nad5* | **2** | TCT->TTT | S->F |
| 216805 | - | G | C->T | 2 | 0 | 1322 | 0 | 3 | 0 | 12 | 0 | 20.00% | CDS | *nad5* | **2** | CCG->CTG | P->L |
| 216744 | - | G | C->T | 0 | 0 | 1104 | 1 | 8 | 0 | 7 | 0 | 53.33% | CDS | *nad5* | **3** | GCC->GCT | A->A |
| 216740 | - | G | C->T | 0 | 0 | 1089 | 0 | 7 | 0 | 8 | 0 | 46.67% | CDS | *nad5* | **1** | CGG->TGG | R->W |
| 215421 | - | G | C->T | 0 | 0 | 2272 | 0 | 16 | 0 | 21 | 0 | 43.24% | CDS | *nad5* | **3** | ACC->ACT | T->T |
| 215406 | - | G | C->T | 0 | 1 | 2318 | 1 | 9 | 0 | 35 | 0 | 20.45% | CDS | *nad5* | **3** | ATC->ATT | I->I |
| 215392 | - | G | C->T | 0 | 0 | 2363 | 0 | 48 | 0 | 8 | 0 | 85.71% | CDS | *nad5* | **2** | GCA->GTA | A->V |
| 215317 | - | G | C->T | 0 | 0 | 2416 | 0 | 51 | 0 | 1 | 0 | 98.08% | CDS | *nad5* | **2** | TCA->TTA | S->L |
| 215308 | - | G | C->T | 0 | 0 | 2397 | 0 | 37 | 0 | 6 | 0 | 86.05% | CDS | *nad5* | **2** | TCC->TTC | S->F |
| 215280 | - | G | C->T | 0 | 0 | 2384 | 1 | 25 | 0 | 6 | 0 | 80.65% | CDS | *nad5* | **3** | ACC->ACT | T->T |
| 231504 | + | C | C->T | 0 | 1052 | 0 | 0 | 0 | 196 | 0 | 13 | 6.22% | intron | *nad5* |  | - |  |
| 231561 | + | C | C->T | 0 | 1120 | 0 | 0 | 0 | 265 | 0 | 20 | 7.02% | intron | *nad5* |  | - |  |
| 433985 | - | G | C->T | 0 | 0 | 1879 | 0 | 87 | 0 | 41 | 0 | 67.97% | CDS | *nad5* | **2** | ACC->ATC | T->I |
| 433967 | - | G | C->T | 2 | 0 | 1980 | 0 | 113 | 0 | 46 | 0 | 71.07% | CDS | *nad5* | **2** | CCG->CTG | P->L |
| 433883 | - | G | C->T | 0 | 0 | 2430 | 0 | 125 | 0 | 44 | 0 | 73.96% | CDS | *nad5* | **2** | TCC->TTC | S->F |
| 433842 | - | G | C->T | 0 | 0 | 2413 | 0 | 103 | 0 | 65 | 0 | 61.31% | CDS | *nad5* | **1** | CCC->TCC | P->F |
| 433777 | - | G | C->T | 0 | 0 | 2291 | 0 | 44 | 0 | 114 | 0 | 27.85% | CDS | *nad5* | **3** | TTC->TTT | F->F |
| 433769 | - | G | C->T | 0 | 0 | 2228 | 1 | 91 | 0 | 62 | 0 | 59.48% | CDS | *nad5* | **2** | TCA->TTA | S->L |
| 433675 | - | G | C->T | 0 | 0 | 1373 | 0 | 12 | 0 | 30 | 0 | 28.57% | CDS | *nad5* | **3** | TTC->TTT | F->F |
| 432845 | - | G | C->T | 0 | 0 | 2037 | 0 | 13 | 0 | 39 | 0 | 25.00% | CDS | *nad5* | **2** | CCA->CTA | P->L |
| 432830 | - | G | C->T | 0 | 0 | 2044 | 0 | 13 | 0 | 43 | 0 | 23.21% | CDS | *nad5* | **2** | CCC->CTC | P->L |
| 432813 | - | G | C->T | 0 | 0 | 2052 | 1 | 33 | 0 | 47 | 0 | 41.25% | CDS | *nad5* | **1** | CGT->TGT | R->C |
| 432773 | - | G | C->T | 1 | 0 | 1959 | 0 | 99 | 0 | 55 | 0 | 64.29% | CDS | *nad5* | **2** | TCG->TTG | S->L |
| 432761 | - | G | C->T | 0 | 0 | 1985 | 0 | 136 | 0 | 38 | 0 | 78.16% | CDS | *nad5* | **2** | TCG->TTG | S->L |
| 432740 | - | G | C->T | 2 | 0 | 1914 | 0 | 135 | 0 | 52 | 0 | 72.19% | CDS | *nad5* | **2** | CCC->CTC | P->L |
| 215441 | - | G | C->T | 0 | 0 | 2179 | 1 | 9 | 0 | 13 | 0 | 40.91% | intron | *nad5* |  | - | - |
| 215445 | - | G | C->T | 0 | 0 | 2230 | 0 | 19 | 0 | 5 | 0 | 79.17% | intron | *nad5* |  | - | - |
| 215446 | - | G | C->T | 0 | 0 | 2203 | 1 | 17 | 0 | 6 | 0 | 73.91% | intron | *nad5* |  | - | - |
| 433095 | - | G | C->T | 0 | 0 | 2066 | 1 | 39 | 0 | 22 | 0 | 63.93% | intron | *nad5* |  | - | - |
| 433495 | - | G | C->T | 1 | 0 | 1625 | 0 | 50 | 0 | 32 | 0 | 60.98% | intron | *nad5* |  | - | - |
| 75857 | + | C | C->T | 0 | 2435 | 0 | 0 | 0 | 12 | 0 | 14 | 53.85% | CDS | *nad6* | **2** | ACG->ATG | T->M |
| 75874 | + | C | C->T | 0 | 2429 | 0 | 2 | 0 | 18 | 0 | 4 | 18.18% | CDS | *nad6* | 1 | CCG->TCG | P->S |
| 75881 | + | C | C->T | 2 | 2381 | 1 | 0 | 0 | 12 | 0 | 10 | 45.45% | CDS | *nad6* | **2** | CCT->CTT | P->L |
| 4203 | + | C | C->T | 0 | 1318 | 0 | 0 | 0 | 13 | 0 | 32 | 71.11% | CDS | *nad7* | **2** | TCC->TTC | S->F |
| 4204 | + | C | C->T | 0 | 1288 | 0 | 0 | 0 | 20 | 0 | 24 | 54.55% | CDS | *nad7* | **3** | TCC->TCT | S->S |
| 4209 | + | C | C->T | 0 | 1362 | 0 | 0 | 0 | 10 | 0 | 27 | 72.97% | CDS | *nad7* | **2** | TCG->TTG | S->L |
| 4215 | + | C | C->T | 0 | 1340 | 0 | 0 | 0 | 11 | 0 | 25 | 69.44% | CDS | *nad7* | **2** | TCT->TTT | S->F |
| 4248 | + | C | C->T | 0 | 1233 | 0 | 0 | 0 | 28 | 0 | 77 | 73.33% | CDS | *nad7* | **2** | CCA->CTA | P->L |
| 4254 | + | C | C->T | 0 | 1248 | 0 | 0 | 0 | 25 | 0 | 76 | 75.25% | CDS | *nad7* | **2** | TCA->TTA | S->L |
| 4260 | + | C | C->T | 0 | 1236 | 0 | 0 | 0 | 24 | 0 | 81 | 77.14% | CDS | *nad7* | **2** | TCG->TTG | S->L |
| 4308 | + | C | C->T | 0 | 1103 | 0 | 0 | 0 | 16 | 0 | 109 | 87.20% | CDS | *nad7* | **2** | TCA->TTA | S->L |
| 4367 | + | C | C->T | 0 | 1052 | 0 | 0 | 0 | 33 | 0 | 38 | 53.52% | CDS | *nad7* | **1** | CAT->TAT | H->Y |
| 4602 | + | C | C->T | 0 | 516 | 0 | 0 | 0 | 53 | 0 | 4 | 7.02% | intron | *nad7* |  | - |  |
| 5207 | + | C | C->T | 0 | 453 | 0 | 0 | 0 | 79 | 0 | 15 | 15.96% | intron | *nad7* |  | - |  |
| 5235 | + | C | C->T | 0 | 512 | 0 | 0 | 0 | 89 | 0 | 9 | 9.18% | intron | *nad7* |  | - |  |
| 5711 | + | C | C->T | 0 | 390 | 0 | 0 | 0 | 34 | 0 | 4 | 10.53% | intron | *nad7* |  | - |  |
| 6730 | + | C | C->T | 0 | 688 | 0 | 1 | 0 | 40 | 0 | 4 | 9.09% | intron | *nad7* |  | - |  |
| 6759 | + | C | C->T | 0 | 872 | 0 | 1 | 0 | 44 | 0 | 5 | 10.20% | intron | *nad7* |  | - |  |
| 6846 | + | C | C->T | 0 | 1270 | 1 | 0 | 0 | 3 | 0 | 27 | 90.00% | CDS | *nad7* | **1** | CAC->TAC | H->Y |
| 6876 | + | C | C->T | 1 | 1370 | 0 | 0 | 0 | 8 | 0 | 43 | 84.31% | CDS | *nad7* | **1** | CAT->TAT | H->Y |
| 6881 | + | C | C->T | 0 | 1426 | 0 | 0 | 0 | 22 | 0 | 31 | 58.49% | CDS | *nad7* | **3** | TCC->TCT | S->S |
| 6883 | + | C | C->T | 0 | 1420 | 0 | 1 | 0 | 8 | 0 | 41 | 83.67% | CDS | *nad7* | **2** | TCA->TTA | S->L |
| 6900 | + | C | C->T | 0 | 1421 | 0 | 0 | 0 | 5 | 0 | 73 | 93.59% | CDS | *nad7* | **1** | CTG->TTG | L->L |
| 6948 | + | C | C->T | 0 | 1545 | 0 | 0 | 0 | 29 | 0 | 168 | 85.28% | CDS | *nad7* | **1** | CGC->TGC | R->C |
| 6985 | + | C | C->T | 0 | 1676 | 1 | 0 | 0 | 8 | 0 | 236 | 96.72% | CDS | *nad7* | **2** | TCA->TTA | S->L |
| 7057 | + | C | C->T | 0 | 1956 | 0 | 0 | 0 | 45 | 0 | 261 | 85.29% | CDS | *nad7* | **2** | TCG->TTG | S->L |
| 7077 | + | C | C->T | 0 | 1942 | 0 | 0 | 0 | 17 | 0 | 310 | 94.80% | CDS | *nad7* | **1** | CCG->TCG | P->S |
| 7595 | + | C | C->T | 1 | 2428 | 0 | 0 | 0 | 184 | 0 | 37 | 16.74% | CDS | *nad7* | 3 | TCC->TCT | S->S |
| 7619 | + | C | C->T | 0 | 2334 | 0 | 3 | 0 | 150 | 0 | 26 | 14.77% | CDS | *nad7* | 3 | ACC->ACT | T->T |
| 7661 | + | C | C->T | 0 | 2294 | 0 | 0 | 0 | 98 | 0 | 28 | 22.22% | CDS | *nad7* | **3** | GTC->GTT | V->V |
| 7689 | + | C | C->T | 1 | 2254 | 0 | 0 | 0 | 5 | 0 | 64 | 92.75% | CDS | *nad7* | **1** | CGT->TGT | R->C |
| 7696 | + | C | C->T | 2 | 2230 | 0 | 0 | 0 | 4 | 0 | 54 | 93.10% | CDS | *nad7* | **2** | ACA->ATA | T->I |
| 7720 | + | C | C->T | 0 | 2235 | 0 | 1 | 0 | 4 | 0 | 23 | 85.19% | CDS | *nad7* | **2** | TCA->TTA | S->L |
| 7756 | + | C | C->T | 0 | 2168 | 0 | 0 | 0 | 1 | 0 | 5 | 83.33% | CDS | *nad7* | **2** | CCA->CTA | P->L |
| 7774 | + | C | C->T | 0 | 2060 | 0 | 0 | 0 | 1 | 0 | 6 | 85.71% | CDS | *nad7* | **2** | ACC->ATC | T->I |
| 7777 | + | C | C->T | 0 | 2028 | 0 | 0 | 0 | 1 | 0 | 6 | 85.71% | CDS | *nad7* | **2** | ACC->ATC | T->I |
| 5206 | + | C | C->T | 0 | 479 | 0 | 0 | 0 | 70 | 0 | 29 | 29.29% | intron | *nad7* |  | - | - |
| 5719 | + | C | C->T | 0 | 416 | 0 | 0 | 0 | 34 | 0 | 10 | 22.73% | intron | *nad7* |  | - | - |
| 5831 | + | C | C->T | 0 | 625 | 0 | 0 | 0 | 45 | 0 | 26 | 36.62% | intron | *nad7* |  | - | - |
| 207028 | + | C | C->T | 0 | 2026 | 0 | 2 | 0 | 7 | 0 | 17 | 70.83% | CDS | *nad9* | **2** | ACG->ATG | T->M |
| 207055 | + | C | C->T | 0 | 1985 | 0 | 0 | 0 | 6 | 0 | 7 | 53.85% | CDS | *nad9* | **2** | CCG->CTG | P->L |
| 207067 | + | C | C->T | 0 | 1986 | 1 | 0 | 0 | 6 | 0 | 8 | 57.14% | CDS | *nad9* | **2** | TCA->TTA | S->L |
| 207118 | + | C | C->T | 0 | 2021 | 0 | 0 | 0 | 4 | 0 | 10 | 71.43% | CDS | *nad9* | **2** | TCA->TTA | S->L |
| 207139 | + | C | C->T | 0 | 2048 | 0 | 0 | 0 | 4 | 0 | 9 | 69.23% | CDS | *nad9* | **2** | CCA->CTA | P->L |
| 207160 | + | C | C->T | 0 | 2108 | 0 | 1 | 0 | 3 | 0 | 7 | 70.00% | CDS | *nad9* | **2** | CCG->CTG | P->L |
| 207337 | + | C | C->T | 0 | 2264 | 1 | 0 | 0 | 12 | 0 | 6 | 33.33% | CDS | *nad9* | **2** | CCA->CTA | P->L |
| 207504 | + | C | C->T | 1 | 2404 | 0 | 0 | 0 | 13 | 0 | 24 | 64.86% | CDS | *nad9* | **1** | CCG->TCG | P->S |
| 207550 | + | C | C->T | 0 | 2370 | 0 | 1 | 0 | 16 | 0 | 40 | 71.43% | CDS | *nad9* | **2** | TCC->TTC | S->F |
| 207559 | + | C | C->T | 0 | 2369 | 1 | 0 | 0 | 15 | 0 | 37 | 71.15% | CDS | *nad9* | **2** | TCC->TTC | S->F |
| 74867 | + | C | C->T | 0 | 1683 | 0 | 5 | 0 | 76 | 0 | 5 | 6.17% | CDS | *rpl16* | 2 | CCG->CTG | P->L |
| 75046 | + | C | C->T | 6 | 2283 | 0 | 0 | 0 | 159 | 0 | 38 | 19.29% | CDS | *rpl16* | 1 | CCA->TCA | P->S |
| 75073 | + | C | C->T | 1 | 2402 | 0 | 0 | 0 | 168 | 0 | 11 | 6.15% | CDS | *rpl16* | 1 | CGG->TGG | R->W |
| 75118 | + | C | C->T | 0 | 2321 | 1 | 0 | 0 | 150 | 0 | 9 | 5.66% | CDS | *rpl16* | 1 | CTT->TTT | L->F |
| 75165 | + | C | C->T | 0 | 2313 | 0 | 0 | 0 | 104 | 0 | 19 | 15.45% | CDS | *rpl16* | 3 | ATC->ATT | I->I |
| 75214 | + | C | C->T | 0 | 2369 | 1 | 0 | 0 | 92 | 0 | 16 | 14.81% | CDS | *rpl16* | 1 | CGA->TGA | R->U |
| 75231 | + | C | C->T | 0 | 2292 | 0 | 1 | 0 | 74 | 0 | 39 | 34.51% | CDS | *rpl16* | **3** | ATC->ATT | I->I |
| 75246 | + | C | C->T | 0 | 2157 | 0 | 2 | 0 | 78 | 0 | 32 | 29.09% | CDS | *rpl16* | **3** | TTC->TTT | F->F |
| 75307 | + | C | C->T | 1 | 2353 | 0 | 1 | 0 | 101 | 0 | 12 | 10.62% | CDS | *rpl16* | 1 | CCT->TCT | P->S |
| 146602 | - | G | C->T | 0 | 0 | 2380 | 0 | 26 | 0 | 22 | 0 | 54.17% | CDS | *rpl5* | **2** | CCG->CTG | P->L |
| 146590 | - | G | C->T | 0 | 1 | 2434 | 0 | 19 | 0 | 27 | 0 | 41.30% | CDS | *rpl5* | **2** | CCG->CTG | P->L |
| 146494 | - | G | C->T | 0 | 0 | 2422 | 0 | 37 | 0 | 32 | 0 | 53.62% | CDS | *rpl5* | **2** | CCG->CTG | P->L |
| 146492 | - | G | C->T | 0 | 0 | 2361 | 0 | 33 | 0 | 34 | 0 | 49.25% | CDS | *rpl5* | **1** | CGC->TGC | R->C |
| 146350 | - | G | C->T | 2 | 0 | 2223 | 0 | 45 | 0 | 29 | 0 | 60.81% | CDS | *rpl5* | **2** | TCC->TTC | S->F |
| 146335 | - | G | C->T | 3 | 0 | 2157 | 0 | 58 | 0 | 21 | 0 | 73.42% | CDS | *rpl5* | **2** | TCG->TTG | S->L |
| 146185 | - | G | C->T | 2 | 0 | 1523 | 0 | 46 | 0 | 30 | 0 | 60.53% | CDS | *rpl5* | **2** | GCA->GTA | A->V |
| 146180 | - | G | C->T | 0 | 0 | 1420 | 0 | 42 | 0 | 25 | 0 | 62.69% | CDS | *rpl5* | **1** | CCG->TCG | P->S |
| 146175 | - | G | C->T | 1 | 0 | 1452 | 0 | 36 | 0 | 29 | 0 | 55.38% | CDS | *rpl5* | **3** | GCC->GCT | A->A |
| 146152 | - | G | C->T | 0 | 0 | 1428 | 0 | 56 | 0 | 20 | 0 | 73.68% | CDS | *rpl5* | **2** | CCA->CTA | P->L |
| 146149 | - | G | C->T | 0 | 0 | 1435 | 0 | 53 | 0 | 23 | 0 | 69.74% | CDS | *rpl5* | **2** | TCG->TTG | S->L |
| 146111 | - | G | C->T | 0 | 0 | 1238 | 0 | 17 | 0 | 45 | 0 | 27.42% | CDS | *rpl5* | **1** | CTC->TTC | L->F |
| 33967 | + | C | C->T | 0 | 1716 | 0 | 3 | 0 | 65 | 0 | 4 | 5.80% | CDS | *rps12* | 1 | CGT->TGT | R->C |
| 34042 | + | C | C->T | 0 | 1592 | 0 | 0 | 0 | 14 | 0 | 65 | 82.28% | CDS | *rps12* | **1** | CGC->TGC | R->C |
| 34045 | + | C | C->T | 0 | 1547 | 0 | 0 | 0 | 40 | 0 | 39 | 49.37% | CDS | *rps12* | **1** | CCG->TCG | P->S |
| 34046 | + | C | C->T | 1 | 1564 | 0 | 0 | 0 | 18 | 0 | 59 | 76.62% | CDS | *rps12* | **2** | CCG->CTG | P->L |
| 34088 | + | C | C->T | 1 | 1536 | 1 | 0 | 0 | 3 | 0 | 73 | 96.05% | CDS | *rps12* | **2** | CCA->CTA | P->L |
| 34162 | + | C | C->T | 0 | 1451 | 0 | 0 | 0 | 52 | 0 | 7 | 11.86% | CDS | *rps12* | 1 | CCA->TTA | P->L |
| 34163 | + | C | C->T | 0 | 1447 | 0 | 0 | 0 | 15 | 0 | 43 | 74.14% | CDS | *rps12* | **2** | CCA->TTA | P->L |
| 34211 | + | C | C->T | 0 | 1318 | 0 | 1 | 0 | 8 | 0 | 36 | 81.82% | CDS | *rps12* | **2** | CCG->CTG | P->L |
| 34226 | + | C | C->T | 1 | 1262 | 0 | 0 | 0 | 5 | 0 | 37 | 88.10% | CDS | *rps12* | **2** | TCC->TTC | S->F |
| 34227 | + | C | C->T | 0 | 1264 | 0 | 0 | 0 | 7 | 0 | 32 | 82.05% | CDS | *rps12* | **3** | TCC->TCT | S->S |
| 34231 | + | C | C->T | 0 | 1310 | 0 | 0 | 0 | 9 | 0 | 34 | 79.07% | CDS | *rps12* | **1** | CGT->TGT | R->C |
| 34253 | + | C | C->T | 0 | 1254 | 0 | 4 | 0 | 8 | 0 | 64 | 88.89% | CDS | *rps12* | **2** | TCG->TTG | S->L |
| 34308 | + | C | C->T | 0 | 1076 | 0 | 0 | 0 | 61 | 0 | 48 | 44.04% | CDS | *rps12* | **3** | CCC->CCT | P->P |
| 34313 | + | C | C->T | 0 | 1097 | 0 | 0 | 0 | 105 | 0 | 7 | 6.25% | CDS | *rps12* | 2 | TCG->TTG | S->L |
| 67593 | + | C | C->T | 1 | 1382 | 0 | 0 | 0 | 7 | 0 | 57 | 89.06% | CDS | *rps13* | **2** | CCA->CTA | P->L |
| 67633 | + | C | C->T | 0 | 1172 | 0 | 0 | 0 | 42 | 0 | 18 | 30.00% | CDS | *rps13* | **3** | GCC->GCT | A->A |
| 67635 | + | C | C->T | 0 | 1187 | 0 | 1 | 0 | 8 | 0 | 49 | 85.96% | CDS | *rps13* | **2** | CCA->CTA | P->L |
| 67673 | + | C | C->T | 0 | 1115 | 0 | 0 | 0 | 58 | 0 | 5 | 7.94% | CDS | *rps13* | 1 | CAG->TAG | Q->U |
| 67679 | + | C | C->T | 0 | 1060 | 0 | 3 | 0 | 9 | 0 | 55 | 85.94% | CDS | *rps13* | **1** | CGT->TGT | R->C |
| 67685 | + | C | C->T | 0 | 1072 | 1 | 0 | 0 | 59 | 0 | 10 | 14.49% | CDS | *rps13* | 1 | CGA->TGA | R->U |
| 67722 | + | C | C->T | 0 | 976 | 0 | 0 | 0 | 3 | 0 | 50 | 94.34% | CDS | *rps13* | **2** | TCG->TTG | S->L |
| 67738 | + | C | C->T | 0 | 977 | 0 | 0 | 0 | 44 | 0 | 9 | 16.98% | CDS | *rps13* | 3 | ATC->ATT | I->I |
| 67804 | + | C | C->T | 0 | 948 | 0 | 0 | 0 | 73 | 0 | 11 | 13.10% | CDS | *rps13* | 3 | GTC->GTT | V->V |
| 67812 | + | C | C->T | 4 | 920 | 0 | 3 | 0 | 80 | 0 | 5 | 5.88% | CDS | *rps13* | 2 | GCG->GTG | A->V |
| 67819 | + | C | C->T | 0 | 958 | 0 | 0 | 0 | 83 | 0 | 5 | 5.68% | CDS | *rps13* | 3 | ATC->ATT | I->I |
| 67832 | + | C | C->T | 0 | 958 | 0 | 0 | 0 | 4 | 0 | 97 | 96.04% | CDS | *rps13* | **1** | CCT->TCT | P->S |
| 67833 | + | C | C->T | 0 | 987 | 0 | 0 | 0 | 92 | 0 | 7 | 7.07% | CDS | *rps13* | 2 | CCT->CTT | P->L |
| 67841 | + | C | C->T | 0 | 957 | 0 | 0 | 0 | 3 | 0 | 99 | 97.06% | CDS | *rps13* | **1** | CGT->TGT | R->C |
| 67895 | + | C | C->T | 0 | 932 | 0 | 0 | 0 | 97 | 0 | 6 | 5.83% | CDS | *rps13* | 1 | CAT->TAT | H->Y |
| 67926 | + | C | C->T | 0 | 809 | 0 | 0 | 0 | 82 | 0 | 12 | 12.77% | CDS | *rps13* | 2 | TCG->TTG | S->L |
| 73087 | + | C | C->T | 0 | 1498 | 0 | 0 | 0 | 12 | 0 | 99 | 89.19% | CDS | *rps19* | **1** | CTC->TTC | L->F |
| 73156 | + | C | C->T | 0 | 1439 | 0 | 0 | 0 | 22 | 0 | 65 | 74.71% | CDS | *rps19* | **1** | CCT->TCT | P->S |
| 73159 | + | C | C->T | 0 | 1450 | 0 | 0 | 0 | 24 | 0 | 64 | 72.73% | CDS | *rps19* | **1** | CCC->TCC | P->S |
| 73187 | + | C | C->T | 0 | 1376 | 0 | 0 | 0 | 23 | 0 | 44 | 65.67% | CDS | *rps19* | **2** | TCC->TTC | S->F |
| 73213 | + | C | C->T | 0 | 1365 | 0 | 2 | 0 | 33 | 0 | 34 | 50.75% | CDS | *rps19* | **1** | CCC->TCC | P->S |
| 73214 | + | C | C->T | 0 | 1390 | 0 | 2 | 0 | 27 | 0 | 38 | 58.46% | CDS | *rps19* | **2** | CCC->CTC | P->L |
| 73230 | + | C | C->T | 0 | 1349 | 0 | 0 | 0 | 53 | 0 | 19 | 26.39% | CDS | *rps19* | **3** | ATC->ATT | I->I |
| 73256 | + | C | C->T | 0 | 1283 | 1 | 0 | 0 | 20 | 0 | 48 | 70.59% | CDS | *rps19* | **2** | TCC->TTC | S->F |
| 73257 | + | C | C->T | 0 | 1313 | 0 | 0 | 0 | 65 | 0 | 7 | 9.72% | CDS | *rps19* | 3 | TCC->TCT | S->S |
| 73362 | + | C | C->T | 0 | 1301 | 0 | 0 | 0 | 15 | 0 | 28 | 65.12% | CDS | *rps3* | **3** | GTC->GTT | V->V |
| 73411 | + | C | C->T | 0 | 1422 | 0 | 0 | 0 | 9 | 0 | 45 | 83.33% | CDS | *rps3* | **1** | CGG->TGG | R->W |
| 73429 | + | C | C->T | 1 | 1534 | 0 | 0 | 0 | 15 | 0 | 46 | 75.41% | CDS | *rps3* | **1** | CAT->TAT | H->Y |
| 73439 | + | C | C->T | 0 | 1451 | 0 | 1 | 0 | 14 | 0 | 51 | 78.46% | CDS | *rps3* | **2** | CCG->CTG | P->L |
| 73460 | + | C | C->T | 1 | 1556 | 1 | 0 | 0 | 15 | 0 | 49 | 76.56% | CDS | *rps3* | **2** | CCT->CTT | P->L |
| 73532 | + | C | C->T | 0 | 1596 | 0 | 0 | 0 | 15 | 0 | 37 | 71.15% | CDS | *rps3* | **2** | CCT->CTT | P->L |
| 73566 | + | C | C->T | 0 | 1511 | 0 | 0 | 0 | 41 | 0 | 9 | 18.00% | CDS | *rps3* | 3 | CTC->CTT | L->L |
| 73571 | + | C | C->T | 0 | 1441 | 0 | 1 | 0 | 44 | 0 | 3 | 6.38% | CDS | *rps3* | 2 | CCT->CTT | P->L |
| 73585 | + | C | C->T | 0 | 1462 | 0 | 0 | 0 | 45 | 0 | 6 | 11.76% | CDS | *rps3* | 1 | CAA->TAA | Q->U |
| 73977 | + | C | C->T | 0 | 1848 | 0 | 0 | 0 | 61 | 0 | 11 | 15.28% | CDS | *rps3* | 3 | TTC->TTT | F->F |
| 73986 | + | C | C->T | 0 | 1897 | 0 | 0 | 0 | 80 | 0 | 5 | 5.88% | CDS | *rps3* | 3 | CTC->CTT | L->L |
| 74042 | + | C | C->T | 0 | 1776 | 0 | 2 | 0 | 64 | 0 | 47 | 42.34% | CDS | *rps3* | **2** | CCG->CTG | P->L |
| 74067 | + | C | C->T | 0 | 1821 | 0 | 0 | 0 | 94 | 0 | 23 | 19.66% | CDS | *rps3* | 3 | ACC->ACT | T->T |
| 74103 | + | C | C->T | 1 | 1876 | 0 | 0 | 0 | 25 | 0 | 87 | 77.68% | CDS | *rps3* | **3** | GTC->GTT | V->V |
| 74114 | + | C | C->T | 0 | 1862 | 1 | 0 | 0 | 108 | 0 | 7 | 6.09% | CDS | *rps3* | 2 | TCC->TTC | S->F |
| 74117 | + | C | C->T | 0 | 1936 | 0 | 0 | 0 | 16 | 0 | 97 | 85.84% | CDS | *rps3* | **2** | TCT->TTT | S->F |
| 74157 | + | C | C->T | 0 | 2057 | 0 | 1 | 0 | 78 | 0 | 5 | 6.02% | CDS | *rps3* | 3 | TTC->TTT | F->F |
| 74183 | + | C | C->T | 0 | 2163 | 0 | 0 | 0 | 5 | 0 | 60 | 92.31% | CDS | *rps3* | **2** | TCC->TTT | S->F |
| 74184 | + | C | C->T | 0 | 2199 | 0 | 2 | 0 | 57 | 0 | 7 | 10.94% | CDS | *rps3* | 3 | TCC->TTT | S->F |
| 74597 | + | C | C->T | 0 | 2003 | 0 | 0 | 0 | 6 | 0 | 115 | 95.04% | CDS | *rps3* | **2** | CCG->CTG | P->L |
| 74662 | + | C | C->T | 0 | 1965 | 0 | 1 | 0 | 8 | 0 | 148 | 94.87% | CDS | *rps3* | **1** | CTG->TTG | L->L |
| 74669 | + | C | C->T | 0 | 1969 | 0 | 0 | 0 | 135 | 0 | 18 | 11.76% | CDS | *rps3* | 2 | TCT->TTT | S->F |
| 74693 | + | C | C->T | 1 | 1878 | 1 | 1 | 0 | 19 | 0 | 133 | 87.50% | CDS | *rps3* | **2** | TCC->TTT | S->F |
| 74694 | + | C | C->T | 0 | 1898 | 0 | 0 | 0 | 29 | 0 | 117 | 80.14% | CDS | *rps3* | **3** | TCC->TTT | S->F |
| 74738 | + | C | C->T | 2 | 1820 | 0 | 3 | 0 | 7 | 0 | 106 | 93.81% | CDS | *rps3* | **2** | CCA->CTA | P->L |
| 74760 | + | C | C->T | 1 | 1814 | 0 | 0 | 0 | 77 | 0 | 26 | 25.24% | CDS | *rps3* | **3** | TTC->TTT | F->F |
| 74812 | + | C | C->T | 0 | 1730 | 0 | 1 | 0 | 78 | 0 | 7 | 8.24% | CDS | *rps3* | **1** | CGG->TGG | R->W |
| 74821 | + | C | C->T | 0 | 1763 | 0 | 0 | 0 | 60 | 0 | 24 | 28.57% | CDS | *rps3* | **1** | CCA->TTA | P->L |
| 74822 | + | C | C->T | 0 | 1741 | 0 | 0 | 0 | 68 | 0 | 15 | 18.07% | CDS | *rps3* | **2** | CCA->TTA | P->L |
| 74866 | + | C | C->T | 0 | 1664 | 0 | 0 | 0 | 3 | 0 | 77 | 96.25% | CDS | *rps3* | **1** | CCG->TCG | P->S |
| 74900 | + | C | C->T | 0 | 1845 | 1 | 0 | 0 | 109 | 0 | 6 | 5.22% | CDS | *rps3* | 2 | ACT->ATT | T->I |
| 74941 | + | C | C->T | 0 | 1915 | 1 | 1 | 0 | 162 | 0 | 10 | 5.81% | CDS | *rps3* | 1 | CGC->TGC | R->C |
| 74973 | + | C | C->T | 1 | 2013 | 0 | 0 | 0 | 116 | 0 | 97 | 45.54% | CDS | *rps3* | **3** | GTC->GTT | V->V |
| 75000 | + | C | C->T | 0 | 2068 | 1 | 0 | 0 | 185 | 0 | 12 | 6.09% | CDS | *rps3* | 3 | GTC->GTT | V->V |
| 75013 | + | C | C->T | 2 | 2121 | 0 | 4 | 0 | 13 | 0 | 187 | 93.50% | CDS | *rps3* | **1** | CCA->TCA | P->S |
| 34535 | + | C | C->T | 2 | 1314 | 0 | 0 | 0 | 5 | 0 | 57 | 91.94% | CDS | *rps4* | **2** | TCT->TTT | S->F |
| 34543 | + | C | C->T | 1 | 1384 | 0 | 0 | 0 | 6 | 0 | 67 | 91.78% | CDS | *rps4* | **1** | CGT->TGT | R->C |
| 34556 | + | C | C->T | 1 | 1464 | 0 | 0 | 0 | 5 | 0 | 79 | 94.05% | CDS | *rps4* | **2** | CCG->CTG | P->L |
| 34646 | + | C | C->T | 0 | 1690 | 0 | 2 | 0 | 10 | 0 | 56 | 84.85% | CDS | *rps4* | **2** | CCG->CTG | P->L |
| 34677 | + | C | C->T | 0 | 1725 | 0 | 0 | 0 | 14 | 0 | 16 | 53.33% | CDS | *rps4* | **3** | CTC->CTT | L->L |
| 34682 | + | C | C->T | 0 | 1743 | 0 | 0 | 0 | 11 | 0 | 18 | 62.07% | CDS | *rps4* | **2** | TCA->TTA | S->L |
| 34711 | + | C | C->T | 0 | 1758 | 0 | 0 | 0 | 10 | 0 | 7 | 41.18% | CDS | *rps4* | **1** | CAT->TAT | H->Y |
| 34775 | + | C | C->T | 0 | 1898 | 0 | 0 | 0 | 7 | 0 | 24 | 77.42% | CDS | *rps4* | **2** | TCA->TTA | S->L |
| 34784 | + | C | C->T | 1 | 1955 | 0 | 0 | 0 | 7 | 0 | 26 | 78.79% | CDS | *rps4* | **2** | CCA->CTA | P->L |
| 34796 | + | C | C->T | 0 | 1947 | 0 | 0 | 0 | 5 | 0 | 28 | 84.85% | CDS | *rps4* | **2** | TCG->TTG | S->L |
| 34818 | + | C | C->T | 0 | 2077 | 0 | 0 | 0 | 44 | 0 | 4 | 8.33% | CDS | *rps4* | 3 | CTC->CTT | L->L |
| 34825 | + | C | C->T | 0 | 2079 | 0 | 0 | 0 | 4 | 0 | 50 | 92.59% | CDS | *rps4* | **1** | CGC->TGC | R->C |
| 34863 | + | C | C->T | 0 | 2082 | 0 | 0 | 0 | 48 | 0 | 8 | 14.29% | CDS | *rps4* | 3 | CAC->CAT | H->H |
| 34872 | + | C | C->T | 0 | 2072 | 0 | 0 | 0 | 52 | 0 | 3 | 5.45% | CDS | *rps4* | 3 | ATC->ATT | I->I |
| 34928 | + | C | C->T | 0 | 2126 | 0 | 0 | 0 | 8 | 0 | 51 | 86.44% | CDS | *rps4* | **2** | CCA->CTA | P->L |
| 34933 | + | C | C->T | 0 | 2090 | 0 | 0 | 0 | 7 | 0 | 43 | 86.00% | CDS | *rps4* | **1** | CCT->TCT | P->S |
| 34979 | + | C | C->T | 0 | 1909 | 0 | 0 | 0 | 5 | 0 | 41 | 89.13% | CDS | *rps4* | **2** | TCC->TTC | S->F |
| 34980 | + | C | C->T | 1 | 1901 | 0 | 0 | 0 | 9 | 0 | 39 | 81.25% | CDS | *rps4* | **3** | TCC->TCT | S->S |
| 34986 | + | C | C->T | 0 | 1872 | 0 | 1 | 0 | 18 | 0 | 26 | 59.09% | CDS | *rps4* | **3** | ATC->ATT | I->I |
| 35007 | + | C | C->T | 1 | 1830 | 0 | 0 | 0 | 43 | 0 | 14 | 24.56% | CDS | *rps4* | **3** | ATC->ATT | I->I |
| 35037 | + | C | C->T | 0 | 1695 | 0 | 0 | 0 | 25 | 0 | 30 | 54.55% | CDS | *rps4* | **3** | CCC->CCT | P->P |
| 35038 | + | C | C->T | 1 | 1766 | 0 | 0 | 0 | 47 | 0 | 9 | 16.07% | CDS | *rps4* | 1 | CGA->TGA | R->U |
| 35058 | + | C | C->T | 0 | 1707 | 0 | 0 | 0 | 46 | 0 | 28 | 37.84% | CDS | *rps4* | **3** | TTC->TTT | F->F |
| 35059 | + | C | C->T | 0 | 1695 | 1 | 0 | 0 | 62 | 0 | 14 | 18.42% | CDS | *rps4* | 1 | CGC->TGC | R->C |
| 35137 | + | C | C->T | 0 | 1900 | 0 | 0 | 0 | 95 | 0 | 6 | 5.94% | CDS | *rps4* | 1 | CAA->TAA | Q->U |
| 35211 | + | C | C->T | 0 | 1591 | 0 | 0 | 0 | 67 | 0 | 7 | 9.46% | CDS | *rps4* | 3 | GAC->GAT | D->D |
| 35228 | + | C | C->T | 0 | 1580 | 0 | 1 | 0 | 3 | 0 | 69 | 95.83% | CDS | *rps4* | **2** | TCG->TTG | S->L |
| 35238 | + | C | C->T | 0 | 1507 | 0 | 2 | 0 | 41 | 0 | 40 | 49.38% | CDS | *rps4* | **3** | TCC->TCT | S->S |
| 35260 | + | C | C->T | 0 | 1279 | 0 | 0 | 0 | 75 | 0 | 7 | 8.54% | CDS | *rps4* | 1 | CCC->TTC | P->F |
| 35261 | + | C | C->T | 1 | 1257 | 0 | 0 | 0 | 74 | 0 | 7 | 8.64% | CDS | *rps4* | 2 | CCC->TTC | P->F |
| 35298 | + | C | C->T | 0 | 1072 | 0 | 0 | 0 | 76 | 0 | 33 | 30.28% | CDS | *rps4* | **3** | CCC->CCT | P->P |
| 35319 | + | C | C->T | 0 | 931 | 0 | 0 | 0 | 10 | 0 | 115 | 92.00% | CDS | *rps4* | **3** | CGC->CGT | R->R |
| 35349 | + | C | C->T | 1 | 875 | 0 | 0 | 0 | 119 | 0 | 7 | 5.56% | CDS | *rps4* | 3 | TCC->TCT | S->S |
| 35363 | + | C | C->T | 0 | 836 | 0 | 0 | 0 | 99 | 0 | 8 | 7.48% | CDS | *rps4* | 2 | TCC->TTC | S->F |
| 35478 | + | C | C->T | 0 | 825 | 0 | 0 | 0 | 36 | 0 | 5 | 12.20% | CDS | *rps4* | 3 | GTC->GTT | V->V |
| 35482 | + | C | C->T | 0 | 846 | 0 | 0 | 0 | 2 | 0 | 38 | 95.00% | CDS | *rps4* | **1** | CAT->TAT | H->Y |
| 35492 | + | C | C->T | 0 | 797 | 0 | 3 | 0 | 1 | 0 | 32 | 96.97% | CDS | *rps4* | **2** | CCG->CTG | P->L |
| 35507 | + | C | C->T | 0 | 798 | 0 | 0 | 0 | 0 | 0 | 35 | 100.00% | CDS | *rps4* | **2** | TCT->TTT | S->F |
| 35558 | + | C | C->T | 0 | 740 | 0 | 0 | 0 | 0 | 0 | 38 | 100.00% | CDS | *rps4* | **2** | CCG->CTG | P->L |
| 35568 | + | C | C->T | 0 | 701 | 0 | 0 | 0 | 30 | 0 | 8 | 21.05% | CDS | *rps4* | **3** | CCC->CCT | P->P |
| 188350 | + | C | C->T | 0 | 1618 | 0 | 0 | 0 | 3 | 0 | 89 | 96.74% | CDS | *sdh4* | **3** | TTC->TTT | F->F |
| 188479 | + | C | C->T | 1 | 1354 | 0 | 0 | 0 | 92 | 0 | 53 | 36.55% | CDS | *sdh4* | **3** | GCC->GCT | A->A |
| 188508 | + | C | C->T | 0 | 1372 | 0 | 1 | 0 | 19 | 0 | 111 | 85.38% | CDS | *sdh4* | **2** | TCC->TTC | S->F |
| 188510 | + | C | C->T | 0 | 1369 | 0 | 0 | 0 | 43 | 0 | 74 | 63.25% | CDS | *sdh4* | **1** | CTA->TTA | L->L |
| 188523 | + | C | C->T | 1 | 1420 | 0 | 0 | 0 | 62 | 0 | 38 | 38.00% | CDS | *sdh4* | **2** | TCG->TTG | S->L |
| 188543 | + | C | C->T | 0 | 1392 | 0 | 0 | 0 | 73 | 0 | 10 | 12.05% | CDS | *sdh4* | 1 | CCC->TTT | P->F |
| 188544 | + | C | C->T | 0 | 1391 | 0 | 1 | 0 | 77 | 0 | 6 | 7.23% | CDS | *sdh4* | 2 | CCC->TTT | P->F |
| 188545 | + | C | C->T | 0 | 1401 | 0 | 0 | 0 | 51 | 0 | 28 | 35.44% | CDS | *sdh4* | **3** | CCC->TTT | P->F |
| 188556 | + | C | C->T | 0 | 1394 | 0 | 0 | 0 | 26 | 0 | 59 | 69.41% | CDS | *sdh4* | **2** | TCG->TTG | S->L |
| 188568 | + | C | C->T | 0 | 1412 | 0 | 0 | 0 | 6 | 0 | 89 | 93.68% | CDS | *sdh4* | **2** | TCA->TTA | S->L |
| 188612 | + | C | C->T | 0 | 1185 | 0 | 0 | 0 | 3 | 0 | 68 | 95.77% | CDS | *sdh4* | **1** | CAC->TAC | H->Y |
| 188674 | + | C | C->T | 1 | 837 | 0 | 0 | 0 | 29 | 0 | 3 | 9.38% | CDS | *sdh4* | 3 | ATC->ATT | I->I |
| 188689 | + | C | C->T | 0 | 692 | 0 | 0 | 0 | 1 | 0 | 9 | 90.00% | CDS | *sdh4* | **3** | GTC->GTT | V->V |
| 188714 | + | C | C->T | 0 | 560 | 0 | 0 | 0 | 1 | 0 | 3 | 75.00% | CDS | *sdh4* | **1** | CAC->TAC | H->Y |
| 407440 | + | T | T->C | 0 | 7 | 0 | 969 | 0 | 47 | 0 | 826 | 94.62% | rRNA | *rrn26* |  | - | - |
| 407692 | + | C | C->T | 0 | 2023 | 0 | 0 | 0 | 1141 | 0 | 3012 | 72.53% | rRNA | *rrn26* |  | - | - |
| 426419 | + | C | C->T | 0 | 1150 | 0 | 0 | 0 | 14 | 0 | 3 | 17.65% | tRNA | *trnY-GUA* |  | - |  |
| 3224 | + | C | C->T | 0 | 94 | 0 | 0 | 0 | 11 | 0 | 4 | 26.67% | - | - |  | - | - |
| 3969 | + | C | C->T | 0 | 1140 | 0 | 0 | 0 | 28 | 0 | 61 | 68.54% | - | - |  | - | - |
| 3987 | + | C | C->T | 0 | 1125 | 0 | 0 | 0 | 13 | 0 | 75 | 85.23% | - | - |  | - | - |
| 3996 | + | C | C->T | 0 | 1119 | 0 | 0 | 0 | 16 | 0 | 61 | 79.22% | - | - |  | - | - |
| 6840 | - | C | G->A | 0 | 1270 | 0 | 0 | 0 | 3 | 0 | 11 | 78.57% | - | - |  | - | - |
| 33480 | + | C | C->T | 0 | 1192 | 0 | 0 | 0 | 5 | 0 | 24 | 82.76% | - | - |  | - | - |
| 34474 | + | C | C->T | 0 | 1144 | 1 | 1 | 0 | 17 | 0 | 30 | 63.83% | - | - |  | - | - |
| 34493 | + | C | C->T | 0 | 1110 | 0 | 0 | 0 | 6 | 0 | 41 | 87.23% | - | - |  | - | - |
| 34500 | + | C | C->T | 0 | 1134 | 1 | 1 | 0 | 36 | 0 | 10 | 21.74% | - | - |  | - | - |
| 35851 | + | C | C->T | 0 | 1434 | 0 | 0 | 0 | 25 | 0 | 16 | 39.02% | - | - |  | - | - |
| 45654 | + | C | C->T | 0 | 822 | 0 | 0 | 0 | 21 | 0 | 7 | 25.00% | - | - |  | - | - |
| 46127 | + | C | C->T | 1 | 738 | 0 | 0 | 0 | 7 | 0 | 15 | 68.18% | - | - |  | - | - |
| 48420 | + | C | C->T | 2 | 1887 | 0 | 1 | 0 | 28 | 0 | 40 | 58.82% | - | - |  | - | - |
| 49164 | + | C | C->T | 0 | 605 | 0 | 0 | 0 | 10 | 0 | 44 | 81.48% | - | - |  | - | - |
| 49283 | + | C | C->T | 0 | 396 | 0 | 0 | 0 | 21 | 0 | 8 | 27.59% | - | - |  | - | - |
| 49350 | + | C | C->T | 0 | 194 | 0 | 0 | 0 | 8 | 0 | 13 | 61.90% | - | - |  | - | - |
| 49421 | + | C | C->T | 0 | 104 | 0 | 0 | 0 | 6 | 0 | 4 | 40.00% | - | - |  | - | - |
| 49563 | + | C | C->T | 0 | 517 | 0 | 0 | 0 | 11 | 0 | 43 | 79.63% | - | - |  | - | - |
| 49564 | + | C | C->T | 0 | 534 | 0 | 0 | 0 | 10 | 0 | 46 | 82.14% | - | - |  | - | - |
| 49739 | + | C | C->T | 0 | 1097 | 0 | 0 | 0 | 28 | 0 | 292 | 91.25% | - | - |  | - | - |
| 51307 | + | C | C->T | 1 | 1877 | 0 | 0 | 0 | 26 | 0 | 282 | 91.56% | - | - |  | - | - |
| 51308 | + | C | C->T | 0 | 1872 | 0 | 0 | 0 | 12 | 0 | 293 | 96.07% | - | - |  | - | - |
| 63340 | + | G | G->A | 1 | 0 | 575 | 0 | 3 | 0 | 0 | 0 | 100.00% | - | - |  | - | - |
| 66054 | + | C | C->T | 1 | 1636 | 0 | 0 | 0 | 6 | 0 | 24 | 80.00% | - | - |  | - | - |
| 66114 | + | C | C->T | 1 | 1777 | 0 | 0 | 0 | 14 | 0 | 5 | 26.32% | - | - |  | - | - |
| 66217 | - | C | G->A | 2 | 1909 | 0 | 0 | 0 | 0 | 0 | 5 | 100.00% | - | - |  | - | - |
| 66521 | - | C | G->A | 0 | 2080 | 0 | 0 | 0 | 0 | 0 | 3 | 100.00% | - | - |  | - | - |
| 67026 | + | C | C->T | 0 | 1191 | 0 | 0 | 0 | 11 | 0 | 21 | 65.63% | - | - |  | - | - |
| 67075 | + | C | C->T | 0 | 1182 | 2 | 0 | 0 | 0 | 0 | 30 | 100.00% | - | - |  | - | - |
| 67556 | + | C | C->T | 0 | 1370 | 0 | 0 | 0 | 34 | 0 | 13 | 27.66% | - | - |  | - | - |
| 67557 | + | C | C->T | 0 | 1419 | 0 | 0 | 0 | 13 | 0 | 31 | 70.45% | - | - |  | - | - |
| 68008 | + | C | C->T | 0 | 343 | 0 | 0 | 0 | 11 | 0 | 21 | 65.63% | - | - |  | - | - |
| 70482 | + | C | C->T | 0 | 1560 | 0 | 1 | 0 | 2 | 0 | 4 | 66.67% | - | - |  | - | - |
| 70636 | + | C | C->T | 0 | 1578 | 0 | 0 | 0 | 9 | 0 | 3 | 25.00% | - | - |  | - | - |
| 70965 | - | C | G->A | 0 | 1125 | 0 | 0 | 0 | 0 | 0 | 4 | 100.00% | - | - |  | - | - |
| 70967 | - | C | G->A | 0 | 1135 | 0 | 0 | 0 | 0 | 0 | 4 | 100.00% | - | - |  | - | - |
| 126529 | - | A | T->C | 1666 | 1 | 1 | 0 | 36 | 0 | 3 | 0 | 92.31% | - | - |  | - | - |
| 146086 | - | G | C->T | 0 | 0 | 1158 | 0 | 37 | 0 | 46 | 0 | 44.58% | - | - |  | - | - |
| 148835 | - | G | C->T | 1 | 0 | 1647 | 1 | 26 | 0 | 71 | 0 | 26.80% | - | - |  | - | - |
| 184866 | + | C | C->T | 0 | 1432 | 0 | 0 | 0 | 120 | 0 | 78 | 39.39% | - | - |  | - | - |
| 184889 | + | C | C->T | 0 | 1511 | 0 | 0 | 0 | 36 | 0 | 139 | 79.43% | - | - |  | - | - |
| 184941 | + | C | C->T | 1 | 1684 | 0 | 2 | 0 | 42 | 0 | 39 | 48.15% | - | - |  | - | - |
| 186502 | + | C | C->T | 0 | 1135 | 0 | 0 | 0 | 62 | 0 | 223 | 78.25% | - | - |  | - | - |
| 189958 | + | C | C->T | 1 | 531 | 0 | 1 | 0 | 26 | 0 | 57 | 68.67% | - | - |  | - | - |
| 192319 | + | C | C->T | 0 | 1580 | 0 | 0 | 0 | 31 | 0 | 21 | 40.38% | - | - |  | - | - |
| 202925 | + | C | C->T | 0 | 1161 | 0 | 0 | 0 | 5 | 0 | 32 | 86.49% | - | - |  | - | - |
| 204369 | + | C | C->T | 1 | 1724 | 0 | 0 | 0 | 8 | 0 | 3 | 27.27% | - | - |  | - | - |
| 204860 | + | C | C->T | 0 | 2129 | 0 | 0 | 0 | 9 | 0 | 3 | 25.00% | - | - |  | - | - |
| 206947 | + | C | C->T | 0 | 1761 | 0 | 0 | 0 | 27 | 0 | 7 | 20.59% | - | - |  | - | - |
| 207022 | + | C | C->T | 2 | 1952 | 0 | 0 | 0 | 5 | 0 | 18 | 78.26% | - | - |  | - | - |
| 207023 | + | C | C->T | 0 | 1982 | 0 | 0 | 0 | 11 | 0 | 13 | 54.17% | - | - |  | - | - |
| 211001 | - | G | C->T | 0 | 0 | 988 | 0 | 7 | 0 | 4 | 0 | 63.64% | - | - |  | - | - |
| 211014 | - | G | C->T | 0 | 0 | 1082 | 1 | 7 | 0 | 6 | 0 | 53.85% | - | - |  | - | - |
| 211026 | - | G | C->T | 0 | 0 | 1139 | 0 | 8 | 0 | 6 | 0 | 57.14% | - | - |  | - | - |
| 211102 | - | G | C->T | 0 | 0 | 1290 | 0 | 17 | 0 | 13 | 0 | 56.67% | - | - |  | - | - |
| 211103 | - | G | C->T | 0 | 0 | 1275 | 1 | 7 | 0 | 22 | 0 | 24.14% | - | - |  | - | - |
| 212127 | - | G | C->T | 0 | 0 | 1729 | 0 | 9 | 0 | 4 | 0 | 69.23% | - | - |  | - | - |
| 212130 | - | G | C->T | 0 | 0 | 1779 | 0 | 8 | 0 | 6 | 0 | 57.14% | - | - |  | - | - |
| 212190 | - | G | C->T | 0 | 0 | 1859 | 0 | 7 | 0 | 6 | 0 | 53.85% | - | - |  | - | - |
| 212195 | - | G | C->T | 0 | 0 | 1826 | 0 | 3 | 0 | 10 | 0 | 23.08% | - | - |  | - | - |
| 212219 | - | G | C->T | 0 | 1 | 1922 | 0 | 4 | 0 | 14 | 0 | 22.22% | - | - |  | - | - |
| 212228 | - | G | C->T | 0 | 0 | 1859 | 0 | 7 | 0 | 14 | 0 | 33.33% | - | - |  | - | - |
| 212260 | - | G | C->T | 0 | 0 | 1886 | 0 | 14 | 0 | 20 | 0 | 41.18% | - | - |  | - | - |
| 212295 | - | G | C->T | 0 | 0 | 1769 | 1 | 18 | 0 | 22 | 0 | 45.00% | - | - |  | - | - |
| 212362 | - | G | C->T | 0 | 0 | 1796 | 0 | 8 | 0 | 29 | 0 | 21.62% | - | - |  | - | - |
| 212433 | - | G | C->T | 0 | 0 | 1731 | 0 | 12 | 0 | 10 | 0 | 54.55% | - | - |  | - | - |
| 212439 | - | G | C->T | 0 | 0 | 1766 | 0 | 5 | 0 | 17 | 0 | 22.73% | - | - |  | - | - |
| 213313 | - | G | C->T | 0 | 0 | 187 | 0 | 4 | 0 | 4 | 0 | 50.00% | - | - |  | - | - |
| 225660 | - | G | C->T | 0 | 0 | 1064 | 0 | 77 | 0 | 48 | 0 | 61.60% | - | - |  | - | - |
| 228938 | - | G | C->T | 0 | 0 | 327 | 0 | 3 | 0 | 4 | 0 | 42.86% | - | - |  | - | - |
| 232244 | - | C | G->A | 0 | 1313 | 0 | 0 | 0 | 35 | 0 | 10 | 22.22% | - | - |  | - | - |
| 233644 | + | C | C->T | 0 | 796 | 0 | 0 | 0 | 22 | 0 | 28 | 56.00% | - | - |  | - | - |
| 233940 | + | C | C->T | 0 | 1733 | 1 | 0 | 0 | 34 | 0 | 51 | 60.00% | - | - |  | - | - |
| 234825 | + | C | C->T | 0 | 425 | 0 | 0 | 0 | 48 | 0 | 37 | 43.53% | - | - |  | - | - |
| 235475 | + | C | C->T | 0 | 224 | 0 | 0 | 0 | 50 | 0 | 16 | 24.24% | - | - |  | - | - |
| 236178 | + | C | C->T | 0 | 1108 | 0 | 0 | 0 | 155 | 0 | 44 | 22.11% | - | - |  | - | - |
| 236577 | + | C | C->T | 0 | 966 | 0 | 0 | 0 | 17 | 0 | 79 | 82.29% | - | - |  | - | - |
| 236594 | + | C | C->T | 0 | 985 | 0 | 0 | 0 | 8 | 0 | 85 | 91.40% | - | - |  | - | - |
| 238279 | - | C | G->A | 1 | 1627 | 0 | 0 | 0 | 0 | 0 | 5 | 100.00% | - | - |  | - | - |
| 238336 | - | C | G->A | 0 | 1506 | 0 | 0 | 0 | 0 | 0 | 4 | 100.00% | - | - |  | - | - |
| 246503 | - | C | G->A | 0 | 1802 | 0 | 0 | 0 | 15 | 0 | 7 | 31.82% | - | - |  | - | - |
| 246745 | + | C | C->T | 0 | 879 | 0 | 0 | 0 | 69 | 0 | 23 | 25.00% | - | - |  | - | - |
| 246865 | + | C | C->T | 0 | 392 | 0 | 0 | 0 | 24 | 0 | 31 | 56.36% | - | - |  | - | - |
| 268178 | + | C | C->T | 0 | 1397 | 0 | 0 | 0 | 182 | 0 | 63 | 25.71% | - | - |  | - | - |
| 268406 | + | C | C->T | 0 | 1832 | 0 | 0 | 0 | 38 | 0 | 549 | 93.53% | - | - |  | - | - |
| 268486 | + | C | C->T | 0 | 1726 | 0 | 0 | 0 | 340 | 0 | 309 | 47.61% | - | - |  | - | - |
| 268501 | + | C | C->T | 0 | 1650 | 0 | 0 | 0 | 494 | 0 | 136 | 21.59% | - | - |  | - | - |
| 268580 | + | C | C->T | 0 | 1461 | 0 | 0 | 0 | 538 | 0 | 140 | 20.65% | - | - |  | - | - |
| 293332 | - | G | C->T | 1 | 2 | 1859 | 0 | 98 | 0 | 10 | 0 | 90.74% | - | - |  | - | - |
| 296233 | - | G | C->T | 0 | 0 | 941 | 0 | 17 | 0 | 43 | 0 | 28.33% | - | - |  | - | - |
| 316517 | - | G | C->T | 0 | 0 | 1177 | 0 | 13 | 0 | 11 | 0 | 54.17% | - | - |  | - | - |
| 316536 | - | G | C->T | 0 | 0 | 1128 | 0 | 7 | 0 | 19 | 0 | 26.92% | - | - |  | - | - |
| 316553 | - | G | C->T | 0 | 0 | 1051 | 0 | 12 | 0 | 23 | 0 | 34.29% | - | - |  | - | - |
| 346185 | - | G | C->T | 0 | 0 | 2098 | 0 | 4 | 0 | 4 | 0 | 50.00% | - | - |  | - | - |
| 346480 | - | G | C->T | 0 | 0 | 1945 | 0 | 3 | 0 | 10 | 0 | 23.08% | - | - |  | - | - |
| 362451 | + | C | C->T | 0 | 513 | 0 | 0 | 0 | 17 | 0 | 10 | 37.04% | - | - |  | - | - |
| 363768 | - | C | G->A | 0 | 2165 | 1 | 0 | 0 | 2 | 0 | 6 | 75.00% | - | - |  | - | - |
| 374067 | - | G | C->T | 0 | 0 | 2089 | 0 | 51 | 0 | 47 | 0 | 52.04% | - | - |  | - | - |
| 374197 | - | G | C->T | 0 | 0 | 1491 | 0 | 61 | 0 | 132 | 0 | 31.61% | - | - |  | - | - |
| 374267 | - | G | C->T | 0 | 0 | 1364 | 0 | 111 | 0 | 19 | 0 | 85.38% | - | - |  | - | - |
| 374329 | - | G | C->T | 1 | 0 | 1209 | 1 | 54 | 0 | 33 | 0 | 62.07% | - | - |  | - | - |
| 375278 | - | G | C->T | 0 | 0 | 1333 | 0 | 20 | 0 | 28 | 0 | 41.67% | - | - |  | - | - |
| 375284 | - | G | C->T | 2 | 0 | 1321 | 0 | 18 | 0 | 29 | 0 | 38.30% | - | - |  | - | - |
| 375396 | - | G | C->T | 0 | 0 | 1637 | 0 | 7 | 0 | 24 | 0 | 22.58% | - | - |  | - | - |
| 376855 | - | G | C->T | 0 | 0 | 1304 | 0 | 6 | 0 | 2 | 0 | 75.00% | - | - |  | - | - |
| 376989 | - | G | C->T | 0 | 0 | 1463 | 0 | 3 | 0 | 6 | 0 | 33.33% | - | - |  | - | - |
| 377241 | - | G | C->T | 0 | 0 | 1118 | 0 | 8 | 0 | 6 | 0 | 57.14% | - | - |  | - | - |
| 394340 | + | C | C->T | 1 | 1366 | 0 | 1 | 0 | 163 | 0 | 64 | 28.19% | - | - |  | - | - |
| 397392 | - | C | G->A | 0 | 1965 | 0 | 0 | 0 | 2 | 0 | 12 | 85.71% | - | - |  | - | - |
| 397430 | - | C | G->A | 1 | 1902 | 0 | 0 | 0 | 2 | 0 | 3 | 60.00% | - | - |  | - | - |
| 3973 | + | C | C->T | 0 | 1115 | 0 | 0 | 0 | 73 | 0 | 17 | 0.188888889 | - | - |  | - |  |
| 4117 | + | C | C->T | 0 | 1302 | 0 | 1 | 0 | 82 | 0 | 5 | 0.057471264 | - | - |  | - |  |
| 4148 | + | C | C->T | 0 | 1348 | 0 | 0 | 0 | 60 | 0 | 6 | 0.090909091 | - | - |  | - |  |
| 6956 | - | C | G->A | 0 | 1488 | 0 | 1 | 0 | 48 | 0 | 5 | 0.094339623 | - | - |  | - |  |
| 6989 | - | C | G->A | 0 | 1667 | 0 | 0 | 0 | 55 | 0 | 9 | 0.140625 | - | - |  | - |  |
| 7947 | + | C | C->T | 0 | 1251 | 1 | 0 | 0 | 22 | 0 | 3 | 0.12 | - | - |  | - |  |
| 33481 | + | C | C->T | 0 | 1255 | 0 | 0 | 0 | 30 | 0 | 6 | 0.166666667 | - | - |  | - |  |
| 33587 | - | C | G->A | 0 | 1828 | 0 | 0 | 0 | 51 | 0 | 3 | 0.055555556 | - | - |  | - |  |
| 33588 | - | C | G->A | 1 | 1849 | 0 | 0 | 0 | 46 | 0 | 8 | 0.148148148 | - | - |  | - |  |
| 34341 | + | C | C->T | 0 | 992 | 0 | 0 | 0 | 118 | 0 | 11 | 0.085271318 | - | - |  | - |  |
| 34376 | + | C | C->T | 1 | 874 | 0 | 1 | 0 | 98 | 0 | 7 | 0.066666667 | - | - |  | - |  |
| 34382 | + | C | C->T | 0 | 865 | 0 | 0 | 0 | 79 | 0 | 14 | 0.150537634 | - | - |  | - |  |
| 34392 | + | C | C->T | 0 | 847 | 0 | 0 | 0 | 86 | 0 | 8 | 0.085106383 | - | - |  | - |  |
| 34397 | + | C | C->T | 0 | 860 | 0 | 0 | 0 | 84 | 0 | 6 | 0.066666667 | - | - |  | - |  |
| 34434 | + | C | C->T | 0 | 961 | 0 | 0 | 0 | 71 | 0 | 10 | 0.12345679 | - | - |  | - |  |
| 35820 | + | C | C->T | 0 | 1263 | 0 | 0 | 0 | 49 | 0 | 4 | 0.075471698 | - | - |  | - |  |
| 48481 | - | C | G->A | 0 | 1736 | 1 | 0 | 0 | 18 | 0 | 4 | 0.181818182 | - | - |  | - |  |
| 49106 | + | C | C->T | 0 | 575 | 0 | 1 | 0 | 26 | 0 | 5 | 0.161290323 | - | - |  | - |  |
| 49647 | + | C | C->T | 0 | 937 | 0 | 0 | 0 | 539 | 0 | 56 | 0.094117647 | - | - |  | - |  |
| 49659 | + | C | C->T | 0 | 1011 | 0 | 0 | 0 | 581 | 0 | 35 | 0.056818182 | - | - |  | - |  |
| 50816 | - | C | G->A | 0 | 2052 | 0 | 3 | 0 | 166 | 0 | 9 | 0.051428571 | - | - |  | - |  |
| 66906 | + | C | C->T | 0 | 1517 | 0 | 0 | 0 | 46 | 0 | 6 | 0.115384615 | - | - |  | - |  |
| 66922 | + | C | C->T | 0 | 1365 | 0 | 1 | 0 | 52 | 0 | 5 | 0.087719298 | - | - |  | - |  |
| 67091 | + | C | C->T | 1 | 1191 | 0 | 0 | 0 | 28 | 0 | 5 | 0.151515152 | - | - |  | - |  |
| 67497 | + | C | C->T | 0 | 1386 | 0 | 0 | 0 | 39 | 0 | 3 | 0.071428571 | - | - |  | - |  |
| 67513 | - | C | G->A | 0 | 1392 | 0 | 1 | 0 | 16 | 0 | 3 | 0.157894737 | - | - |  | - |  |
| 67862 | - | C | G->A | 0 | 999 | 0 | 0 | 0 | 17 | 0 | 3 | 0.15 | - | - |  | - |  |
| 67932 | + | C | C->T | 0 | 742 | 0 | 0 | 0 | 85 | 0 | 10 | 0.105263158 | - | - |  | - |  |
| 73080 | - | C | G->A | 0 | 1426 | 0 | 0 | 0 | 31 | 0 | 5 | 0.138888889 | - | - |  | - |  |
| 74131 | - | C | G->A | 0 | 1915 | 0 | 0 | 0 | 45 | 0 | 3 | 0.0625 | - | - |  | - |  |
| 74952 | - | C | G->A | 1 | 1989 | 0 | 0 | 0 | 87 | 0 | 6 | 0.064516129 | - | - |  | - |  |
| 75072 | - | C | G->A | 0 | 2351 | 0 | 4 | 0 | 58 | 0 | 5 | 0.079365079 | - | - |  | - |  |
| 75156 | - | C | G->A | 0 | 2241 | 0 | 0 | 0 | 29 | 0 | 4 | 0.121212121 | - | - |  | - |  |
| 126529 | - | A | T->C | 1666 | 1 | 1 | 0 | 36 | 0 | 3 | 0 | 0.076923077 | - | - |  | - |  |
| 183994 | + | C | C->T | 0 | 527 | 0 | 0 | 0 | 264 | 0 | 15 | 0.053763441 | - | - |  | - |  |
| 184883 | + | C | C->T | 0 | 1502 | 0 | 0 | 0 | 164 | 0 | 17 | 0.093922652 | - | - |  | - |  |
| 186395 | - | C | G->A | 0 | 1156 | 0 | 0 | 0 | 129 | 0 | 7 | 0.051470588 | - | - |  | - |  |
| 186451 | - | C | G->A | 0 | 1203 | 0 | 0 | 0 | 112 | 0 | 6 | 0.050847458 | - | - |  | - |  |
| 206124 | + | C | C->T | 0 | 373 | 0 | 0 | 0 | 13 | 0 | 3 | 0.1875 | - | - |  | - |  |
| 206952 | + | C | C->T | 0 | 1746 | 0 | 0 | 0 | 29 | 0 | 3 | 0.09375 | - | - |  | - |  |
| 232251 | + | C | C->T | 1 | 1327 | 0 | 1 | 0 | 169 | 0 | 10 | 0.055865922 | - | - |  | - |  |
| 232799 | + | C | C->T | 0 | 481 | 0 | 0 | 0 | 113 | 0 | 19 | 0.143939394 | - | - |  | - |  |
| 232880 | + | C | C->T | 0 | 236 | 0 | 0 | 0 | 57 | 0 | 3 | 0.05 | - | - |  | - |  |
| 233925 | + | C | C->T | 0 | 1711 | 0 | 0 | 0 | 72 | 0 | 4 | 0.052631579 | - | - |  | - |  |
| 234623 | - | C | G->A | 0 | 979 | 0 | 0 | 0 | 98 | 0 | 6 | 0.057692308 | - | - |  | - |  |
| 234650 | - | C | G->A | 0 | 889 | 0 | 0 | 0 | 41 | 0 | 3 | 0.068181818 | - | - |  | - |  |
| 234837 | + | C | C->T | 0 | 485 | 0 | 0 | 0 | 100 | 0 | 11 | 0.099099099 | - | - |  | - |  |
| 234881 | + | C | C->T | 0 | 575 | 0 | 0 | 0 | 158 | 0 | 15 | 0.086705202 | - | - |  | - |  |
| 236895 | - | C | G->A | 0 | 298 | 0 | 0 | 0 | 51 | 0 | 3 | 0.055555556 | - | - |  | - |  |
| 236952 | + | C | C->T | 0 | 315 | 0 | 0 | 0 | 93 | 0 | 16 | 0.146788991 | - | - |  | - |  |
| 246119 | - | C | G->A | 1 | 1560 | 0 | 0 | 0 | 24 | 0 | 3 | 0.111111111 | - | - |  | - |  |
| 246742 | + | C | C->T | 0 | 893 | 0 | 0 | 0 | 78 | 0 | 12 | 0.133333333 | - | - |  | - |  |
| 247648 | + | C | C->T | 0 | 380 | 0 | 0 | 0 | 67 | 0 | 4 | 0.056338028 | - | - |  | - |  |
| 247660 | + | C | C->T | 0 | 463 | 0 | 0 | 0 | 66 | 0 | 7 | 0.095890411 | - | - |  | - |  |
| 247680 | + | C | C->T | 0 | 577 | 0 | 0 | 0 | 72 | 0 | 12 | 0.142857143 | - | - |  | - |  |
| 267402 | - | C | G->A | 1 | 1532 | 0 | 0 | 0 | 106 | 0 | 6 | 0.053571429 | - | - |  | - |  |
| 267516 | - | C | G->A | 0 | 1296 | 0 | 4 | 0 | 193 | 0 | 12 | 0.058536585 | - | - |  | - |  |
| 272407 | - | C | G->A | 0 | 620 | 0 | 0 | 0 | 40 | 0 | 5 | 0.111111111 | - | - |  | - |  |
| 272532 | + | C | C->T | 0 | 619 | 1 | 3 | 0 | 65 | 0 | 6 | 0.084507042 | - | - |  | - |  |
| 362062 | + | C | C->T | 0 | 1088 | 1 | 0 | 0 | 27 | 0 | 4 | 0.129032258 | - | - |  | - |  |
| 362226 | + | C | C->T | 0 | 902 | 1 | 0 | 0 | 24 | 0 | 3 | 0.111111111 | - | - |  | - |  |
| 362231 | + | C | C->T | 0 | 879 | 0 | 0 | 0 | 24 | 0 | 5 | 0.172413793 | - | - |  | - |  |
| 394230 | + | C | C->T | 0 | 1002 | 0 | 0 | 0 | 99 | 0 | 17 | 0.146551724 | - | - |  | - |  |
| 394390 | + | C | C->T | 0 | 1326 | 0 | 0 | 0 | 164 | 0 | 13 | 0.073446328 | - | - |  | - |  |
| 396080 | + | C | C->T | 0 | 585 | 0 | 0 | 0 | 64 | 0 | 6 | 0.085714286 | - | - |  | - |  |
| 396528 | + | C | C->T | 0 | 277 | 0 | 0 | 0 | 82 | 0 | 5 | 0.057471264 | - | - |  | - |  |
| 396556 | + | C | C->T | 0 | 245 | 0 | 0 | 0 | 67 | 0 | 9 | 0.118421053 | - | - |  | - |  |
| 424549 | + | C | C->T | 0 | 849 | 0 | 0 | 0 | 182 | 0 | 10 | 0.052083333 | - | - |  | - |  |
| 424582 | - | C | G->A | 0 | 880 | 1 | 1 | 0 | 82 | 0 | 6 | 0.068181818 | - | - |  | - |  |
| 424991 | + | C | C->T | 0 | 2033 | 0 | 0 | 0 | 768 | 0 | 51 | 0.062271062 | - | - |  | - |  |
