# Supplementary material for: The complete mitochondrial genome of Taxus cuspidata (Taxaceae): eight protein-coding genes have transferred to the nuclear genome
Source: BMC Evol Biol. 2020 Jan 20;20:10. doi: 10.1186/s12862-020-1582-1 (PMC6971862; doi:10.1186/s12862-020-1582-1)
Supplement: Supplementary file 9 — Additional file 9: Figure S5. Examples of genes with striking divergence between observed and predicted (PREP with cutoff value = 0.2, PREPACT with filter threshold = 20%) RNA editing sites. The horizontal line represents gene length, and the vertical line indicates the position of RNA editing site. [file 12862_2020_1582_MOESM9_ESM.pdf]

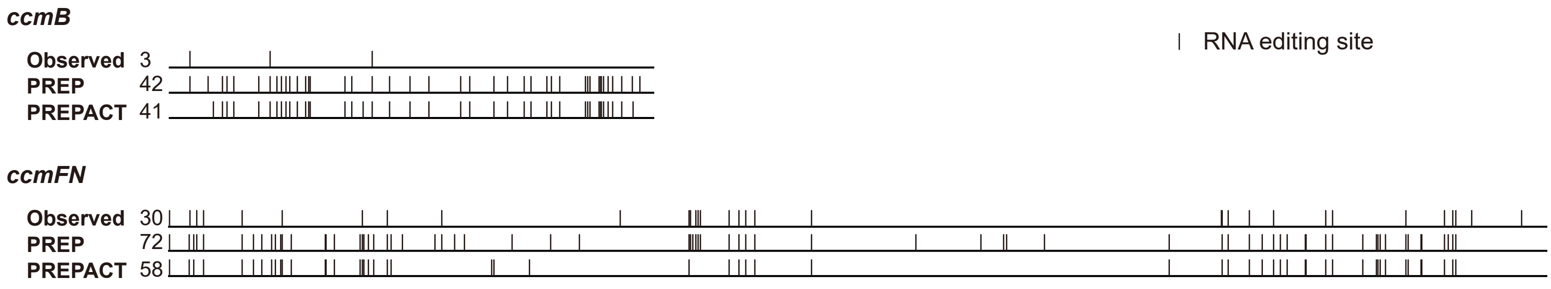

Additional file 9: Figure S5. Examples of genes with striking divergence between observed and predicted (PREP with cutoff value = 0.2, PREPACT with filter threshold = 20%) RNA editing sites. The horizontal line represents gene length, and the vertical line indicates the position of RNA editing site.
