# Supplementary material for: The complete mitochondrial genome of Taxus cuspidata (Taxaceae): eight protein-coding genes have transferred to the nuclear genome
Source: BMC Evol Biol. 2020 Jan 20;20:10. doi: 10.1186/s12862-020-1582-1 (PMC6971862; doi:10.1186/s12862-020-1582-1)
Supplement: Supplementary file 10 — Additional file 10: Table S5. Comparison of RNA editing sites among observed, predicted by PREP and PREPACT, respectively, in the Taxus mitogenome. [file 12862_2020_1582_MOESM10_ESM.docx]

**Additional file 10: Table S5.** Comparison of RNA editing sites among observed, predicted by PREP and PREPACT, respectively, in the *Taxus* mitogenome

| **Gene** | **Nt Pos** | **Codon**  **Phase** | **Edit Type** | **Codon**  **Change** | **Amino Acid**  **Change** | **Observed** | **PREP** | **PREPACT** |
| --- | --- | --- | --- | --- | --- | --- | --- | --- |
| *atp1* | 2 | 2 | C->T | ACG=>ATG | T=>M | + | - | - |
| *atp1* | 17 | 2 | C->T | CCA=>CTA | P=>L | + | + | + |
| *atp1* | 20 | 2 | C->T | TCG=>TTG | S=>L | + | + | + |
| *atp1* | 82 | 1 | C->T | CCA=>TCA | P=>S | + | + | + |
| *atp1* | 182 | 2 | C->T | CCT=>CTT | P=>L | + | + | + |
| *atp1* | 398 | 2 | C->T | GCG=>GTG | A=>V | - | + | + |
| *atp1* | 557 | 2 | C->T | TCA=>TTA | S=>L | + | + | + |
| *atp1* | 619 | 1 | C->T | CGC=>TGC | R=>C | + | + | + |
| *atp1* | 625 | 1 | C->T | CAT=>TAT | H=>Y | + | + | + |
| *atp1* | 677 | 2 | C->T | CCC=>CTC | P=>L | - | + | + |
| *atp1* | 679 | 1 | C->T | CCG=>TCG | P=>S | - | + | + |
| *atp1* | 727 | 1 | C->T | CCG=>TCG | P=>S | + | + | + |
| *atp1* | 752 | 2 | C->T | CCG=>CTG | P=>L | + | + | + |
| *atp1* | 763 | 1 | C->T | CCC=>TCC | P=>S | + | + | + |
| *atp1* | 788 | 2 | C->T | TCT=>TTT | S=>F | + | + | + |
| *atp1* | 830 | 2 | C->T | CCG=>CTG | P=>L | + | + | + |
| *atp1* | 869 | 2 | C->T | TCG=>TTG | S=>L | + | + | + |
| *atp1* | 872 | 2 | C->T | CCT=>CTT | P=>L | + | + | + |
| *atp1* | 916 | 1 | C->T | CAT=>TAT | H=>Y | + | + | + |
| *atp1* | 920 | 2 | C->T | TCG=>TTG | S=>L | + | + | + |
| *atp1* | 935 | 2 | C->T | TCG=>TTG | S=>L | + | + | + |
| *atp1* | 1048 | 1 | C->T | CCC=>TCC | P=>S | + | + | + |
| *atp1* | 1069 | 1 | C->T | CGT=>TGT | R=>C | + | + | + |
| *atp1* | 1073 | 2 | C->T | CCG=>CTG | P=>L | + | + | + |
| *atp1* | 1085 | 2 | C->T | CCC=>CTC | P=>L | + | + | + |
| *atp1* | 1126 | 1 | C->T | CCT=>TCT | P=>S | + | + | + |
| *atp1* | 1177 | 1 | C->T | CGC=>TGC | R=>C | + | - | - |
| *atp1* | 1187 | 2 | C->T | TCA=>TTA | S=>L | + | + | + |
| *atp1* | 1193 | 2 | C->T | CCC=>CTC | P=>L | + | + | + |
| *atp1* | 1199 | 2 | C->T | TCG=>TTG | S=>L | + | + | + |
| *atp1* | 1226 | 2 | C->T | TCT=>TTT | S=>F | + | + | + |
| *atp1* | 1235 | 2 | C->T | TCT=>TTT | S=>F | + | + | + |
| *atp1* | 1247 | 2 | C->T | CCT=>CTT | P=>L | + | + | + |
| *atp1* | 1256 | 2 | C->T | GCG=>GUG | A=>V | - | - | + |
| *atp1* | 1301 | 2 | C->T | CCG=>CTG | P=>L | + | + | + |
| *atp1* | 1318 | 1 | C->T | CCA=>TCA | P=>S | + | + | - |
| *atp1* | 1354 | 1 | C->T | CAT=>TAT | H=>Y | + | + | + |
| *atp1* | 1433 | 2 | C->T | CCA=>CTA | P=>L | + | - | - |
| *atp1* | 1454 | 2 | C->T | CCA=>CTA | P=>L | + | + | + |
| *atp1* | 1505 | 2 | C->T | CCA=>CTA | P=>L | + | + | + |
| *atp1* | 1514 | 2 | C->T | TCT=>TTT | S=>F | + | + | + |
| *atp1* | 1517 | 2 | C->T | TCA=>TTA | S=>L | + | + | + |
| *atp1* | 1532 | 2 | C->T | TCA=>TTA | S=>L | + | + | - |
| *atp4* | 8 | 2 | C->T | TCG=>TTG | S=>L | - | + | - |
| *atp4* | 29 | 2 | C->T | CCA=>CTA | P=>L | + | + | + |
| *atp4* | 44 | 2 | C->T | CCA=>CTA | P=>L | + | + | + |
| *atp4* | 77 | 2 | C->T | TCA=>TTA | S=>L | + | + | + |
| *atp4* | 95 | 2 | C->T | ACG=>ATG | T=>M | - | + | + |
| *atp4* | 109 | 1 | C->T | CGT=>TGT | R=>C | + | + | + |
| *atp4* | 113 | 2 | C->T | TCC=>TTC | S=>F | + | + | + |
| *atp4* | 158 | 2 | C->T | TCC=>TTT | S=>F | + | + | + |
| *atp4* | 159 | 3 | C->T | TCC=>TTT | S=>F | + | - | - |
| *atp4* | 164 | 2 | C->T | GCG=>GTG | A=>V | - | + | + |
| *atp4* | 214 | 1 | C->T | CTC=>TTC | L=>F | - | + | - |
| *atp4* | 236 | 2 | C->T | TCT=>TTT | S=>F | + | - | - |
| *atp4* | 238 | 1 | C->T | CCG=>UCG | P=>S | - | - | + |
| *atp4* | 239 | 2 | C->T | CCG=>CTG | P=>L | + | + | - |
| *atp4* | 314 | 2 | C->T | CCG=>CTG | P=>L | + | - | - |
| *atp4* | 323 | 2 | C->T | ACG=>ATG | T=>M | + | - | - |
| *atp4* | 368 | 2 | C->T | TCA=>TTA | S=>L | + | + | + |
| *atp4* | 380 | 2 | C->T | CCA=>CTA | P=>L | + | + | + |
| *atp4* | 392 | 2 | C->T | TCA=>TTA | S=>L | + | + | + |
| *atp4* | 401 | 2 | C->T | CCT=>CTT | P=>L | + | + | + |
| *atp4* | 404 | 2 | C->T | CCA=>CTA | P=>L | + | + | - |
| *atp4* | 464 | 2 | C->T | TCC=>TTC | S=>F | - | + | + |
| *atp4* | 487 | 1 | C->T | CCC=>TCC | P=>S | - | + | - |
| *atp4* | 509 | 2 | C->T | CCC=>CTT | P=>I | + | - | - |
| *atp4* | 510 | 3 | C->T | CCC=>CTT | P=>I | + | - | - |
| *atp4* | 518 | 2 | C->T | CCG=>CTG | P=>L | + | - | + |
| *atp4* | 580 | 1 | C->T | CCG=>TTG | P=>L | + | - | - |
| *atp4* | 581 | 2 | C->T | CCG=>TTG | P=>L | + | - | - |
| *atp4* | 617 | 2 | C->T | CCG=>CTG | P=>L | + | - | - |
| *atp4* | 647 | 2 | C->T | GCG=>GTG | A=>V | - | + | - |
| *atp4* | 650 | 2 | C->T | CCC=>CTC | P=>L | + | - | - |
| *atp4* | 668 | 2 | C->T | CCT=>CTT | P=>L | + | - | - |
| *atp4* | 694 | 1 | C->T | CGA=>TGA | R=>X | - | + | - |
| *atp6* | 2 | 2 | C->T | ACG=>ATG | T=>M | + | + | - |
| *atp6* | 65 | 2 | C->T | TCG=>TTG | S=>L | + | + | - |
| *atp6* | 88 | 1 | C->T | CCT=>TCT | P=>S | + | + | + |
| *atp6* | 104 | 2 | C->T | CCA=>CTA | P=>L | + | + | + |
| *atp6* | 110 | 2 | C->T | ACC=>ATC | T=>I | - | + | - |
| *atp6* | 137 | 2 | C->T | TCC=>TTC | S=>F | - | + | + |
| *atp6* | 140 | 2 | C->T | GCC=>GTC | A=>V | - | + | + |
| *atp6* | 199 | 1 | C->T | CAT=>TAT | H=>Y | + | + | + |
| *atp6* | 218 | 2 | C->T | CCG=>CTG | P=>L | + | + | + |
| *atp6* | 266 | 2 | C->T | TCC=>TTC | S=>F | - | + | + |
| *atp6* | 269 | 2 | C->T | TCC=>TTC | S=>F | - | + | + |
| *atp6* | 281 | 2 | C->T | CCG=>CTG | P=>L | - | + | + |
| *atp6* | 307 | 1 | C->T | CGT=>TGT | R=>C | + | + | + |
| *atp6* | 314 | 2 | C->T | CCC=>CTC | P=>L | + | + | + |
| *atp6* | 338 | 2 | C->T | TCC=>TTC | S=>F | + | + | + |
| *atp6* | 368 | 2 | C->T | TCG=>TTG | S=>L | + | + | + |
| *atp6* | 413 | 2 | C->T | TCT=>TTT | S=>F | + | + | + |
| *atp6* | 436 | 1 | C->T | CCC=>TTC | P=>F | - | + | - |
| *atp6* | 437 | 2 | C->T | CCC=>TTC | P=>F | - | + | + |
| *atp6* | 446 | 2 | C->T | TCA=>TTA | S=>L | - | + | + |
| *atp6* | 473 | 2 | C->T | TCA=>TTA | S=>L | - | + | + |
| *atp6* | 485 | 2 | C->T | TCA=>TTA | S=>L | - | + | + |
| *atp6* | 494 | 2 | C->T | CCT=>CTT | P=>L | - | + | + |
| *atp6* | 505 | 1 | C->T | CCT=>TCT | P=>S | - | + | + |
| *atp6* | 511 | 1 | C->T | CGC=>TGC | R=>C | - | + | + |
| *atp6* | 530 | 2 | C->T | TCA=>TTA | S=>L | + | + | + |
| *atp6* | 542 | 2 | C->T | TCA=>TTA | S=>L | + | + | + |
| *atp6* | 572 | 2 | C->T | TCA=>TTA | S=>L | + | + | + |
| *atp6* | 598 | 1 | C->T | CGG=>TGG | R=>W | + | + | + |
| *atp6* | 644 | 2 | C->T | CCC=>CTC | P=>L | + | + | + |
| *atp6* | 656 | 2 | C->T | TCA=>TTA | S=>L | + | - | - |
| *atp6* | 671 | 2 | C->T | CCG=>CTG | P=>L | + | + | + |
| *atp6* | 701 | 2 | C->T | TCA=>TTA | S=>L | + | + | + |
| *atp6* | 725 | 2 | C->T | TCA=>TTA | S=>L | - | + | + |
| *atp6* | 736 | 1 | C->T | CAC=>TAC | H=>Y | + | + | + |
| *atp6* | 740 | 2 | C->T | CCG=>CTG | P=>L | + | + | + |
| *atp6* | 763 | 1 | C->T | CAA=>TAA | Q=>U | + | + | - |
| *atp8* | 46 | 1 | C->T | CGG=>TGG | R=>W | + | + | + |
| *atp8* | 52 | 1 | C->T | CGC=>TGC | R=>C | + | + | + |
| *atp8* | 73 | 1 | C->T | CAT=>TAT | H=>Y | - | + | + |
| *atp8* | 79 | 1 | C->T | CCC=>UCC | P=>S | - | - | + |
| *atp8* | 80 | 2 | C->T | CCC=>CTC | P=>L | - | + | + |
| *atp8* | 101 | 2 | C->T | CCT=>CTT | P=>L | - | + | - |
| *atp8* | 107 | 2 | C->T | CCC=>CTC | P=>L | + | - | - |
| *atp8* | 119 | 2 | C->T | CCC=>CTC | P=>L | + | + | + |
| *atp8* | 125 | 2 | C->T | CCA=>CTA | P=>L | + | + | + |
| *atp8* | 142 | 1 | C->T | CCA=>TCA | P=>S | - | + | + |
| *atp8* | 160 | 1 | C->T | CGG=>TGG | R=>W | + | - | - |
| *atp8* | 194 | 2 | C->T | TCG=>TTG | S=>L | + | + | + |
| *atp8* | 206 | 2 | C->T | TCT=>TTT | S=>F | + | + | + |
| *atp8* | 218 | 2 | C->T | GCA=>GTA | A=>V | - | + | + |
| *atp8* | 301 | 1 | C->T | CCA=>TCA | P=>S | + | - | - |
| *atp8* | 302 | 2 | C->T | CCA=>CTA | P=>L | + | - | - |
| *atp8* | 307 | 1 | C->T | CGT=>TGT | R=>C | - | + | + |
| *atp8* | 364 | 1 | C->T | CCG=>TCG | P=>S | - | + | + |
| *atp8* | 388 | 1 | C->T | CCC=>TCC | P=>S | - | + | + |
| *atp8* | 401 | 2 | C->T | TCT=>TTT | S=>F | + | - | - |
| *atp8* | 442 | 1 | C->T | CAT=>TAT | H=>Y | + | - | - |
| *atp9* | 20 | 2 | C->T | TCA=>TTA | S=>L | - | + | + |
| *atp9* | 50 | 2 | C->T | TCA=>TTA | S=>L | - | + | + |
| *atp9* | 65 | 2 | C->T | GCC=>GTC | A=>V | - | + | + |
| *atp9* | 83 | 2 | C->T | TCC=>TTC | S=>F | - | + | + |
| *atp9* | 88 | 1 | C->T | CCC=>TCC | P=>S | - | + | + |
| *atp9* | 92 | 2 | C->T | TCG=>TTG | S=>L | - | + | + |
| *atp9* | 100 | 1 | C->T | CCC=>TCC | P=>S | - | + | + |
| *atp9* | 134 | 2 | C->T | TCA=>TTA | S=>L | - | + | + |
| *atp9* | 142 | 1 | C->T | CAC=>TAC | H=>Y | - | + | + |
| *atp9* | 152 | 2 | C->T | TCG=>TTG | S=>L | - | + | + |
| *atp9* | 164 | 2 | C->T | CCA=>CTA | P=>L | - | + | + |
| *atp9* | 191 | 2 | C->T | TCG=>TTG | S=>L | - | + | + |
| *atp9* | 212 | 2 | C->T | CCA=>CTA | P=>L | - | + | + |
| *atp9* | 215 | 2 | C->T | TCC=>TTC | S=>F | - | + | + |
| *atp9* | 223 | 1 | C->T | CGA=>TGA | R=>X | - | + | + |
| *ccmB* | 28 | 1 | C->T | CAT=>TAT | H=>Y | + | + | - |
| *ccmB* | 52 | 1 | C->T | CCC=>TCC | P=>S | - | + | - |
| *ccmB* | 59 | 2 | C->T | CCA=>CUA | P=>L | - | - | + |
| *ccmB* | 71 | 2 | C->T | TCT=>TTT | S=>F | - | + | + |
| *ccmB* | 77 | 2 | C->T | CCA=>CTA | P=>L | - | + | + |
| *ccmB* | 86 | 2 | C->T | TCG=>TTG | S=>L | - | + | + |
| *ccmB* | 119 | 2 | C->T | TCT=>TTT | S=>F | - | + | + |
| *ccmB* | 134 | 2 | C->T | TCA=>TTA | S=>L | + | + | + |
| *ccmB* | 143 | 2 | C->T | TCC=>TTC | S=>F | - | + | + |
| *ccmB* | 149 | 2 | C->T | TCA=>TTA | S=>L | - | + | + |
| *ccmB* | 155 | 2 | C->T | CCG=>CTG | P=>L | - | + | + |
| *ccmB* | 160 | 1 | C->T | CGG=>TGG | R=>W | - | + | + |
| *ccmB* | 170 | 2 | C->T | CCG=>CTG | P=>L | - | + | + |
| *ccmB* | 181 | 1 | C->T | CTT=>TTT | L=>F | - | + | + |
| *ccmB* | 185 | 2 | C->T | CCC=>CTC | P=>L | - | + | + |
| *ccmB* | 187 | 1 | C->T | CCC=>TCC | P=>S | - | + | + |
| *ccmB* | 233 | 2 | C->T | TCG=>TTG | S=>L | - | + | + |
| *ccmB* | 242 | 2 | C->T | TCA=>TTA | S=>L | - | + | + |
| *ccmB* | 257 | 2 | C->T | UCG=>UUG | S=>L | - | - | + |
| *ccmB* | 269 | 2 | C->T | CCA=>CTA | P=>L | + | + | + |
| *ccmB* | 292 | 1 | C->T | CGG=>TGG | R=>W | - | + | + |
| *ccmB* | 319 | 1 | C->T | CGT=>TGT | R=>C | - | + | + |
| *ccmB* | 344 | 2 | C->T | CCG=>CTG | P=>L | - | + | + |
| *ccmB* | 386 | 2 | C->T | CCA=>CTA | P=>L | - | + | + |
| *ccmB* | 398 | 2 | C->T | CCG=>CTG | P=>L | - | + | + |
| *ccmB* | 430 | 1 | C->T | CGT=>TGT | R=>C | - | + | + |
| *ccmB* | 448 | 1 | C->T | CCC=>TCC | P=>S | - | + | + |
| *ccmB* | 470 | 2 | C->T | TCG=>TTG | S=>L | - | + | + |
| *ccmB* | 479 | 2 | C->T | TCA=>TTA | S=>L | - | + | + |
| *ccmB* | 500 | 2 | C->T | TCG=>TTG | S=>L | - | + | + |
| *ccmB* | 506 | 2 | C->T | CCA=>CTA | P=>L | - | + | + |
| *ccmB* | 517 | 1 | C->T | CGT=>TGT | R=>C | - | + | + |
| *ccmB* | 551 | 2 | C->T | CCT=>CTT | P=>L | - | + | + |
| *ccmB* | 554 | 2 | C->T | TCA=>TTA | S=>L | - | + | + |
| *ccmB* | 557 | 2 | C->T | TCG=>TTG | S=>L | - | + | + |
| *ccmB* | 569 | 2 | C->T | TCC=>TTC | S=>F | - | + | + |
| *ccmB* | 571 | 1 | C->T | CCT=>TTT | P=>F | - | + | + |
| *ccmB* | 572 | 2 | C->T | CCT=>TTT | P=>F | - | + | + |
| *ccmB* | 575 | 2 | C->T | CCG=>CTG | P=>L | - | + | + |
| *ccmB* | 581 | 2 | C->T | GCA=>GTA | A=>V | - | + | + |
| *ccmB* | 587 | 2 | C->T | CCC=>CTC | P=>L | - | + | + |
| *ccmB* | 599 | 2 | C->T | TCG=>TTG | S=>L | - | + | + |
| *ccmB* | 613 | 1 | C->T | CCA=>UCA | P=>S | - | - | + |
| *ccmB* | 614 | 2 | C->T | CCA=>CTA | P=>L | - | + | - |
| *ccmB* | 623 | 2 | C->T | TCA=>TTA | S=>L | - | + | - |
| *ccmC* | 2 | 2 | C->T | ACG=>ATG | T=>M | + | + | + |
| *ccmC* | 76 | 1 | C->T | CGG=>TGG | R=>W | + | + | - |
| *ccmC* | 113 | 2 | C->T | CCT=>CTT | P=>L | + | + | + |
| *ccmC* | 134 | 2 | C->T | TCT=>TTT | S=>F | + | + | + |
| *ccmC* | 179 | 2 | C->T | GCA=>GTA | A=>V | - | + | + |
| *ccmC* | 184 | 1 | C->T | CGG=>TGG | R=>W | + | + | + |
| *ccmC* | 194 | 2 | C->T | CCG=>CTG | P=>L | + | + | - |
| *ccmC* | 202 | 1 | C->T | CAT=>TAT | H=>Y | - | + | + |
| *ccmC* | 227 | 2 | C->T | TCT=>TTT | S=>F | - | + | + |
| *ccmC* | 230 | 2 | C->T | CCG=>CTG | P=>L | - | + | + |
| *ccmC* | 260 | 2 | C->T | CCC=>CTC | P=>L | - | + | + |
| *ccmC* | 298 | 1 | C->T | CCC=>TTC | P=>F | - | + | + |
| *ccmC* | 299 | 2 | C->T | CCC=>TTC | P=>F | - | + | + |
| *ccmC* | 331 | 1 | C->T | CGG=>TGG | R=>W | - | + | + |
| *ccmC* | 346 | 1 | C->T | CGG=>TGG | R=>W | - | + | + |
| *ccmC* | 358 | 1 | C->T | CGG=>TGG | R=>W | - | + | + |
| *ccmC* | 377 | 2 | C->T | CCA=>CTA | P=>L | - | + | + |
| *ccmC* | 382 | 1 | C->T | CCT=>TCT | P=>S | - | + | + |
| *ccmC* | 395 | 2 | C->T | TCG=>TTG | S=>L | - | + | + |
| *ccmC* | 398 | 2 | C->T | TCC=>TTC | S=>F | - | + | - |
| *ccmC* | 410 | 2 | C->T | CCG=>CTG | P=>L | - | + | + |
| *ccmC* | 458 | 2 | C->T | TCC=>TTC | S=>F | - | + | - |
| *ccmC* | 482 | 2 | C->T | ACA=>ATA | T=>I | - | + | + |
| *ccmC* | 497 | 2 | C->T | TCC=>TTC | S=>F | - | + | + |
| *ccmC* | 499 | 1 | C->T | CCT=>TCT | P=>S | - | + | + |
| *ccmC* | 521 | 2 | C->T | CCG=>CTG | P=>L | - | + | + |
| *ccmC* | 560 | 2 | C->T | ACA=>ATA | T=>I | - | + | + |
| *ccmC* | 575 | 2 | C->T | CCC=>CTC | P=>L | - | + | + |
| *ccmC* | 596 | 2 | C->T | TCT=>TTT | S=>F | - | + | + |
| *ccmC* | 605 | 2 | C->T | TCC=>TTC | S=>F | - | + | + |
| *ccmC* | 608 | 2 | C->T | CCC=>CTC | P=>L | - | + | + |
| *ccmC* | 614 | 2 | C->T | TCA=>TTA | S=>L | - | + | + |
| *ccmC* | 626 | 2 | C->T | TCG=>TTG | S=>L | + | + | + |
| *ccmC* | 628 | 1 | C->T | CTT=>TTT | L=>F | - | + | + |
| *ccmC* | 656 | 2 | C->T | CCA=>CTA | P=>L | + | + | + |
| *ccmC* | 661 | 1 | C->T | CTT=>TTT | L=>F | - | + | + |
| *ccmC* | 695 | 2 | C->T | TCT=>TTT | S=>F | + | - | - |
| *ccmC* | 737 | 2 | C->T | CCA=>CTA | P=>L | - | + | - |
| *ccmFC* | 11 | 2 | C->T | CCG=>CTG | P=>L | + | + | + |
| *ccmFC* | 29 | 2 | C->T | TCC=>TTC | S=>F | + | + | + |
| *ccmFC* | 38 | 2 | C->T | TCC=>TTC | S=>F | + | + | + |
| *ccmFC* | 50 | 2 | C->T | CCT=>CTT | P=>L | + | + | + |
| *ccmFC* | 52 | 1 | C->T | CGT=>TGT | R=>C | + | + | + |
| *ccmFC* | 103 | 1 | C->T | CCC=>TCC | P=>S | + | + | + |
| *ccmFC* | 107 | 2 | C->T | ACA=>AUA | T=>I | + | - | + |
| *ccmFC* | 119 | 2 | C->T | TCT=>TTT | S=>F | - | + | + |
| *ccmFC* | 122 | 2 | C->T | TCC=>TTC | S=>F | - | + | + |
| *ccmFC* | 145 | 1 | C->T | CCC=>TCC | P=>S | + | - | - |
| *ccmFC* | 146 | 2 | C->T | CCC=>CTC | P=>L | + | + | + |
| *ccmFC* | 149 | 2 | C->T | ACA=>ATA | T=>I | - | + | + |
| *ccmFC* | 155 | 2 | C->T | CCC=>CTC | P=>L | + | + | - |
| *ccmFC* | 160 | 1 | C->T | CCC=>TCC | P=>S | - | + | - |
| *ccmFC* | 161 | 2 | C->T | CCC=>CUC | P=>L | - | - | + |
| *ccmFC* | 163 | 1 | C->T | CCU=>UUU | P=>F | - | - | + |
| *ccmFC* | 164 | 2 | C->T | CCT=>CTT | P=>L | - | + | + |
| *ccmFC* | 181 | 1 | C->T | CCC=>TCC | P=>S | + | + | + |
| *ccmFC* | 239 | 2 | C->T | CCC=>CTC | P=>L | - | + | - |
| *ccmFC* | 287 | 2 | C->T | CCA=>CTA | P=>L | + | - | - |
| *ccmFC* | 341 | 2 | C->T | TCA=>TTA | S=>L | - | + | - |
| *ccmFC* | 350 | 2 | C->T | TCC=>TTC | S=>F | - | + | - |
| *ccmFC* | 403 | 1 | C->T | CCA=>TCA | P=>S | + | - | - |
| *ccmFC* | 404 | 2 | C->T | CCA=>CTA | P=>L | - | + | - |
| *ccmFC* | 415 | 1 | C->T | CGC=>TGC | R=>C | + | + | - |
| *ccmFC* | 440 | 2 | C->T | TCC=>TTC | S=>F | - | + | - |
| *ccmFC* | 442 | 1 | C->T | CGC=>TGC | R=>C | + | + | - |
| *ccmFC* | 448 | 1 | C->T | CTT=>TTT | L=>F | - | + | - |
| *ccmFC* | 451 | 1 | C->T | CTC=>TTC | L=>F | - | + | - |
| *ccmFC* | 536 | 2 | C->T | GCT=>GTT | A=>V | - | + | - |
| *ccmFC* | 571 | 1 | C->T | CGC=>TGC | R=>C | + | + | + |
| *ccmFC* | 614 | 2 | C->T | ACA=>ATA | T=>I | - | + | + |
| *ccmFC* | 752 | 2 | C->T | CCG=>CTG | P=>L | + | + | + |
| *ccmFC* | 758 | 2 | C->T | TCG=>TTG | S=>L | + | - | - |
| *ccmFC* | 772 | 1 | C->T | CAT=>TAT | H=>Y | + | - | - |
| *ccmFC* | 820 | 1 | C->T | CCA=>TCA | P=>S | + | - | - |
| *ccmFC* | 832 | 1 | C->T | CCC=>TCC | P=>S | + | - | - |
| *ccmFC* | 857 | 2 | C->T | TCG=>TTG | S=>L | + | - | - |
| *ccmFC* | 935 | 2 | C->T | TCT=>TTT | S=>F | + | + | + |
| *ccmFC* | 937 | 1 | C->T | CTC=>TTC | L=>F | + | + | + |
| *ccmFC* | 971 | 2 | C->T | GCG=>GTG | A=>V | + | + | + |
| *ccmFC* | 989 | 2 | C->T | CCA=>CTA | P=>L | - | + | + |
| *ccmFC* | 1001 | 2 | C->T | CCA=>CTA | P=>L | - | + | + |
| *ccmFC* | 1010 | 2 | C->T | CCG=>CTG | P=>L | - | + | + |
| *ccmFC* | 1075 | 1 | C->T | CCT=>TTT | P=>F | - | + | + |
| *ccmFC* | 1076 | 2 | C->T | CCT=>TTT | P=>F | - | + | + |
| *ccmFC* | 1084 | 1 | C->T | CGG=>TGG | R=>W | - | + | + |
| *ccmFC* | 1105 | 1 | C->T | CCC=>TCC | P=>S | - | + | + |
| *ccmFC* | 1109 | 2 | C->T | CCG=>CTG | P=>L | - | + | + |
| *ccmFC* | 1159 | 1 | C->T | CGG=>TGG | R=>W | - | + | + |
| *ccmFC* | 1165 | 1 | C->T | CGA=>TGA | R=>X | - | + | - |
| *ccmFN* | 2 | 2 | C->T | ACG=>ATG | T=>M | + | + | + |
| *ccmFN* | 28 | 1 | C->T | CCG=>TCG | P=>S | - | + | + |
| *ccmFN* | 29 | 2 | C->T | CCG=>CTG | P=>L | + | - | - |
| *ccmFN* | 34 | 1 | C->T | CTT=>TTT | L=>F | - | + | + |
| *ccmFN* | 38 | 2 | C->T | CCG=>CTG | P=>L | + | + | - |
| *ccmFN* | 47 | 2 | C->T | TCC=>TTC | S=>F | + | + | + |
| *ccmFN* | 98 | 2 | C->T | CCT=>CTT | P=>L | + | + | + |
| *ccmFN* | 113 | 2 | C->T | ACT=>ATT | T=>I | - | + | + |
| *ccmFN* | 124 | 1 | C->T | CTC=>TTC | L=>F | - | + | + |
| *ccmFN* | 137 | 2 | C->T | TCG=>TTG | S=>L | - | + | + |
| *ccmFN* | 142 | 1 | C->T | CGT=>TGT | R=>C | - | + | + |
| *ccmFN* | 149 | 2 | C->T | ACT=>ATT | T=>I | - | + | + |
| *ccmFN* | 151 | 1 | C->T | CCT=>TCT | P=>S | + | + | + |
| *ccmFN* | 163 | 1 | C->T | CCC=>TCC | P=>S | - | + | + |
| *ccmFN* | 208 | 1 | C->T | CCC=>TTC | P=>F | - | + | + |
| *ccmFN* | 209 | 2 | C->T | CCC=>TTC | P=>F | - | + | + |
| *ccmFN* | 220 | 1 | C->T | CCG=>TCG | P=>S | - | + | + |
| *ccmFN* | 254 | 2 | C->T | CCA=>CTA | P=>L | - | + | + |
| *ccmFN* | 257 | 2 | C->T | CCA=>CTA | P=>L | + | + | + |
| *ccmFN* | 259 | 1 | C->T | CGG=>TGG | R=>W | - | + | + |
| *ccmFN* | 265 | 1 | C->T | CGG=>TGG | R=>W | - | + | + |
| *ccmFN* | 272 | 2 | C->T | CCA=>CTA | P=>L | - | + | + |
| *ccmFN* | 290 | 2 | C->T | CCC=>CTC | P=>L | + | + | + |
| *ccmFN* | 295 | 1 | C->T | CGT=>TGT | R=>C | - | + | + |
| *ccmFN* | 310 | 1 | C->T | CCC=>TCC | P=>S | - | + | - |
| *ccmFN* | 353 | 2 | C->T | TCT=>TTT | S=>F | - | + | - |
| *ccmFN* | 362 | 2 | C->T | TCG=>TTG | S=>L | + | + | - |
| *ccmFN* | 379 | 1 | C->T | CCC=>TCC | P=>S | - | + | - |
| *ccmFN* | 392 | 2 | C->T | CCC=>CTC | P=>L | - | + | - |
| *ccmFN* | 428 | 2 |  | CCG=>CUG | P=>L | - | - | + |
| *ccmFN* | 431 | 2 |  | GCC=>GUC | A=>V | - | - | + |
| *ccmFN* | 455 | 2 | C->T | GCT=>GTT | A=>V | - | + | - |
| *ccmFN* | 478 | 1 |  | CUC=>UUC | L=>F | - | - | + |
| *ccmFN* | 506 | 2 | C->T | CCC=>CTC | P=>L | - | + | - |
| *ccmFN* | 544 | 1 | C->T | CTT=>TTT | L=>F | - | + | - |
| *ccmFN* | 598 | 1 | C->T | CTT=>TTT | L=>F | + | - | - |
| *ccmFN* | 689 | 2 | C->T | TCC=>TTC | S=>F | + | + | + |
| *ccmFN* | 691 | 1 | C->T | CCT=>TCT | P=>S | - | + | + |
| *ccmFN* | 694 | 1 | C->T | CTT=>TTT | L=>F | - | + | - |
| *ccmFN* | 698 | 2 | C->T | TCC=>TTC | S=>F | - | + | + |
| *ccmFN* | 701 | 2 | C->T | CCA=>CTA | P=>L | - | + | + |
| *ccmFN* | 704 | 2 | C->T | TCG=>TTG | S=>L | + | + | - |
| *ccmFN* | 742 | 1 | C->T | CGT=>TGT | R=>C | + | + | + |
| *ccmFN* | 755 | 2 | C->T | CCT=>CTT | P=>L | + | + | + |
| *ccmFN* | 764 | 2 | C->T | TCA=>TTA | S=>L | + | + | + |
| *ccmFN* | 776 | 2 | C->T | CCG=>CTG | P=>L | + | + | + |
| *ccmFN* | 851 | 2 | C->T | CCA=>CTA | P=>L | + | + | + |
| *ccmFN* | 989 | 2 | C->T | TCC=>TTC | S=>F | - | + | - |
| *ccmFN* | 1075 | 1 | C->T | CCC=>TCC | P=>S | - | + | - |
| *ccmFN* | 1105 | 1 | C->T | CTC=>TTC | L=>F | - | + | - |
| *ccmFN* | 1109 | 2 | C->T | TCT=>TTT | S=>F | - | + | - |
| *ccmFN* | 1159 | 1 | C->T | CAT=>TAT | H=>Y | - | + | - |
| *ccmFN* | 1324 | 1 | C->T | CGG=>TGG | R=>W | - | + | + |
| *ccmFN* | 1393 | 1 | C->T | CCG=>TCG | P=>S | + | - | - |
| *ccmFN* | 1394 | 2 | C->T | CCG=>CTG | P=>L | + | + | + |
| *ccmFN* | 1402 | 1 | C->T | CGG=>TGG | R=>W | + | + | + |
| *ccmFN* | 1430 | 2 | C->T | CCA=>CTA | P=>L | + | + | + |
| *ccmFN* | 1447 | 1 | C->T | CAT=>TAT | H=>Y | - | + | + |
| *ccmFN* | 1462 | 1 | C->T | CGG=>TGG | R=>W | + | + | + |
| *ccmFN* | 1471 | 1 | C->T | CGG=>TGG | R=>W | - | + | + |
| *ccmFN* | 1480 | 1 | C->T | CGG=>TGG | R=>W | - | + | + |
| *ccmFN* | 1504 | 1 | C->T | CCC=>TTC | P=>F | - | + | + |
| *ccmFN* | 1505 | 2 | C->T | CCC=>TTC | P=>F | - | + | + |
| *ccmFN* | 1531 | 1 | C->T | CGT=>TGT | R=>C | + | + | + |
| *ccmFN* | 1540 | 1 | C->T | CCA=>TCA | P=>S | + | + | + |
| *ccmFN* | 1580 | 2 | C->T | CCC=>CTC | P=>L | - | + | + |
| *ccmFN* | 1598 | 2 | C->T | TCA=>TTA | S=>L | - | + | + |
| *ccmFN* | 1600 | 1 | C->T | CGC=>TGC | R=>C | - | + | + |
| *ccmFN* | 1603 | 1 | C->T | CGT=>TGT | R=>C | - | + | + |
| *ccmFN* | 1610 | 2 | C->T | TCG=>TTG | S=>L | - | + | + |
| *ccmFN* | 1637 | 2 | C->T | TCG=>TTG | S=>L | + | + | + |
| *ccmFN* | 1640 | 2 | C->T | CCA=>CTA | P=>L | - | + | + |
| *ccmFN* | 1657 | 1 | C->T | CCT=>TTT | P=>F | - | + | + |
| *ccmFN* | 1658 | 2 | C->T | CCT=>TTT | P=>F | - | + | + |
| *ccmFN* | 1688 | 2 | C->T | CCA=>CTA | P=>L | + | + | + |
| *ccmFN* | 1693 | 1 | C->T | CGG=>TGG | R=>W | - | + | - |
| *ccmFN* | 1699 | 1 | C->T | CTC=>TTC | L=>F | + | + | + |
| *ccmFN* | 1703 | 2 | C->T | CCT=>CTT | P=>L | + | + | + |
| *ccmFN* | 1724 | 2 | C->T | CCG=>CTG | P=>L | + | - | - |
| *ccmFN* | 1790 | 2 | C->T | ACG=>ATG | T=>M | + | - | - |
| *cob* | 67 | 1 | C->T | CAT=>TAT | H=>Y | - | + | + |
| *cob* | 91 | 1 | C->T | CAT=>TAT | H=>Y | - | + | + |
| *cob* | 109 | 1 | C->T | CCG=>TCG | P=>S | - | + | - |
| *cob* | 110 | 2 | C->T | CCG=>CUG | P=>L | - | - | + |
| *cob* | 124 | 1 | C->T | CGT=>TGT | R=>C | - | + | - |
| *cob* | 128 | 2 | C->T | CCA=>CTA | P=>L | - | + | + |
| *cob* | 131 | 2 | C->T | GCC=>GTC | A=>V | - | + | + |
| *cob* | 158 | 2 | C->T | CCA=>CTA | P=>L | - | + | + |
| *cob* | 188 | 2 | C->T | CCA=>CTA | P=>L | - | + | + |
| *cob* | 278 | 2 | C->T | TCC=>TTC | S=>F | - | + | + |
| *cob* | 293 | 2 | C->T | CCC=>CTC | P=>L | - | + | + |
| *cob* | 316 | 1 | C->T | CAT=>TAT | H=>Y | - | + | + |
| *cob* | 371 | 2 | C->T | TCC=>TTC | S=>F | - | + | + |
| *cob* | 374 | 2 | C->T | CCA=>CTA | P=>L | - | + | + |
| *cob* | 395 | 2 | C->T | TCC=>TTC | S=>F | - | + | + |
| *cob* | 403 | 1 | C->T | CAT=>TAT | H=>Y | - | + | + |
| *cob* | 407 | 2 | C->T | GCA=>GTA | A=>V | - | + | + |
| *cob* | 415 | 1 | C->T | CGG=>TGG | R=>W | + | + | + |
| *cob* | 433 | 1 | C->T | CGG=>TGG | R=>W | + | + | + |
| *cob* | 458 | 2 | C->T | TCA=>TTA | S=>L | + | + | + |
| *cob* | 491 | 2 | C->T | ACA=>ATA | T=>I | + | + | + |
| *cob* | 499 | 1 | C->T | CGG=>TGG | R=>W | - | + | + |
| *cob* | 503 | 2 | C->T | CCG=>CTG | P=>L | - | + | + |
| *cob* | 517 | 1 | C->T | CCC=>TCC | P=>S | - | + | + |
| *cob* | 536 | 2 | C->T | TCA=>TTA | S=>L | - | + | + |
| *cob* | 548 | 2 | C->T | TCT=>TTT | S=>F | - | + | + |
| *cob* | 554 | 2 | C->T | CCT=>CTT | P=>L | + | + | + |
| *cob* | 563 | 2 | C->T | TCA=>TTA | S=>L | - | + | + |
| *cob* | 571 | 1 | C->T | CTT=>TTT | L=>F | - | + | + |
| *cob* | 578 | 2 | C->T | CCA=>CTA | P=>L | + | + | + |
| *cob* | 593 | 2 | C->T | CCT=>CTT | P=>L | - | + | + |
| *cob* | 602 | 2 | C->T | CCG=>CTG | P=>L | - | + | + |
| *cob* | 706 | 1 | C->T | CGG=>TGG | R=>W | + | + | + |
| *cob* | 716 | 2 | C->T | TCC=>TTC | S=>F | + | + | + |
| *cob* | 724 | 1 | C->T | CTC=>TTC | L=>F | + | + | + |
| *cob* | 728 | 2 | C->T | TCT=>TTT | S=>F | + | + | - |
| *cob* | 743 | 2 | C->T | TCT=>TTT | S=>F | + | + | + |
| *cob* | 833 | 2 | C->T | TCC=>TTC | S=>F | + | + | + |
| *cob* | 836 | 2 | C->T | CCA=>CTA | P=>L | + | + | + |
| *cob* | 844 | 1 | C->T | CAT=>TAT | H=>Y | + | + | + |
| *cob* | 854 | 2 | C->T | CCT=>CTT | P=>L | + | + | + |
| *cob* | 875 | 2 | C->T | TCA=>TTA | S=>L | + | + | - |
| *cob* | 907 | 1 | C->T | CCA=>TCA | P=>S | + | - | - |
| *cob* | 908 | 2 | C->T | CCA=>CTA | P=>L | + | - | - |
| *cob* | 988 | 1 | C->T | CGG=>TGG | R=>W | + | + | + |
| *cob* | 1013 | 2 | C->T | CCA=>CTA | P=>L | + | + | + |
| *cob* | 1016 | 2 | C->T | TCA=>TTA | S=>L | + | + | + |
| *cob* | 1021 | 1 | C->T | CGG=>TGG | R=>W | + | + | + |
| *cob* | 1072 | 1 | C->T | CCT=>TCT | P=>S | + | + | + |
| *cob* | 1075 | 1 | C->T | CCA=>TCA | P=>S | + | + | + |
| *cob* | 1082 | 2 | C->T | GCT=>GTT | A=>V | - | + | + |
| *cob* | 1084 | 1 | C->T | CTC=>TTC | L=>F | + | + | + |
| *cob* | 1139 | 2 | C->T | ACC=>ATC | T=>I | - | + | - |
| *cox1* | 2 | 2 | C->T | ACG=>ATG | T=>M | + | + | - |
| *cox1* | 31 | 1 | C->T | CCC=>TCC | P=>S | + | + | + |
| *cox1* | 61 | 1 | C->T | CAT=>TAT | H=>Y | + | + | + |
| *cox1* | 65 | 2 | C->T | TCA=>TTA | S=>L | + | + | - |
| *cox1* | 113 | 2 | C->T | CCA=>CTA | P=>L | + | + | + |
| *cox1* | 200 | 2 | C->T | TCT=>TTT | S=>F | + | + | + |
| *cox1* | 203 | 2 | C->T | CCC=>CTC | P=>L | + | + | + |
| *cox1* | 206 | 2 | C->T | ACG=>ATG | T=>M | + | + | + |
| *cox1* | 209 | 2 | C->T | ACC=>ATC | T=>I | + | + | + |
| *cox1* | 269 | 2 | C->T | CCT=>CTT | P=>L | + | + | + |
| *cox1* | 317 | 2 | C->T | TCC=>TTC | S=>F | + | + | + |
| *cox1* | 319 | 1 | C->T | CGG=>TGG | R=>W | + | + | + |
| *cox1* | 355 | 1 | C->T | CCA=>TCA | P=>S | + | + | + |
| *cox1* | 362 | 2 | C->T | TCG=>TTG | S=>L | + | + | + |
| *cox1* | 446 | 2 | C->T | CCC=>CTC | P=>L | + | + | + |
| *cox1* | 455 | 2 | C->T | TCT=>TTT | S=>F | - | + | + |
| *cox1* | 461 | 2 | C->T | CCT=>CTT | P=>L | + | + | + |
| *cox1* | 467 | 2 | C->T | CCA=>CTA | P=>L | - | + | + |
| *cox1* | 478 | 1 | C->T | CCA=>TCA | P=>S | + | + | + |
| *cox1* | 481 | 1 | C->T | CCC=>TCC | P=>S | - | + | + |
| *cox1* | 560 | 2 | C->T | CCC=>CTC | P=>L | - | + | + |
| *cox1* | 562 | 1 | C->T | CCT=>TTT | P=>F | - | + | + |
| *cox1* | 563 | 2 | C->T | CCT=>TTT | P=>F | - | + | + |
| *cox1* | 578 | 2 | C->T | CCA=>CTA | P=>L | - | + | + |
| *cox1* | 667 | 1 | C->T | CCT=>TCT | P=>S | + | + | + |
| *cox1* | 668 | 2 | C->T | CCT=>CTT | P=>L | + | + | + |
| *cox1* | 713 | 2 | C->T | CCC=>CTC | P=>L | - | + | + |
| *cox1* | 715 | 1 | C->T | CCT=>TTT | P=>F | - | + | + |
| *cox1* | 716 | 2 | C->T | CCT=>TTT | P=>F | - | + | + |
| *cox1* | 724 | 1 | C->T | CTC=>TTC | L=>F | + | + | + |
| *cox1* | 755 | 2 | C->T | CCG=>CTG | P=>L | + | + | + |
| *cox1* | 796 | 1 | C->T | CCG=>TCG | P=>S | + | + | + |
| *cox1* | 842 | 2 | C->T | ACC=>ATC | T=>I | + | + | + |
| *cox1* | 848 | 2 | C->T | ACT=>ATT | T=>I | + | + | + |
| *cox1* | 871 | 1 | C->T | CGG=>TGG | R=>W | + | + | + |
| *cox1* | 919 | 1 | C->T | CAC=>TAC | H=>Y | + | + | + |
| *cox1* | 923 | 2 | C->T | TCT=>TTT | S=>F | + | + | + |
| *cox1* | 1025 | 2 | C->T | CCA=>CTA | P=>L | + | + | + |
| *cox1* | 1028 | 2 | C->T | TCT=>TTT | S=>F | + | + | + |
| *cox1* | 1040 | 2 | C->T | TCC=>TTC | S=>F | + | + | + |
| *cox1* | 1052 | 2 | C->T | TCC=>TTC | S=>F | - | + | + |
| *cox1* | 1067 | 2 | C->T | CCT=>CTT | P=>L | - | + | + |
| *cox1* | 1082 | 2 | C->T | CCG=>CTG | P=>L | - | + | + |
| *cox1* | 1090 | 1 | C->T | CCC=>TCC | P=>S | - | + | + |
| *cox1* | 1120 | 1 | C->T | CAT=>TAT | H=>Y | + | + | + |
| *cox1* | 1138 | 1 | C->T | CCC=>TTC | P=>F | - | + | + |
| *cox1* | 1139 | 2 | C->T | CCC=>TTC | P=>F | - | + | + |
| *cox1* | 1151 | 2 | C->T | CCT=>CTT | P=>L | - | + | + |
| *cox1* | 1153 | 1 | C->T | CCC=>TCC | P=>S | - | + | + |
| *cox1* | 1175 | 2 | C->T | TCA=>TTA | S=>L | - | + | + |
| *cox1* | 1195 | 1 | C->T | CGG=>TGG | R=>W | - | + | + |
| *cox1* | 1211 | 2 | C->T | TCT=>TTT | S=>F | - | + | + |
| *cox1* | 1235 | 2 | C->T | TCA=>TTA | S=>L | + | + | + |
| *cox1* | 1252 | 1 | C->T | CGG=>TGG | R=>W | + | + | + |
| *cox1* | 1282 | 1 | C->T | CTC=>TTC | L=>F | + | + | + |
| *cox1* | 1301 | 2 | C->T | TCG=>TTG | S=>L | + | + | + |
| *cox1* | 1384 | 1 | C->T | CAT=>TAT | H=>Y | + | + | + |
| *cox1* | 1405 | 1 | C->T | CGT=>TGT | R=>C | + | + | + |
| *cox1* | 1408 | 1 | C->T | CGT=>TGT | R=>C | + | + | + |
| *cox1* | 1415 | 2 | C->T | TCC=>TTC | S=>F | + | + | + |
| *cox1* | 1484 | 2 | C->T | CCA=>CTA | P=>L | - | + | - |
| *cox2* | 16 | 1 | C->T | CTC=>TTC | L=>F | + | - | - |
| *cox2* | 49 | 1 | C->T | CCT=>TCT | P=>S | + | - | - |
| *cox2* | 83 | 2 | C->T | TCT=>TTT | S=>F | + | + | - |
| *cox2* | 125 | 2 | C->T | TCA=>TTA | S=>L | + | + | + |
| *cox2* | 149 | 2 | C->T | CCA=>CTA | P=>L | + | + | + |
| *cox2* | 161 | 2 | C->T | TCG=>TTG | S=>L | + | + | + |
| *cox2* | 166 | 1 | C->T | CTC=>TTC | L=>F | - | + | + |
| *cox2* | 173 | 2 | C->T | TCA=>TTA | S=>L | + | + | - |
| *cox2* | 175 | 1 | C->T | CGG=>TGG | R=>W | + | + | - |
| *cox2* | 191 | 2 | C->T | GCU=>GUU | A=>V | - | - | + |
| *cox2* | 194 | 2 | C->T | TCA=>TTA | S=>L | + | + | + |
| *cox2* | 265 | 1 | C->T | CGG=>TGG | R=>W | + | + | + |
| *cox2* | 290 | 2 | C->T | CCG=>CTG | P=>L | + | + | + |
| *cox2* | 320 | 2 | C->T | CCG=>CTG | P=>L | + | + | + |
| *cox2* | 385 | 1 | C->T | CGG=>TGG | R=>W | + | + | + |
| *cox2* | 391 | 1 | C->T | CGG=>TGG | R=>W | + | + | + |
| *cox2* | 443 | 2 | C->T | TCT=>TTT | S=>F | + | + | + |
| *cox2* | 473 | 2 | C->T | CCA=>CTA | P=>L | + | + | + |
| *cox2* | 488 | 2 | C->T | TCG=>TTG | S=>L | + | + | + |
| *cox2* | 497 | 2 | C->T | TCG=>TTG | S=>L | + | + | + |
| *cox2* | 539 | 2 | C->T | CCA=>CTA | P=>L | + | + | + |
| *cox2* | 566 | 2 | C->T | GCA=>GTA | A=>V | + | + | + |
| *cox2* | 571 | 1 | C->T | CAU=>UAU | H=>Y | - | - | + |
| *cox2* | 577 | 1 | C->T | CGG=>TGG | R=>W | + | + | + |
| *cox2* | 644 | 2 | C->T | CCG=>CTG | P=>L | + | + | + |
| *cox2* | 688 | 1 | C->T | CGC=>TGC | R=>C | + | + | - |
| *cox2* | 712 | 1 | C->T | CCU=>UCU | P=>S | - | - | + |
| *cox2* | 719 | 2 | C->T | GCC=>GTC | A=>V | - | + | + |
| *cox2* | 754 | 1 | C->T | CGG=>TGG | R=>W | + | + | - |
| *cox2* | 758 | 2 | C->T | GCA=>GTA | A=>V | - | + | + |
| *cox3* | 2 | 2 | C->T | ACG=>ATG | T=>M | + | - | - |
| *cox3* | 19 | 1 | C->T | CAT=>TAT | H=>Y | + | + | + |
| *cox3* | 155 | 2 | C->T | CCA=>CTA | P=>L | + | + | + |
| *cox3* | 172 | 1 | C->T | CGG=>TGG | R=>W | + | + | + |
| *cox3* | 256 | 1 | C->T | CCG=>TCG | P=>S | + | - | - |
| *cox3* | 257 | 2 | C->T | CCG=>CTG | P=>L | + | + | + |
| *cox3* | 283 | 1 | C->T | CTC=>TTC | L=>F | + | + | + |
| *cox3* | 298 | 1 | C->T | CGG=>TGG | R=>W | + | + | + |
| *cox3* | 349 | 1 | C->T | CGG=>TGG | R=>W | + | + | + |
| *cox3* | 506 | 2 | C->T | TCA=>TTA | S=>L | - | + | + |
| *cox3* | 548 | 2 | C->T | TCT=>TTT | S=>F | + | - | - |
| *cox3* | 580 | 1 | C->T | CAC=>TAC | H=>Y | + | + | + |
| *cox3* | 586 | 1 | C->T | CCT=>TCT | P=>S | + | + | + |
| *cox3* | 599 | 2 | C->T | TCA=>TTA | S=>L | + | + | + |
| *cox3* | 647 | 2 | C->T | CCA=>CTA | P=>L | + | + | + |
| *cox3* | 674 | 2 | C->T | TCG=>TTG | S=>L | + | + | + |
| *cox3* | 734 | 2 | C->T | TCC=>TTC | S=>F | - | + | + |
| *cox3* | 735 | 0 | C->T | TCC=>TCT | S=>S | + | - | - |
| *cox3* | 748 | 1 | C->T | CGG=>TGG | R=>W | + | + | - |
| *cox3* | 758 | 2 | C->T | CCA=>CTA | P=>L | + | + | - |
| *cox3* | 771 | 0 | C->T | ATC=>ATT | I=>I | + | - | - |
| *cox3* | 788 | 2 | C->T | CCA=>CTA | P=>L | + | - | - |
| *matR* | 8 | 2 | C->T | TCC=>TTC | S=>F | + | + | - |
| *matR* | 77 | 2 | C->T | TCC=>TTC | S=>F | - | + | - |
| *matR* | 118 | 1 | C->T | CCG=>TCG | P=>S | - | + | - |
| *matR* | 173 | 2 | C->T | TCA=>TTA | S=>L | + | - | - |
| *matR* | 238 | 1 | C->T | CCA=>TCA | P=>S | + | + | - |
| *matR* | 295 | 1 | C->T | CTC=>TTC | L=>F | - | + | - |
| *matR* | 332 | 2 | C->T | CCG=>CTG | P=>L | + | - | - |
| *matR* | 355 | 1 | C->T | CCG=>TCG | P=>S | - | + | - |
| *matR* | 359 | 2 | C->T | GCG=>GTG | A=>V | - | + | - |
| *matR* | 379 | 1 | C->T | CCC=>TCC | P=>S | - | + | - |
| *matR* | 446 | 2 | C->T | ACT=>ATT | T=>I | - | + | - |
| *matR* | 473 | 2 | C->T | GCC=>GTC | A=>V | - | + | - |
| *matR* | 487 | 1 | C->T | CCT=>TCT | P=>S | - | + | - |
| *matR* | 532 | 1 | C->T | CCC=>TCC | P=>S | - | + | - |
| *matR* | 547 | 1 | C->T | CCC=>TCC | P=>S | - | + | - |
| *matR* | 593 | 2 | C->T | GCG=>GTG | A=>V | - | + | - |
| *matR* | 746 | 2 | C->T | TCC=>TTC | S=>F | - | + | - |
| *matR* | 874 | 1 | C->T | CCT=>TTT | P=>F | - | + | - |
| *matR* | 875 | 2 | C->T | CCT=>TTT | P=>F | - | + | - |
| *matR* | 953 | 2 | C->T | CCC=>CTC | P=>L | - | + | - |
| *matR* | 1052 | 2 | C->T | CCC=>CTC | P=>L | - | + | - |
| *matR* | 1172 | 2 | C->T | CCG=>CTG | P=>L | + | + | - |
| *matR* | 1271 | 2 | C->T | CCC=>CTC | P=>L | + | + | - |
| *matR* | 1355 | 2 | C->T | CCG=>CTG | P=>L | - | + | - |
| *matR* | 1382 | 2 | C->T | GCG=>GTG | A=>V | - | + | - |
| *matR* | 1985 | 2 | C->T | GCT=>GTT | A=>V | - | + | - |
| *matR* | 2167 | 1 | C->T | CCT=>TCT | P=>S | - | + | - |
| *matR* | 2257 | 1 | C->T | CCT=>TCT | P=>S | - | + | - |
| *matR* | 2330 | 2 | C->T | CCC=>CTC | P=>L | - | + | - |
| *matR* | 2746 | 1 | C->T | CCC=>TCC | P=>S | - | + | - |
| *matR* | 2749 | 1 | C->T | CCT=>TCT | P=>S | - | + | - |
| *matR* | 2969 | 2 | C->T | ACC=>ATC | T=>I | + | + | - |
| *matR* | 3050 | 2 | C->T | ACC=>ATC | T=>I | - | + | + |
| *matR* | 3097 | 1 | C->T | CGG=>TGG | R=>W | + | + | + |
| *matR* | 3124 | 1 | C->T | CCG=>TCG | P=>S | + | - | - |
| *matR* | 3125 | 2 | C->T | CCG=>CTG | P=>L | + | + | + |
| *matR* | 3130 | 1 | C->T | CAC=>TAC | H=>Y | - | + | + |
| *matR* | 3142 | 1 | C->T | CGC=>TGC | R=>C | + | + | + |
| *matR* | 3203 | 2 | C->T | TCC=>TTC | S=>F | + | + | + |
| *matR* | 3209 | 2 | C->T | CCT=>CTT | P=>L | + | + | + |
| *matR* | 3248 | 2 | C->T | CCG=>CTG | P=>L | + | + | + |
| *matR* | 3302 | 2 | C->T | TCC=>TTC | S=>F | - | + | + |
| *matR* | 3394 | 1 | C->T | CAG=>TAG | Q=>X | - | + | - |
| *mttB* | 2 | 2 | C->T | ACG=>ATG | T=>M | - | + | - |
| *mttB* | 23 | 2 | C->T | CCU=>CUU | P=>L | - | - | + |
| *mttB* | 26 | 2 | C->T | CCG=>CTG | P=>L | + | + | + |
| *mttB* | 35 | 2 | C->T | GCT=>GTT | A=>V | - | + | + |
| *mttB* | 47 | 2 | C->T | UCC=>UUC | S=>F | - | - | + |
| *mttB* | 59 | 2 | C->T | TCG=>TTG | S=>L | + | + | + |
| *mttB* | 67 | 1 | C->T | CCC=>TCC | P=>S | + | - | - |
| *mttB* | 68 | 2 | C->T | CCC=>CTC | P=>L | - | + | + |
| *mttB* | 74 | 2 | C->T | TCG=>TTG | S=>L | - | + | + |
| *mttB* | 91 | 1 | C->T | CAT=>TAT | H=>Y | - | + | + |
| *mttB* | 100 | 1 | C->T | CCG=>TCG | P=>S | - | + | + |
| *mttB* | 110 | 2 | C->T | TCA=>TTA | S=>L | - | + | + |
| *mttB* | 116 | 2 | C->T | TCT=>TTT | S=>F | - | + | + |
| *mttB* | 119 | 2 | C->T | CCA=>CTA | P=>L | - | + | + |
| *mttB* | 122 | 2 | C->T | TCG=>TTG | S=>L | - | + | + |
| *mttB* | 154 | 1 | C->T | CCG=>TCG | P=>S | - | + | + |
| *mttB* | 160 | 1 | C->T | CCT=>TTT | P=>F | - | + | + |
| *mttB* | 161 | 2 | C->T | CCT=>TTT | P=>F | - | + | + |
| *mttB* | 166 | 1 | C->T | CGT=>TGT | R=>C | - | + | + |
| *mttB* | 176 | 2 | C->T | TCG=>TTG | S=>L | - | + | + |
| *mttB* | 188 | 2 | C->T | TCT=>TTT | S=>F | - | + | + |
| *mttB* | 212 | 2 | C->T | CCA=>CTA | P=>L | - | + | + |
| *mttB* | 220 | 1 | C->T | CGC=>TGC | R=>C | - | + | + |
| *mttB* | 224 | 2 | C->T | UCC=>UUC | S=>F | - | - | + |
| *mttB* | 230 | 2 | C->T | TCC=>TTC | S=>F | - | + | + |
| *mttB* | 242 | 2 | C->T | TCC=>TTC | S=>F | - | + | - |
| *mttB* | 265 | 1 | C->T | CCT=>TTT | P=>F | - | + | + |
| *mttB* | 266 | 2 | C->T | CCT=>TTT | P=>F | - | + | + |
| *mttB* | 269 | 2 | C->T | TCG=>TTG | S=>L | - | + | + |
| *mttB* | 319 | 1 | C->T | CAC=>TAC | H=>Y | - | + | + |
| *mttB* | 323 | 2 | C->T | CCA=>CTA | P=>L | - | + | + |
| *mttB* | 341 | 2 | C->T | TCC=>TTC | S=>F | - | + | + |
| *mttB* | 344 | 2 | C->T | CCG=>CTG | P=>L | + | + | + |
| *mttB* | 361 | 1 | C->T | CUU=>UUU | L=>F | - | - | + |
| *mttB* | 367 | 1 | C->T | CGG=>TGG | R=>W | - | + | + |
| *mttB* | 371 | 2 | C->T | GCA=>GTA | A=>V | - | + | + |
| *mttB* | 385 | 1 | C->T | CGG=>TGG | R=>W | - | + | + |
| *mttB* | 395 | 2 | C->T | CCA=>CTA | P=>L | - | + | + |
| *mttB* | 404 | 2 | C->T | GCG=>GTG | A=>V | - | + | + |
| *mttB* | 460 | 1 | C->T | CAT=>TAT | H=>Y | - | + | + |
| *mttB* | 470 | 2 | C->T | TCA=>TTA | S=>L | - | + | + |
| *mttB* | 485 | 2 | C->T | CCG=>CTG | P=>L | - | + | + |
| *mttB* | 493 | 1 | C->T | CCA=>TCA | P=>S | - | + | + |
| *mttB* | 496 | 1 | C->T | CCA=>TCA | P=>S | - | + | + |
| *mttB* | 502 | 1 | C->T | CGC=>TGC | R=>C | - | + | + |
| *mttB* | 524 | 2 | C->T | GCG=>GTG | A=>V | - | + | + |
| *mttB* | 529 | 1 | C->T | CGT=>TGT | R=>C | - | + | + |
| *mttB* | 536 | 2 | C->T | CCA=>CTA | P=>L | - | + | + |
| *mttB* | 542 | 2 | C->T | CCG=>CTG | P=>L | - | + | + |
| *mttB* | 566 | 2 | C->T | TCC=>TTC | S=>F | - | + | + |
| *mttB* | 584 | 2 | C->T | TCT=>TTT | S=>F | - | + | + |
| *mttB* | 596 | 2 | C->T | TCC=>TTC | S=>F | - | + | + |
| *mttB* | 598 | 1 | C->T | CCG=>TCG | P=>S | - | + | + |
| *mttB* | 604 | 1 | C->T | CCC=>UUC | P=>F | - | - | + |
| *mttB* | 605 | 2 | C->T | CCC=>CTC | P=>L | - | + | + |
| *mttB* | 659 | 2 | C->T | TCG=>TTG | S=>L | + | + | - |
| *mttB* | 662 | 2 | C->T | CCT=>CTT | P=>L | - | + | + |
| *mttB* | 671 | 2 | C->T | CCG=>CTG | P=>L | + | + | + |
| *mttB* | 692 | 2 | C->T | TCT=>TTT | S=>F | + | + | + |
| *mttB* | 701 | 2 | C->T | TCG=>TTG | S=>L | + | + | + |
| *nad1* | 2 | 2 | C->T | ACG=>ATG | T=>M | + | + | - |
| *nad1* | 11 | 2 | C->T | GCT=>GTT | A=>V | + | - | - |
| *nad1* | 29 | 2 | C->T | CCC=>CTC | P=>L | + | + | + |
| *nad1* | 68 | 2 | C->T | TCA=>TTA | S=>L | + | + | + |
| *nad1* | 97 | 1 | C->T | CTC=>TTC | L=>F | + | + | + |
| *nad1* | 134 | 2 | C->T | TCG=>TTG | S=>L | + | - | - |
| *nad1* | 167 | 2 | C->T | TCG=>TTG | S=>L | + | + | + |
| *nad1* | 173 | 2 | C->T | TCA=>TTA | S=>L | + | + | + |
| *nad1* | 193 | 1 | C->T | CCA=>TCA | P=>S | + | + | + |
| *nad1* | 215 | 2 | C->T | TCT=>TTT | S=>F | + | + | + |
| *nad1* | 221 | 2 | C->T | TCT=>TTT | S=>F | + | + | + |
| *nad1* | 245 | 2 | C->T | TCT=>TTT | S=>F | + | + | + |
| *nad1* | 265 | 1 | C->T | CGG=>TGG | R=>W | + | + | + |
| *nad1* | 281 | 2 | C->T | TCT=>TTT | S=>F | + | + | + |
| *nad1* | 286 | 1 | C->T | CAT=>TAT | H=>Y | - | + | + |
| *nad1* | 299 | 2 | C->T | TCG=>TTG | S=>L | + | + | + |
| *nad1* | 308 | 2 | C->T | TCG=>TTG | S=>L | + | + | + |
| *nad1* | 325 | 1 | C->T | CAT=>TAT | H=>Y | + | + | + |
| *nad1* | 332 | 2 | C->T | TCT=>TTT | S=>F | + | + | + |
| *nad1* | 404 | 2 | C->T | CCG=>CTG | P=>L | + | + | + |
| *nad1* | 413 | 2 | C->T | TCA=>TTA | S=>L | + | + | + |
| *nad1* | 436 | 1 | C->T | CCT=>TCT | P=>S | + | + | + |
| *nad1* | 439 | 1 | C->T | CAC=>TAC | H=>Y | + | + | + |
| *nad1* | 481 | 1 | C->T | CGT=>TGT | R=>C | - | + | + |
| *nad1* | 490 | 1 | C->T | CCT=>TCT | P=>S | - | + | + |
| *nad1* | 493 | 1 | C->T | CGT=>TGT | R=>C | - | + | + |
| *nad1* | 500 | 2 | C->T | TCG=>TTG | S=>L | + | + | + |
| *nad1* | 515 | 2 | C->T | ACG=>ATG | T=>M | - | + | + |
| *nad1* | 569 | 2 | C->T | TCC=>TTC | S=>F | + | + | + |
| *nad1* | 572 | 2 | C->T | TCC=>TTC | S=>F | - | + | + |
| *nad1* | 577 | 1 | C->T | CCT=>TCT | P=>S | + | + | + |
| *nad1* | 580 | 1 | C->T | CGT=>TGT | R=>C | - | + | + |
| *nad1* | 584 | 2 | C->T | CCA=>CTA | P=>L | - | + | + |
| *nad1* | 607 | 1 | C->T | CTT=>TTT | L=>F | + | + | + |
| *nad1* | 635 | 2 | C->T | TCA=>TTA | S=>L | + | + | + |
| *nad1* | 653 | 2 | C->T | GCA=>GTA | A=>V | + | + | + |
| *nad1* | 664 | 1 | C->T | CCG=>TCG | P=>S | - | + | + |
| *nad1* | 674 | 2 | C->T | TCT=>TTT | S=>F | - | + | + |
| *nad1* | 683 | 2 | C->T | TCT=>TTT | S=>F | - | + | + |
| *nad1* | 685 | 1 | C->T | CTT=>TTT | L=>F | - | + | + |
| *nad1* | 689 | 2 | C->T | CCA=>CTA | P=>L | - | + | + |
| *nad1* | 743 | 2 | C->T | CCA=>CTA | P=>L | + | + | + |
| *nad1* | 751 | 1 | C->T | CGG=>TGG | R=>W | + | + | + |
| *nad1* | 806 | 2 | C->T | TCT=>TTT | S=>F | + | + | + |
| *nad1* | 844 | 1 | C->T | CGG=>TGG | R=>W | + | + | + |
| *nad1* | 884 | 2 | C->T | TCA=>TTA | S=>L | + | + | + |
| *nad1* | 898 | 1 | C->T | CGG=>TGG | R=>W | + | + | + |
| *nad1* | 911 | 2 | C->T | TCG=>TTG | S=>L | + | + | + |
| *nad1* | 928 | 1 | C->T | CGG=>TGG | R=>W | + | + | - |
| *nad2* | 11 | 2 | C->T | CCT=>CTT | P=>L | - | + | + |
| *nad2* | 17 | 2 | C->T | CCA=>CTA | P=>L | + | + | + |
| *nad2* | 41 | 2 | C->T | CCC=>CTC | P=>L | - | + | - |
| *nad2* | 62 | 2 | C->T | TCG=>TTG | S=>L | + | + | + |
| *nad2* | 65 | 2 | C->T | CCC=>CTC | P=>L | + | + | + |
| *nad2* | 119 | 2 | C->T | GCA=>GTA | A=>V | - | + | + |
| *nad2* | 146 | 2 | C->T | CCC=>CTC | P=>L | - | + | + |
| *nad2* | 253 | 1 | C->T | CGC=>TGC | R=>C | - | + | + |
| *nad2* | 335 | 2 | C->T | TCT=>TTT | S=>L | + | + | + |
| *nad2* | 350 | 2 | C->T | TCA=>TTA | S=>L | + | + | + |
| *nad2* | 356 | 2 | C->T | CCA=>CTA | P=>L | + | + | + |
| *nad2* | 361 | 1 | C->T | CCT=>TCT | P=>S | + | + | + |
| *nad2* | 416 | 2 | C->T | TCA=>TTA | S=>L | + | + | + |
| *nad2* | 442 | 1 | C->T | CCT=>TTT | P=>F | + | + | + |
| *nad2* | 443 | 2 | C->T | CCT=>TTT | =>F | + | + | + |
| *nad2* | 523 | 1 | C->T | CCC=>TCC | P=>S | + | + | + |
| *nad2* | 539 | 2 | C->T | TCG=>TTG | S=>L | + | + | + |
| *nad2* | 683 | 2 | C->T | TCT=>TTT | S=>L | + | + | + |
| *nad2* | 689 | 2 | C->T | TCC=>TTT | S=>F | + | + | + |
| *nad2* | 690 | 3 | C->T | TCC=>TTT | S=>F | + | - | - |
| *nad2* | 733 | 1 | C->T | CAT=>TAT | H=>Y | + | + | + |
| *nad2* | 767 | 2 | C->T | TCC=>TTC | S=>F | - | + | - |
| *nad2* | 793 | 1 | C->T | CCT=>TTT | P=>F | - | + | + |
| *nad2* | 794 | 2 | C->T | CCT=>TTT | P=>F | - | + | + |
| *nad2* | 815 | 2 | C->T | TCT=>TTT | S=>F | + | + | + |
| *nad2* | 818 | 2 | C->T | ACC=>ATC | T=>I | - | + | + |
| *nad2* | 820 | 1 | C->T | CAT=>TAT | H=>Y | - | + | + |
| *nad2* | 842 | 2 | C->T | TCG=>TTG | S=>L | + | + | + |
| *nad2* | 862 | 1 | C->T | CGC=>TGC | R=>C | + | + | + |
| *nad2* | 874 | 1 | C->T | CCC=>TCC | P=>S | + | + | + |
| *nad2* | 884 | 2 | C->T | CCA=>CTA | P=>L | - | + | + |
| *nad2* | 926 | 2 | C->T | CCT=>CTT | P=>L | - | + | + |
| *nad2* | 934 | 1 | C->T | CAT=>TAT | H=>Y | - | + | + |
| *nad2* | 940 | 1 | C->T | CCG=>TCG | P=>S | - | + | + |
| *nad2* | 964 | 1 | C->T | CGT=>TGT | R=>C | - | + | + |
| *nad2* | 968 | 2 | C->T | ACC=>ATC | T=>I | - | + | + |
| *nad2* | 1027 | 1 | C->T | CAT=>TAT | H=>Y | + | + | + |
| *nad2* | 1034 | 2 | C->T | TCA=>TTA | S=>L | + | + | + |
| *nad2* | 1052 | 2 | C->T | TCC=>TTC | S=>F | + | + | + |
| *nad2* | 1064 | 2 | C->T | CCG=>CTG | P=>L | + | + | + |
| *nad2* | 1112 | 2 | C->T | CCA=>CTA | P=>L | - | + | + |
| *nad2* | 1133 | 2 | C->T | CCG=>CTG | P=>L | - | + | + |
| *nad2* | 1145 | 2 | C->T | TCT=>TTT | S=>F | - | + | + |
| *nad2* | 1160 | 2 | C->T | TCC=>TTC | S=>F | - | + | + |
| *nad2* | 1162 | 1 | C->T | CCA=>TCA | P=>S | - | + | + |
| *nad2* | 1195 | 1 | C->T | CGC=>TGC | R=>C | - | + | + |
| *nad2* | 1207 | 1 | C->T | CAT=>TAT | H=>Y | - | + | + |
| *nad2* | 1211 | 2 | C->T | TCG=>TTG | S=>L | - | + | + |
| *nad2* | 1214 | 2 | C->T | TCC=>TTC | S=>F | - | + | + |
| *nad2* | 1217 | 2 | C->T | TCC=>TTC | S=>F | - | + | + |
| *nad2* | 1240 | 1 | C->T | CAC=>TAC | H=>Y | - | + | + |
| *nad2* | 1247 | 2 | C->T | CCA=>CTA | P=>L | + | + | + |
| *nad2* | 1282 | 1 | C->T | CGT=>TGT | R=>C | + | + | + |
| *nad2* | 1288 | 1 | C->T | CAT=>TAT | H=>Y | - | + | + |
| *nad2* | 1304 | 2 | C->T | GCG=>GTG | A=>V | + | + | + |
| *nad2* | 1318 | 1 | C->T | CCC=>TTC | P=>F | - | + | + |
| *nad2* | 1319 | 2 | C->T | CCC=>TTC | P=>F | - | + | + |
| *nad2* | 1376 | 2 | C->T | CCA=>CTA | P=>L | + | + | + |
| *nad2* | 1379 | 2 | C->T | CCA=>CTA | P=>L | - | + | + |
| *nad2* | 1397 | 2 | C->T | TCC=>TTC | S=>F | - | + | + |
| *nad2* | 1406 | 2 | C->T | TCA=>TTA | S=>L | - | + | + |
| *nad2* | 1415 | 2 | C->T | CCG=>CTG | P=>L | - | + | + |
| *nad2* | 1423 | 1 | C->T | CCT=>TCT | P=>S | - | + | + |
| *nad2* | 1430 | 2 | C->T | TCG=>TTG | S=>L | - | + | + |
| *nad2* | 1451 | 2 | C->T | ACG=>ATG | T=>M | - | + | + |
| *nad2* | 1457 | 2 | C->T | CCA=>CTA | P=>L | - | + | + |
| *nad3* | 22 | 1 | C->T | CGT=>TGT | R=>C | + | + | + |
| *nad3* | 31 | 1 | C->T | CCA=>TTA | P=>L | + | - | - |
| *nad3* | 32 | 2 | C->T | CCA=>TTA | P=>L | + | + | + |
| *nad3* | 124 | 1 | C->T | CAC=>TAC | H=>Y | + | + | + |
| *nad3* | 137 | 2 | C->T | TCC=>TTC | S=>F | + | + | + |
| *nad3* | 181 | 1 | C->T | CAT=>TAT | H=>Y | - | + | + |
| *nad3* | 185 | 2 | C->T | CCG=>CTG | P=>L | - | + | + |
| *nad3* | 190 | 1 | C->T | CCC=>TCC | P=>S | - | + | + |
| *nad3* | 199 | 1 | C->T | CCT=>TTT | P=>F | - | + | + |
| *nad3* | 200 | 2 | C->T | CCT=>TTT | P=>F | - | + | + |
| *nad3* | 206 | 2 | C->T | ACC=>ATC | T=>I | - | + | + |
| *nad3* | 208 | 1 | C->T | CCC=>TTC | P=>F | - | + | + |
| *nad3* | 209 | 2 | C->T | CCC=>TTC | P=>F | - | + | + |
| *nad3* | 227 | 2 | C->T | TCC=>TTC | S=>F | - | + | + |
| *nad3* | 251 | 2 | C->T | CCC=>CTC | P=>L | - | + | + |
| *nad3* | 269 | 2 | C->T | TCC=>TTC | S=>F | + | + | + |
| *nad3* | 305 | 2 | C->T | TCG=>TTG | S=>L | + | + | + |
| *nad3* | 322 | 1 | C->T | CAT=>TAT | H=>Y | + | + | + |
| *nad3* | 349 | 1 | C->T | CGG=>TGG | R=>W | + | + | - |
| *nad4L* | 20 | 2 | C->T | TCA=>TTA | S=>L | - | + | - |
| *nad4L* | 28 | 1 | C->T | CCT=>TCT | P=>S | + | + | + |
| *nad4L* | 40 | 1 | C->T | CCC=>TTC | P=>F | + | + | + |
| *nad4L* | 41 | 2 | C->T | CCC=>TTC | P=>F | - | + | + |
| *nad4L* | 47 | 2 | C->T | TCG=>TTG | S=>L | - | + | + |
| *nad4L* | 55 | 1 | C->T | CGG=>TGG | R=>W | - | + | + |
| *nad4L* | 65 | 2 | C->T | TCC=>TTC | S=>F | - | + | - |
| *nad4L* | 68 | 2 | C->T | CCA=>CTA | P=>L | - | + | + |
| *nad4L* | 95 | 2 | C->T | TCA=>TTA | S=>L | - | + | + |
| *nad4L* | 110 | 2 | C->T | TCA=>TTA | S=>L | - | + | + |
| *nad4L* | 116 | 2 | C->T | TCA=>TTA | S=>L | + | + | + |
| *nad4L* | 119 | 2 | C->T | CCA=>CTA | P=>L | - | + | + |
| *nad4L* | 136 | 1 | C->T | CCT=>TTT | P=>F | - | + | + |
| *nad4L* | 137 | 2 | C->T | CCT=>CTT | P=>L | + | + | + |
| *nad4L* | 140 | 2 | C->T | TCG=>TTG | S=>L | - | + | + |
| *nad4L* | 148 | 1 | C->T | CCC=>TCC | P=>S | - | + | + |
| *nad4L* | 158 | 2 | C->T | CCG=>CTG | P=>L | - | + | + |
| *nad4L* | 179 | 2 | C->T | TCA=>TTA | S=>L | + | + | + |
| *nad4L* | 191 | 2 | C->T | TCG=>TTG | S=>L | + | + | + |
| *nad4L* | 239 | 2 | C->T | CCG=>CTG | P=>L | + | + | - |
| *nad4L* | 281 | 2 | C->T | TCT=>TTT | S=>F | + | + | - |
| *nad4L* | 289 | 1 | C->T | CGC=>TGC | R=>C | + | + | + |
| *nad4L* | 301 | 1 | C->T | CGA=>TGA | R=>U | + | + | + |
| *nad4* | 2 | 2 | C->T | ACG=>ATG | T=>M | + | + | + |
| *nad4* | 16 | 1 | C->T | CGT=>TGT | R=>C | + | + | - |
| *nad4* | 29 | 2 | C->T | TCC=>TTC | S=>F | + | + | - |
| *nad4* | 49 | 1 | C->T | CCG=>TTG | P=>L | + | - | - |
| *nad4* | 50 | 2 | C->T | CCG=>TTG | P=>L | + | + | - |
| *nad4* | 137 | 2 | C->T | CCC=>CTT | P=>L | + | + | + |
| *nad4* | 138 | 0 | C->T | CCC=>CTT | P=>L | + | - | - |
| *nad4* | 166 | 1 | C->T | CGG=>TGG | R=>W | + | + | + |
| *nad4* | 229 | 1 | C->T | CAT=>TAT | H=>Y | + | + | + |
| *nad4* | 268 | 1 | C->T | CCC=>TCC | P=>S | + | + | + |
| *nad4* | 272 | 2 | C->T | TCA=>TTA | S=>L | + | + | + |
| *nad4* | 299 | 2 | C->T | CCA=>CTA | P=>L | + | + | + |
| *nad4* | 310 | 1 | C->T | CGC=>TGC | R=>C | + | + | + |
| *nad4* | 371 | 2 | C->T | CCA=>CTA | P=>L | + | + | + |
| *nad4* | 376 | 1 | C->T | CGC=>TGC | R=>C | - | + | + |
| *nad4* | 403 | 1 | C->T | CGC=>TGC | R=>C | + | + | + |
| *nad4* | 410 | 2 | C->T | CCG=>CTG | P=>L | - | + | + |
| *nad4* | 427 | 1 | C->T | CAT=>TAT | H=>Y | + | + | + |
| *nad4* | 436 | 1 | C->T | CCC=>TTC | P=>F | + | + | + |
| *nad4* | 437 | 2 | C->T | CCC=>TTC | P=>F | + | + | + |
| *nad4* | 467 | 2 | C->T | ACT=>ATT | T=>I | - | + | + |
| *nad4* | 511 | 1 | C->T | CAT=>TAT | H=>Y | - | + | + |
| *nad4* | 524 | 2 | C->T | CCA=>CTA | P=>L | - | + | + |
| *nad4* | 533 | 2 | C->T | TCA=>TTA | S=>L | - | + | + |
| *nad4* | 536 | 2 | C->T | CCC=>CTC | P=>L | - | + | + |
| *nad4* | 547 | 1 | C->T | CCT=>TTT | P=>F | - | + | + |
| *nad4* | 548 | 2 | C->T | CCT=>TTT | P=>F | - | + | + |
| *nad4* | 554 | 2 | C->T | CCA=>CTA | P=>L | - | + | + |
| *nad4* | 557 | 2 | C->T | CCA=>CTA | P=>L | - | + | + |
| *nad4* | 566 | 2 | C->T | CCG=>CTG | P=>L | - | + | + |
| *nad4* | 569 | 2 | C->T | TCG=>TTG | S=>L | - | + | + |
| *nad4* | 574 | 1 | C->T | CTC=>TTC | L=>F | + | - | - |
| *nad4* | 578 | 2 | C->T | TCC=>TTC | S=>F | - | + | - |
| *nad4* | 599 | 2 | C->T | TCG=>TTG | S=>L | + | + | + |
| *nad4* | 611 | 2 | C->T | TCA=>TTA | S=>L | - | + | + |
| *nad4* | 623 | 2 | C->T | TCC=>TTC | S=>F | + | + | + |
| *nad4* | 644 | 2 | C->T | TCT=>TTT | S=>F | - | + | + |
| *nad4* | 647 | 2 | C->T | CCA=>CTA | P=>L | - | + | + |
| *nad4* | 658 | 1 | C->T | CCT=>TTT | P=>F | - | + | + |
| *nad4* | 659 | 2 | C->T | CCT=>TTT | P=>F | - | + | + |
| *nad4* | 661 | 1 | C->T | CCC=>TTC | P=>F | - | + | + |
| *nad4* | 662 | 2 | C->T | CCC=>TTC | P=>F | - | + | + |
| *nad4* | 667 | 1 | C->T | CCT=>TCT | P=>S | - | + | + |
| *nad4* | 671 | 2 | C->T | TCC=>TTC | S=>F | - | + | + |
| *nad4* | 706 | 1 | C->T | CGG=>TGG | R=>W | + | + | + |
| *nad4* | 755 | 2 | C->T | TCG=>TTG | S=>L | + | + | + |
| *nad4* | 767 | 2 | C->T | CCT=>CTT | P=>L | - | + | + |
| *nad4* | 770 | 2 | C->T | TCA=>TTA | S=>L | + | + | + |
| *nad4* | 776 | 2 | C->T | TCG=>TTG | S=>L | - | + | + |
| *nad4* | 794 | 2 | C->T | TCG=>TTG | S=>L | - | + | + |
| *nad4* | 832 | 1 | C->T | CGC=>TGC | R=>C | + | + | + |
| *nad4* | 835 | 1 | C->T | CCC=>TTC | P=>F | - | + | + |
| *nad4* | 836 | 2 | C->T | CCC=>TTC | P=>F | + | + | + |
| *nad4* | 883 | 1 | C->T | CCC=>TCC | P=>S | + | + | + |
| *nad4* | 887 | 2 | C->T | CCG=>CTG | P=>L | - | + | + |
| *nad4* | 911 | 2 | C->T | CCT=>CTT | P=>L | + | + | + |
| *nad4* | 928 | 1 | C->T | CAC=>TAC | H=>Y | + | + | + |
| *nad4* | 934 | 1 | C->T | CCA=>TCA | P=>S | + | + | + |
| *nad4* | 947 | 2 | C->T | ACG=>ATG | T=>M | - | + | + |
| *nad4* | 952 | 1 | C->T | CCT=>TCT | P=>S | + | - | - |
| *nad4* | 953 | 2 | C->T | CCT=>CTT | P=>L | - | + | + |
| *nad4* | 977 | 2 | C->T | CCG=>CTG | P=>L | - | + | + |
| *nad4* | 1010 | 2 | C->T | TCG=>TTG | S=>L | + | + | + |
| *nad4* | 1028 | 2 | C->T | CCG=>CTG | P=>L | + | + | + |
| *nad4* | 1033 | 1 | C->T | CCT=>TCT | P=>S | - | + | + |
| *nad4* | 1036 | 1 | C->T | CCA=>TCA | P=>S | - | + | + |
| *nad4* | 1043 | 2 | C->T | CCT=>CTT | P=>L | - | + | + |
| *nad4* | 1046 | 2 | C->T | TCT=>TTT | S=>F | - | + | + |
| *nad4* | 1049 | 2 | C->T | CCA=>CTA | P=>L | - | + | + |
| *nad4* | 1064 | 2 | C->T | CCG=>CTG | P=>L | - | + | + |
| *nad4* | 1088 | 2 | C->T | CCC=>CTC | P=>L | + | + | + |
| *nad4* | 1091 | 2 | C->T | GCT=>GTT | A=>V | - | + | + |
| *nad4* | 1109 | 2 | C->T | TCA=>TTA | S=>L | - | + | + |
| *nad4* | 1129 | 1 | C->T | CCC=>TTC | P=>F | - | + | + |
| *nad4* | 1130 | 2 | C->T | CCC=>TTC | P=>F | - | + | + |
| *nad4* | 1132 | 1 | C->T | CCT=>TCT | P=>S | - | + | + |
| *nad4* | 1142 | 2 | C->T | TCC=>TTC | S=>F | - | + | + |
| *nad4* | 1148 | 2 | C->T | TCT=>TTT | S=>F | - | + | + |
| *nad4* | 1172 | 2 | C->T | TCA=>TTA | S=>L | + | + | + |
| *nad4* | 1190 | 2 | C->T | TCT=>TTT | S=>L | + | + | + |
| *nad4* | 1205 | 2 | C->T | CCC=>CTC | P=>L | + | + | + |
| *nad4* | 1285 | 1 | C->T | CCC=>TCC | P=>S | + | + | + |
| *nad4* | 1307 | 2 | C->T | GCG=>GTG | A=>V | + | + | + |
| *nad4* | 1312 | 1 | C->T | CTC=>TTC | L=>F | + | + | - |
| *nad4* | 1354 | 1 | C->T | CCA=>TTA | P=>L | + | - | - |
| *nad4* | 1355 | 2 | C->T | CCA=>TTA | P=>L | + | + | + |
| *nad4* | 1373 | 2 | C->T | ACG=>ATG | L=>M | + | - | - |
| *nad4* | 1387 | 1 | C->T | CTT=>TTT | L=>F | + | + | + |
| *nad4* | 1403 | 2 | C->T | GCC=>GTC | A=>V | - | + | + |
| *nad4* | 1405 | 1 | C->T | CGG=>TGG | R=>W | - | + | - |
| *nad4* | 1417 | 1 | C->T | CAC=>TAC | H=>Y | - | + | - |
| *nad4* | 1433 | 2 | C->T | CCG=>CTG | P=>L | + | + | - |
| *nad4* | 1438 | 1 | C->T | CGT=>TGT | R=>C | - | + | - |
| *nad4* | 1450 | 1 | C->T | CCC=>TCC | P=>S | - | + | + |
| *nad4* | 1483 | 1 | C->T | CAU=>UAU | H=>Y | - | - | + |
| *nad4* | 1486 | 1 | C->T | CGA=>TGA | R=>X | - | + | + |
| *nad5* | 8 | 2 | C->T | TCA=>TTA | S=>L | + | + | + |
| *nad5* | 17 | 2 | C->T | GCA=>GTA | A=>V | - | + | + |
| *nad5* | 23 | 2 | C->T | TCG=>TTG | S=>L | - | + | + |
| *nad5* | 32 | 2 | C->T | CCC=>CTC | P=>L | - | + | + |
| *nad5* | 40 | 1 | C->T | CCC=>TCC | P=>S | - | + | + |
| *nad5* | 68 | 2 | C->T | CCA=>CTA | P=>L | + | + | + |
| *nad5* | 118 | 1 | C->T | CCT=>TCT | P=>S | + | + | + |
| *nad5* | 125 | 2 | C->T | TCA=>TTA | S=>L | + | + | - |
| *nad5* | 134 | 2 | C->T | ACT=>ATT | T=>I | - | + | - |
| *nad5* | 140 | 2 | C->T | TCT=>TTT | S=>F | + | + | + |
| *nad5* | 142 | 1 | C->T | CAT=>TAT | H=>Y | - | + | + |
| *nad5* | 155 | 2 | C->T | CCG=>CTG | P=>L | + | + | + |
| *nad5* | 176 | 2 | C->T | CCA=>CTA | P=>L | - | + | + |
| *nad5* | 220 | 1 | C->T | CGG=>TGG | R=>W | + | + | + |
| *nad5* | 275 | 2 | C->T | GCA=>GTA | A=>V | + | + | - |
| *nad5* | 350 | 2 | C->T | TCA=>TTA | S=>L | + | + | + |
| *nad5* | 359 | 2 | C->T | TCC=>TTC | S=>F | + | + | + |
| *nad5* | 442 | 1 | C->T | CAT=>TAT | H=>Y | - | + | + |
| *nad5* | 553 | 1 | C->T | CGT=>TGT | R=>C | - | + | + |
| *nad5* | 563 | 2 | C->T | CCC=>CTC | P=>L | - | + | + |
| *nad5* | 581 | 2 | C->T | TCC=>TTC | S=>F | - | + | + |
| *nad5* | 598 | 1 | C->T | CGT=>TGT | R=>C | - | + | + |
| *nad5* | 673 | 1 | C->T | CGT=>TGT | R=>C | - | + | + |
| *nad5* | 680 | 2 | C->T | TCA=>TTA | S=>L | - | + | + |
| *nad5* | 685 | 1 | C->T | CCT=>TTT | P=>F | - | + | + |
| *nad5* | 686 | 2 | C->T | CCT=>TTT | P=>F | - | + | + |
| *nad5* | 730 | 1 | C->T | CGG=>TGG | R=>W | - | + | + |
| *nad5* | 791 | 2 | C->T | ACG=>ATG | T=>M | - | + | + |
| *nad5* | 794 | 2 | C->T | GCA=>GTA | A=>V | - | + | + |
| *nad5* | 809 | 2 | C->T | TCC=>TTC | S=>F | - | + | + |
| *nad5* | 833 | 2 | C->T | CCA=>CTA | P=>L | - | + | + |
| *nad5* | 836 | 2 | C->T | TCT=>TTT | S=>F | - | + | + |
| *nad5* | 844 | 1 | C->T | CCA=>TCA | P=>S | - | + | + |
| *nad5* | 896 | 2 | C->T | TCC=>TTC | S=>F | - | + | - |
| *nad5* | 929 | 2 | C->T | CCG=>CTG | P=>L | - | + | + |
| *nad5* | 949 | 1 | C->T | CCA=>TCA | P=>S | - | + | + |
| *nad5* | 955 | 1 | C->T | CGC=>TGC | R=>C | - | + | + |
| *nad5* | 979 | 1 | C->T | CTT=>TTT | L=>F | - | + | + |
| *nad5* | 994 | 1 | C->T | CCC=>TCC | P=>S | - | + | + |
| *nad5* | 1039 | 1 | C->T | CCC=>TTC | P=>F | - | + | + |
| *nad5* | 1040 | 2 | C->T | CCC=>TTC | P=>F | - | + | + |
| *nad5* | 1052 | 2 | C->T | CCA=>CTA | P=>L | - | + | + |
| *nad5* | 1058 | 2 | C->T | TCG=>TTG | S=>L | - | + | + |
| *nad5* | 1175 | 2 | C->T | CCA=>CTA | P=>L | - | + | + |
| *nad5* | 1223 | 2 | C->T | TCG=>TTG | S=>L | - | + | + |
| *nad5* | 1270 | 1 | C->T | CGG=>TGG | R=>W | - | + | + |
| *nad5* | 1285 | 1 | C->T | CCT=>TCT | P=>S | - | + | + |
| *nad5* | 1295 | 2 | C->T | TCC=>TTC | S=>F | - | + | + |
| *nad5* | 1303 | 1 | C->T | CAC=>TAC | H=>Y | - | + | + |
| *nad5* | 1319 | 2 | C->T | TCG=>TTG | S=>L | - | + | + |
| *nad5* | 1340 | 2 | C->T | GCA=>GTA | A=>V | - | + | + |
| *nad5* | 1351 | 1 | C->T | CCA=>TCA | P=>S | - | + | + |
| *nad5* | 1436 | 2 | C->T | TCC=>TTC | S=>F | - | + | + |
| *nad5* | 1484 | 2 | C->T | TCC=>TTC | S=>F | - | + | + |
| *nad5* | 1486 | 1 | C->T | CGG=>TGG | R=>W | - | + | + |
| *nad5* | 1495 | 1 | C->T | CCC=>TCC | P=>S | - | + | + |
| *nad5* | 1499 | 2 | C->T | CCT=>CTT | P=>L | - | + | + |
| *nad5* | 1502 | 2 | C->T | TCC=>TTC | S=>F | - | + | + |
| *nad5* | 1508 | 2 | C->T | CCA=>CTA | P=>L | - | + | + |
| *nad5* | 1559 | 2 | C->T | ACC=>ATC | T=>I | + | + | + |
| *nad5* | 1577 | 2 | C->T | CCG=>CTG | P=>L | + | + | + |
| *nad5* | 1661 | 2 | C->T | TCC=>TTC | S=>F | + | + | + |
| *nad5* | 1682 | 2 | C->T | TCT=>TTT | S=>F | - | + | + |
| *nad5* | 1702 | 1 | C->T | CCC=>TCC | P=>F | + | + | + |
| *nad5* | 1703 | 2 | C->T | CCC=>TTC | P=>F | - | + | + |
| *nad5* | 1742 | 2 | C->T | CCG=>CTG | P=>L | - | + | + |
| *nad5* | 1775 | 2 | C->T | TCA=>TTA | S=>L | + | + | + |
| *nad5* | 1895 | 2 | C->T | CCA=>CTA | P=>L | + | + | + |
| *nad5* | 1910 | 2 | C->T | CCC=>CTC | P=>L | + | + | + |
| *nad5* | 1912 | 1 | C->T | CTT=>TTT | L=>F | - | + | + |
| *nad5* | 1916 | 2 | C->T | GCG=>GTG | A=>V | - | + | + |
| *nad5* | 1927 | 1 | C->T | CGT=>TGT | R=>C | + | + | + |
| *nad5* | 1939 | 1 | C->T | CTT=>TTT | L=>F | - | + | - |
| *nad5* | 1948 | 1 | C->T | CCT=>TCT | P=>S | - | + | + |
| *nad5* | 1967 | 2 | C->T | TCG=>TTG | S=>L | + | + | + |
| *nad5* | 1979 | 2 | C->T | TCG=>TTG | S=>L | + | + | + |
| *nad5* | 2000 | 2 | C->T | CCC=>CTC | P=>L | + | - | - |
| *nad6* | 2 | 2 | C->T | ACG=>ATG | T=>M | + | - | + |
| *nad6* | 7 | 1 | C->T | CTT=>TTT | L=>F | - | + | - |
| *nad6* | 17 | 2 | C->T | TCG=>TTG | S=>L | - | + | + |
| *nad6* | 19 | 1 | C->T | CCG=>TCG | P=>S | + | + | + |
| *nad6* | 26 | 2 | C->T | CCT=>CTT | P=>L | + | + | + |
| *nad6* | 37 | 1 | C->T | CCC=>TCC | P=>S | - | + | + |
| *nad6* | 44 | 2 | C->T | TCG=>TTG | S=>L | - | + | + |
| *nad6* | 76 | 1 | C->T | CCC=>TCC | P=>S | - | + | + |
| *nad6* | 83 | 2 | C->T | CCG=>CTG | P=>L | - | + | + |
| *nad6* | 86 | 2 | C->T | TCC=>TTC | S=>F | - | + | + |
| *nad6* | 89 | 2 | C->T | TCC=>TTC | S=>F | - | + | + |
| *nad6* | 95 | 2 | C->T | CCA=>CTA | P=>L | - | + | + |
| *nad6* | 103 | 1 | C->T | CGC=>TGC | R=>C | - | + | + |
| *nad6* | 119 | 2 | C->T | TCA=>TTA | S=>L | - | + | + |
| *nad6* | 122 | 2 | C->T | CCC=>CTC | P=>L | - | + | + |
| *nad6* | 128 | 2 | C->T | TCG=>TTG | S=>L | - | + | + |
| *nad6* | 137 | 2 | C->T | CCT=>CTT | P=>L | - | + | + |
| *nad6* | 142 | 1 | C->T | CCC=>TTC | P=>F | - | + | + |
| *nad6* | 143 | 2 | C->T | CCC=>TTC | P=>F | - | + | + |
| *nad6* | 145 | 1 | C->T | CCC=>TTC | P=>F | - | + | + |
| *nad6* | 146 | 2 | C->T | CCC=>TTC | P=>F | - | + | + |
| *nad6* | 158 | 2 | C->T | TCC=>TTC | S=>F | - | + | + |
| *nad6* | 161 | 2 | C->T | CCG=>CTG | P=>L | - | + | + |
| *nad6* | 173 | 2 | C->T | ACA=>ATA | T=>I | - | + | + |
| *nad6* | 191 | 2 | C->T | TCA=>TTA | S=>L | - | + | + |
| *nad6* | 194 | 2 | C->T | TCC=>TTC | S=>F | - | + | + |
| *nad6* | 199 | 1 | C->T | CCC=>TTC | P=>F | - | + | + |
| *nad6* | 200 | 2 | C->T | CCC=>TTC | P=>F | - | + | + |
| *nad6* | 215 | 2 | C->T | TCG=>TTG | S=>L | - | + | - |
| *nad6* | 251 | 2 | C->T | TCG=>TTG | S=>L | - | + | + |
| *nad6* | 260 | 2 | C->T | CCA=>CTA | P=>L | - | + | + |
| *nad6* | 289 | 1 | C->T | CTT=>TTT | L=>F | - | + | + |
| *nad6* | 292 | 1 | C->T | CGG=>TGG | R=>W | - | + | + |
| *nad6* | 305 | 2 | C->T | TCC=>TTC | S=>F | - | + | + |
| *nad6* | 307 | 1 | C->T | CCC=>TTC | P=>F | - | + | + |
| *nad6* | 308 | 2 | C->T | CCC=>TTC | P=>F | - | + | + |
| *nad6* | 314 | 2 | C->T | CCA=>CTA | P=>L | - | + | + |
| *nad6* | 325 | 1 | C->T | CAC=>TAC | H=>Y | - | + | - |
| *nad6* | 379 | 1 | C->T | CAT=>TAT | H=>Y | - | + | + |
| *nad6* | 410 | 2 | C->T | TCG=>TTG | S=>L | - | + | + |
| *nad6* | 419 | 2 | C->T | TCG=>TTG | S=>L | - | + | + |
| *nad6* | 428 | 2 | C->T | TCA=>TTA | S=>L | - | + | + |
| *nad6* | 433 | 1 | C->T | CAT=>TAT | H=>Y | - | + | + |
| *nad6* | 446 | 2 | C->T | TCC=>TTC | S=>F | - | + | + |
| *nad6* | 451 | 1 | C->T | CGG=>TGG | R=>W | - | + | + |
| *nad6* | 470 | 2 | C->T | CCC=>CTC | P=>L | - | + | + |
| *nad6* | 476 | 2 | C->T | CCA=>CTA | P=>L | - | + | + |
| *nad6* | 479 | 2 | C->T | TCA=>TTA | S=>L | - | + | + |
| *nad6* | 500 | 2 | C->T | ACA=>ATA | T=>I | - | + | + |
| *nad6* | 569 | 2 | C->T | TCC=>TTC | S=>F | - | + | - |
| *nad7* | 32 | 2 | C->T | TCC=>TTC | S=>F | + | + | + |
| *nad7* | 38 | 2 | C->T | TCG=>TTG | S=>L | + | + | + |
| *nad7* | 44 | 2 | C->T | TCT=>TTT | S=>F | + | + | + |
| *nad7* | 77 | 2 | C->T | CCA=>CTA | P=>L | + | + | + |
| *nad7* | 83 | 2 | C->T | TCA=>TTA | S=>L | + | + | + |
| *nad7* | 89 | 2 | C->T | TCG=>TTG | S=>L | + | + | + |
| *nad7* | 137 | 2 | C->T | TCA=>TTA | S=>L | + | + | + |
| *nad7* | 196 | 1 | C->T | CAT=>TAT | H=>Y | + | + | + |
| *nad7* | 214 | 1 | C->T | CAC=>TAC | H=>Y | + | + | + |
| *nad7* | 244 | 1 | C->T | CAT=>TAT | H=>Y | + | + | + |
| *nad7* | 251 | 2 | C->T | TCA=>TTA | S=>L | + | + | + |
| *nad7* | 316 | 1 | C->T | CGC=>TGC | R=>C | + | + | + |
| *nad7* | 353 | 2 | C->T | TCA=>TTA | S=>L | + | + | + |
| *nad7* | 425 | 2 | C->T | TCG=>TTG | S=>L | + | + | + |
| *nad7* | 445 | 1 | C->T | CCG=>TCG | P=>S | + | + | + |
| *nad7* | 566 | 2 | C->T | TCA=>TTA | S=>L | - | + | + |
| *nad7* | 595 | 1 | C->T | CGG=>TGG | R=>W | - | + | + |
| *nad7* | 671 | 2 | C->T | CCA=>CTA | P=>L | - | + | + |
| *nad7* | 715 | 1 | C->T | CAC=>TAC | H=>Y | - | + | + |
| *nad7* | 722 | 2 | C->T | GCC=>GTC | A=>V | - | + | + |
| *nad7* | 836 | 2 | C->T | CCT=>CTT | P=>L | - | + | + |
| *nad7* | 913 | 1 | C->T | CCC=>TCC | P=>S | - | + | + |
| *nad7* | 926 | 2 | C->T | TCA=>TTA | S=>L | - | + | + |
| *nad7* | 1057 | 1 | C->T | CGT=>TGT | R=>C | + | + | + |
| *nad7* | 1064 | 2 | C->T | ACA=>ATA | T=>I | + | + | + |
| *nad7* | 1088 | 2 | C->T | TCA=>TTA | S=>L | + | + | - |
| *nad7* | 1097 | 2 | C->T | CCT=>CTT | P=>L | - | + | + |
| *nad7* | 1103 | 2 | C->T | TCT=>TTT | S=>F | - | + | - |
| *nad7* | 1124 | 2 | C->T | CCA=>CTA | P=>L | + | + | - |
| *nad7* | 1142 | 2 | C->T | ACC=>ATC | T=>I | + | + | + |
| *nad7* | 1145 | 2 | C->T | ACC=>ATC | T=>I | + | + | + |
| *nad9* | 2 | 2 | C->T | ACG=>ATG | T=>M | + | + | + |
| *nad9* | 28 | 1 | C->T | CCG=>TCG | P=>S | - | + | + |
| *nad9* | 29 | 2 | C->T | CCG=>CTG | P=>L | + | - | - |
| *nad9* | 41 | 2 | C->T | TCA=>TTA | S=>L | + | + | - |
| *nad9* | 92 | 2 | C->T | TCA=>TTA | S=>L | + | - | - |
| *nad9* | 113 | 2 | C->T | CCA=>CTA | P=>L | + | + | + |
| *nad9* | 134 | 2 | C->T | CCG=>CTG | P=>L | + | + | + |
| *nad9* | 158 | 2 | C->T | TCC=>TTC | S=>F | - | + | + |
| *nad9* | 178 | 1 | C->T | CGC=>TGC | R=>C | - | + | + |
| *nad9* | 218 | 2 | C->T | GCG=>GTG | A=>V | - | + | + |
| *nad9* | 223 | 1 | C->T | CAT=>TAT | H=>Y | - | + | + |
| *nad9* | 230 | 2 | C->T | TCA=>TTA | S=>L | - | + | + |
| *nad9* | 233 | 2 | C->T | CCA=>CTA | P=>L | - | + | + |
| *nad9* | 250 | 1 | C->T | CCA=>TCA | P=>S | - | + | + |
| *nad9* | 298 | 1 | C->T | CCG=>TCG | P=>S | - | + | + |
| *nad9* | 311 | 2 | C->T | CCA=>CTA | P=>L | + | + | + |
| *nad9* | 328 | 1 | C->T | CGG=>TGG | R=>W | - | + | + |
| *nad9* | 356 | 2 | C->T | TCC=>TTC | S=>F | - | + | + |
| *nad9* | 368 | 2 | C->T | TCC=>TTC | S=>F | - | + | + |
| *nad9* | 398 | 2 | C->T | TCA=>TTA | S=>L | - | + | + |
| *nad9* | 406 | 1 | C->T | CAC=>TAC | H=>Y | - | + | + |
| *nad9* | 413 | 2 | C->T | TCC=>TTC | S=>F | - | + | + |
| *nad9* | 440 | 2 | C->T | TCC=>TTC | S=>F | - | + | + |
| *nad9* | 478 | 1 | C->T | CCG=>TCG | P=>S | + | - | - |
| *nad9* | 524 | 2 | C->T | TCC=>TTC | S=>F | + | + | + |
| *nad9* | 533 | 2 | C->T | TCC=>TTC | S=>F | + | + | + |
| *rpl5* | 34 | 1 | C->T | CAC=>TAC | H=>Y | - | + | + |
| *rpl5* | 59 | 2 | C->T | CCG=>CTG | P=>L | + | + | + |
| *rpl5* | 65 | 2 | C->T | CCA=>CTA | P=>L | - | + | + |
| *rpl5* | 71 | 2 | C->T | CCG=>CTG | P=>L | + | + | + |
| *rpl5* | 76 | 1 | C->T | CAC=>TAC | H=>Y | - | + | + |
| *rpl5* | 89 | 2 | C->T | ACG=>ATG | T=>M | - | + | + |
| *rpl5* | 104 | 2 | C->T | TCG=>TTG | S=>L | - | + | + |
| *rpl5* | 166 | 1 | C->T | CCG=>UCG | P=>S | - | - | + |
| *rpl5* | 167 | 2 | C->T | CCG=>CTG | P=>L | + | + | - |
| *rpl5* | 169 | 1 | C->T | CGC=>TGC | R=>C | + | + | + |
| *rpl5* | 209 | 2 | C->T | TCT=>TTT | S=>F | - | + | - |
| *rpl5* | 269 | 2 | C->T | TCC=>TTC | S=>F | - | + | - |
| *rpl5* | 311 | 2 | C->T | TCC=>TTC | S=>F | + | + | + |
| *rpl5* | 326 | 2 | C->T | TCG=>TTG | S=>L | + | + | + |
| *rpl5* | 349 | 1 | C->T | CTT=>TTT | L=>F | - | + | - |
| *rpl5* | 385 | 1 | C->T | CCG=>TCG | P=>S | - | + | + |
| *rpl5* | 443 | 2 | C->T | TCC=>TTC | S=>F | - | + | + |
| *rpl5* | 476 | 2 | C->T | GCA=>GTA | A=>V | + | + | + |
| *rpl5* | 481 | 1 | C->T | CCG=>TCG | P=>S | + | + | + |
| *rpl5* | 509 | 2 | C->T | CCA=>CTA | P=>L | + | + | + |
| *rpl5* | 512 | 2 | C->T | TCG=>TTG | S=>L | + | + | + |
| *rpl5* | 527 | 2 | C->T | TCG=>TTG | S=>L | - | + | + |
| *rpl5* | 550 | 1 | C->T | CTC=>TTC | L=>F | + | - | - |
| *rpl16* | 38 | 2 | C->T | CCG=>CTG | P=>L | + | - | - |
| *rpl16* | 137 | 2 | C->T | GCG=>GTG | A=>V | - | + | - |
| *rpl16* | 185 | 2 | C->T | CCA=>CTA | P=>L | - | + | - |
| *rpl16* | 217 | 1 | C->T | CCA=>TCA | P=>S | + | - | - |
| *rpl16* | 244 | 1 | C->T | CGG=>TGG | R=>W | + | - | - |
| *rpl16* | 289 | 1 | C->T | CTT=>TTT | L=>F | + | - | - |
| *rpl16* | 385 | 1 | C->T | CGA=>TGA | R=>U | + | - | - |
| *rpl16* | 478 | 1 | C->T | CCT=>TCT | P=>S | + | - | - |
| *rpl16* | 500 | 2 | C->T | GCG=>GTG | A=>V | - | + | - |
| *rpl16* | 518 | 2 | C->T | CCA=>CTA | P=>L | - | + | - |
| *rpl16* | 586 | 1 | C->T | CGT=>TGT | R=>C | - | + | - |
| *rps3* | 58 | 1 | C->T | CCA=>TCA | P=>S | - | + | + |
| *rps3* | 64 | 1 | C->T | CGG=>TGG | R=>W | + | + | + |
| *rps3* | 82 | 1 | C->T | CAT=>TAT | H=>Y | + | + | + |
| *rps3* | 92 | 2 | C->T | CCG=>CTG | P=>L | + | + | + |
| *rps3* | 113 | 2 | C->T | CCT=>CTT | P=>L | + | + | + |
| *rps3* | 185 | 2 | C->T | CCT=>CTT | P=>L | + | - | - |
| *rps3* | 194 | 2 | C->T | TCT=>TTT | S=>F | - | + | + |
| *rps3* | 217 | 1 | C->T | CTC=>TTC | L=>F | - | + | + |
| *rps3* | 224 | 2 | C->T | CCT=>CTT | P=>L | + | + | + |
| *rps3* | 238 | 1 | C->T | CAA=>TAA | Q=>U | + | - | - |
| *rps3* | 293 | 2 | C->T | GCU=>GUU | A=>V | - | - | + |
| *rps3* | 301 | 1 | C->T | CTC=>TTC | L=>F | - | + | - |
| *rps3* | 340 | 1 | C->T | CCG=>TCG | P=>S | - | + | - |
| *rps3* | 637 | 1 | C->T | CTC=>TTC | L=>F | - | + | + |
| *rps3* | 640 | 1 | C->T | CTC=>TTC | L=>F | - | + | + |
| *rps3* | 695 | 2 | C->T | CCG=>CTG | P=>L | + | - | - |
| *rps3* | 767 | 2 | C->T | TCC=>TTC | S=>F | + | + | - |
| *rps3* | 770 | 2 | C->T | TCT=>TTT | S=>F | + | + | - |
| *rps3* | 788 | 2 | C->T | ACT=>ATT | T=>I | - | + | - |
| *rps3* | 812 | 2 | C->T | GCC=>GTC | A=>V | - | + | - |
| *rps3* | 836 | 2 | C->T | TCC=>TTC | S=>F | + | - | + |
| *rps3* | 887 | 2 | C->T | CCA=>CTA | P=>L | - | + | - |
| *rps3* | 919 | 1 | C->T | CTC=>TTC | L=>F | - | + | - |
| *rps3* | 928 | 1 | C->T | CCA=>TCA | P=>S | - | + | - |
| *rps3* | 1039 | 1 | C->T | CTC=>TTC | L=>F | - | + | - |
| *rps3* | 1046 | 2 | C->T | TCT=>TTT | S=>F | - | + | - |
| *rps3* | 1064 | 2 | C->T | TCC=>TTC | S=>F | - | + | + |
| *rps3* | 1106 | 2 | C->T | TCT=>TTT | S=>F | - | + | + |
| *rps3* | 1250 | 2 | C->T | CCG=>CTG | P=>L | + | + | + |
| *rps3* | 1289 | 2 | C->T | TCG=>TTG | S=>L | - | + | + |
| *rps3* | 1322 | 2 | C->T | TCT=>TTT | S=>F | + | - | - |
| *rps3* | 1346 | 2 | C->T | TCC=>TTC | S=>F | + | - | - |
| *rps3* | 1391 | 2 | C->T | CCA=>CTA | P=>L | + | + | + |
| *rps3* | 1465 | 1 | C->T | CGG=>TGG | R=>W | + | - | - |
| *rps3* | 1474 | 1 | C->T | CCA=>TTA | P=>L | + | - | - |
| *rps3* | 1475 | 2 | C->T | CCA=>TTA | P=>L | + | - | - |
| *rps3* | 1519 | 1 | C->T | CCG=>TCG | P=>S | + | + | + |
| *rps3* | 1553 | 2 | C->T | ACT=>ATT | T=>I | + | - | - |
| *rps3* | 1589 | 2 | C->T | GCA=>GTA | A=>V | - | + | + |
| *rps3* | 1594 | 1 | C->T | CGC=>TGC | R=>C | + | - | - |
| *rps3* | 1626 | 0 | C->T | GTC=>GTT | V=>V | + | - | - |
| *rps3* | 1666 | 1 | C->T | CCA=>TCA | P=>S | + | + | + |
| *rps3* | 1672 | 1 | C->T | CCA=>TCA | P=>S | - | + | - |
| *rps4* | 20 | 2 | C->T | TCT=>TTT | S=>F | + | + | + |
| *rps4* | 28 | 1 | C->T | CGT=>TGT | R=>C | + | + | + |
| *rps4* | 41 | 2 | C->T | CCG=>CTG | P=>L | + | + | - |
| *rps4* | 131 | 2 | C->T | CCG=>CTG | P=>L | + | - | - |
| *rps4* | 167 | 2 | C->T | TCA=>TTA | S=>L | + | + | - |
| *rps4* | 191 | 2 | C->T | CCC=>CTC | P=>L | - | + | + |
| *rps4* | 196 | 1 | C->T | CAT=>TAT | H=>Y | + | + | - |
| *rps4* | 260 | 2 | C->T | TCA=>TTA | S=>L | + | + | - |
| *rps4* | 269 | 2 | C->T | CCA=>CTA | P=>L | + | + | - |
| *rps4* | 281 | 2 | C->T | TCG=>TTG | S=>L | + | + | - |
| *rps4* | 310 | 1 | C->T | CGC=>TGC | R=>C | + | + | - |
| *rps4* | 358 | 1 | C->T | CGT=>TGT | R=>C | - | + | + |
| *rps4* | 413 | 2 | C->T | CCA=>CTA | P=>L | + | + | + |
| *rps4* | 418 | 1 | C->T | CCT=>TCT | P=>S | + | + | + |
| *rps4* | 464 | 2 | C->T | TCC=>TTC | S=>F | + | + | + |
| *rps4* | 523 | 1 | C->T | CGA=>TGA | R=>U | + | - | - |
| *rps4* | 544 | 1 | C->T | CGC=>TGC | R=>C | + | - | - |
| *rps4* | 622 | 1 | C->T | CAA=>TAA | Q=>U | + | - | - |
| *rps4* | 649 | 1 | C->T | CCT=>TTT | P=>F | - | + | + |
| *rps4* | 650 | 2 | C->T | CCT=>TTT | P=>F | - | + | + |
| *rps4* | 713 | 2 | C->T | TCG=>TTG | S=>L | + | + | + |
| *rps4* | 722 | 2 | C->T | TCC=>TTC | S=>F | - | + | - |
| *rps4* | 745 | 1 | C->T | CCC=>TTC | P=>F | + | - | - |
| *rps4* | 746 | 2 | C->T | CCC=>TTC | P=>F | + | - | - |
| *rps4* | 829 | 1 | C->T | CCC=>TCC | P=>S | - | + | - |
| *rps4* | 842 | 2 | C->T | TCT=>TTT | S=>F | - | + | - |
| *rps4* | 848 | 2 | C->T | TCC=>TTC | S=>F | + | - | - |
| *rps4* | 859 | 1 | C->T | CCC=>TCC | P=>S | - | + | - |
| *rps4* | 890 | 2 | C->T | CCC=>CTC | P=>L | - | + | - |
| *rps4* | 967 | 1 | C->T | CAT=>TAT | H=>Y | + | + | - |
| *rps4* | 977 | 2 | C->T | CCG=>CTG | P=>L | + | + | - |
| *rps4* | 992 | 2 | C->T | TCT=>TTT | S=>F | + | + | - |
| *rps4* | 1043 | 2 | C->T | CCG=>CTG | P=>L | + | + | - |
| *rps4* | 1052 | 2 | C->T | CCC=>CTC | P=>L | - | + | - |
| *rps12* | 25 | 1 | C->T | CGT=>TGT | R=>C | + | - | - |
| *rps12* | 100 | 1 | C->T | CGC=>TGC | R=>C | + | + | + |
| *rps12* | 103 | 1 | C->T | CCG=>TCG | P=>S | + | - | - |
| *rps12* | 104 | 2 | C->T | CCG=>CTG | P=>L | + | + | - |
| *rps12* | 146 | 2 | C->T | CCA=>CTA | P=>L | + | + | + |
| *rps12* | 220 | 1 | C->T | CCA=>TTA | P=>L | + | - | - |
| *rps12* | 221 | 2 | C->T | CCA=>TTA | P=>L | + | + | + |
| *rps12* | 269 | 2 | C->T | CCG=>CTG | P=>L | + | + | + |
| *rps12* | 284 | 2 | C->T | TCC=>TTC | S=>F | + | + | + |
| *rps12* | 289 | 1 | C->T | CGT=>TGT | R=>C | + | + | + |
| *rps12* | 311 | 2 | C->T | TCG=>TTG | S=>L | + | + | + |
| *rps12* | 371 | 2 | C->T | TCG=>TTG | S=>L | + | - | - |
| *rps13* | 19 | 1 | C->T | CCA=>TCA | P=>S | - | + | + |
| *rps13* | 20 | 2 | C->T | CCA=>CTA | P=>L | + | - | - |
| *rps13* | 62 | 2 | C->T | CCA=>CTA | P=>L | + | + | + |
| *rps13* | 98 | 2 | C->T | ACU=>AUU | T=>I | - | + | + |
| *rps13* | 100 | 1 | C->T | CAG=>TAG | Q=>U | + | - | - |
| *rps13* | 106 | 1 | C->T | CGT=>TGT | R=>C | + | + | + |
| *rps13* | 112 | 1 | C->T | CGA=>TGA | R=>U | + | - | - |
| *rps13* | 149 | 2 | C->T | TCG=>TTG | S=>L | + | + | + |
| *rps13* | 239 | 2 | C->T | GCG=>GTG | A=>V | + | - | - |
| *rps13* | 259 | 1 | C->T | CCT=>TTT | P=>F | + | + | + |
| *rps13* | 260 | 2 | C->T | CCT=>TTT | P=>F | + | - | - |
| *rps13* | 268 | 1 | C->T | CGT=>TGT | R=>C | + | + | + |
| *rps13* | 322 | 1 | C->T | CAT=>TAT | H=>Y | + | - | - |
| *rps13* | 353 | 2 | C->T | TCG=>TTG | S=>L | + | - | - |
| *rps19* | 43 | 1 | C->T | CTC=>TTC | L=>F | + | + | + |
| *rps19* | 112 | 1 | C->T | CCT=>TCT | P=>S | + | + | + |
| *rps19* | 115 | 1 | C->T | CCC=>TCC | P=>S | + | + | + |
| *rps19* | 122 | 2 | C->T | CCG=>CTG | P=>L | - | + | + |
| *rps19* | 139 | 1 | C->T | CGC=>TGC | R=>C | - | + | + |
| *rps19* | 143 | 2 | C->T | TCC=>TTC | S=>F | + | + | - |
| *rps19* | 169 | 1 | C->T | CCC=>TCC | P=>S | + | + | + |
| *rps19* | 170 | 2 | C->T | CCC=>CTC | P=>L | + | + | + |
| *rps19* | 212 | 2 | C->T | TCC=>TTC | S=>F | + | + | + |
| *rps19* | 262 | 1 | C->T | CCG=>UCG | P=>S | - | - | + |
| *rps19* | 269 | 2 | C->T | ACC=>ATC | T=>I | - | + | - |
| *sdh4* | 15 | 0 | C->T | TTC=>TTT | F=>F | + | - | - |
| *sdh4* | 83 | 2 | C->T | ACA=>ATA | T=>I | - | + | + |
| *sdh4* | 157 | 1 | C->T | CTT=>TTT | L=>F | - | + | - |
| *sdh4* | 173 | 2 | C->T | TCC=>TTC | S=>F | + | - | - |
| *sdh4* | 188 | 2 | C->T | TCG=>TTG | S=>L | + | - | - |
| *sdh4* | 208 | 1 | C->T | CCC=>TTC | P=>F | - | + | - |
| *sdh4* | 209 | 2 | C->T | CCC=>TTC | P=>F | + | - | - |
| *sdh4* | 221 | 2 | C->T | TCG=>TTG | S=>L | + | + | - |
| *sdh4* | 233 | 2 | C->T | TCA=>TTA | S=>L | + | + | + |
| *sdh4* | 238 | 1 | C->T | CGG=>TGG | R=>W | - | + | + |
| *sdh4* | 251 | 2 | C->T | GCG=>GTG | A=>V | - | + | + |
| *sdh4* | 277 | 1 | C->T | CAC=>TAC | H=>Y | + | + | + |
| *sdh4* | 326 | 2 | C->T | TCG=>TTG | S=>L | - | + | + |
| *sdh4* | 359 | 2 | C->T | CCG=>CTG | P=>L | - | + | - |
| *sdh4* | 365 | 2 | C->T | CCA=>CTA | P=>L | - | + | - |
| *sdh4* | 379 | 1 | C->T | CAC=>TAC | H=>Y | + | - | - |
| *sdh4* | 382 | 1 | C->T | CAC=>TAC | H=>Y | - | + | - |
| **Total** |  |  |  |  |  | **581** | **1102** | **953** |
