# Supplementary figures and images for: The complete mitochondrial genome of Taxus cuspidata (Taxaceae): eight protein-coding genes have transferred to the nuclear genome
Source: BMC Evol Biol. 2020 Jan 20;20:10. doi: 10.1186/s12862-020-1582-1 (PMC6971862; doi:10.1186/s12862-020-1582-1)

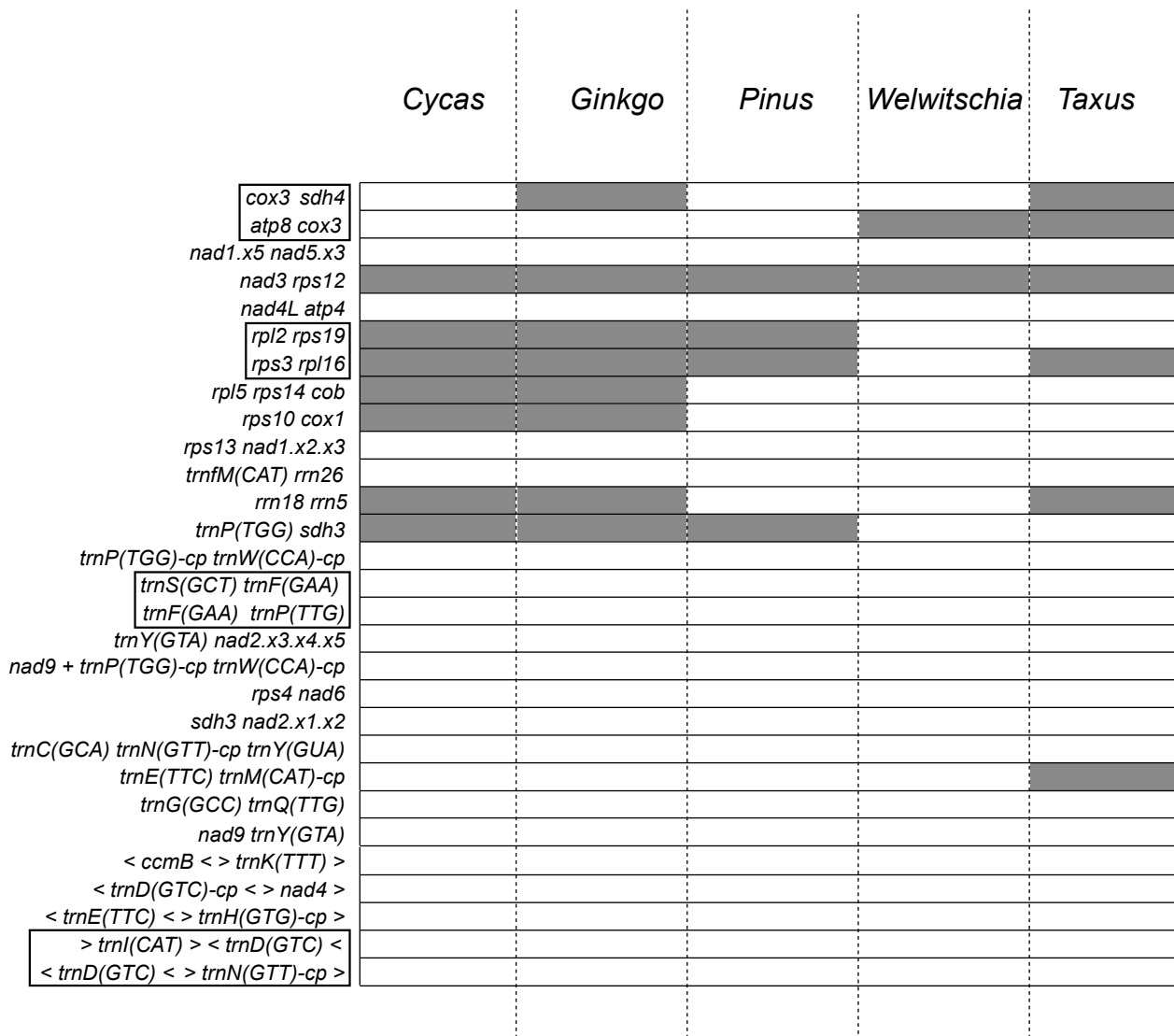

**Additional file 11: Figure S6. Mitochondrial gene clusters across gymnosperms.**

Supplement: Supplementary file 11 — Additional file 11: Figure S6. Mitochondrial gene clusters across gymnosperms. [file 12862_2020_1582_MOESM11_ESM.pdf]
