# Supplementary material for: The complete mitochondrial genome of Taxus cuspidata (Taxaceae): eight protein-coding genes have transferred to the nuclear genome
Source: BMC Evol Biol. 2020 Jan 20;20:10. doi: 10.1186/s12862-020-1582-1 (PMC6971862; doi:10.1186/s12862-020-1582-1)
Supplement: Supplementary file 12 — Additional file 12: Figure S7. (A) Total length of repeats in the mitogenomes of gymnosperms. The values in the bar indicate the number of repeat pairs. (B) Copy number of repeat pairs in each species. [file 12862_2020_1582_MOESM12_ESM.pdf]

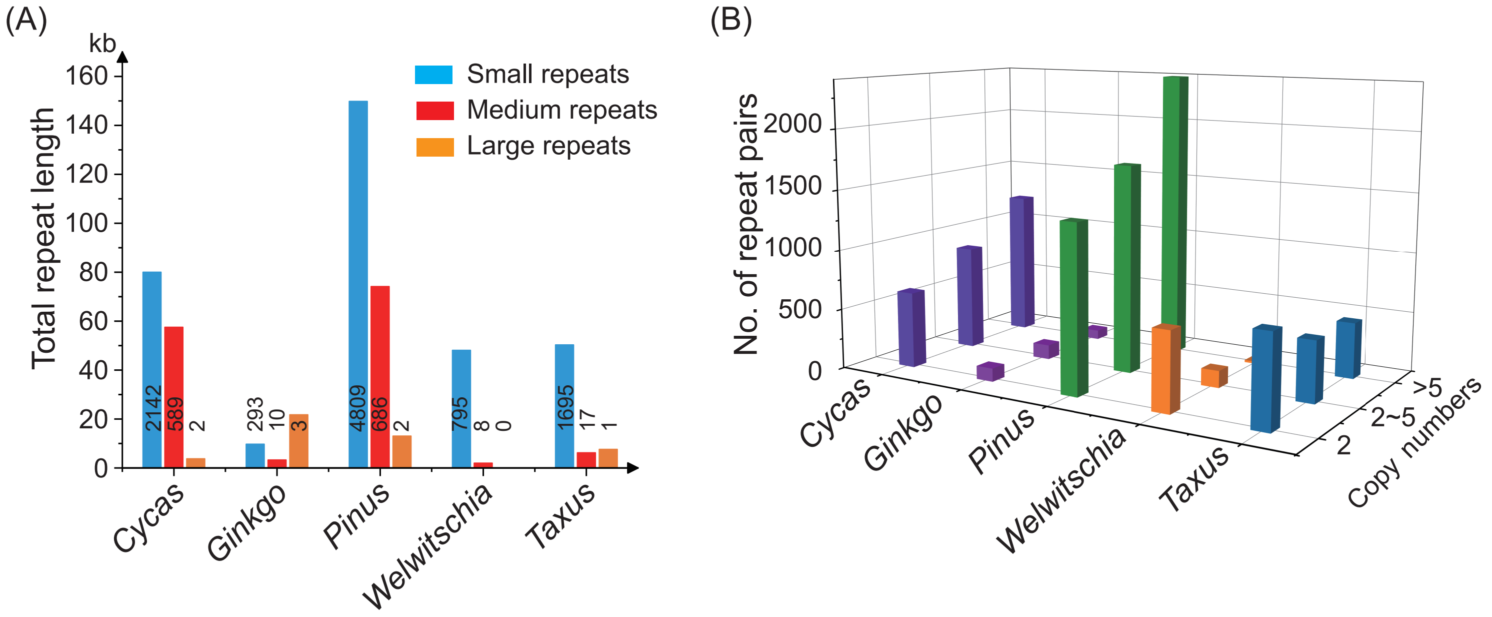

Additional file 12: Figure S7. (A) Total length of repeats in the mitogenomes of gymnosperms. The values in the bar indicate the number of repeat pairs. (B) Copy number of repeat pairs in each species.
