# Supplementary material for: The complete mitochondrial genome of Taxus cuspidata (Taxaceae): eight protein-coding genes have transferred to the nuclear genome
Source: BMC Evol Biol. 2020 Jan 20;20:10. doi: 10.1186/s12862-020-1582-1 (PMC6971862; doi:10.1186/s12862-020-1582-1)
Supplement: Supplementary file 13 — Additional file 13 Table S6. Nuclear-derived repetitive sequences in the sampled gymnosperm mitogenomes. [file 12862_2020_1582_MOESM13_ESM.docx]

**Additional file 13: Table S6**. Nuclear-derived repetitive sequences in the sampled gymnosperm mitogenomes.

|  | ***Cycas*** | ***Ginkgo*** | ***Pinus*** | ***Welwitschia*** | ***Taxus*** |
| --- | --- | --- | --- | --- | --- |
| *CIN4* |  | 42 | 593 |  |  |
| *Copia* | 2269 | 57 | 1402 | 190 | 78 |
| *Gypsy* |  | 84 | 1089 | 64 | 135 |
| *DNA transposons* |  |  | 213 | 565 | 528 |
| *Small RNA* | 1152 | 1744 | 2029 | 1738 | 2606 |
| Total | 3421 | 1927 | 5326 | 2557 | 3347 |
