# Supplementary material for: The complete mitochondrial genome of Taxus cuspidata (Taxaceae): eight protein-coding genes have transferred to the nuclear genome
Source: BMC Evol Biol. 2020 Jan 20;20:10. doi: 10.1186/s12862-020-1582-1 (PMC6971862; doi:10.1186/s12862-020-1582-1)
Supplement: Supplementary file 14 — Additional file 14: Figure S8. Synonymous (A) and nonsynonymous (B) sequence divergence in the conserved region of mitochondrial protein-coding genes in gymnosperms. Dots indicate mitochondrial protein-coding genes that have not transferred to the nucleus in all five gymnosperm species. Circles indicate the transferred genes in Taxus and Welwitschia and their homologous genes in Cycas, Ginkgo and Pinaceae. [file 12862_2020_1582_MOESM14_ESM.pdf]

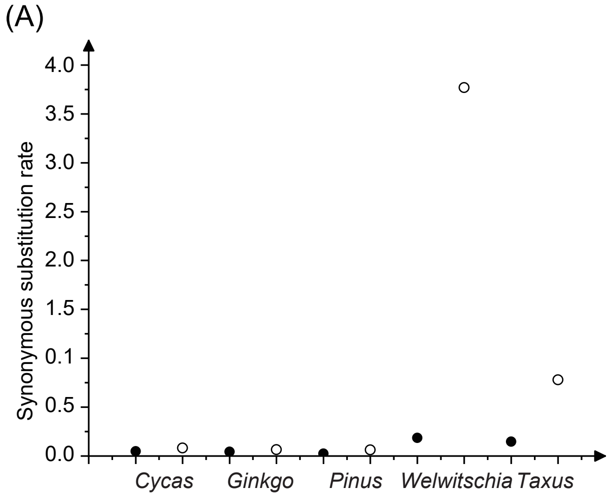

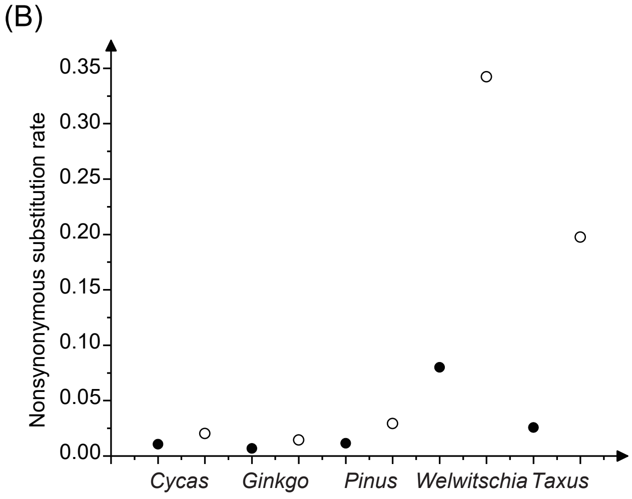

Additional file 14: Figure S8. Synonymous (A) and nonsynonymous (B) sequence divergence in the conserved region of mitochondrial protein-coding genes in gymnosperms. Dots indicate mitochondrial protein-coding genes that have not transferred to the nucleus in all five gymnosperm species. Circles indicate the transferred genes in *Taxus* and *Welwitschia* and their homologous genes in *Cycas*, *Ginkgo* and Pinaceae.
