# Supplementary material for: The complete mitochondrial genome of Taxus cuspidata (Taxaceae): eight protein-coding genes have transferred to the nuclear genome
Source: BMC Evol Biol. 2020 Jan 20;20:10. doi: 10.1186/s12862-020-1582-1 (PMC6971862; doi:10.1186/s12862-020-1582-1)
Supplement: Supplementary file 15 — Additional file 15: Table S7. Number of RNA editing sites predicted from PREP-Mt. [file 12862_2020_1582_MOESM15_ESM.docx]

**Additional file 15: Table S7.** Number of RNA editing sites predicted from PREP-Mt.

| **Gene** | ***Cycas*** | ***Ginkgo*** | ***Pinus*** | ***Welwitschia*** | ***Taxus*** |
| --- | --- | --- | --- | --- | --- |
| ***nad1*** | 48 | 59 | 27 | 4 | 47 |
| ***nad2*** | 54 | 70 | 50 | 3 | 65 |
| ***nad3*** | 29 | 37 | 25 | 3 | 18 |
| ***nad4*** | 91 | 101 | 82 | 1 | 89 |
| ***nad4L*** | 16 | 20 | 18 | 4 | 23 |
| ***nad5*** | 89 | 101 | 81 | 5 | 76 |
| ***nad6*** | 35 | 45 | 41 | 22 | 49 |
| ***nad7*** | 31 | 46 | 35 | 10 | 31 |
| ***nad9*** | 18 | 23 | 17 | 0 | 23 |
| ***sdh3*** | 28 | 25 | 23 |  |  |
| ***sdh4*** | 11 | 14 | 8 | 3 | 12 |
| ***cob*** | 53 | 6 | 53 | 5 | 50 |
| ***cox1*** | 63 | 5 | 74 | 2 | 61 |
| ***cox2*** | 21 | 31 | 15 | 2 | 25 |
| ***cox3*** | 29 | 40 | 34 | 1 | 16 |
| ***atp1*** | 42 | 22 | 3 | 4 | 39 |
| ***atp4*** | 17 | 24 | 20 | 10 | 20 |
| ***atp6*** | 48 | 58 | 50 | 0 | 36 |
| ***atp8*** | 14 | 17 | 19 | 6 | 14 |
| ***atp9*** | 12 | 16 | 10 | 0 | 15 |
| ***ccmB*** | 45 | 43 | 40 | 23 | 42 |
| ***ccmC*** | 43 | 43 | 50 | 10 | 37 |
| ***ccmFc*** | 34 | 38 | 38 | 16 | 40 |
| ***ccmFn*** | 48 | 57 | 58 | 16 | 72 |
| ***matR*** | 31 | 36 | 29 | 29 | 40 |
| ***mttB*** | 45 | 54 | 44 | 17 | 54 |
| ***rpl2*** | 24 | 28 | 22 |  |  |
| ***rpl5*** | 19 | 20 | 16 |  | 21 |
| ***rpl10*** | 3 | 15 | 12 | 6 |  |
| ***rpl16*** | 11 | 12 | 10 |  | 5 |
| ***rps1*** | 10 | 16 | 19 |  |  |
| ***rps2*** | 14 | 24 | 20 |  |  |
| ***rps3*** | 23 | 35 | 29 | 13 | 30 |
| ***rps4*** | 35 | 42 | 40 | 9 | 27 |
| ***rps7*** | 14 | 19 | 15 |  |  |
| ***rps10*** | 8 | 4 | 9 |  |  |
| ***rps11*** | 5 | 5 | 4 |  |  |
| ***rps12*** | 15 | 17 | 11 | 1 | 8 |
| ***rps13*** | 10 | 13 | 8 |  | 7 |
| ***rps14*** | 10 | 10 | 10 |  |  |
| ***rps19*** | 10 | 15 | 10 |  | 10 |
| **Total** | 1206 | 1306 | 1179 | 225 | 1102 |
