# Supplementary material for: The complete mitochondrial genome of Taxus cuspidata (Taxaceae): eight protein-coding genes have transferred to the nuclear genome
Source: BMC Evol Biol. 2020 Jan 20;20:10. doi: 10.1186/s12862-020-1582-1 (PMC6971862; doi:10.1186/s12862-020-1582-1)
Supplement: Supplementary file 16 — Additional file 16: Table S8. Comparison of the predicted (by PREP) and observed RNA editing sites in the sampled gymnosperm mitogenomes. [file 12862_2020_1582_MOESM16_ESM.docx]

**Additional file 16: Table S8.** Comparison of the predicted (by PREP) and observed RNA editing sites in the sampled gymnosperm mitogenomes.

| **Gene** | ***Cycas*^a^** | | | ***Ginkgo*^b^** | | | ***Welwitschia*^d^** | | | ***Pinus*** | ***Taxus*** | | |
| --- | --- | --- | --- | --- | --- | --- | --- | --- | --- | --- | --- | --- | --- |
|  | **Observed** | **Predicted** | **O-P** | **Observed** | **Predicted** | **O-P** | **Observed** | **Predicted** | **O-P** | **Predicted** | **Observed** | **Predicted** | **O-P** |
| ***atp1*** | 21^p^ | 15 | 6 | 7^p^ | 7 | 0 | 0 | 2 | -2 | 5 | 39 | 39 | 0 |
| ***atp4*** | 15 | 17 | -2 | 21^p^ | 20 | 1 | u | u | u | 22 | 24 | 20 | -2 |
| ***atp6*** | 54^p^ | 45 | 9 | u | u | u | u | u | u | 52 | 22 | 36 | -14 |
| ***atp8*** | 15 | 14 | 1 | u | u | u | 0 | 6 | -6 | 21 | 12 | 14 | -4 |
| ***atp9*** | 14 | 12 | 2 | u | u | u | 0 | 0 | 0 | 12 | 0 | 15 | -15 |
| ***ccmB*** | 36^p^ | 35 | 1 | u | u | u | u | u | u | 42 | 3 | 42 | -39 |
| ***ccmC*** | 8 | 43 | -35 | 29^p^ | 31 | -2 | u | u | u | 52 | 9 | 37 | -28 |
| ***ccmFc*** | u | u | u | u | u | u | 3 | 6 | -3 | 40 | 25 | 40 | -20 |
| ***ccmFn*** | u | u | u | 22^p^ | 21 | 1 | u | u | u | 60 | 30 | 72 | -51 |
| ***cob*** | 54 | 53 | 1 | u | u | u | 0 | 3 | -3 | 55 | 25 | 50 | -28 |
| ***cox1*** | 11^p^ | 9 | 2 | 1^p^ | 3 | -2 | 0 | 2 | -2 | 76 | 39 | 61 | -22 |
| ***cox2*** | u | u | u | 28^p^ | 26 | 2 | u | u | u | 17 | 24 | 25 | -3 |
| ***cox3*** | 29 | 29 | 0 | 40 | 40 | 0 | 0 | 1 | -1 | 36 | 20 | 16 | 4 |
| ***matR*** | u | u | u | u | u | u | 5 | 17 | -12 | 31 | 14 | 40 | -26 |
| ***mttB*** | 53 | 45 | 8 | 51^p^ | 41 | 10 | u | u | u | 46 | 8 | 54 | -46 |
| ***nad1*** | u | u | u | 57^p^ | 52 | 5 | u | u | u | 29 | 36 | 47 | -15 |
| ***nad2*** | u | u | u | 79^p^ | 66 | 13 | 0 | 2 | -2 | 52 | 28 | 65 | -38 |
| ***nad3*** | 30 | 29 | 1 | 40 | 37 | 3 | u | u | u | 27 | 9 | 18 | -10 |
| ***nad4*** | u | u | u | 95^p^ | 84 | 11 | 0 | 1 | -1 | 84 | 45 | 89 | -52 |
| ***nad4L*** | 17 | 16 | 1 | u | u | u | 0 | 4 | -4 | 20 | 10 | 23 | -13 |
| ***nad5*** | u | u | u | 74^p^ | 67 | 7 | 1 | 4 | -3 | 83 | 21 | 76 | -55 |
| ***nad6*** | u | u | u | u | u | u | u | u | u | 43 | 3 | 49 | -47 |
| ***nad7*** | u | u | u | 55 | 44 | 11 | 7 | 8 | -1 | 37 | 21 | 31 | -10 |
| ***nad9*** | 24 | 18 | 6 | u | u | u | u | u | u | 19 | 10 | 23 | -13 |
| ***rpl2*** | u | u | u | u | u | u | t | t | t | 24 | t | t | t |
| ***rpl5*** | 12 | 19 | -7 | u | u | u | t | t | t | 18 | 11 | 21 | -10 |
| ***rpl10*** | u | u | u | u | u | u | 0 | 6 | -6 | 14 | t | t | t |
| ***rpl16*** | 13 | 11 | 2 | 10^p^ | 8 | 2 | t | t | t | 12 | 6 | 5 | -2 |
| ***rps1*** | 17 | 10 | 7 | u | u | u | t | t | t | 21 | t | t | t |
| ***rps2*** | 17 | 14 | 3 | u | u | u | t | t | t | 22 | t | t | t |
| ***rps3*** | u | u | u | 35^c^ | 36 | -1 | u | u | u | 31 | 23 | 30 | -18 |
| ***rps4*** | u | u | u | u | u | u | 4 | 9 | -5 | 42 | 24 | 27 | -9 |
| ***rps7*** | 10 | 14 | -4 | u | u | u | t | t | t | 17 | t | t | t |
| ***rps10*** |  |  |  | u | u | u | t | t | t | 11 | t | t | t |
| ***rps11*** | 14 | 5 | 9 | u | u | u | t | t | t | 6 | t | t | t |
| ***rps12*** | 16 | 15 | 1 | u | u | u | u | u | u | 13 | 12 | 8 | 1 |
| ***rps13*** | 8 | 10 | -2 | u | u | u | t | t | t | 10 | 12 | 7 | -1 |
| ***rps14*** | 8 | 10 | -2 | u | u | u | t | t | t | 12 | t | t | t |
| ***rps19*** | 11 | 10 | 1 | 15^c^ | 15 | 0 | t | t | t | 12 | 7 | 10 | -3 |
| ***sdh3*** | 27 | 28 | -1 | u | u | u | t | t | t | 25 | t | t | t |
| ***sdh4*** | u | u | u | u | u | u | 0 | 2 | -2 | 10 | 10 | 12 | -5 |
| **Total** | 534 | 526 | 8 | 659 | 598 | 61 | 20 | 73 | -53 | 1261 | 582 | 1102 | -520 |

A, data from Salmans et al. (2010); b, data from Guo et al. (2016); c, data from Regina and Quagliariello (2010); d, data from Funk et al. (2016) and Fan et al. (2019); p, partial cDNA; u, unvalidated by RT-PCR; t, genes have transferred to the nuclear genome or lost.
